# Engineered macrophages as near-infrared light activated drug vectors for chemo-photodynamic therapy of primary and bone metastatic breast cancer

**DOI:** 10.1038/s41467-021-24564-0

**Published:** 2021-07-14

**Authors:** Yanjuan Huang, Zilin Guan, Xiuling Dai, Yifeng Shen, Qin Wei, Lingling Ren, Jingwen Jiang, Zhanghong Xiao, Yali Jiang, Di Liu, Zeqian Huang, Xiaoyu Xu, Yong Luo, Chunshun Zhao

**Affiliations:** School of Pharmaceutical Sciences, Sun Yat-sen University, Guangzhou, People’s Republic of China

**Keywords:** Bone cancer, Breast cancer, Bone metastases

## Abstract

Patients with primary and bone metastatic breast cancer have significantly reduced survival and life quality. Due to the poor drug delivery efficiency of anti-metastasis therapy and the limited response rate of immunotherapy for breast cancer, effective treatment remains a formidable challenge. In this work, engineered macrophages (Oxa(IV)@ZnPc@M) carrying nanomedicine containing oxaliplatin prodrug and photosensitizer are designed as near-infrared (NIR) light-activated drug vectors, aiming to achieve enhanced chemo/photo/immunotherapy of primary and bone metastatic tumors. Oxa(IV)@ZnPc@M exhibits an anti-tumor M1 phenotype polarization and can efficiently home to primary and bone metastatic tumors. Additionally, therapeutics inside Oxa(IV)@ZnPc@M undergo NIR triggered release, which can kill primary tumors via combined chemo-photodynamic therapy and induce immunogenic cell death simultaneously. Oxa(IV)@ZnPc@M combined with anti-PD-L1 can eliminate primary and bone metastatic tumors, activate tumor-specific antitumor immune response, and improve overall survival with limited systemic toxicity. Therefore, this all-in-one macrophage provides a treatment platform for effective therapy of primary and bone metastatic tumors.

## Abstract (web summary)

Bone metastases are associated with poor prognosis in patients with breast cancer and limited therapeutic options. Here the authors exploit near-infrared light responsive macrophages for the tumor-selective delivery of oxaliplatin prodrug for chemo-photodynamic therapy of primary and bone metastatic breast cancer.

## Introduction

Breast cancer is the leading cause of cancer deaths in women worldwide, and >70% of patients have evidence of bone metastasis^1–3^, which induces life-threatening skeletal-related events that severely reduce survival and life quality^4–6^. Surgical resection can remove the primary tumor, but it hardly means healing, secondary metastases and recurrence usually occur. Meanwhile, due to high-frequency micrometastatic nodules, simple surgery and locoregional irradiation can hardly eliminate bone metastatic tumor cells completely. Chemotherapy has evolved as a cornerstone for primary and bone metastasis treatment, but it is also limited by poor tumor specificity and chemo-protective natures^7,8^ of the bone microenvironment. Consequently, effective treatment is often accompanied by severe dose-limiting toxicity. Therefore, therapeutic strategies that can efficiently and simultaneously eliminate both primary and bone metastatic tumors to improve therapeutic specificity and efficacy are urgently needed.

In recent years, immunotherapy that stimulates the host’s immune system to elicit promising antitumor immune responses has ushered a new chapter in treating malignant tumors^9–11^. Despite promise, only a fraction of cancer patients benefit from immunotherapy, and the lack of tumor specificity causes specific immunotoxicity in a considerable subset of patients receiving treatment^12–17^. Photodynamic therapy (PDT) that kills cancer cells by utilizing non-toxic photosensitizers, light and oxygen to generate cytotoxic reactive oxygen species (ROS), is an effective, spatiotemporal controllable and minimally invasive therapeutic modality^18^. In addition, PDT-induced tumor cell debris can be served as a source of tumor-associated antigens (TAAs) to stimulate antitumor immune responses^19,20^. This form of cell death is called immunogenic cell death (ICD) and is characterized by the release of TAAs and danger-associated molecular patterns (DAMPs), which are captured by dendritic cells (DCs) and macrophages, then processed and presented to adaptive immune cells, stimulating an antigen-specific immune response toward a broad-spectrum of solid tumors^13,19^. Recently, some chemotherapeutics, such as oxaliplatin (Oxa), doxorubicin, and mitoxantrone have also been demonstrated to induce ICD^21,22^. ICD induction served as “in situ vaccines” can be an intriguing strategy that can safely and effectively stimulate an immunogenic hot tumor microenvironment to improve current immunotherapy outcomes. However, due to low drug delivery efficiency, PDT and chemotherapy alone cannot completely destroy the tumors, and the induced ICD is not yet robust enough to generate sufficient immune responses to efficiently prohibit tumor growth and metastasis. Therefore, effective and accurate delivery of cytotoxic agents or ICD-inducers are extremely important for improved therapeutic effect.

Nano-based drug-delivery carriers and targeted systems have been widely used to improve tumor targeting abilities, but they are only effective in well-vascularized tumors^23–25^. However, well-vascularized primary tumors usually have tortuous vasculatures and elevated hydrostatic pressure, resulting in poor penetrability. While bone metastatic tumors, especially multiple micrometastatic nodules, are limited in well-built vasculature, leading to inadequate drug concentration in metastatic tumor to fully realize the synchronous therapeutic potential^24,26,27^. Meanwhile, bone-seeking molecules (e.g. bisphosphonate) functionalized nanocarriers mainly focused on delivering drugs to bone metastatic site, making it difficult to treat primary tumors simultaneously. Furthermore, reported data suggest that only a tiny percentage of the intravenous injected nanoparticles (0.7%, median) actually reach the tumors after experiencing numerous biological barriers, especially the clearance by the reticuloendothelial system (RES)^28,29^. The distinct specificities of the primary and metastatic tumors, and the limited targeting efficiency of nanocarries aggravated the difficulty of drug carriers’ design and efficient therapy.

To circumvent these limitations, an emerging strategy is to use body’s own circulatory cells as drug delivery vehicles^30^. In recent years, cell-based systems, such as erythrocytes^31,32^, platelets, stem cells, monocytes/macrophages, lymphocytes, dendritic cells, neutrophils, have emerged as interesting alternatives to biological drug carriers^33^. These cell systems possess numerous advantages including prolonged blood circulation time, tumor-tropic property, improved biocompatibility and inherent biodegradability^34^. Recently, a number of drug delivery candidates based on erythrocytes, dendritic cells, and stem cells have emerged in clinical trials^34,35^. In addition, chimeric antigen receptor (CAR) T cell therapy as a potential clinical treatment is now being exploited in several hematologic and solid tumor types^36^. Macrophages, a kind of immunocytes-based novel drug delivery systems, can sense cancer-related chemokine and cytokine cues, actively transmigrate into the tumor tissues, overcome various biological barriers and evade unwanted immune reactions^37–40^. Inspired by natural trafficking activity, impressive phagocytosis and stealth properties, macrophages would be a potentially appealing vehicle (Trojan Horse) for selective delivery of therapeutic agents to both primary and bone-metastatic nodules. Importantly, compared with nanoparticles constructed from non-degradable inorganic and organic materials, live-cell-based delivery systems have unparalleled biocompatibility.

**Fig. 1:**
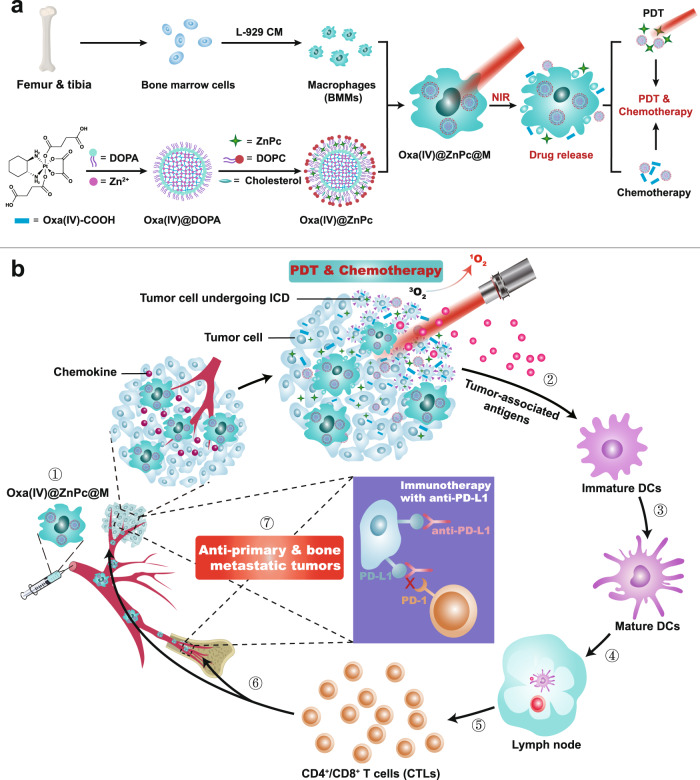
The preparation and mechanism of the artificially engineered macrophages. **a** Schematic illustration of the preparation of Oxa(IV)@ZnPc@M. **b** The mechanism of Oxa(IV)@ZnPc@M mediated chemo-photodynamic therapy to trigger robust antitumor immune responses and potentiate PD-L1 blockade immunotherapies for anti-primary and bone metastatic tumors. Photodynamics therapy (PDT), immunogenic cell death (ICD), Dendritic cells (DCs). ① Intravenous injection and tumor distribution of Oxa(IV)@ZnPc@M, ② PDT and chemotherapy of primary tumor to induce ICD and the release of tumor-associated antigens, ③ ④ DCs maturation and antigen presentation, ⑤ ⑥ CTLs traffic to tumors, and ⑦ Inhibition of primary and bone metastatic tumors simultaneously combined with anti-PD-L1.

In this work, we report on macrophage-mediated and light-triggered accurate delivery of cytotoxic agents that effectively kill cancer cells and generate “in situ vaccines” simultaneously, achieving enhanced chemo/photo/immunotherapy of primary and bone metastatic breast cancers when combined with checkpoint blockade (Fig. 1). Oxa(IV)@ZnPc is constructed by cross-linking Zn^2+^ with the carboxyl group of Oxa(IV)-COOH, a prodrug of Oxa, to form the core, and photosensitizer Zinc phthalocyanine (ZnPc) is concurrently loaded in the lipid bilayer via hydrophobic interaction. To improve tumor tropism capacity, Oxa(IV)@ZnPc is further loaded into bone marrow-derived macrophages (BMMs) to prepare near-infrared (NIR) light-activated macrophages (Oxa(IV)@ZnPc@M). Oxa(IV)@ZnPc@M shows high drug loading with unimpaired cellular functions. Meanwhile, drug loading polarizes macrophages to the antitumor M1 phenotype, giving macrophages the potential to exert antitumor effects themselves. Oxa(IV)@ZnPc@M has a superior pharmacokinetics profile and can efficiently home to the primary and bone metastatic tumors. In addition, therapeutic agent inside Oxa(IV)@ZnPc@M undergoes NIR-triggered release, then effectively kills primary tumors via combined chemo-photodynamic therapy, and induces ICD simultaneously to potentiate the antitumor immune response. In particular, Oxa(IV)@ZnPc@M mediated chemo-photodynamic therapy combined with anti-PD-L1 can effectively eliminate the primary and bone metastatic tumors, and boost the tumor-infiltration of cytotoxic CD8^+^ and CD4^+^ T cells with little systemic toxicity in two breast cancer models. In summary, the artificially engineered macrophages hold considerable promise as controlled drug reservoirs and ICD-inducing carriers to achieve synchronous chemo/photo/immunotherapy of primary and bone metastatic breast cancer.

## Results

### Preparation and characterization of Oxa(IV)@ZnPc

The core of nanoparticles (Oxa(IV)@DOPA) was prepared by cross-linking Zn^2+^ with the carboxyl groups of the prodrug Oxa(IV)-COOH (Supplementary Figs. 1–6), which was further stabilized by DOPA via Zn-phosphate interactions. The fourier transform infrared spectroscopy (FTIR) spectrum of Oxa(IV)@DOPA showed the characteristic peaks of Oxa(IV)-COOH, the disappearance of 1740 cm^−1^ peak of DOPA, and the blue shift of P=O and P–O–C peaks of DOPA to 1068 and 997 cm^−1^, respectively, indicated the successful coating of DOPA via coordination interaction (Supplementary Fig. 7). Results from x-ray photoelectron spectroscopy (XPS) (Supplementary Fig. 8) confirmed the surface of Oxa(IV)@DOPA contains Zn, P, Pt, and N atoms. ZnPc, a photosensitizer, was encapsulated into the outer layer of lipid via hydrophobic interactions to form core-shell nanoparticles Oxa(IV)@ZnPc.

**Fig. 2:**
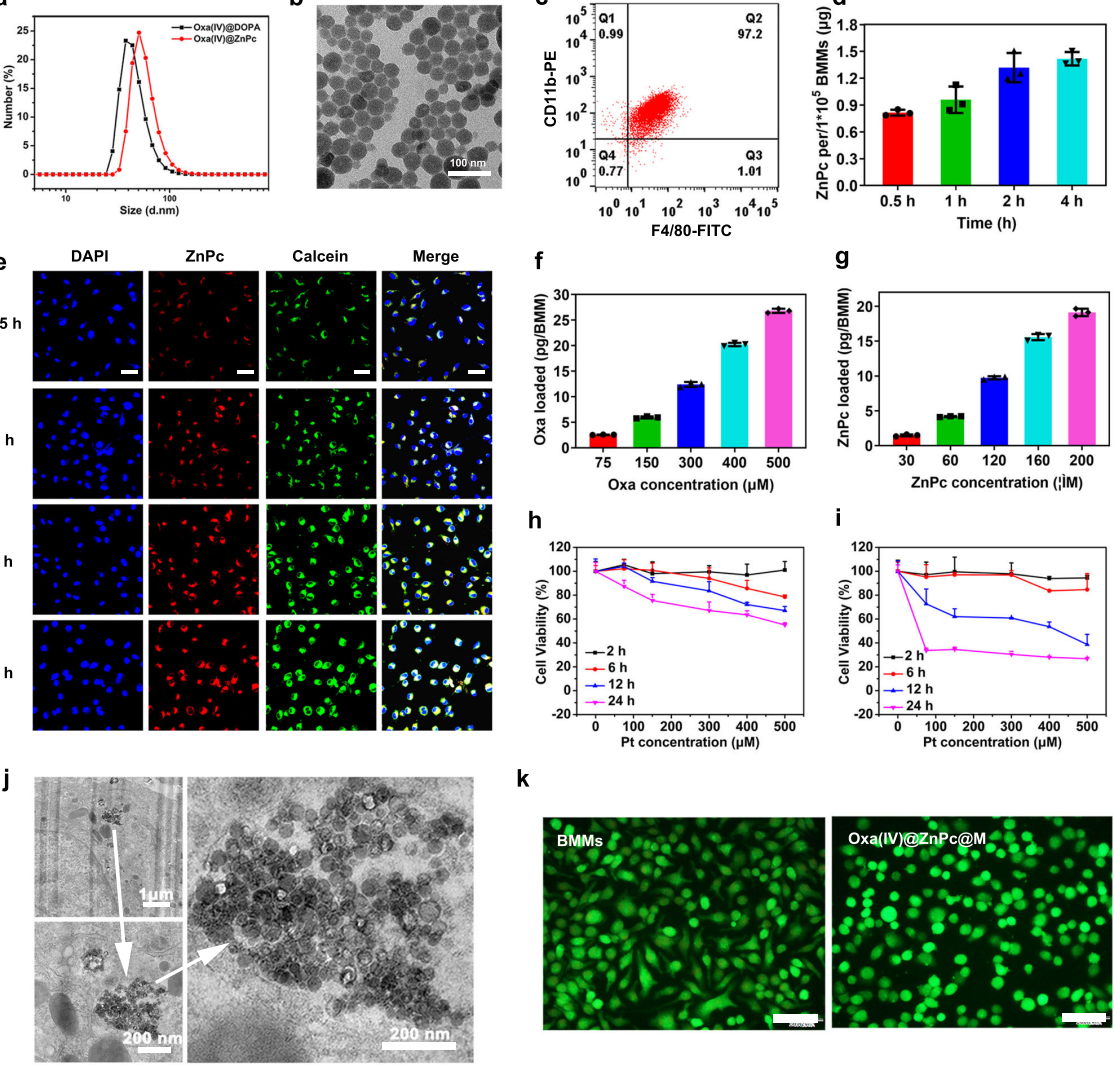
The preparation and characterization of Oxa(IV)@ZnPc and Oxa(IV)@ZnPc@M. **a** Average diameters of Oxa(IV)@DOPA and Oxa(IV)@ZnPc measured by DLS. **b** TEM image of Oxa(IV)@ZnPc, this experiment was performed three times with similar results. **c** Flow cytometric analysis of the purity of BMMs cultured in L929-CM doubly stained with F4/80-FITC and CD11b-PE. Cellular uptake by BMMs (**d**) quantitatively determined by fluorescence spectra (*n* = 3 technical replicates, the experiment is representative of three independent experiments) and **e** observed by CLSM at 0.5, 1, 2, and 4 h incubation with Oxa(IV)@ZnPc and Cal@ZnPc nanoparticles, respectively, *n* = 3 technical replicates, the experiment is representative of two independent experiments. Scale bars = 30 µm. **f** Intracellular Oxa contents (**g**) ZnPc contents at 2 h incubation with different concentrations of Oxa(IV)@ZnPc (*n* = 3 technical replicates, the experiment is representative of three independent experiments). Cell viability at 2 h, 6 h, 12 h, and 24 h via CCK8 assay after incubation with **h** Oxa(IV)@ZnPc and **i** free Oxa at different platinum concentrations for 2 h (*n* = 3 technical replicates, the experiment is representative of three independent experiments). **j** Cellular uptake by BMMs observed by TEM at 2 h incubation with Oxa(IV)@ZnPc, this experiment was performed one time. **k** Live/Dead cell staining results of BMMs and Oxa(IV)@ZnPc@M at 2 h after drug loading, this experiment was performed two times with similar results. Scale bars = 50 µm. All data were presented as mean ± SD. Source data are provided as a Source data file.

The average sizes of Oxa(IV)@DOPA and Oxa(IV)@ZnPc were ~46 nm and 58 nm, respectively (Fig. 2a). Transmission electron microscopy (TEM) images of Oxa(IV)@ZnPc showed well-dispersed uniform spherical nanoparticles with diameter ~40 nm (Fig. 2b). ZnPc in H_2_O had no UV-vis and fluorescence absorption due to molecular aggregation behavior (Supplementary Fig. 9a, b), while Oxa(IV)@ZnPc in H_2_O had the same UV-vis and fluorescence spectrum as ZnPc in DMSO, with characteristic monomer absorption and fluorescence emission peaks at 672 nm and 678 nm respectively, indicating that ZnPc was finely dispersed in Oxa(IV)@ZnPc, which enabled a strong ability to generate ^1^O_2_, as evidenced by the reaction with SOSG (Supplementary Fig. 9c). Oxa(IV)@ZnPc showed near-neutral zeta potential at about −7 mV, and exhibited negligible particle size change for 1 week incubation, indicating good stability in physiological conditions (Supplementary Fig. 9d).

The release of platinum from Oxa(IV)@ZnPc was in a pH dependent manner, with <20% released at pH 7.4 within 12 h, while a significantly increased release at pH 5.5 was observed (Supplementary Fig. 10a). This was attributed to the formation of the characteristic acid-cleavable coordination bonds in the core of Oxa(IV)@ZnPc. Free ZnPc is an extremely hydrophobic photosensitizer, it can easily accumulate in the aqueous solution, resulting in UV-vis and fluorescence quenching (Supplementary Fig. 9a, b). Therefore, it is hard to investigate the drug release behavior of ZnPc. We tried to use PBS buffer containing 2% tween 80 as release medium, the release of ZnPc from Oxa(IV)@ZnPc was slow under the current release medium. The drug release rate at pH 5.5 was slightly higher than pH 6.5 and pH 7.4 (Supplementary Fig. 10b), this might be due to that the acid-cleavable coordination bonds in the core of Oxa(IV)@ZnPc could fracture in acidic conditions, leading to the decomposition of nanoparticles and the re-assembly of the outer lipid.

The characterization of fluorescent model nanoparticle Cal@ZnPc, and the control nanoparticle SPP@ZnPc for single PDT effect were shown in Supplementary Fig. 11. The average sizes of SPP@DOPA and Cal@DOPA detected by DLS were ~32 nm, and the average sizes of SPP@ZnPc and Cal@ZnPc were ~43 nm (Supplementary Fig. 11a, b). TEM images of SPP@ZnPc and Cal@ZnPc were showed well-dispersed uniform spherical nanoparticles (Supplementary Fig. 11c). Both SPP@ZnPc and Cal@ZnPc were showed near-neutral zeta potential at ~−7 mV (Supplementary Fig. 11d). The drug loading of Zn in SPP@ZnPc was detected by Inductively Coupled Plasma-Atomic Emission Spectrometry (ICP-AES) and calculated to be ~14.7%, and the drug loading of Cal in Cal@ZnPc was detected by fluorescence spectrometer and calculated to be ~28.1%. The UV-vis and fluorescence spectrums of SPP@ZnPc were similar to that of Oxa(IV)@ZnPc (Supplementary Fig. 11e, f), suggesting that ZnPc was successfully loaded and finely dispersed in the lipid bilayer. The UV-vis and fluorescence spectrums of Cal@ZnPc showed the UV-vis and fluorescence characteristic peaks of calcein and ZnPc (Supplementary Fig. 11g–i). All of these results suggested the successfully preparations of SPP@ZnPc and Cal@ZnPc.

### In vitro chemo-photodynamic therapy of Oxa(IV)@ZnPc

Oxa(IV)@ZnPc showed time-dependent cellular uptake (Supplementary Fig. 12). Oxa(IV)@ZnPc’s ability to generate ROS intracellular was further confirmed by confocal laser scanning microscopy (CLSM) using fluorescence probe 2′,7′-Dichlorofluorescein diacetate (DCFH-DA) (Supplementary Fig. 13). Oxa(IV)@Lip (lipid coated but without ZnPc loading) displayed lower cytotoxicity than free Oxa (half maximal inhibitory concentration (IC_50_) ~ 3.407 μM) toward 4T1 cells, possibly due to the time-consuming drug release behavior (Supplementary Fig. 14a, b). Oxa(IV)@Lip and Oxa(IV)@ZnPc exhibited similar cytotoxicity without light, indicating ZnPc was non-toxic in dark. As a control nanoparticle for single PDT, SPP@ZnPc (using low-toxic sodium pyrophosphate (SPP) to replace Oxa prodrug) had no cytotoxicity under dark, but showed high cytotoxicity after irradiation, with IC_50_ ~ 0.075 μM. However, Oxa(IV)@ZnPc exhibited the highest cytotoxicity effect under irradiation, the IC_50_ of platinum and ZnPc were 0.476 μM and 0.048 μM, respectively, and the calculated cooperativity index (CI) value was 0.78 (0.476/3.407 + 0.048/0.075 = 0.78), below 1, suggesting a synergistic chemo-photodynamic therapeutic effect^41^. Live/dead staining and apoptosis results were consistent with that of MTT (Supplementary Fig. 14c, d). Oxa and Oxa(IV)@ZnPc showed less amount of live cells (green fluorescence) and a slightly increased dead cells (red fluorescence) compared with Control (+) group, because free Oxa and Oxa released from Oxa(IV)@ZnPc can inhibit tumor cell proliferation. Oxa(IV)@ZnPc with irradiation displayed the largest amount of dead cells and almost none live cells. In addition, Oxa(IV)@ZnPc with light induced the highest apoptotic cells (79.8%), which was higher than single chemotherapy (38.2%) and PDT (66.1%).

Cell-surface exposure of calreticulin (CRT), secretion of adenosine triphosphate (ATP), and release of high mobility group box 1 protein (HMGB-1) are characteristic biomarkers of cells undergoing ICD^19^. CLSM observation and quantitative flow cytometry detection showed that unirradiated Oxa and Oxa(IV)@ZnPc could induce CRT exposure (Supplementary Fig. 15a, b). SPP@ZnPc only induced CRT expression under irradiation, indicating that PDT process can induce ICD. Oxa(IV)@ZnPc (+) exhibited higher green fluorescence than Oxa and SPP@ZnPc (+), suggesting a stronger capacity to induce CRT expression. Meanwhile, the highest ATP secretion and HMGB-1 release in Oxa(IV)@ZnPc (+) were also detected (Supplementary Fig. 15c, d). These results supported synergistic chemo-photodynamic therapy of Oxa(IV)@ZnPc to induce ICD in 4T1 cells.

### Preparation and characterization of Oxa(IV)@ZnPc@M

BMMs were isolated from mouse bone marrow and cultured with DMEM containing L929 cell-conditioned media (L929-CM) or commercial Macrophage-Colony Stimulating Factor (M-CSF) for 6 or 7 days. The CD11b^+^F4/80^+^ phenotype cells were quantified to be higher than 95% (Fig. 2c and Supplementary Fig. 16a), and no difference was observed between the two media, the former was used in subsequent studies.

To optimize the incubation time for nanoparticles loading, firstly, whether core-shell nanoparticles (Oxa(IV)@ZnPc) could integrally enter the cells were verified, and Cal@ZnPc (using fluorescent dye calcein to replace Oxa prodrug) was selected as the model nanoparticle. Cal@ZnPc was rapidly taken up by BMMs for its intrinsic phagocytosis ability, incubation for 2 h leading to significant amount of cellular uptake, while extending the incubation time to 4 h only further increased the cellular uptake for a little bit (Fig. 2e). In addition, quantitative determination of ZnPc content per 1 × 10^5^ BMMs also verified that 2 h incubation was efficient (Fig. 2d). Therefore, 2 h incubation was selected to load the nanoparticles. Subsequently, different concentrations of Oxa(IV)@ZnPc were incubated with BMMs for 2 h, and the loading amounts of platinum and ZnPc per BMM were seemingly concentration dependent (Fig. 2f, g). After 2 h of incubation, the drug medium was replaced with fresh medium, and then further incubated for 2 h, 6 h, 12 h, and 24 h, and cell viability was determined using the CCK8 kit. For comparison, the cytotoxicity of free Oxa and ZnPc@Lip were performed. ZnPc@Lip without light had negligible cytotoxicity, indicating the cytotoxicity of Oxa(IV)@ZnPc to BMMs was induced by platinum (Supplementary Fig. 16b). When the initial platinum concentration was 400 μM, cell viability was >80% at 6 h, and maintained at ~70% or higher at 12 h, while increasing incubation concentration to 500 μM, cell viability was <70% at 12 h (Fig. 2h). Meanwhile, under the same incubation concentration, free Oxa exhibited significant cytotoxicity than Oxa(IV)@ZnPc at 6 h, 12 h, and 24 h incubation (Fig. 2i). Based on drug loading and cell viability, Oxa(IV)@ZnPc with platinum concentration 400 μM and ZnPc concentration 160 μM were chosen for drug loading. Under these conditions, BMMs had high drug loading, with approximately 20 pg Oxa and 15 pg ZnPc per cell (Fig. 2f, g). TEM image showed a large number of Oxa(IV)@ZnPc within BMMs (Fig. 2j), further verifying the high-efficient phagocytosis capacity of BMMs. Live/dead cell staining assay displayed that almost all Oxa(IV)@ZnPc loaded BMMs were alive (green fluorescence) after 2 h of drug incubation (Fig. 2k). These results indicated that compared with direct loading free drug, loading nanoparticles constructed by prodrug could not only increase drug loading but also reduce cytotoxicity to carrier cells in a short time, thereby saving valuable time for tumor tropism.

### Cell migration assay

**Fig. 3:**
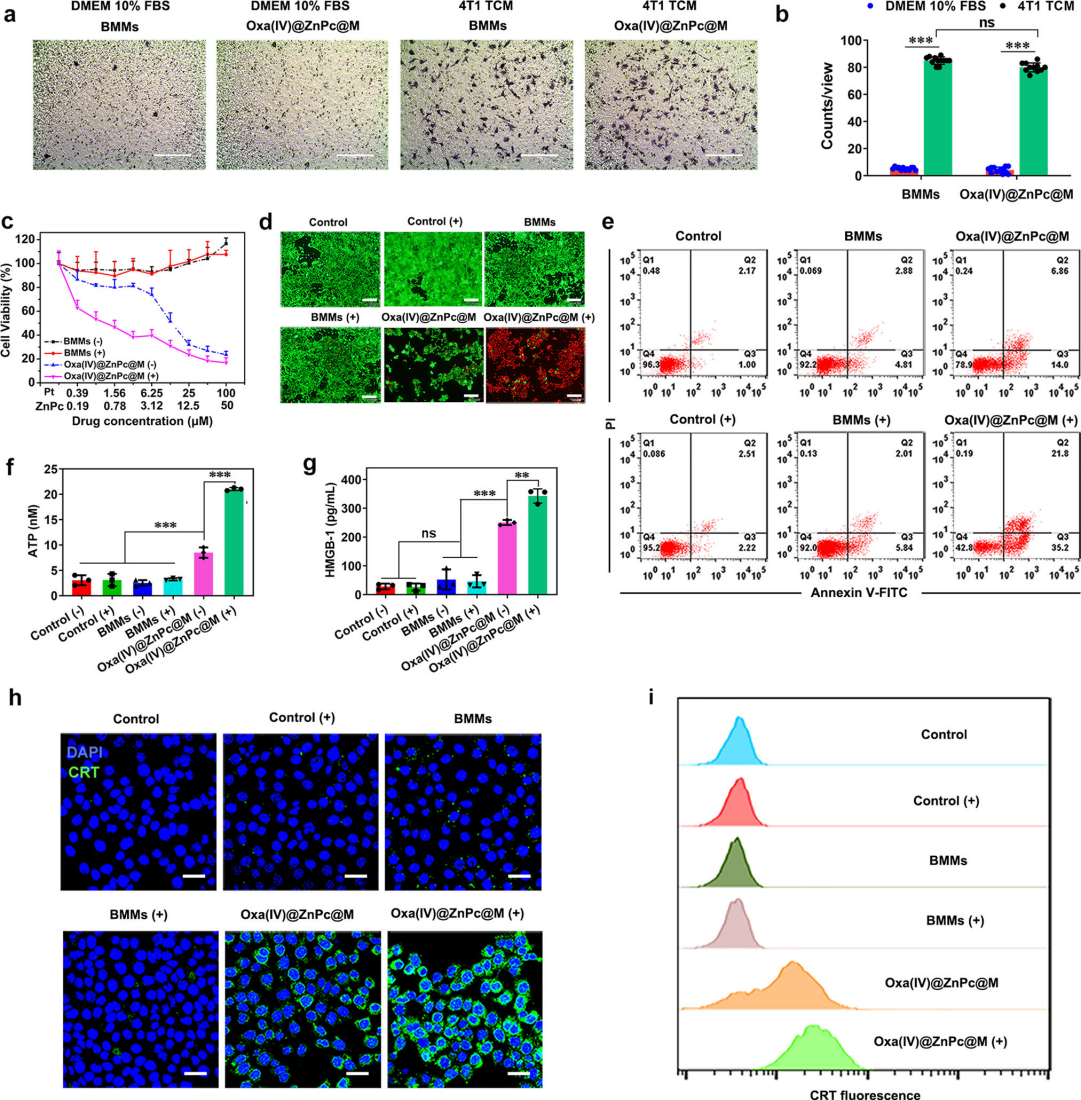
The tumor tropism, cell killing, and ICD induction ability of Oxa(IV)@ZnPc@M in vitro. **a** Optical microscopy images of migratory BMMs or Oxa(IV)@ZnPc@M stained by crystal violet using a transwell system with cell growth medium (DMEM 10% FBS) or 4T1 TCM added in the bottom chamber. Scale bars = 200 µm. **b** Quantitative evaluation of migratory cells per representative view (*n* = 12 technical replicates, ***p < 0.0001), the experiment is representative of three independent experiments. **c** Cell viability (*n* = 3 technical replicates, the experiment is representative of three independent experiments) and **d** live/dead cell staining assay of 4T1 cells after treatment by BMMs and Oxa(IV)@ZnPc@M with or without light irradiation, this experiment was performed two times with similar results. Scale bars = 200 µm. **e** Quantitative apoptosis analysis of 4T1 cells stained with Annexin V-FITC/PI. Quantitative determination of **f** ATP secretion (***p < 0.0001, ***p < 0.0001) and **g** HMGB-1 release of 4T1 cells after various treatment (*n* = 3 technical replicates, the experiment is representative of three independent experiments, ***p < 0.0001, **p = 0.0017). **h** CLSM images (this experiment was performed two times with similar results) and **i** Flow cytometric examination of CRT exposure on the surface of 4T1 cells (the experiment is representative of three independent experiments). Scale bars = 30 µm. All data were presented as mean ± SD. Statistical significance was calculated via the unpaired two-tailed *t* test b or ordinary one-way ANOVA with a Tukey’s test **f**, **g**. “ns” represents not significant, and *p < 0.05, **p < 0.01, ***p < 0.001. Source data are provided as a Source data file.

The tumoritropic migration experiment was conducted using a transwell system to investigate whether loading of Oxa(IV)@ZnPc, though not fatal, would impair the function of BMMs. The 4T1 tumor conditioned medium (TCM) was collected and added into the bottom chamber, BMMs and Oxa(IV)@ZnPc@M were separately seeded onto the upper chamber. Oxa(IV)@ZnPc@M could efficiently transmigrate the well in 4T1 TCM, with the migration cells counted to ~80 per view, slightly <~85 for untreated BMMs (Fig. 3a, b). Meanwhile, little cell transmigration in cell growth medium was observed. The result indicated that Oxa(IV)@ZnPc@M possessed favorable tumor tropism capacity similar to untreated BMMs, which is essential for cell-based delivery.

### Cytotoxicity, apoptosis and ICD induced by Oxa(IV)@ZnPc@M

The cytotoxicity against 4T1 cells was investigated by incubating Oxa(IV)@ZnPc@M with 4T1 cells. BMMs were non-cytotoxic to 4T1 cells with or without irradiation. Oxa(IV)@ZnPc@M showed moderate cytotoxicity without light, while a strikingly increased cytotoxicity was observed after irradiation, which could be explained by the combination effect of PDT by ZnPc and chemotherapy by released platinum (Fig. 3c). Live/dead cell staining and apoptosis assays further confirmed the result. Oxa(IV)@ZnPc@M with irradiation exhibited a large amount of dead cells (red fluorescence) (Fig. 3d), and a remarkable increase in apoptotic cells (57.2%) compared to the treatment under dark (21.1%) (Fig. 3e).

The induction of ICD in 4T1 cells by co-culture with Oxa(IV)@ZnPc@M was evaluated by measuring CRT exposure, ATP secretion, and HMGB-1 release. Oxa(IV)@ZnPc@M treated cells exhibited obvious ATP secretion and HMGB-1 release under dark. After irradiation, a significantly increased ATP secretion and HMGB-1 release were observed (Fig. 3f, g), indicating the stronger capacity of chemo-photodynamic therapy to induce ATP secretion and HMGB-1 release. Meanwhile, CLSM observation and quantitative flow cytometry detection also showed remarkably increased CRT exposure in Oxa(IV)@ZnPc@M (+) compared to Oxa(IV)@ZnPc@M (Fig. 3h, i). In contrast, BMMs incubation did not induce ICD with or without light, similar to the control group. These results revealed that platinum and ZnPc could be released from Oxa(IV)@ZnPc@M, which induced ICD by platinum under dark, and enhanced ICD induction under irradiation due to the combined effect of chemotherapy and PDT.

### In vitro drug release from Oxa(IV)@ZnPc@M

**Fig. 4:**
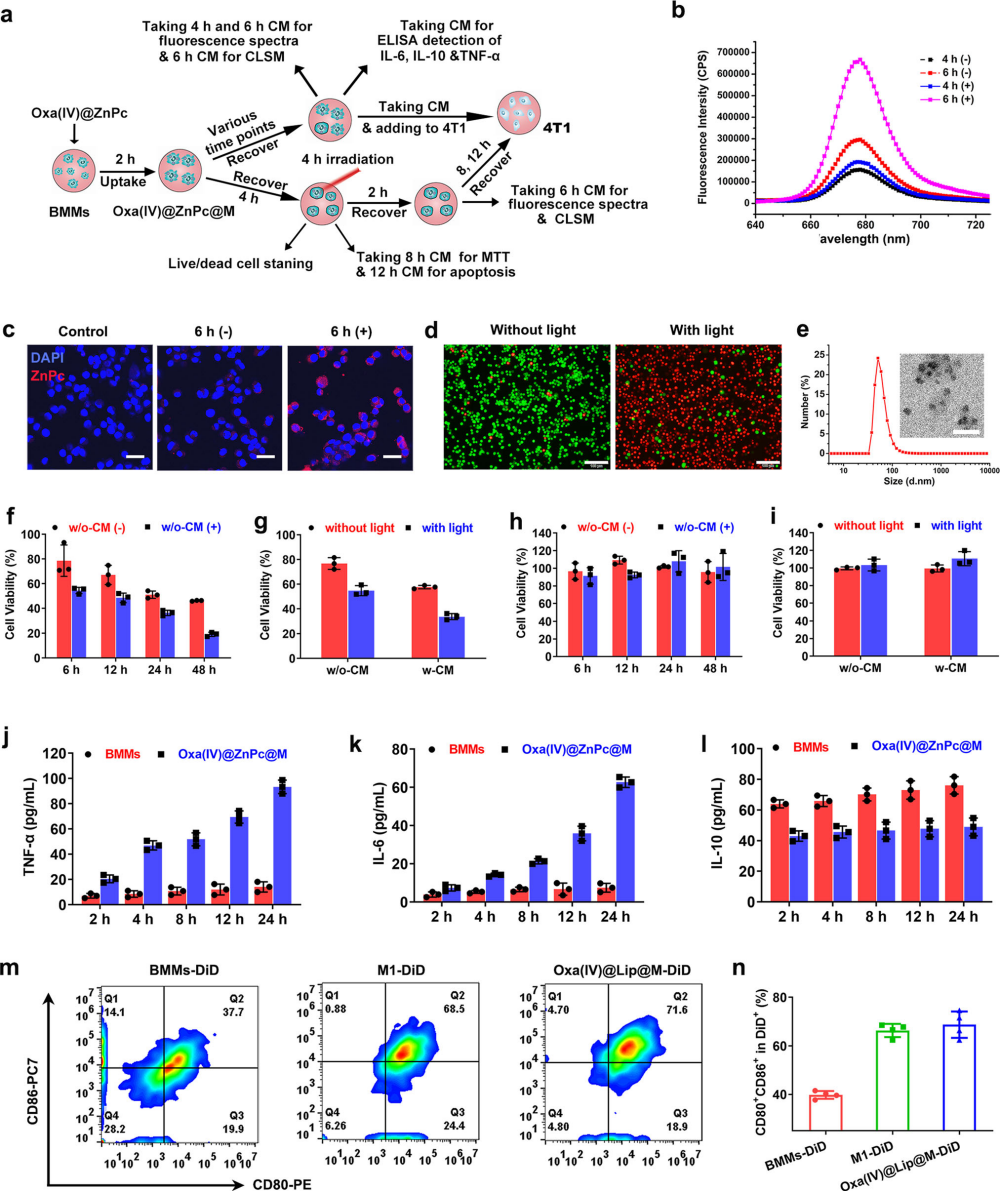
The in vitro drug release and cell phenotypes change of Oxa(IV)@ZnPc@M. **a** Schematic of the experimental design of cell phenotypes change and in vitro light-triggered drug release. **b** ZnPc fluorescence intensity of the 6 h (−) and the 6 h (+) supernatant. **c** CLSM observation of ZnPc fluorescence intensity of the 6 h (−) and 6 h (+) supernatant in 4T1 cells, this experiment was performed two times with similar results. Scale bars = 30 µm. **d** Live/dead cell staining of BMMs irradiated with LED light for 10 min at 4 h after Oxa(IV)@ZnPc loading, this experiment was performed two times with similar results. Scale bars = 100 µm. **e** Average size of CM taking from Oxa(IV)@ZnPc@M detected by DLS. An inset photograph of the TEM image. Scale bars = 100 nm. Cell viability of 4T1 cells treated with **f** w/o-CM, and **g** w-CM taken from Oxa(IV)@ZnPc@M (*n* = 3 technical replicates, the experiment is representative of three independent experiments). Cells were irradiated after 4 h cellular uptake and then recovered for an additional 4 h prior to harvest the w-CM. Cell viability of 4T1 cells treated with **h** w/o-CM and **i** w-CM taken from BMMs (*n* = 3 technical replicates, the experiment is representative of three independent experiments). Secretion of **j** TNF-α, **k** IL-6, and **l** IL-10 from Oxa(IV)@ZnPc@M (*n* = 3 technical replicates, the experiment is representative of three independent experiments). **m** Representative flow cytometric analysis of the expression of M1 marker (CD80, CD86). **n** Quantitative analysis of CD80^+^CD86^+^ cells in the DiD positive macrophages (*n* = 4 individual animals). All data were presented as mean ± SD. Source data are provided as a Source data file.

One major obstacle for cell-based drug delivery is the inefficient release of drug payloads from host cells. Therefore, the drug release from Oxa(IV)@ZnPc@M under physiological environment and NIR-triggered conditions were explored (Fig. 4a). The 4 h (−) group (without light irradiation) and 4 h (+) group (with light irradiation at 4 h) before light irradiation showed similar fluorescence intensity. In contrast, the 6 h (+) group, which received light irradiation at 4 h and left for additional 2 h, showed significantly higher fluorescence intensity than that of 6 h (−) group (Fig. 4b). CLSM observation further confirmed the result (Fig. 4c). The strong fluorescence signal of the conditioned medium (CM) indicated the existence of ZnPc dispersed in nanoparticles, which prevented aggregation induced fluorescence quenching (Fig. 4b, c). Meanwhile, drug release under physiological conditions was slow, with <30% released at 24 h. However, cells subjected to light displayed a sudden increase in drug release, with 48% released at 2 h after irradiation. The light triggered drug release might be explained by increased membrane permeability and cell membrane rupture induced by PDT^42,43^, as evidenced by the killing of host cells (Supplementary Fig. 17a), and the increase of the membrane permeability of PI (Fig. 4d). Intriguingly, the therapeutics in BMMs may not be completely released as free molecules, nanoparticles were observed when analyzing the CM of Oxa(IV)@ZnPc@M by dynamic light scattering (DLS) and TEM (Fig. 4e), which was consistent with the fluorescence emission spectrum of the CM (Fig. 4b).

Further, the supernatants taken from Oxa(IV)@ZnPc@M with (w-CM) or without (w/o-CM) light irradiation were incubated with 4T1 cells seeded on 96-well plates. The cytotoxicity of w/o-CM collected from different time points gradually increased with time, and light exposure further increased the cytotoxicity at all detected time points, indicating platinum and ZnPc might be released simultaneously (Fig. 4f). However, the 6 h and 12 h w/o-CM caused a slight decrease in cell viability, but the 24 h and 48 h w/o-CM showed enhanced cytotoxicity, which possibly ascribed to the delayed drug release from Oxa(IV)@ZnPc@M under physiological conditions. Notably, the cytotoxicity of w-CM showed a sharp increase no matter irradiation or not compared with that of w/o-CM, further confirming the light-triggered simultaneous release of platinum and ZnPc (Fig. 4g). The w-CM taking from Oxa(IV)@ZnPc@M induced 20.73% and 48.9% of apoptosis cells without or with light, respectively, which exhibited higher apoptosis cells than w/o-CM without (14.37%) or with (35.0%) light, respectively (Supplementary Fig. 17b). For comparison, these experiments were also conducted using the supernatants taken from untreated BMMs, and they were non-toxic to 4T1 cells (Fig. 4h, i). These results suggested that Oxa(IV)@ZnPc@M could remain relatively stable in physiological environment, with predominant drugs entrapped in cells. Once arriving at the target site and being exposed to light, the PDT process not only affected the host cells to trigger the acceleration release of therapeutics, but also directly killed tumor cells by the natural excreted drugs.

### Macrophage phenotype detection in vitro and in vivo

The impact of Oxa(IV)@ZnPc loading on BMMs’ phenotypic change in vitro was investigated by analyzing the secretion of cytokines, including tumor necrosis factor (TNF-α), interleukin-6 (IL-6), and interleukin-10 (IL-10) (Fig. 4a), and measuring the surface markers of macrophage (CD80, CD86). Compared with untreated BMMs, proinflammatory markers TNF-α and IL-6 displayed significantly enhanced secretion (Fig. 4j, k), by contrast, anti-inflammation maker IL-10 showed reduced secretion (Fig. 4l). For the detection of surface maker, typical LPS treated M1 macrophages and IL-4 treated M2 macrophages were served as controls, and the expression of iNOS (M1 maker) and Arg-1 (M2 maker) were verified by western blot (Supplementary Fig. 18). The population of CD80^+^CD86^+^ cells treated with Oxa(IV)@ZnPc showed similar to LPS treated M1 cells (Supplementary Fig. 19). These results suggested that after Oxa(IV)@ZnPc loading, BMMs could be polarized toward the proinflammation and antitumor M1 phenotypes^44,45^. In order to explore the reason for macrophage polarization, the effects of free ZnPc, free Oxa, ZnPc@Lip, and SPP@ZnPc were further compared. Free ZnPc, ZnPc@Lip, and SPP@ZnPc showed similar CD80^+^CD86^+^ cells to IL-4 treated M2 cells, while free Oxa with concentration 100 μM–2.5 μM showed similar CD80^+^CD86^+^ cells to LPS treated M1 cells (Supplementary Fig. 19), indicating that the polarization of macrophages may attributed to the released Oxa.

Next, whether the drug loaded macrophages could retain the M1 phenotype in tumor was further conducted. Cell membrane dye DiD was used to label macrophages. In order to eliminate the interference of the fluorescent dye ZnPc, which is non-toxic under dark, we used Oxa(IV)@Lip (lipid coated but without ZnPc loading) to replace Oxa(IV)@ZnPc for drug loading. The DiD labeled blank BMMs and drug loaded BMMs were named as BMMs-DiD and Oxa(IV)@Lip@M-DiD. LPS treated M1 BMMs (M1-DiD) were served as positive control. The population of CD80^+^CD86^+^ cells in DiD positive macrophages of Oxa(IV)@Lip@M were counted to be ~70%, which was similar to that of LPS treated M1 cells, while blank BMMs only counted to be ~37% (Fig. 4m, n), indicating that the drug loaded macrophages could retain their M1 phenotype in the tumor tissue.

### In vivo pharmacokinetics and biodistribution studies

**Fig. 5:**
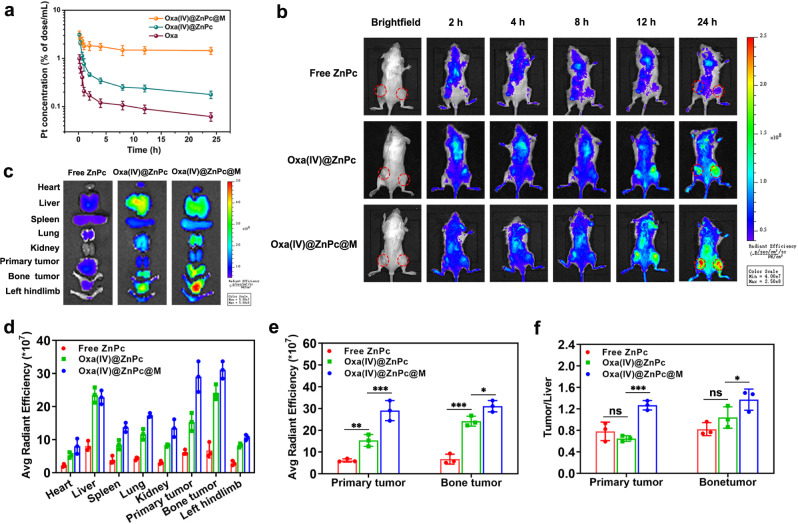
In vivo pharmacokinetics and tumor distribution. **a** Pharmacokinetics profiles in SD rats after intravenous injection of free Oxa, Oxa(IV)@ZnPc and Oxa(IV)@ZnPc@M (*n* = 3 individual animals). **b** Fluorescence imaging of free ZnPc, Oxa(IV)@ZnPc and Oxa(IV)@ZnPc@M distribution in primary tumors and bone metastatic tumors in 4T1 tumor-bearing mice. Red circle represents tumor area. **c** Ex vivo fluorescence imaging of the excised organs and tumors at 24 h post-injection. **d** Quantitative fluorescence intensity of the excised organs and tumors at 24 h post-injection (*n* = 3 individual animals). **e** Quantification fluorescence intensity of primary tumors and bone metastatic tumors (**p = 0.0042, ***p = 0.0002, ***p < 0.0001, *p = 0.0259). **f** Quantification fluorescence intensity of tumor: liver at 24 h post-injection (***p = 0.0007, *p = 0.0461). Red circle represents tumor area. All data were presented as mean ± SD. Statistical significance was calculated via two-way ANOVA with a Tukey’s test. “ns” represents not significant, and *p < 0.05, **p < 0.01, ***p < 0.001. Source data are provided as a Source data file.

For pharmacokinetics studies, SD rats were intravenously injected with free Oxa, Oxa(IV)@ZnPc and Oxa(IV)@ZnPc@M at an equal Oxa dose of 1 mg/kg. The platinum content in plasma was detected by graphite furnace atomic absorption spectrometer (GFAAS). Oxa(IV)@ZnPc@M showed elongated blood circulation time in comparison with Oxa(IV)@ZnPc and free Oxa and the platinum content was 4 times and 8.5 times higher than Oxa(IV)@ZnPc at 2 h and 24 h post-injection (Fig. 5a). In addition, Oxa(IV)@ZnPc@M showed ~3.9-fold longer blood clearance half-life (t_1/2_) and 17.5-fold higher area under curves (AUC) compared with Oxa(IV)@ZnPc respectively (Supplementary Table 1). Pharmacokinetics results indicated that loading nanoparticles into BMMs could camouflage nanoparticles to bypass the reticuloendothelial system (RES), thereby endowing a superior long blood circulation time and stealth properties in vivo.

The tumor tropism of Oxa(IV)@ZnPc@M was examined in bilateral 4T1-tumor-bearing mice using ZnPc as fluorescence tracer. Obvious distribution of Oxa(IV)@ZnPc@M in tumor site was observed at 2 h post-injection, while Oxa(IV)@ZnPc was obviously distributed at 8 h post-injection. In addition, Oxa(IV)@ZnPc and Oxa(IV)@ZnPc@M in both primary tumors and bone metastatic tumors were well retained up to 24 h post-injection, and the distribution of Oxa(IV)@ZnPc@M in tumor was higher than Oxa(IV)@ZnPc at all-time points (Fig. 5b). The ex vivo imaging and quantitative ROI analysis at 24 h post-injection further confirmed higher tumor distribution of Oxa(IV)@ZnPc@M than Oxa(IV)@ZnPc, with 1.9-fold and 1.3-fold higher fluorescence intensity in primary tumor and bone metastatic tumor, respectively (Fig. 5c–e). Meanwhile, the quantitative tumor-to-liver ratio of Oxa(IV)@ZnPc@M was significantly higher than Oxa(IV)@ZnPc (Fig. 5f). These results indicated that Oxa(IV)@ZnPc@M could effectively home to the primary and bone metastatic tumors. Oxa(IV)@ZnPc’s increase in tumor accumulation over ZnPc could be ascribed to enhanced penetration and retention effect, while Oxa(IV)@ZnPc@M’s better tumor targeting ability than Oxa(IV)@ZnPc might be explained by the superior pharmacokinetics and the intrinsic chemotactic migration ability of BMMs to primary and metastatic tumors^29,46^.

### In vivo antitumor efficiency

**Fig. 6:**
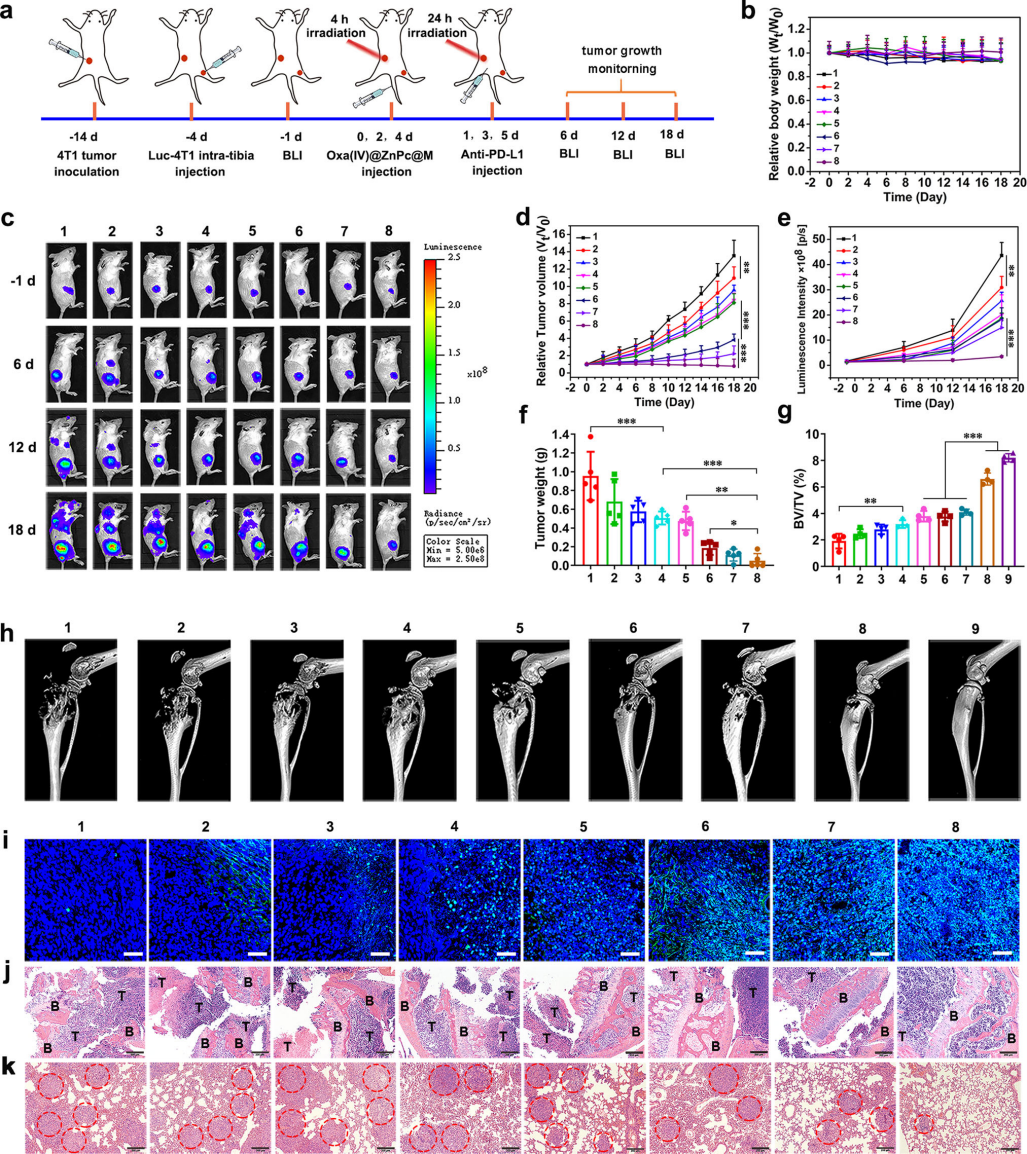
Therapeutic efficacy of Oxa(IV)@ZnPc@M combined with anti-PD-L1 for primary and bone metastatic breast cancer in 4T1-bearing mice. **a** Schematic illustration of experimental design. **b** Relative body weight after various treatments (1: PBS (+); 2: free Oxa; 3: Oxa(IV)@ZnPc; 4: Oxa(IV)@ZnPc@M; 5: BMMs + anti-PD-L1; 6: Oxa(IV)@ZnPc (+); 7: Oxa(IV)@ZnPc@M (+); 8: Oxa(IV)@ZnPc@M (+) + anti-PD-L1; 9: Normal, “(+)” represent laser irradiation, applied for all the antitumor studies in vivo) (*n* = 5 individual animals). **c** Representative in vivo bioluminescence images tracking the growth of bone metastatic tumors. **d** Relative tumor growth curves of primary tumors (*n* = 5 individual animals, **p = 0.0098, ***p < 0.0001, ***p = 0.0002). **e** Quantitative luminescence intensity curves of bone metastatic tumors (*n* = 4 individual animals, **p = 0.0024, ***p = 0.0005). **f** Average weight of ex vivo primary tumors (*n* = 5 individual animals, ***p = 0.0006, ***p = 0.0005, **p = 0.0013, *p = 0.0269). **g** The architecture parameters of BV/TV (*n* = 4 technical replicates based on calculation parameters from one of the representative mouse of each group, **p = 0.0015, ***p < 0.0001). **h** 3D micro-CT reconstruction images of bone metastatic tibias. **i** TUNEL staining of the primary tumors. Scale bar = 50 µm. **j** H&E staining of the tumor-bearing tibias and **k** the lung tissues, this experiment was performed one time. T represents tumor and B represents bone. Red circle represents tumor metastatic area. Scale bar = 200 µm. All data were presented as mean ± SD. Statistical significance was evaluated via one-way ANOVA with a Tukey’s test **d**, **f**, **g** or Dunnett’s test **e**. *p < 0.05, **p < 0.01, ***p < 0.001. Source data are provided as a Source data file.

Encouraged by the efficient tumor homing and ICD-induction capacity of Oxa(IV)@ZnPc@M, we next evaluated whether the antitumor immunity triggered by chemo-photodynamic therapy of Oxa(IV)@ZnPc@M could be harnessed to potentiate checkpoint blockade therapy (e.g. anti-PD-L1) to enhance antitumor and anti-metastasis efficacy. As 4T1 is a well-known aggressive tumor model with high metastasis rate, we first evaluated the antitumor effect in 4T1 tumor model. A bilateral 4T1 tumor-bearing mouse model was developed, where subcutaneous injection of 4T1 cells in the left flank was assigned as primary tumors for local NIR irradiation, and intra-tibia injection of Luc-4T1 cells into the right tibia was set as an artificial model of bone metastatic tumor. When the primary tumors reached ~100–150 mm^3^, mice were randomly assigned to eight groups (*n* = 5): (1) PBS (+), (2) free Oxa, (3) Oxa(IV)@ZnPc, (4) Oxa(IV)@ZnPc@M, (5) BMMs + anti-PD-L1, (6) Oxa(IV)@ZnPc (+), (7) Oxa(IV)@ZnPc@M (+), and (8) Oxa(IV)@ZnPc@M (+) + anti-PD-L1. The formulations were intravenously injected at an equal platinum dose of 1 mg/kg and ZnPc dose of 0.75 mg/kg (~1 × 10^6^ cells/mouse) every 2 days for a total of three injections. “(+)” represents irradiation with NIR laser (671 nm, 250 mW cm^−2^) for 10 min at 4 h and 24 h post-injection. After the second irradiation, the mice were intraperitoneally injected with anti-PD-L1 antibody (100 μg/mouse) (Fig. 6a). The growths of primary tumors and bone metastatic tumors were monitored by calipers measurement and bioluminescence imaging (BLI), respectively. Oxa and Oxa(IV)@ZnPc showed a slight inhibitory effect on primary and bone metastatic tumors compared with PBS (+) group (Fig. 6c–e). Oxa(IV)@ZnPc@M showed a slightly enhanced antitumor effect compared to Oxa(IV)@ZnPc, which might be attributed to the superior pharmacokinetics, intrinsic tumor accumulation, and the polarization toward the antitumor M1 phenotype. BMMs + anti-PD-L1 group exhibited moderate inhibitory effects on primary and bone metastatic tumors, since BMMs was non-toxic to 4T1 cells, this group could be used to represent the anti-PD-L1 effect alone. Oxa(IV)@ZnPc (+) and Oxa(IV)@ZnPc@M (+) treatments displayed significantly enhanced antitumor efficiency for primary tumors. Compared with the PBS (+) group, the tumor volumes were reduced by 75% and 84%, respectively, indicating remarkable chemo-photodynamic therapeutic effect. However, they failed to exert inhibitory effect on bone metastatic tumors, only similar to the BMMs + anti-PD-L1 group. Oxa(IV)@ZnPc@M (+) + anti-PD-L1 exerted the strongest antitumor effect among all groups, with primary tumor shrinkage rate up to 94% compared with PBS (+) group, and the least increase in luminescence intensity of bone metastatic tumors compared to all groups (Fig. 6c–e). Meanwhile, Oxa(IV)@ZnPc@M (+) + anti-PD-L1 acquired the maximum reduction in tumor volume and tumor weight (Fig. 6f and Supplementary Fig. 20a). Hematoxylin-eosin (H&E) staining and terminal deoxynucleotidyl transferase dUTP nick-end labeling (TUNEL) analysis further uncovered that Oxa(IV)@ZnPc@M (+) + anti-PD-L1 induced most remarkable apoptosis and necrosis of the primary and bone metastatic tumors (Fig. 6i and Supplementary Figs. 21 and 22a).

Bone metastases from breast cancer are often accompanied with osteolysis^47^. Thus the severity of bone osteolysis of the ex vivo tibias was evaluated by 3D micro-CT. Tibias in PBS (+), Oxa, Oxa(IV)@ZnPc, and Oxa(IV)@ZnPc@M were severely eroded and comminuted fractures were observed. Tibias in BMMs + anti-PD-L1, Oxa(IV)@ZnPc (+) and Oxa(IV)@ZnPc@M (+) groups exhibited a slight alleviation of bone destruction compared with PBS (+) group. Fortunately, the integrity of tibia was well preserved in the Oxa(IV)@ZnPc@M (+) + anti-PD-L1 group, with only minor damage compared to the normal group (Fig. 6h). Quantitative ROI analysis of bone parameters (e.g. BV/TV, Tb. N, and BS) further confirmed the result (Fig. 6g and Supplementary Fig. 20b, c). Meanwhile, H&E staining of mice tibias also displayed the least degree of bone osteolysis in Oxa(IV)@ZnPc@M (+) + anti-PD-L1 group, while the tibias of PBS (+) group developed severe osteolytic bone lesions, with a large amount of proliferative tumor cells filled in the damaged bones (Fig. 6j). Because osteolysis is induced by osteoclast’s bone resorption, tartrate-resistant acid phosphatase (TRAP) staining of osteoclasts can represent the severity degree of osteolysis in vivo. As expected, Oxa(IV)@ZnPc@M(+) + anti-PD-L1 group showed a greatly reduced TRAP positive cells (Supplementary Fig. 22b), which might be attributed to the combined suppression of tumor cells, thereby inhibiting osteoclasts activation simultaneously. All of these results indicated that ICD induced by chemo-photodynamic therapy of Oxa(IV)@ZnPc@M (+) combined with anti-PD-L1 blockade could effectively inhibit primary and bone metastatic breast cancer.

Unexpectedly, intra-tibia injection of 4T1 cells induced spontaneous lung metastases, as demonstrated by BLI (Fig. 6c). Obvious lung metastases were observed in PBS (+), Oxa, Oxa(IV)@ZnPc, and Oxa(IV)@ZnPc@M group on day 22 after the intra-tibia injection of Luc-4T1 cells. In contrast, lung metastases in mice treated with Oxa(IV)@ZnPc@M (+) + anti-PD-L1 were substantially suppressed. H&E staining displayed a large number of metastatic lesions in the lungs of PBS (+), Oxa, Oxa(IV)@ZnPc, and Oxa(IV)@ZnPc@M groups, and a moderate amount of tumor cells occupied in the lungs of BMMs + anti-PD-L1, Oxa(IV)@ZnPc (+), and Oxa(IV)@ZnPc@M (+) groups. However, the lung metastases were markedly limited in Oxa(IV)@ZnPc@M (+) + anti-PD-L1 group (Fig. 6k), and no obvious body weight loss was detected (Fig. 6b), suggesting the good biocompatibility of the cell-based formulation and the combined chemo/photo/immunotherapy. However, after day 12, all other treatment groups exhibited a slight weight loss, which might be caused by potential lung metastases. All these results revealed that Oxa(IV)@ZnPc@M combined with anti-PD-L1 accommodated a robust capacity of not only anti-bone metastasis but also against the related lung metastasis.

### Mechanism of systematic antitumor immune responses

**Fig. 7:**
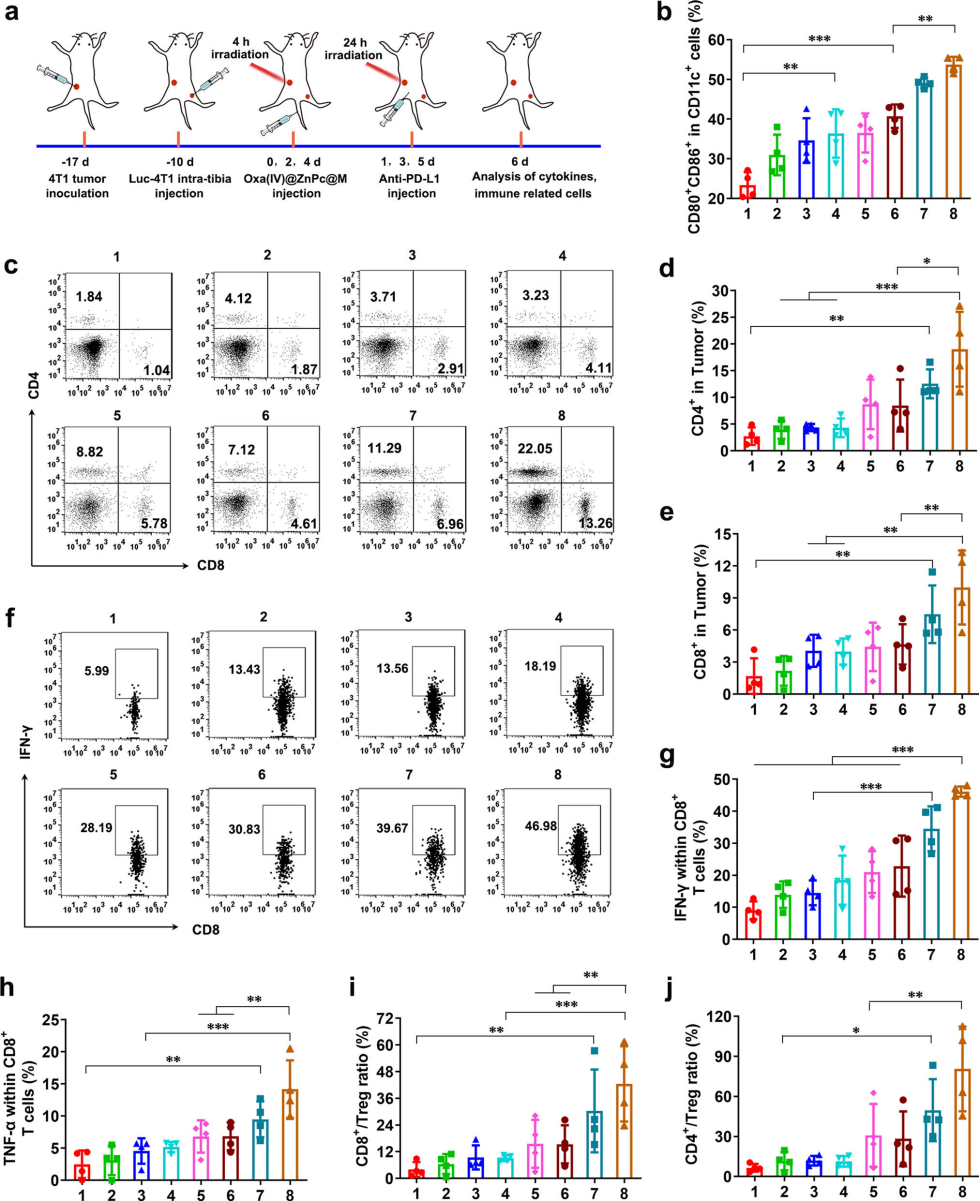
Systematic antitumor immune responses mechanism in 4T1-bearing mice. **a** Schematic illustration of experimental design. **b** Quantitation of the percent of mature DCs (CD11c^+^CD80^+^CD86^+^) (**p = 0.0048, ***p = 0.0001, **p = 0.0046) in tumor-draining lymph nodes on day 2 after various treatment (1: PBS (+); 2: free Oxa; 3: Oxa(IV)@ZnPc; 4: Oxa(IV)@ZnPc@M; 5: BMMs + anti-PD-L1; 6: Oxa(IV)@ZnPc (+); 7: Oxa(IV)@ZnPc@M (+); 8: Oxa(IV)@ZnPc@M (+) + anti-PD-L1, “(+)” represent with laser irradiation, applied for all the studies in vivo). **c** The representative flow cytometric plots of tumor-infiltrating CD4^+^ T cells and CD8^+^ T cells. Quantitation of the percent of tumor-infiltrating **d** CD4^+^T cells (**p = 0.0060, ***p = 0.0002, *p = 0.0102) and **e** CD8^+^T cell in tumors analyzed on day 6 after various treatment (**p = 0.0047, **p = 0.0038, **p = 0.0098). **f** The representative flow cytometric plots of IFN-γ within CD8^+^ T cells. Quantitation of the percent of **g** IFN-γ within CD8^+^ T cells (***p = 0.0005, ***p < 0.0001) and **h** TNF-α within CD8^+^ T cells in tumors analyzed on day 6 after various treatment (**p = 0.0047, ***p = 0.0001, **p = 0.0030). Quantitation of the percent of **i** intratumoral ratio of CD8^+^ T cells to Treg (**p = 0.0096, ***p = 0.0009, **p = 0.0078) and **j** CD4^+^ T cells to Treg (*p = 0.0340, **p = 0.0040). All data were presented as mean ± SD (*n* = 4 individual animals). Statistical significance was calculated via ordinary one-way ANOVA with a Tukey’s test **b** or Dunnett’s test **d**, **e** and **g**–**j**. *p < 0.05, **p < 0.01, ***p < 0.001. Source data are provided as a Source data file.

As 4T1 is a highly accepted poorly immunogenic tumor model^48^, we firstly used the 4T1-tumor-bearing mice to verify whether Oxa(IV)@ZnPc@M-augmented chemo-photodynamic therapy could create a immunogenic hot tumor microenvironment to boost antitumor immune response. The 4T1-tumor-bearing mice received the same treatment procedure as antitumor studies (Fig. 7a). Tumor tissues were isolated at day 6 for examination of chemotherapy/PDT-induced ICD. Immunofluorescence (IF) and immunohistochemistry (IHC) analysis of the tumor sections exhibited that Oxa(IV)@ZnPc@M (+) dramatically enhanced CRT exposure and HMGB-1 release in vivo, obviously higher than Oxa and Oxa(IV)@ZnPc@M (Supplementary Fig. 23a, b), verifying the magnification of ICD-induction efficacy by targeted chemo-photodynamic therapy. Next, the induction of dendritic cells (DCs) maturation in tumor-draining lymph node was detected by flow cytometry. The matured DCs were denoted as CD11c^+^CD80^+^CD86^+^ cells. According to the result, the Oxa(IV)@ZnPc@M (+) + anti-PD-L1 treatment showed the best effect on promoting DCs maturation, with the expression of CD80 and CD86 dramatically up-regulated compared with other treatment groups (Fig. 7b and Supplementary Fig. 24), indicating the best effect on maturating and activating of DCs, which showed great potential to stimulate the subsequent tumor-specific immune response.

It is well-known that CD8^+^ T cells can release perforin, granzymes, and granulysin to kill tumor cells, and CD4^+^ T cells play crucial roles in the regulation of adaptive immunity^18,49^. Therefore, we next assessed the CD8^+^ and CD4^+^ T cells in bone metastatic tumors at day 6. Mice treated with Oxa(IV)@ZnPc@M (+) + anti-PD-L1 exhibited the highest effect in promoting intratumoral infiltration of CD8^+^ and CD4^+^ T cells, which were 5.9-fold and 6.9-fold of the PBS (+) group, respectively. Mice treated with BMMs + anti-PD-L1 and Oxa(IV)@ZnPc (+) led to moderate increase of CD8^+^ and CD4^+^ T cells, by contrast, mice treated with Oxa(IV)@ZnPc@M (+) showed a lightly increased effect (Fig. 7c–e and Supplementary Fig. 25). IF staining of CD8^+^ T cells and IHC staining of CD4^+^ T cells further verified the result (Supplementary Fig. 23c). Except a higher population of infiltrated CD8^+^ T cells, interferon-γ (IFN-γ)-positive CD3^+^CD8^+^IFN-γ^+^ T cells and TNF-α-positive CD3^+^CD8^+^ TNF-α^+^ T cells were also important to the therapeutic effect. Notably, the levels of CD3^+^CD8^+^IFN-γ^+^ T cells and CD3^+^CD8^+^TNF-α^+^ T cells in tumor were significantly elevated by Oxa(IV)@ZnPc@M (+) + anti-PD-L1 treatment (Fig. 7f, g and Supplementary Fig. 26). In addition, the population of Treg (CD3^+^CD4^+^Foxp3^+^) of Oxa(IV)@ZnPc@M (+) + anti-PD-L1 group decreased compared with PBS (+) group (Supplementary Fig. 27a–c), and the CD8^+^T/Treg ratio and CD4^+^T/Treg ratio were remarkably increased compared with other groups (Fig. 7i, j). All of these results revealed that Oxa(IV)@ZnPc@M (+) treatment or combined with anti-PD-L1 could significantly promote tumor-infiltration of antitumor lymphocyte. In addition, proinflammartory cytokines (IFN-γ, TNF- α) in mouse serum were measured by ELISA kit. Oxa(IV)@ZnPc (+) and Oxa(IV)@ZnPc@M (+) groups had higher serum concentration of IFN-γ and TNF-α than the PBS (+) group and the non-laser group, indicating that PDT could induce acute inflammatory response. Oxa(IV)@ZnPc@M (+) + anti-PD-L1 significantly increased the secretion of IFN-γ and TNF-α, which were 8.0-, 6.6-fold higher than those of the PBS (+) group, respectively (Supplementary Fig. 28), indicating the successful elicitation of host defense and cellular immunity by the macrophage-mediated chemo/photo/immunotherapy system.

Next, to further verify the advantages of using Oxa(IV)@ZnPc@M, whether Oxa(IV)@ZnPc@M could re-educate tumor-associated macrophages (TAM) from tumor-promoting M2 phenotype toward tumor-killing M1 phenotype was evaluated, where M1 macrophages were denoted as CD86^+^CD206^−^F4/80^+^ cells, and M2 macrophages were denoted as CD86^−^CD206^+^F4/80^+^ cells. Free Oxa and Oxa(IV)@ZnPc exhibited increased percentage of M1 macrophages and decreased percentage of M2 macrophages compared with PBS group, which might be attributed to the effect of Oxa. In comparison with free Oxa and Oxa(IV)@ZnPc, the percentage of M1 macrophages in Oxa(IV)@ZnPc@M group was markedly increased, and the percentage of M2 macrophages was significantly decreased, accompanied by remarkably elevated M1/M2 ratio, which was 1.58-fold of Oxa(IV)@ZnPc and 2.15-fold of free Oxa (Supplementary Fig. 29). The result might be ascribed to the combination effect of Oxa and the M1 phenotype of Oxa(IV)@ZnPc@M that can produce proinflammatory cytokines to re-educate TAM toward tumor-killing M1 phenotypes, enhancing the antitumor effect.

### Immune memory effect

**Fig. 8:**
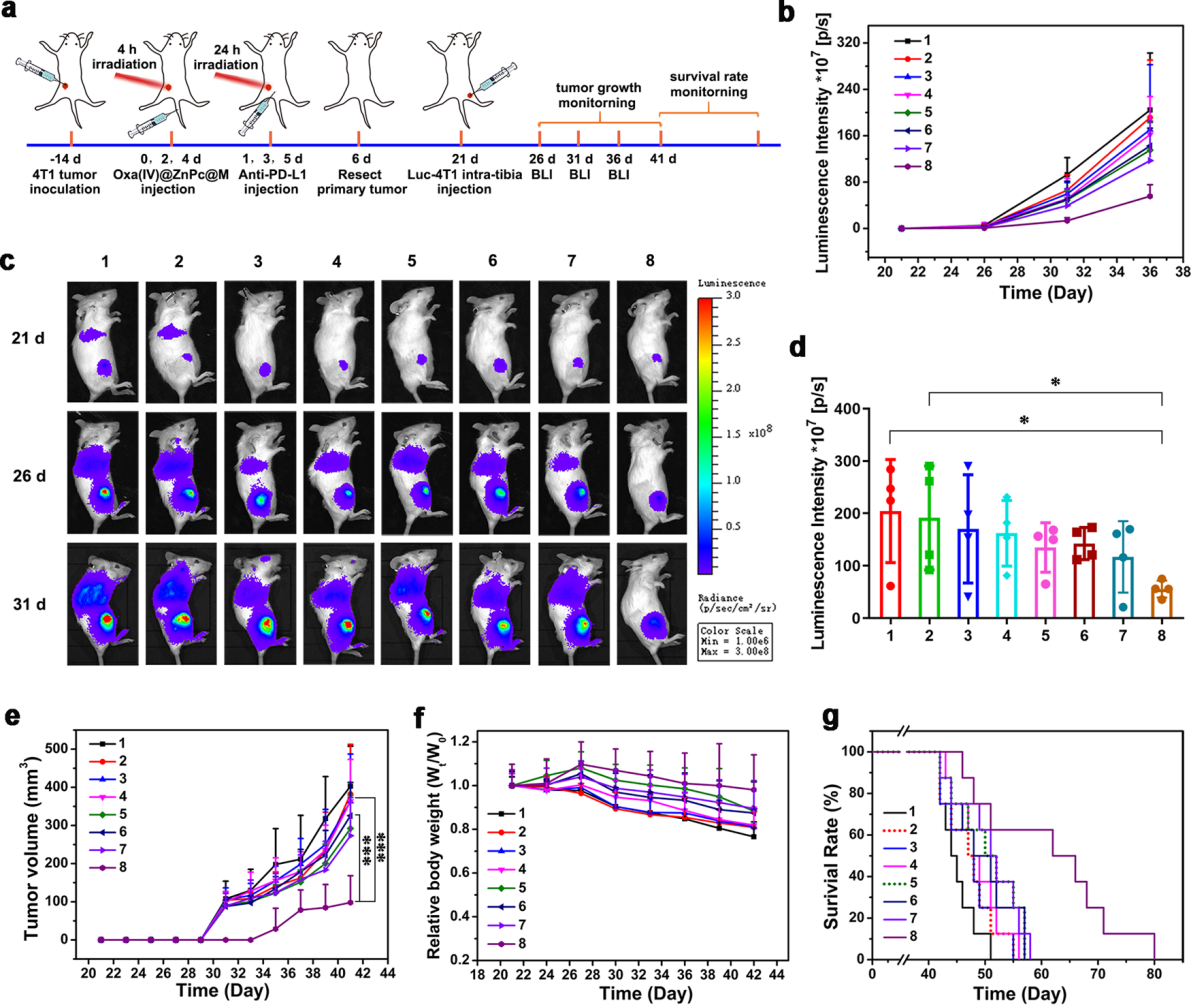
Long-term immune memory effects in 4T1-bearing mice. **a** Schematic illustration of experimental. **b** Tumor growth curves of rechallenged tumors monitored by BLI after various treatments (1: PBS (+); 2: free Oxa; 3: Oxa(IV)@ZnPc; 4: Oxa(IV)@ZnPc@M; 5: BMMs + anti-PD-L1; 6: Oxa(IV)@ZnPc (+); 7: Oxa(IV)@ZnPc@M (+); 8: Oxa(IV)@ZnPc@M (+) + anti-PD-L1, “(+)” represent with laser irradiation, applied for all the studies in vivo) (*n* = 4 individual animals). **c** Representative in vivo bioluminescence images tracking the growth of intra-tibia injected Luc-4T1 cells (*n* = 4 individual animals). **d** Quantitative luminescence intensity of Luc-4T1 bone metastatic tumors at the last BLI (*p = 0.0245, *p = 0.0356) (*n* = 4 individual animals). **e** Tumor growth curves of rechallenged tumors monitored by caliper measurement (*n* = 8 individual animals, the volume of mice without tumors is recorded as zero, ***p = 0.0003, ***p < 0.0001). **f** Relative body weight changes after various treatment (*n* = 8 individual animals). **g** Overall survival curves of mice after various treatments. All values were presented as means ± SD. Statistical significance was calculated via ordinary one-way ANOVA with a Dunnett’s test. *p < 0.05, **p < 0.01, ***p < 0.001. Source data are provided as a Source data file.

Long-term immune memory effect plays a crucial role in protecting tumor recurrence and metastasis. To investigate the immune memory potency of Oxa(IV)@ZnPc@M combined with PD-L1 blockade, Balb/c mice bearing 4T1 primary tumors were received the same treatment procedure as the antitumor study. Residual tumor masses of all treatment groups were surgically resected, and the mice were rechallenged with Luc-4T1 cells via intra-tibia injection. The growths of bone metastatic tumors were examined by BLI and caliper measurement, and the metastasis of tumor cells into the lung was observed by BLI (Fig. 8a). Except for Oxa(IV)@ZnPc@M (+) + anti-PD-L1 group, the rapid growth of the re-inoculated bone metastatic tumors were observed by BLI in the other groups (Fig. 8b–d), and visible bone metastatic tumors that could be measured by caliper were observed at the 10th day post-inoculation. None of mice in the Oxa(IV)@ZnPc@M (+) + anti-PD-L1 group exhibited visible bone metastatic tumors on day 10 post-inoculation, only 25% of the mice showed visible bone metastatic tumors on day 14 post-inoculation, indicting the growth of the re-inoculated tumors was inhibited (Fig. 8e). Of note, only the Oxa(IV)@ZnPc@M (+) + anti-PD-L1 group showed inhibited lung metastasis and negligible weight loss, all the other groups were developed obvious lung metastasis and detectable weight loss (Fig. 8c, f). In addition, mice in the PBS (+) group showed a 30-day life span after inoculation, and mice in the other six groups died within 37 days after inoculation. In remarked contrast, 50% of mice in the Oxa(IV)@ZnPc@M (+) + anti-PD-L1 group survived over 44 days after inoculation (Fig. 8g), demonstrating the protective long-term immune memory effect of the targeted chemo/photo/immunotherapy system.

**Fig. 9:**
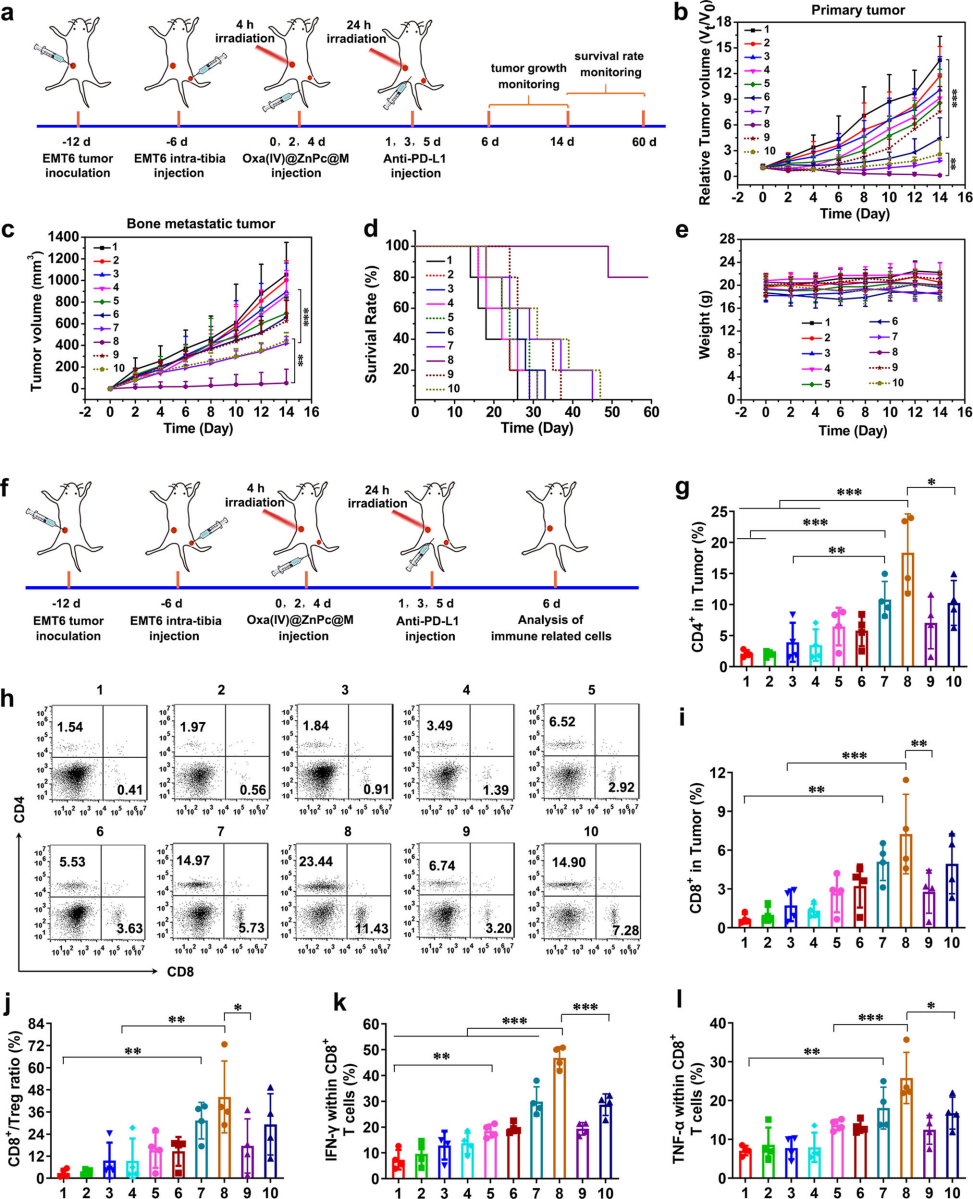
Therapeutic efficacy and systematic antitumor immune responses mechanism in EMT6-bearing mice. **a** Schematic illustration of experimental design. Tumor growth curves of **b** primary tumors (***p = 0.0001, **p = 0.0041) and **c** bone metastatic tumors (***p = 0.0006, **p = 0.0062) after various treatments (1: PBS (+); 2: free Oxa; 3: Oxa(IV)@ZnPc; 4: Oxa(IV)@ZnPc@M; 5: BMMs + anti-PD-L1; 6: Oxa(IV)@ZnPc (+); 7: Oxa(IV)@ZnPc@M (+); 8: Oxa(IV)@ZnPc@M (+) + anti-PD-L1; 9: Oxa + anti-PD-L1; and 10: Oxa(IV)@ZnPc (+) + anti-PD-L1, “(+)” represent laser irradiation, applied for all the antitumor studies in vivo) (*n* = 6 individual animals). **d** Overall survival curves. **e** Body weight after various treatments (*n* = 6 individual animals). **f** Schematic illustration of experimental design of antitumor immune evaluation. **g** Quantitation of the percent of tumor-infiltrating CD4^+^T cells (***p = 0.0008, **p = 0.0095, ***p < 0.0001, *p = 0.0467). **h** The representative flow cytometric plots of tumor-infiltrating CD4^+^ T cells and CD8^+^ T cells (*n* = 4 individual animals). Quantitation of the percent of **i** tumor-infiltrating CD8^+^T cell (**p = 0.0049, ***p = 0.0004, **p = 0.0044), **j** intratumoral ratio of CD8^+^ T cells to Treg (**p = 0.0014, **p = 0.0018, *p = 0.0219), **k** IFN-γ within CD8^+^ T cells (**p = 0.0043, ***p < 0.0001, ***p < 0.0001) and **l** TNF-α within CD8^+^ T cells (**p = 0.0031, ***p = 0^.^0010, *p = 0.0177) (*n* = 4 individual animals). All data were presented as mean ± SD. Statistical significance was calculated via the unpaired two-tailed t test b, ordinary one-way ANOVA with a Tukey’s test **g** or Dunnett’s test **c**, **i**–**l**. *p < 0.05, **p < 0.01, ***p < 0.001. Source data are provided as a Source data file.

Antitumor and immune response efficacy were confirmed in EMT6 bone metastases. EMT6 is a representative preclinical model of triple negative breast cancer (TNBC), similar to 4T1, it was commonly used for immunological evaluation^50,51^. Thus, the antitumor effect and the mechanism of systematic antitumor immune responses were further verified on EMT6 model. Compared with PBS (+) group, Oxa and Oxa(IV)@ZnPc showed a slight inhibitory effect on primary and bone metastatic tumors, and BMMs + anti-PD-L1 group exhibited moderate inhibitory effect. Oxa + anti-PD-L1 showed remarkably enhanced effect compared with Oxa, but comparable or a little stronger inhibitory effect than anti-PD-L1, this might be caused by the relative low dosage of Oxa injected. After laser irradiation, the antitumor efficiency of Oxa(IV)@ZnPc (+) and Oxa(IV)@ZnPc@M (+) group on primary tumors were significantly enhanced, with tumor volume cut down by 72.6% and 89.5%, respectively (Fig. 9b, c), suggesting remarkable chemo-photodynamic therapeutic effect. Meanwhile, Oxa(IV)@ZnPc@M (+) showed stronger antitumor effect on bone metastatic tumors than Oxa(IV)@ZnPc (+), possibly due to the superior pharmacokinetics and intrinsic tumor accumulation of the cell-based pharmaceutics. However, Oxa(IV)@ZnPc (+) combined with anti-PD-L1 only exhibited comparable effect to Oxa(IV)@ZnPc@M (+) along. Notably, when Oxa(IV)@ZnPc@M (+) combined with anti-PD-L1, strongest antitumor effect among all groups on both primary and bone metastatic tumors were observed, with tumor shrinkage rate up to 99.4% and 95.1% respectively compared with PBS (+) group (Fig. 9b, c). The antitumor effect on EMT6 model was consistent with that of 4T1 model. In addition, mice in the PBS (+) group showed a 28-day life span after inoculation, and mice in the other groups without laser irradiation died within 38 days after inoculation. Oxa(IV)@ZnPc@M (+) showed a significantly prolonged life span, with ~50 days after inoculation, which was comparable to Oxa(IV)@ZnPc (+). In remarked contrast, 80% of mice in the Oxa(IV)@ZnPc@M (+) + anti-PD-L1 group survived over 60 days after inoculation (Fig. 9d), and tumors of these mice were cured, demonstrating the prominent effect of the cell-based targeted chemo/photo/immunotherapy system. Besides, no obvious body weight loss was observed after various treatment (Fig. 9e), further confirming the good biocompatibility of the cell-based formulation and the cell formulation mediated chemo/photo/immunotherapy.

Consistent with the results in 4T1 model, Oxa(IV)@ZnPc@M (+) + anti-PD-L1 treated mice exhibited the highest frequency of CD8^+^ and CD4^+^ T cells infiltration, which were 10.4-fold and 8.7-fold higher than the PBS (+) group, respectively. Mice treated with Oxa(IV)@ZnPc@M (+) showed a slightly increased CD8^+^ and CD4^+^ T cells compared with BMMs + anti-PD-L1 and Oxa(IV)@ZnPc (+), and it showed comparable effect to Oxa(IV)@ZnPc (+) + anti-PD-L1 group (Fig. 9g–i and Supplementary Fig. 30). The proportion of Treg (CD3^+^CD4^+^Foxp3^+^) of Oxa(IV)@ZnPc@M (+) + anti-PD-L1 group decreased compared with PBS (+) group, and the CD8^+^ T/Treg ratio and CD4^+^ T/Treg ratio were significantly increased compared with other groups (Fig. 9j and Supplementary Fig. 31). Notably, except that a higher population of infiltrated CD8^+^ T cells, the levels of CD3^+^CD8^+^IFN-γ^+^ T cells and CD3^+^CD8^+^TNF-α^+^ T cells in tumor were significantly elevated by Oxa(IV)@ZnPc@M (+) + anti-PD-L1 treatment (Fig. 9k, l and Supplementary Fig. 32). All of these results revealed that Oxa(IV)@ZnPc@M (+) treatment or combined with anti-PD-L1 could significantly promote tumor-infiltration of antitumor lymphocyte. At the meanwhile, consistent with 4T1, Oxa(IV)@ZnPc@M showed the same ability to re-educate TAM from tumor-promoting M2 phenotype toward tumor-killing M1 phenotype in EMT6 model. As is shown in Supplementary Fig. 33, in comparison with free Oxa and Oxa(IV)@ZnPc, the percentage of M1 macrophages in Oxa(IV)@ZnPc@M group was markedly increased, and the percentage of M2 macrophages was significantly decreased, accompanied by remarkably elevated M1/M2 ratio, which was 1.66-fold of Oxa(IV)@ZnPc and 2.45-fold of free Oxa.

### Toxicity evaluation

**Fig. 10:**
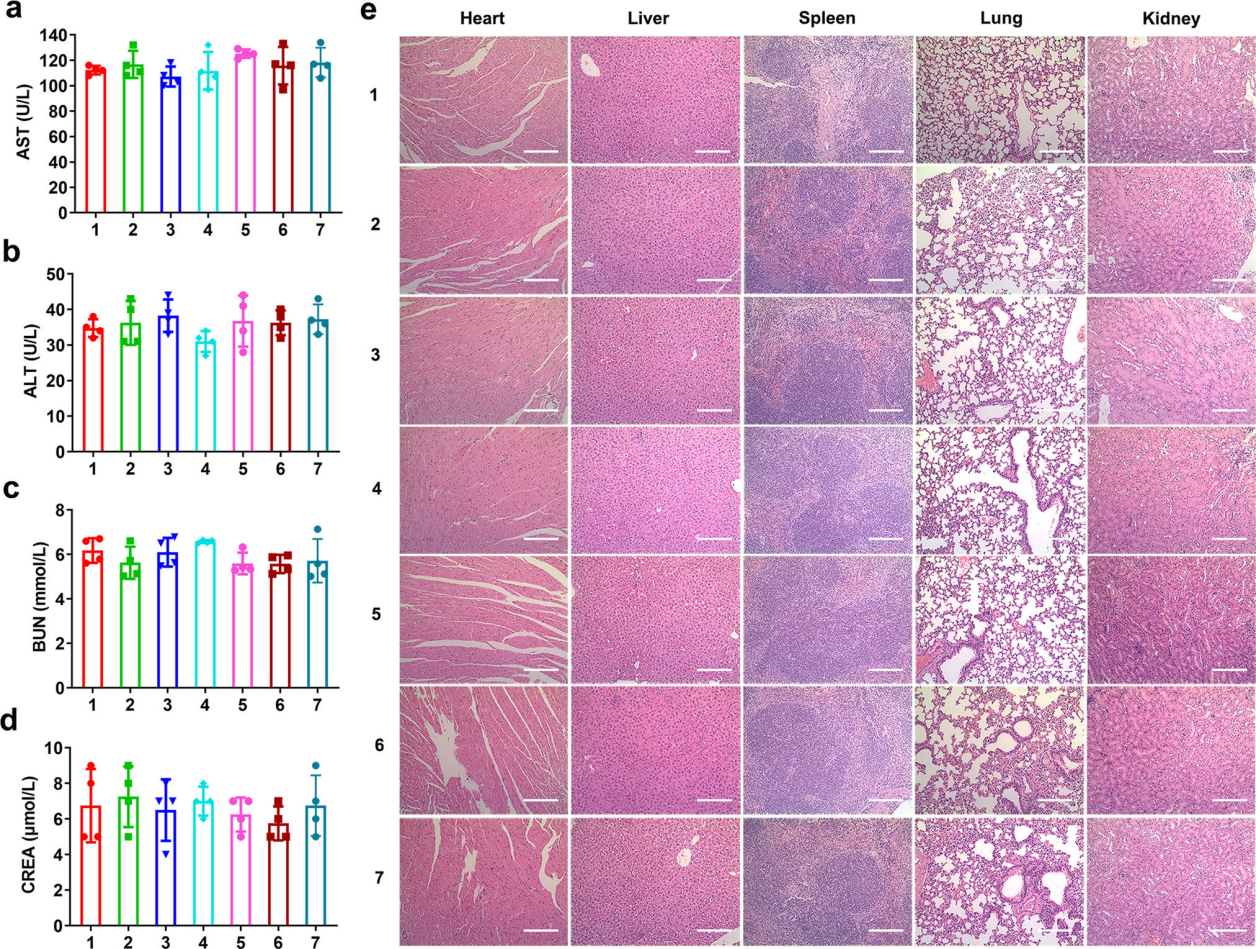
Toxicity studies. Plasma levels of **a** AST, **b** ALT, **c** BUN, and **d** CREA after multi-dose injections of different drug formulations (1: PBS; 2: free Oxa; 3: Oxa(IV)@ZnPc; 4: Oxa(IV)@ZnPc@M; 5: BMMs; 6: Oxa(IV)@ZnPc@M + anti-PD-L1; and 7: BMMs + anti-PD-L1) (*n* = 4 individual animals). No significant difference of the treatment group compared with PBS group. **e** H&E staining of major organs after multi-dose injections, this experiment was performed two times with similar results. Scale bars = 200 µm. All data were presented as mean ± SD. Source data are provided as a Source data file.

The systematic toxicity of Oxa(IV)@ZnPc@M was evaluated by detecting the blood routine and biochemical indexes in healthy Balb/c mice. Biochemical indexes, including alanine transaminase (ALT) and aspartate aminotransferase (AST), and blood urea nitrogen (BUN) and creatinine (CREA) were detected. Compared with PBS group, the CREA level of Oxa(IV)@ZnPc@M + anti-PD-L1 group (single dose) decreased, but it was within the normal range. In addition, no significant differences were detected in biochemical indexes (ALT, AST, BUN, and CREA) of all treatment groups compared with PBS group with single-dose and multi-dose injection (Fig. 10a–d and Supplementary Fig. 34a–d), indicating negligible hepatotoxicity and nephrotoxicity. Meanwhile, the complete blood count analysis found that all indices of all treatment groups were within the normal range, similar to the PBS group (Supplementary Tables 2–3). No pathological changes of major organs (i.e., heart, liver, spleen, lung, and kidney) by H&E staining were observed in all treatment groups compared with the PBS group (Fig. 10e and Supplementary Fig. 34e). Overall, the hematology and histopathology analysis verified that Oxa(IV)@ZnPc@M and BMMs have superior biocompatibility and could serve as a safe carriers.

## Discussion

Bone metastases are highly frequency and associated with poor prognosis in patients with breast cancer, and none effective therapeutic options currently available. Recently, bone-targeted nonocarriers have been exploited for chemotherapy, photothermal, or photodynamics therapy of breast cancer bone metastasis^52–54^. However, bone-targeted nanocarriers may face numerous biological obstacles after intravenous injection, and it is difficult to synchronously treat primary tumors. Thus, it is critical to develop versatile drug delivery vectors and effective treatment modalities that can eliminate primary and bone metastatic tumors simultaneously. In this work, we proposed macrophage-mediated drug vector for the tumor-selective delivery and NIR-activated release of Oxa prodrug and photosensitizer to achieve chemo-photodynamic augmented immunotherapy of primary and bone metastatic breast cancer. Our engineered macrophages have been demonstrated to effectively traffic to primary and bone metastatic tumors, overcoming the limitations of current bone-targeted nanocarriers (Fig. 5b–f).

To achieve high drug loading with unimpaired cellular functions, firstly, an Oxa prodrug-based core-shell nanocarrier Oxa(IV)@ZnPc was designed. Thanks to the prodrug-based nanotechnology and the inactivated photosensitizer, BMMs bear a high drug loading at ~20 pg Oxa and 15 pg ZnPc per cell with high cell viability (Fig. 2f, g). Because the acidifcation of phagosomes and the fusion with lysosomes are a time-consuming processes^55^, thus drugs can be temporarily stabilized in the core of Oxa(IV)@ZnPc to avoid poisoning macrophages, buying time for Oxa(IV)@ZnPc@M to navigate to tumors. However, controlled or stimuli-responsive drug release from the cell-based drug delivery systems at the target site to avoid non-specific drug release is still a challenge. Herein, we demonstrated that benefiting from the photo-activated PDT, Oxa(IV)@ZnPc@M served as drug reservoir, therapeutics inside Oxa(IV)@ZnPc@M undergo NIR triggered release, overcoming the uncontrolled release (the major obstacle) faced by cell-based vehicles (Fig. 4b, c, g). But what is disappointing is that the price of triggering the release of the drug is the suicide of the carrier macrophages, and the specific mechanism for NIR-triggered drug release still needs to be further explored.

It is well documented that macrophages are highly plastic cells that can polarize to antitumor M1 phenotype and tumor-promoting M2 phenotype^56^. M1 macrophages, also known as classically activated macrophage, can produce proinflammatory cytokines through biosynthesis pathways^56^, such as TNF-α, nitric oxide and ROS. Li et al. reported that loading superparamagnetic iron oxide nanoparticles (HIONs) into macrophages reprogramed them to M1 phenotype, which can generate bioactive components to suppress tumor growth and prime in situ TAM to antitumor M1 phenotype^57^. It is interesting that our engineered macrophages exhibited M1 phenotype, and Oxa loaded in macrophages may be the cause of phenotypic polarization (Fig. 4j–l and Supplementary Fig. 19). In addition, Oxa(IV)@ZnPc@M can retain the M1 phenotype in tumor (Fig. 4m, n), though Oxa(IV)@ZnPc@M in primary tumor can be killed after light irradiation, while Oxa(IV)@ZnPc@M that home to the bone metastatic tumors without light irradiation can still has the ability to re-educate TAM to M1 phenotype (Supplementary Fig. 29), which can inhibit the tumor directly and reprogram the immunosuppressive tumor microenvironment to some extent.

So far, extensive literatures have reported that ICD provides dying cancer cells can served as “in situ vaccines” to elicit immune responses and create an immunogenic hot tumor microenvironment, thus potentiating current immunotherapy outcomes and expanding the benificiaries for the “cold” breast cancer^19,58^. Oxa and photodynamic therapy are currently widely accepted ICD inducers, while numerous published literature use nanocarriers as delivery vehicles^58–62^. Our macrophage-mediated “ICD inducers” vector exhibited higher delivery efficiency and ICD inducibility than nanocarriers, and exhibited the strongest function in activating of DCs, promoting intratumoral infiltration of CD8^+^ and CD4^+^ T cells and inhibiting of primary and bone metastatic tumors (Figs. 5–8). Therefore, we provide a cell-based treatment platform whether for the efficient delivery of ICD inducers or for the effective treatment of bone metastasis that has received very little attention.

It is reported that collateral damage to normal cells by cytotoxic agents results in the release of self-antigens, leading to immune tolerance or suppression^58^. In order to reduce collateral damage, we have made the following efforts. Firstly, body’s own macrophages with good biocompatibility were selected as drug delivery vehicles. Secondly, exploit engineered macrophages to accurately deliver prodrug-based cytotoxic agent to the tumor site to reduce off-target effect. Thirdly, cytotoxic chemotherapy was combined with PDT, an effective and minimally invasive therapeutic modality, which enabled the cytotoxic agent to be released in a NIR controlled mode, further reducing the toxicity to normal tissues. According to the toxicity data (Fig. 10, Supplementary Fig. 34, and Supplementary Tables 2–3), Oxa(IV)@ZnPc@M showed superior hematology and histopathology biocompatibility. However, since Oxa(IV)@ZnPc@M exhibited M1 phenotype in vivo, the potential side effects of the M1 macrophages that secret pro-inflammatory factors in normal tissues are rarely noticed and deserve more attention. In addition, a more detailed assessment of the systemic long-term toxicity of Oxa(IV)@ZnPc@M, especially the toxicity to immune organs, will be further investigated in our future work. Last but not least, due to the expensive scale-up and long-term stability of the cellular-based biologics, great efforts still need to be paid for the clinical translation of Oxa(IV)@ZnPc@M.

In summary, we developed a NIR-responsive macrophage-mediated drug vector and “in situ vaccines” generator, which selectively kills cancer cells by chemo-photodynamic therapy, and boosts cancer immunity via ICD induction simultaneously, achieving enhanced chemo/photo/immunotherapy of primary tumors and bone metastases combined with anti-PD-L1. This work highlights that controlled and accurate delivery of cytotoxic agents with “ICD induction” functions by macrophage-mediated delivery combined with anti-PD-L1 would be a promising therapeutic strategy for efficient synchronous therapy of primary and bone metastatic breast cancer.

## Methods

### Materials

Oxaliplatin (Oxa, >98%) was purchased from Energy Chemical (Shanghai, China). Sodium pyrophosphate (Na_4_P_2_O_7_•10H_2_O, SPP, > 99.0%) was obtainted from Tianjing Fuchen Chemical Reagents Factory. Zinc phthalocyanine (ZnPc, >97%), fluorescein diacetate (FDA), 2′,7′-Dichlorofluorescein diacetate (DCFH-DA) and 3-(4,5-dimethyl-2-thiazolyl)-2,5-diphenyl-2-H-tetrazolium bromide (MTT) were obtained from Sigma-Aldrich (St. Louis, USA). Succinic anhydride (SA, >99%) was purchased from Aladdin Industrial Corporation (Shanghai, China). 1,1-Dioctadecyl-3,3,3,3-tetramethylindodicarbocyanine (DiD) were purchased from AAT Bioquest Inc. (Sunnyvale, CA, USA). Cholesterol, 1,2-dioleoyl-sn-glycero-3-phosphate (DOPA) and 1, 2 -dioleoylsn-glycero-3-phosphocholine (DOPC) were obtained from Avanti Polar Lipids, Inc. (Alabaster, USA). Calcein was purchased from Solarbio life sciences (Beijing, China). Annexin V/PI Apoptosis Detection Kit, and ELISA Kit for the detection of IFN-γ, TNF-α, IL-6, and IL-10 were obtained from MultiSciences (Hangzhou, China). Mouse HMGB-1 (High mobility group protein B1) ELISA Kit was purchased from elabscience (Wuhan, China). ATP assay kit, Propidium Iodide (PI), and CCK8 kit were purchased from Beyotime (Shanghai, China). Anti-CD3e-FITC[145-2C11](Cat# 11-0031-81, diluted 1:100 with 4% FBS), anti-CD8α-PE[53-6.7](Cat# 12-0081-82, diluted 1:80 with 4% FBS), anti-CD4-APC[RM4-5](Cat# 17-0042-82, diluted 1:160 with 4% FBS), anti-Foxp3-PE[NRRF-30](Cat# 12-4771-80, diluted 1:80 with 4% FBS), anti-IFN-γ-APC[XMG1.2](Cat# 17-7311-81, diluted 1:160 with 4% FBS), anti-TNF-α-PC7[MP6-XT22](Cat# 25-7321-80, diluted 1:160 with 4% FBS), anti-F4/80-FITC[BM8](Cat# 11-4801-81, diluted 1:50 with 4% FBS), anti-CD11b-APC[M1/70](Cat# 17-0112-81, diluted 1:160 with 4% FBS), anti-CD86(B7-2)-PC7[GL1](Cat# 25-0862-80, diluted 1:80 with 4% FBS), anti-CD206-PE[MR6F3](Cat# 12-2061-82, diluted 1:160 with 4% FBS), anti-CD11c-FITC[N418](Cat# 11-0114-81, diluted 1:200 with 4% FBS), anti-CD80(B7-1)-PE[16-10A1](Cat# 12-0801-81, diluted 3:1000 with 4% FBS), anti-CD16/32[93](Cat# 14-0161-81, diluted 1:100 with 4% FBS) antibodies, and Fixable Viability Dye eFluor^TM^ 450 (Cat# 65-0863, diluted 1:1000 with PBS) and eFluor^TM^ 780 (Cat# 65-0865, diluted 1:1000 with PBS) were purchased from eBiosciences (Hatfield, UK). Anti-rabbit calreticulin polyclonal antibody (Cat# 27298-1-AP, diluted 1:250 with 5%BSA), anti-rabbit HMGB1 antibody (Cat# 10829-1-AP, diluted 1:100 with 5%BSA), and anti-rabbit Alexa Fluor® 488 secondary antibody (Cat# srbAF488-1, diluted 1:100) were purchased from Proteintech Group (USA). Ultrapure water (18.2 Ω) was obtained from an Ultra Bio Mk2 ultrapure system (Elga, UK). d-Luciferin potassium salt was purchased from Goldbio (USA). InVivoPlus anti-mouse PD-L1 (B7-H1) antibody (α-PD-L1, Clone: 10 F.9G2, Cat# BP0101) was purchased from BioXcell (USA). All other chemicals were used as analytical grade without further purification.

### Cell lines

4T1 cells and L929 cells were purchased from the Laboratory Animal Center of Sun Yat-sen University (Guangzhou, China), and cultured in Dulbecco’s Modified Eagle Medium (DMEM, Gibco) supplemented with 10% fetal bovine serum (FBS, Gibico) and 1% antibiotics. Luc-4T1 cells (FH1114) stably expressing high levels of luciferase and EMT6 cells were purchased from FuHeng Cell Cente (Shanghai, China) and maintained in RPMI-1640 medium containing 10% FBS and 1% antibiotics.

### Animals

Healthy Balb/c female mice (5–6 weeks), C57BL/6 female mice (6–8 weeks), and Sprague-Dawley male rats (SD, 180–200 g) were obtained from the Laboratory Animal Center of Sun Yat-sen University (Guangzhou, China). All animal experiments were conducted under protocols approved by the Institutional Animal Care and Use Committee of Sun Yat-sen University. The animals were hosted in an SPF barrier environment with temperature at 20–26 °C and humidity at 40%–70%, under the 12 h/12 h dark/light cycle.

### Isolation and identification of bone marrow-derived macrophages

To prepare BMMs, C57BL/6 mice (8–12 weeks) were killed and the femur and tibia were isolated. Bone marrow cells were flushed out with DMEM medium using a 26G needle, and then the cells were filtered through 70 µm cell strainers (Corning) to remove debris. After centrifugation (150 g, 6 min), the cells were dissociated into single-cell suspensions and seeded on a 10 cm dish with 10 mL of DMEM containing 15% FBS, 1% antibiotics and 30% L929 cell-conditioned media (L929-CM) or 20 ng/mL of commercial Macrophage-Colony Stimulating Factor (M-CSF) (Invitrogen). L929-CM, a source of M-CSF, was prepared by growing L929 to confluence and further maintained for one week, and then the supernatants were collected by centrifugation and filtration. After incubating for 30 h and on the fourth day, fresh L929-CM containing medium was replaced, and adherent cells were harvested on the 6th or 7th days. Differentiation of BMMs was detected by flow cytometry (Guava EasyCyte 6-2L, Merck Millpore) by double staining with FITC-F4/80 and PE-CD11b antibodies. The result proved that 97% of cells were CD11b^+^F4/80^+^.

### Synthesis and characterization of Oxa(IV)-COOH

Oxa (200 mg, 0.50 mmol) was dissolved in 2 mL of 30% H_2_O_2_. The reaction was stirred in a water bath at 50 °C under dark for 2 h, then stirred overnight at room temperature, and then cooled at 4 °C for several hours. The resulting product (Oxa(IV)-OH) was obtained by pumping filtration, washed with ice deionized water, ethanol, and diethyl ether, and then dried in a vacuum (170 mg, yield 85%). ^1^H NMR (400 MHz, DMSO-d6): 7.68 ppm (br, 2H), 6.89 ppm (br, 2H), and 0.9–2.10 ppm (m, 10H). ESI-MS ([M-H]^−^) calculated for C_8_H_16_N_2_O_6_Pt (431.2), found 430.1.

Oxa(IV)-COOH was synthesized by reacting SA (93 mg, 0.93 mmol) with Oxa(IV)-OH (100 mg, 0.23 mmol) in 2 mL of DMF at 70 °C for 24 h in the dark. The solution was completely removed under reduced pressure, and the resultant was redissolved in acetone, then filtrated and repeatedly washed with acetone to obtain raw product. Oxa(IV)-COOH was purified by dissolving in methanol, and then precipitated by adding ethyl ether, followed by filtration, washing with ethyl ether, and drying in a vacuum (yield 75%). ^1^H NMR (400 MHz, DMSO-d6), 8.00–8.50 ppm (br, 4H), 0.95–2.25 ppm (m, 10H), 2.25–2.50 ppm (m, 8H), and 12.06 (s, 2H). ESI-MS ([M-H]^−^) calculated for C_16_H_24_N_2_O_12_ (631.2), found 630.0.

### Preparation of Oxa(IV)@ZnPc nanoparticles

DOPA-coated nanoparticles, Oxa(IV)@DOPA, were synthesized according to our previously reported method^63^. Briefly, 200 µL of Oxa(IV)-COOH sodium salt solution (8.83 mg/mL) and 200 µL of Zn(NO_3_)_2_ aqueous solution (25 mg/mL) were separately added to 5 mL of microemulsion (0.3 M TritonX-100 and 1.5 M hexanol in cyclohexane) under vigorous stirring at room temperature for 10 min. Thirty microliters of DOPA (138 mM in CHCl_3_) was added to the Zn^2+^ phase, and the mixture was stirred. The Oxa(IV)-COOH phase was then slowly added to Zn^2+^ phase with stirring and the reaction was continued for additional 45 min. Thereafter, 10 mL of ethanol was added, and the precipitated particles were harvested by centrifugation (17,745×*g*, 15 min), and then washed twice with ethanol and THF. The resulting nanoparticles were re-dispersed in CHCl_3_ and stored at 4 °C. SPP@DOPA and Cal@DOPA were synthesized according to the above method but with the addition of sodium pyrophosphate (SPP) or calcain (a fluorescent dye) aqueous solution instead of Oxa(IV)-COOH.

Oxa(IV)@ZnPc was prepared by adding a CHCl_3_ solution of DOPC/cholesterol (2:1 molar ratio) and a DMSO solution of ZnPc to Oxa(IV)@DOPA, mixed, and then CHCl_3_ was completely evaporated at 50 °C. The resulting lipid film was rehydrated in PBS under ultrasound, and centrifuged at high speed, and then the particles were resuspended in PBS or DMEM. SPP@ZnPc and Cal@ZnPc were prepared similarly, but add SPP@DOPA or Cal@DOPA instead of Oxa(IV)@DOPA. Oxa(IV)@LiP was prepared without adding ZnPc. ZnPc@LiP was prepared without adding Oxa(IV)@DOPA.

### Characterization of Oxa(IV)@ZnPc nanoparticles

Particle size and zeta potential of Oxa(IV)@ZnPc were measured by dynamic light scattering (DLS) using a Malvern Zetasizer Nano ZS90 (Malvern Instruments, Malvern, UK). Transmission electron microscopy (TEM) (JEM-1400, JEOL, Japan) was applied to measure the size and morphology of nanoparticles. The coordination structure of Oxa(IV)@DOPA was confirmed by Fourier transform infrared spectroscopy (FTIR) (VERTEX 70, Bruker, Germany). X-ray photoelectron spectroscopy (XPS) (Thermo Fisher Scientific, ESCALAB 250) was performed to investigate the atomic composition of Oxa(IV)@DOPA on the surface. Graphite Furnace Atomic Absorption Spectrometer (GFAAS, AAnalyst 800, Perkin–Elmer, USA) was applied to detect the Oxa loading content in nanoparticles. The content of Zn was detected by Inductively Coupled Plasma-Atomic Emission Spectrometry (ICP-AES, Optima 8300, Perkin–Elmer). The amount of ZnPc and calcein was determined by fluorescence spectrometer (Fluoromax-4, HORIBA, USA, Ex = 670 nm, Em = 690 nm). The UV-vis and fluorescence spectrum of Oxa(IV)@ZnPc, SPP@ZnPc and Cal@ZnPc were acquired by UV − vis spectroscopy (TECHCOMP, UV2600 spectrophotometer) and fluorescence spectroscopy, respectively.

The ^1^O_2_ produced by Oxa(IV)@ZnPc was detected by ^1^O_2_ sensor SOSG. Briefly, Oxa(IV)@ZnPc and free ZnPc aqueous solution with the same amount of ZnPc were mixed with SOSG stock (1 mM) at final concentration 1 µM. The mixed solution was exposed to a laser (20 mW cm^−2^, 671 nm) or kept in dark for different times respectively, and then the increase in fluorescence intensity of SOSG at Ex = 504 nm and Em = 525 nm, were applied to indicate the production of ^1^O_2_. The stability of Oxa(IV)@ZnPc in PBS supplemented with 10% FBS was evaluated by monitoring particle size changes by DLS for one week.

For in vitro drug release of Oxa from Oxa(IV)@ZnPc, briefly, 1 mL of Oxa(IV)@ZnPc suspension was sealed in dialysis bag (MWCO: 3500 Da), and then immersed in 50 mL of PBS buffer (pH 7.4, 6.5, and 5.0) with stirring at 100 rpm in a shaker at 37 °C, 2 mL of sample was withdraw from the medium and replaced with an equal volume of buffer solution. The samples were dried, redissolved in 1% HNO_3_, and the platinum content was detected by GFAAS. For in vitro drug release of ZnPc from Oxa(IV)@ZnPc, PBS buffer containing 2% tween 80 (pH 7.4, 6.5, and 5.0) was used, and the content of ZnPc was directly detected by fluorescence spectrometer (Ex = 670 nm, Em = 690 nm).

### Cellular uptake, ROS generation, and cytotoxicity of Oxa(IV)@ZnPc

The cellular uptake and intracellular ROS generation of Oxa(IV)@ZnPc were evaluated by confocal laser scanning microscopy (CLSM) (LSM 710, Zeiss, Germany). 4T1 cells were seeded on glass coverslips in a 12-well plate (1 × 10^5^ cells/well) and incubated overnight. Cells were incubated with Oxa(IV)@ZnPc containing 2.5 µM of ZnPc for 2 h, 4 h, 8 h, and 12 h for uptake studies. For intracellular ROS generation, after incubation for 12 h, the drug media was replaced by DCFH-DA (10 μM) and maintained for 30 min, then the cells were washed, followed by exposing or not to 20 mW cm^−2^ LED light (671 nm) for 5 min, then fixed with 4% paraformaldehyde, stained with DAPI and directly visualized by CLSM.

To evaluate the cytotoxicity of nanoparticles, 4T1 cells were seeded on 96-well plates (2.5 × 10^3^ cells/well) overnight, and then cells were incubated with Oxa, Oxa(IV)@Lip, SPP@ZnPc, and Oxa(IV)@ZnPc at various drug concentrations for 24 h. The drug media was then replaced with fresh media, and the cells were irradiated or not with a LED light (671 nm, 20 mW cm^−2^) for 5 min, and then incubated for another 24 h prior to MTT assay, and the absorbance at 490 nm was recorded by a microplate reader (ELX800, Bio-Tek, USA). For live/dead cell imaging, when incubation for 12 h after 5 min of irradiation (671 nm, 20 mW cm^−2^), 4T1 cells were co-stained by FDA and PI to distinguish live and dead cells respectively, and then imaged by an inverted fluorescent microscope (IX73, Olympus, Japan).

### Induction of ICD and apoptosis by Oxa(IV)@ZnPc

In vitro CRT exposure induced by Oxa(IV)@ZnPc was investigated by flow cytometry and IF staining. 4T1 cells were seeded on glass coverslips in 24-well plates or six-well plates for IF or Flow cytometry, respectively. After 24 h of incubation, cells were treated with Oxa, SPP@ZnPc, and Oxa(IV)@ZnPc at an equivalent platinum dose of 6.25 µM and ZnPc dose of 0.625 µM for 24 h, and then cells were exposed to LED light or not (671 nm, 20 mW cm^−2^) for 5 min. After 4 h of further incubation, cells were washed and fixed with 1% paraformaldehyde for 10 min, blocked with 5% BSA for 1 h, and then cells were incubated with primary anti-CRT antibody for 1.5 h, washed again and incubated with Alexa Fluor 488 anti-rabbit secondary antibody for 1 h, washed and then analyzed by flow cytometry, or stained with DAPI and visualized by CLSM. HMGB-1 concentrations in the supernatant of 4T1 cells following the indicated treatment were detected by an ELISA kit according to the manufacturer’s protocol. For the extracellular secretion of ATP, the supernatants of 4T1 cells that were further incubated for 12 h after 5 min light irradiation were collected and detected by a ATP assay kit.

To evaluate apoptosis induced by Oxa(IV)@ZnPc, 4T1 cells seeded in six-well plates (1 × 10^5^ cells/well) were incubated with Oxa, SPP@ZnPc, and Oxa(IV)@ZnPc (6.25 µM of platinum and 0.625 µM of ZnPc) for 24 h, then cells were irradiated or not with LED light (671 nm, 20 mW cm^−2^) for 5 min, and then further incubated for 12 h. All cells were collected, stained with Annexin V/PI Apoptosis Kit according to the manufacturer’s instructions, and then detected by flow cytometry.

### Preparation and characterization of Oxa(IV)@ZnPc loaded BMMs (Oxa(IV)@ZnPc@M)

The optimal loading efficiency of BMMs was screened by estimating the incubation time, the amount of drug within BMMs and cell viability. To screen the optimal incubation time, BMMs were seeded on glass coverslips in 12-well plates (1 × 10^5^ cells/well) for CLSM. BMMs were co-incubated with Cal@ZnPc for 0.5 h, 1 h, 2 h, and 4 h for CLSM observation. In addition, the quantitative fluorescence intensity of intracellular ZnPc was further performed using fluorescence spectroscopy (Ex = 670 nm, Em = 690 nm). All of the experiments above confirmed that 2 h incubation could effectively load the nanoparticles.

To screen the optimal incubation concentration of nanoparticles, different concentrations of platinum (500, 400, 300, 150, and 75 µM) and ZnPc (200, 160, 120, 60, and 30 µM) of Oxa(IV)@ZnPc were incubated with BMMs for 2 h, then cells were collected and washed three times with PBS and then counted. For platinum content detection, cells were digested with concentrated HNO_3_, heated at 80 °C for several hours, then dried and dissolved with 1% HNO_3_, and finally detected by GFAAS. For ZnPc content detection, after completely removing the solution by evaporation, the samples were dissolved with CHCl_3_, then evaporated and redissolved in DMSO, and finally analyzed by fluorescence spectroscopy (Ex = 670 nm, Em = 690 nm). Subsequently, the cell viability of BMMs after drug incubation was evaluated. Briefly, BMMs were seeded on 96-well plates overnight. Cells were then treated with free Oxa, Oxa(IV)@ZnPc, and ZnPc@Lip at various platinum (500, 400, 300, 150, and 75 µM) or ZnPc (200, 160, 120, 60, and 30 µM) concentrations for 2 h. After that, the drug media was removed and gently washed with PBS, and then further cultured with fresh medium for 2 h, 6 h, 12 h, and 24 h, respectively. At scheduled time points, cell viability was determined using the CCK-8 kit according to the manufacturer’s instruments.

Under the optimized incubation concentration and time, the drug loading capacity of BMMs and cell viability of BMMs after drug loading were further evaluated by TEM and live/dead staining assay, respectively. After incubation with Oxa(IV)@ZnPc for 2 h, the cells were washed, pelleted by centrifugation (1000×*g*, 10 min), and fixed with 2.5% glutaraldehyde at 4 °C for 1 day. The pellet was then treated with 1% osmium tetroxide (OsO_4_) for 1.5 h, dehydrated in a series of alcohol, and then substituted with 100% acetone. The cells were then embedded in SPI 812, solidified at 60 °C and sliced to a thickness of 100 nm, and finally observed by TEM. BMMs seeded in 12-well plates were incubation with Oxa(IV)@ZnPc for 2 h, BMMs were co-stained by FDA and PI and then imaged for live and dead cells. In the end, the loading process was optimized by incubating at 400 µM platinum and 160 µM ZnPc for 2 h.

### Cell migration assay

Cell migration assay was conducted using 24-well polycarbonate membrane inserts (8 µm pore size, Corning Inc., NY, USA). BMMs and Oxa(IV)@ZnPc@M suspensions (200 µL) were seeded in the upper chamber of the inserts (5 × 10^4^ cells/well), 800 µL cell growth medium (10% FBS), or tumor conditioned medium (TCM) collected from 4T1 cells were added to the bottom chambers. After migration for 6 h, the migratory cells were fixed with 4% paraformaldehyde and stained with crystal violet. Cells on the top surface of the membrane were removed by a cotton-tipped swab. The migratory cells on the bottom of the membrane were imaged (EVOS FL Auto, Life Technologies, USA) and counted microscopically in five random fields.

### In vitro cytotoxicity and apoptosis assay

To investigate the cytotoxicity of Oxa(IV)@ZnPc@M, 4T1 cells were seeded into 96-well plates (2.5 × 10^3^ cells/well) and allowed to adapt for 24 h. BMMs and Oxa(IV)@ZnPc@M were co-incubated with 4T1 cells at various counts of cells. After 24 h of incubation, the cells were irradiated or not with a LED light (671 nm, 20 mW cm^−2^) for 10 min, then cultured for another 24 h prior to MTT assay. Cytotoxicity was further confirmed by live/dead cell imaging, 12 h after the 10 min light irradiation (671 nm, 20 mW cm^−2^), the treated 4T1 cells were co-stained by FDA and PI and then imaged by an inverted fluorescent microscope.

To measure cell apoptosis induced by Oxa(IV)@ZnPc@M, 4T1 cells seeded in six-well plates (1 × 10^5^ cells/well) were treated with BMMs and Oxa(IV)@ZnPc@M at platinum concentration 6.25 µM and ZnPc concentration 3.12 µM (~1.25 × 10^5^ cells) for 24 h, then cells were exposed to LED light (671 nm, 20 mW cm^−2^) or not for 10 min, after further incubation for 12 h, all cells were harvested and stained with Annexin V/PI Apoptosis Kit according to the manufacturer’s instructions, and then analyzed by flow cytometry (Guava EasyCyte 6-2L, Merck Millpore).

### In vitro ICD induction

In vitro CRT exposure induced by Oxa(IV)@ZnPc@M was evaluated by IF staining and flow cytometry. 4T1 cells were separately seeded on glass coverslips in 24-well plates (2.5 × 10^4^ cells/well) or six-well plates (1 × 10^5^ cells/well) for IF or flow cytometry, respectively. Twenty-four hours later, the cells were exposed to BMMs and Oxa(IV)@ZnPc@M (platinum 6.25 µM and ZnPc 3.12 µM) for 24 h, then cells were exposed to LED light or not (671 nm, 20 mW cm^−2^) for 10 min. After further incubation for 4 h, cells were washed, fixed with 1% paraformaldehyde for 10 min, blocked with 5% BSA for 1 h, and then cells were incubated with rabbit anti-CRT antibody for 1.5 h, washed again and incubated with Alexa Fluor 488-conjugated secondary antibody for 1 h, washed and then analyzed by flow cytomtry (Guava EasyCyte 6-2 L, Merck Millpore), or stained with DAPI and observed by CLSM. For extracellular ATP secretion and HMGB-1 release detection, 4T1 cells were seeded in six-well plates and allowed to adhere for 24 h. Cells were exposed to BMMs and Oxa(IV)@ZnPc@M (platinum 6.25 µM and ZnPc 3.12 µM, ~1.25 × 10^5^ cells) for 24 h, and then irradiated or not with LED light (671 nm, 20 mW cm^−2^) for 10 min. The cell culture supernatants were collected at 4 h and 12 h after irradiation for HMGB-1 and ATP detection, respectively.

### In vitro drug release from Oxa(IV)@ZnPc@M

To investigate drug release behaviors, BMMs were loaded with Oxa(IV)@ZnPc for 2 h, washed three times with ice-cold PBS, and then dispersed in 1 mL of fresh medium and placed in 37 °C incubator (5 million cells). At predetermined time points, the mixture was centrifuged (1000x*g*, 5 min), supernatant (400 µL) was withdrawn, and an equal volume of fresh medium was added. For light-triggered drug release, after the supernatant at 4 h were taken, cells were exposed to a LED light (671 nm, 20 mW cm^−2^) for 10 min. The amount of ZnPc was detected by fluorescence spectroscopy.

To evaluate the cytotoxicity of the drug release medium to 4T1 cells, BMMs and Oxa(IV)@ZnPc@M were seeded in six-well plates with complete growth medium, supernatants were collected and centrifuged (1000 × *g*, 10 min) to remove cell debris at scheduled time points (6 h, 12 h, 24 h, and 48 h). 4T1 cells pre-cultured in 96-well plates overnight were incubated with the supernatant taken from BMMs and Oxa(IV)@ZnPc@M for 24 h and then cells were irradiated or not with LED light (671 nm, 20 mW cm^−2^, 10 min), and then incubated for additional 24 h prior to MTT assay. To evaluate the laser-triggered drug release, after incubating for 4 h, BMMs and Oxa(IV)@ZnPc@M were exposed or not to LED light (671 nm, 20 mW cm^−2^) for 10 min. At 4 h and 8 h after irradiation, the supernatants were collected after centrifugation (1000×*g*, 10 min). The 4 h supernatants was applied for MTT and cellular uptake assays, and the 8 h supernatants was applied for cell apoptosis assays.

### BMMs cell phenotype in vitro and in vivo

The cell phenotype change in vitro was evaluated by detecting the cytokines secretion and the surface markers of macrophage (M1 maker CD80 and CD86). For cytokines secretion detection, BMMs and Oxa(IV)@ZnPc@M with the same cell numbers and culture conditions were seeded on six-well plates. Cell supernatants from BMMs and Oxa(IV)@ZnPc@M were collected at different times points (2 h, 4 h, 8 h, 12 h, and 24 h), and the concentrations of cytokines (TNF-α, IL-6, and IL-10) were measured by ELISA kits. For surface marker detection, BMMs were seeded in six-well plates and treated with Oxa(IV)@ZnPc, free ZnPc, SPP@ZnPc, ZnPc@Lip, and free Oxa for 2 h, then cells were washed, after further incubation for 12 h, all cells were harvested, stained with anti-CD80-FITC and anti-CD86-PE, and then analyzed by flow cytometry (Guava EasyCyte 6-2L, Merck Millpore). Typical LPS (500 ng/mL) and IFN-γ (40 ng/mL) treated M1 macrophages, and IL-4 (40 ng/mL) treated M2 macrophages were used as control, and the expression of iNOS and Arg-1 by M1 macrophages and M2 macrophages respectively were verified by western blot.

Next, whether the drug loaded macrophages can retain the M1 phenotype in tumor was verified by detecting the surface marker of macrophages using flow cytometry (CytoFLEX-S, Beckman Coulter). The cell membrane dye DiD was used to label macrophages. In order to eliminate the interference of the fluorescent dye ZnPc, we used Oxa(IV)@Lip (lipid coated but without ZnPc loading) to replace Oxa(IV)@ZnPc for drug loading. The DiD labeled blank BMMs and drug loaded BMMs were named as BMMs-DiD and Oxa(IV)@Lip@M-DiD. In addition, typical LPS (500 ng/mL) and IFN-γ (40 ng/mL) treated M1 BMMs (M1-DiD) were served as positive control. Briefly, mice bearing 4T1 tumors were intravenously injected with saline, BMMs-DiD, M1-DiD, and Oxa(IV)@Lip@M-DiD. At 8 h post-injection, tumors were harvested, cut into small pieces and ground up, then passed through 200 μm and 70 μm filter to obtain single-cell suspensions. The tumor cells were first incubated with anti-mouse CD16/32 antibody to prevent non-specific staining, then it was further stained with anti-CD80-PE and anti-CD86-PC7 according to manufacturer’s instructions, and then analyzed by flow cytometry (CytoFLEX-S, Beckman Coulter).

### Bilateral 4T1 and EMT6 tumor model

To establish a bilateral 4T1 and TMT6 tumor model, female Balb/c mice (18-20 g) were injected subcutaneously with 1 × 10^6^ 4T1 or EMT6 cells in the left flank (primary tumor), one week or 6 days after inoculation of 4T1 or EMT6 respectively, 1 × 10^5^ 4T1 cells or 5 × 10^5^ EMT6 cells were injected into the medullary cavity of the right tibia through a 26-gauge needle under anesthesia (bone metastatic tumor).

### Pharmacokinetics and biodistribution

To evaluate in vivo pharmacokinetics profiles, SD rats (*n* = 3) were intravenously injected with free Oxa, Oxa(IV)@ZnPc and Oxa(IV)@ZnPc@M at an equal platinum dose of 1 mg/kg. Blood samples were harvested at 10 min, 20 min, 40 min, 60 min, 1 h, 2 h, 4 h, 8 h, 12 h, and 24 h post-injection via the tail of rats. The samples were centrifuged to obtain plasma, which were digested in aqua regia, then heated and dried, finally redissolved in 1% HNO_3_ and detected by GFAAS.

To investigate the in vivo distribution, mice bearing 4T1 primary and bone metastatic tumors were randomly divided into three groups (*n* = 3), and intravenously injected with free ZnPc, Oxa(IV)@ZnPc, and Oxa(IV)@ZnPc@M with an equivalent ZnPc dose of 1 mg/kg. Mice were imaged by an in vivo imaging system (IVIS) (Lumina XRMS Series III, PerkinElmer, USA, Ex = 660 nm, Em = 710 nm) at 2 h, 4 h, 8 h, 12 h, and 24 h post-injection. At 24 h post-injection, hearts, livers, spleens, lungs, kidneys, primary tumors, right tumor-bearing hindlimbs, and left healthy hindlimbs were excised for ex vivo imaging.

### In vivo antitumor efficiency on 4T1 tumor model and EMT6 tumor models

For 4T1 tumor model, Balb/c female mice (5–6 weeks) were subcutaneously injected with 1 × 10^6^ 4T1 cells in the left flank (primary tumor). When the primary tumors reached ~80 mm^3^, which was ~10 days after inoculation, 1 × 10^5^ Luc-4T1 cells were intra-tibia injected into the right tibia using a 26-gauge needle under anesthesia. Three days after intra-tibia injection, mice were intraperitoneally injected with d-luciferin (150 mg/kg), and 10–20 min later, bioluminescence imaging (BLI) was performed to image the bone metastatic tumors by a noninvasive IVIS imaging system. The next day, the mice bearing primary tumors (100–150 mm^3^) and bone metastatic tumors were randomly divided into eight groups (*n* = 5), and intravenously injected with various formulations including following: (1) PBS (+), (2) free Oxa, (3) Oxa(IV)@ZnPc, (4) Oxa(IV)@ZnPc@M, (5) BMMs + anti-PD-L1, (6) Oxa(IV)@ZnPc (+), (7) Oxa(IV)@ZnPc@M (+), (8) Oxa(IV)@ZnPc@M (+) + anti-PD-L1. “(+)” represents laser irradiation. The injection was conducted every 2 days for three times at an equal platinum dose of 1 mg/kg and ZnPc dose of 0.75 mg/kg (~1 × 10^6^ cells/mouse). The fourth hour and 24 h post-injection, the primary tumors were irradiated with a NIR laser (671 nm, 250 mW cm^−2^) and continued for 10 min, respectively. After the second irradiation, mice were intraperitineally injected with anti-PD-L1 antibody (100 μg/mouse). Tumor size of primary tumors and body weight were monitored every other day. Tumor volume was measured using calipers and calculated as length × width^2^ × 0.5. The growths of bone metastatic tumors were recorded by BLI at days 3, 10, 16, and 22 after 4T1 cells inoculation as described above.

At the end of experiment, mice were killed and tumors were excised, the primary tumors (*n* = 5) were weighed and photographed. Primary tumors of each group were fixed with 4% paraformaldehyde and sectioned for hematoxylin-eosin (H&E) and terminal deoxynucleotidyl transferase dUTP nick-end labeling (TUNEL) staining analysis. The excised bone metastatic hindlimbs were fixed with 4% paraformaldehyde, and analyzed by a Biograph 3D micro-CT device (ZKKS-MCT-Sharp-I, Caskaisheng, China). The 3D images were reconstructed and analyzed using the ZKKS-Micro CT 4.1 analysis software, and the morphometric parameters, such as bone volume/tissue volume (BV/TV), trabecular numbers (Tb. N), and bone surface (BS), were calculated automatically with an identical ROI. Bone tissues were decalcified in 10% EDTA for 3 weeks. Bone-metastatic tumors and bone tissues were embedded in paraffin and sectioned; tumors were stained with H&E, and bone tissues were stained with H&E and tartrate-resistant acid phosphatase (TRAP). Meanwhile, lungs were also resected, fixed with 4% paraformaldehyde, then sectioned and stained with H&E to evaluate the metastatic area.

For EMT6 tumor model, Balb/c female mice (5–6 weeks) were subcutaneously injected with 1 × 10^6^ EMT6 cells in the left flank (primary tumor). When the primary tumors reached ~50 mm^3^, which was ~6 days after inoculation, 5 × 10^5^ EMT6 cells were intra-tibia injected into the right tibia using a 26-gauge needle under anesthesia. Six days after intra-tibia injection, the mice bearing primary tumors (100–150 mm^3^) and bone metastatic tumors were randomly divided into various groups (*n* = 6), and intravenously injected with various formulations including following: (1) PBS ( + ), (2) free Oxa, (3) Oxa(IV)@ZnPc, (4) Oxa(IV)@ZnPc@M, (5) BMMs + anti-PD-L1, (6) Oxa(IV)@ZnPc (+), (7) Oxa(IV)@ZnPc@M (+), (8) Oxa(IV)@ZnPc@M (+) + anti-PD-L1, (9) Oxa + anti-PD-L1, and (10) Oxa(IV)@ZnPc (+) + anti-PD-L1. “(+)” represents laser irradiation. The treatment schedule and dosage were consistent with the 4T1 model. Tumor size and body weight were monitored every other day. Tumor volume was measured using calipers and calculated as length × width^2^ × 0.5.

### Immune mechanism evaluation in vivo

The mice bearing bilateral 4T1 or EMT6 tumor were randomly assigned into eight groups, and received the same treatment regimen as the antitumor experiment. For the detection of DCs maturation in 4T1 model, the next day after the first treatment, the inguinal lymph nodes of each treated mouse were harvested, and then ground up to obtain single-cell suspensions. These cells were stained with eFluor^TM^ 450, anti-CD11c-FITC, anti-CD80-PE, and anti-CD86-PC7 antibodies, then analyzed by flow cytometry (CytoFLEX-S, Beckman Coulter) to delineate mature DC cells.

For the detection of other immune-related cells, the next day after the last irradiation, serum from the mice were collected, and the production of TNF-α and IFN-γ were detected by ELISA kits following standard protocols. Meanwhile, tumors were harvested, cut into small pieces and ground up, then passed through 200 μm and 70 μm filter to obtain single-cell suspensions. All the cells were treated with ACK Lysis Buffer, washed, and then collected by centrifugation. Cells were first incubated with anti-mouse CD16/32 antibody to prevent non-specific staining. The tumor cells were stained with anti-CD3-FITC, anti-CD8a-PE, and anti-CD4-APC antibodies for CTLs infiltration evaluation. For Treg evaluation, tumor cells were stained with anti-CD3e-FITC, anti-CD4-APC, and anti-Foxp3-PE antibodies. To analyze tumor-associated macrophage (TAMs), tumor cells were stained with anti-CD11b-APC, anti-F4/80-FITC, anti-CD206-PE, and anti-CD86-PC7. In addition, tumor cells were stained with anti-CD3e-FITC, anti-CD8a-PE, anti-IFN-γ-APC, and anti-TNF-α-PC7 for assessing IFN-γ and TNF-α in CD8^+^ T cells. Live/dead cell discrimination was conducted using eFluor^TM^ 780 according to manufacturer’s instructions. The immune-related cells in the metastatic tumor of mice bearing bilateral EMT6 were evaluated using the same procedure as well.

In addition, the primary tumors of 4T1 were isolated and fixed, embedded in paraffin and sectioned. IF and immunohistochemistry (IHC) staining were performed for CRT and HMGB-1 analysis, respectively. The bone metastatic tumors of 4T1 were also sectioned, IF and IHC staining were conducted to analyze the presence of CD4^+^ and CD8^+^ T cells in tumors, respectively.

Immune memory effect. The immune memory effect was conducted by tumor rechallenge studies. 4T1 cells (1 × 10^6^) were subcutaneously injected in the left flank. When the tumor volume reached ~100–150 mm^3^, mice were randomly assigned into eight groups (*n* = 8), and received regiments specified in the antitumor experiment. The next day after the last irradiation, the primary tumors were surgically resected. After 15 days, 1 × 10^5^ Luc-4T1 cells were challenged on the right tibia by intra-tibia injection. Tumor size of bone metastatic tumors were measured every other day and the tumor volume was calculated as length × width^2^ × 0.5. Meanwhile, BLI was also conducted to monitor the growths of bone metastatic tumors on days 5, 10, and 15 after inoculation. In addition, survival rates of mice after different treatment were also recorded.

### Toxicity evaluation

Healthy Balb/c mice were randomly divided into seven groups (*n* = 10, five for single dose and the other for multiple dose), and were intravenously injected with PBS, free Oxa, Oxa(IV)@ZnPc, BMMs, BMMs + anti-PD-L1, Oxa(IV)@ZnPc@M, and Oxa(IV)@ZnPc@M + anti-PD-L1 at platinum dose of 1 mg/kg and ZnPc dose of 0.75 mg/kg (~1 × 10^6^ cells/mouse) on day 0 (single dose) and days 0, 2, and 4 (multiple dose). On day 1 (single dose) and day 5 (multiple dose), whole blood and serum of each mouse were collected for complete blood count test and biochemistry analysis, respectively. Biochemistry index, including alanine transaminase (ALT) and aspartate aminotransferase (AST), and blood urea nitrogen (BUN) and creatinine (CREA), were detected to indicate liver function and kidney function, respectively. Meanwhile, major organs, including hearts, livers, spleens, lungs, and kidneys, were excised, fixed with 4% paraformaldehyde and sectioned for H&E staining.

### Statistical analysis

All the data were presented as mean ± standard deviation (SD). The unpaired two-tailed *t* test was used for two group comparisons and ordinary one-way (or two way) ANOVA with a Tukey’s test or Dunnett’s test were used for multiple group comparisons. All the statistical analyses were performed using Graphpad Prism 8.3.0 (GraphPad software, CA, USA).Statistical significance was set at *p < 0.05, **p < 0.01, ***p < 0.001.

### Reporting summary

Further information on research design is available in the Nature Research Reporting Summary linked to this article.

## Supplementary information

Supplementary Information

Reporting Summary

## Data Availability

The data that support the findings of this study are available within the Article, Supplementary Information or Source Data file. Source data are provided with this paper.

## Source data

Source data

## Peer Review File

Peer Review File
