# Supplementary material for: Engineered macrophages as near-infrared light activated drug vectors for chemo-photodynamic therapy of primary and bone metastatic breast cancer
Source: Nat Commun. 2021 Jul 14;12:4310. doi: 10.1038/s41467-021-24564-0 (PMC8280231; doi:10.1038/s41467-021-24564-0)
Supplement: Supplementary file 1 — Supplementary Information [file 41467_2021_24564_MOESM1_ESM.pdf]

Supplementary Information

Engineered macrophages as near-infrared light activated drug vectors for chemo-photodynamic therapy of primary and bone metastatic breast cancer

Yanjuan Huang, Zilin Guan, Xiuling Dai, Yifeng Shen, Qin Wei, Lingling Ren, Jingwen Jiang, Zhanghong Xiao, Yali Jiang, Di Liu, Zeqian Huang, Xiaoyu Xu, Yong Luo, Chunshun Zhao*

School of Pharmaceutical Sciences, Sun Yat-sen University, Guangzhou, 510006, People's Republic of China.

*Corresponding author: Chunshun Zhao, Tel: +86 20 39943118; Fax: +86 20 39943118.
E-mail address: zhaocs@mail.sysu.edu.cn.

Supplementary Figures

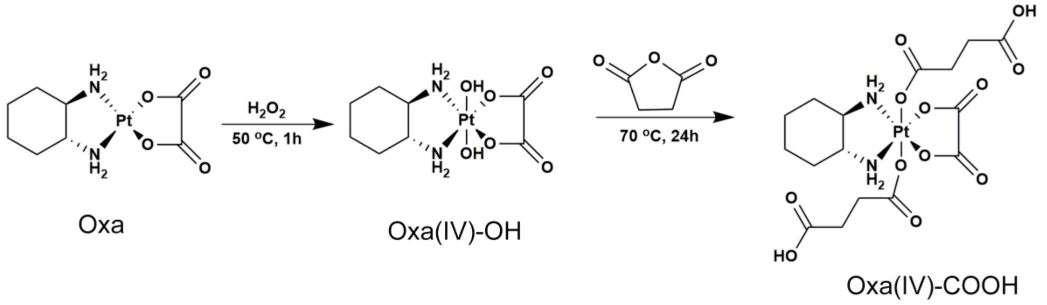

Supplementary Figure 1. Synthesis of Oxa(IV)-OH and Oxa(IV)-COOH.

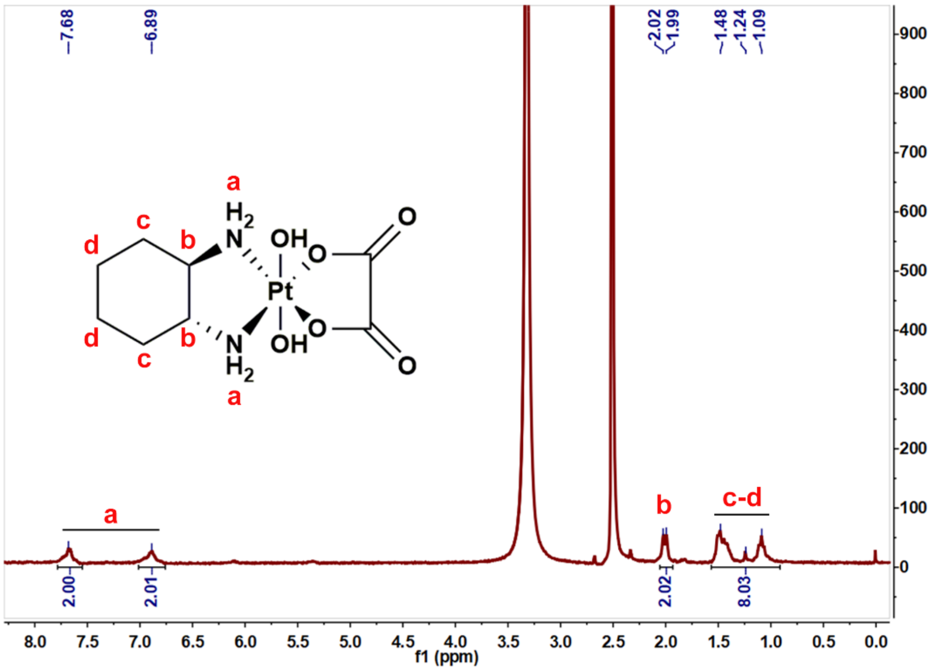

Supplementary Figure 2. ^{1}H NMR spectra of Oxa(IV)-OH in DMSO-d6.

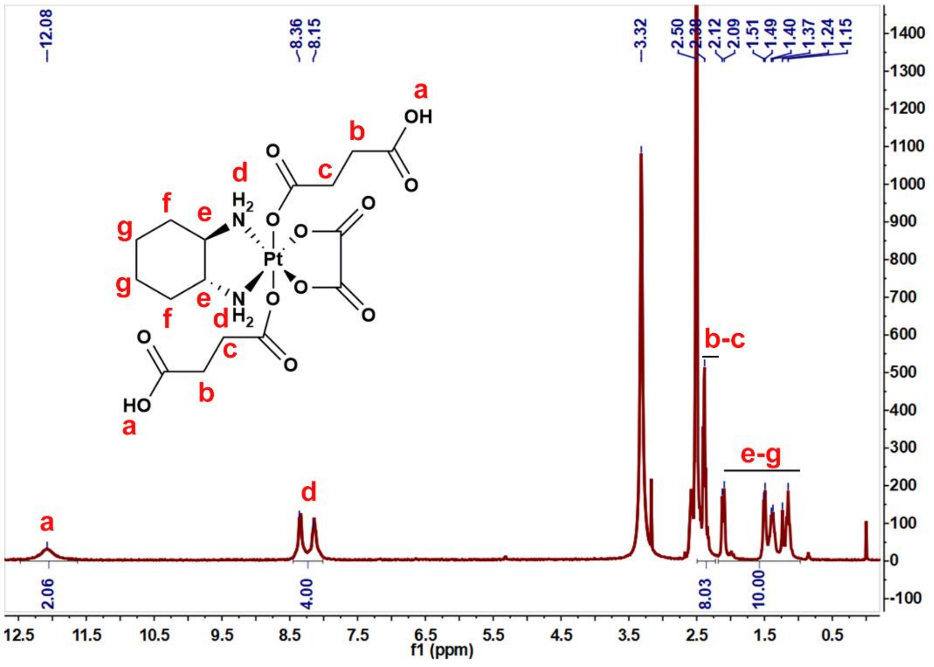

Supplementary Figure 3. ^{1}H NMR spectra of Oxa(IV)-COOH in DMSO-d6.

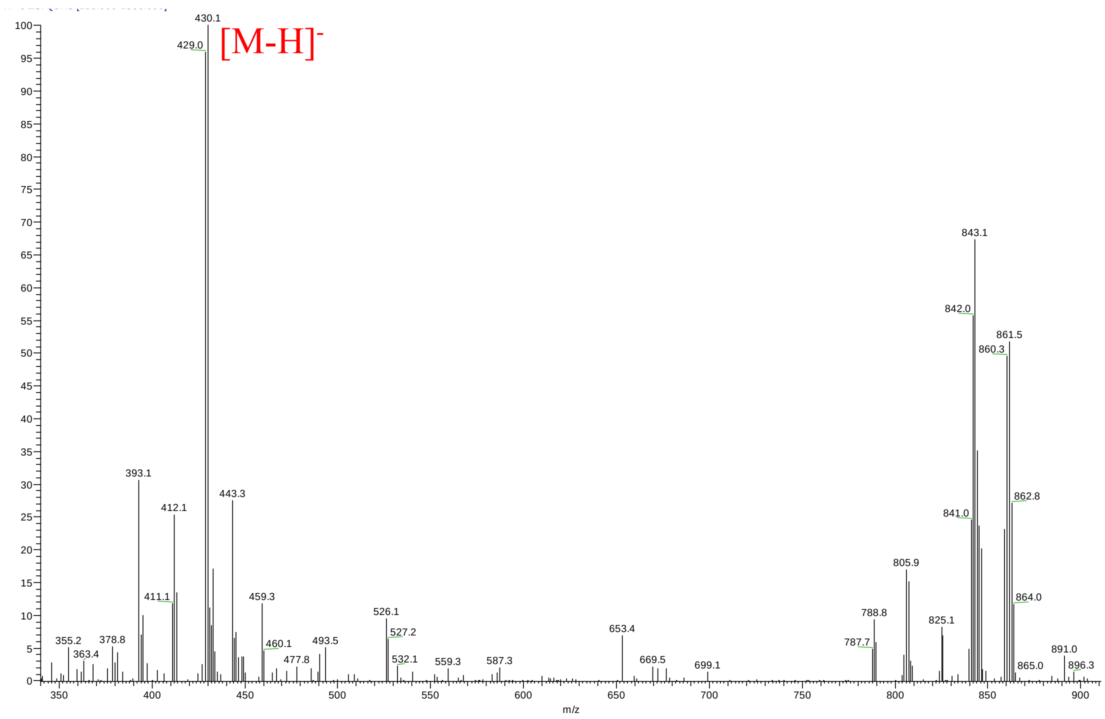

Supplementary Figure 4. ESI-MS spectra of Oxa(IV)-OH.

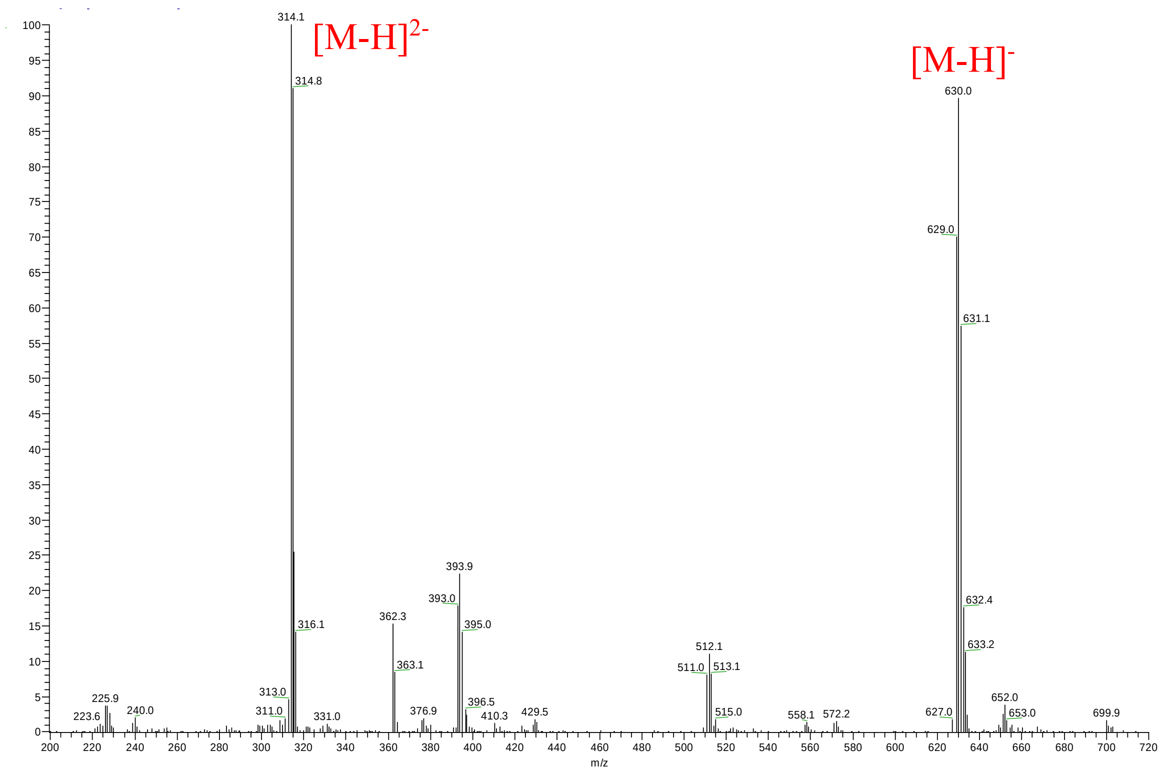

Supplementary Figure 5. ESI-MS spectra of Oxa(IV)-COOH.

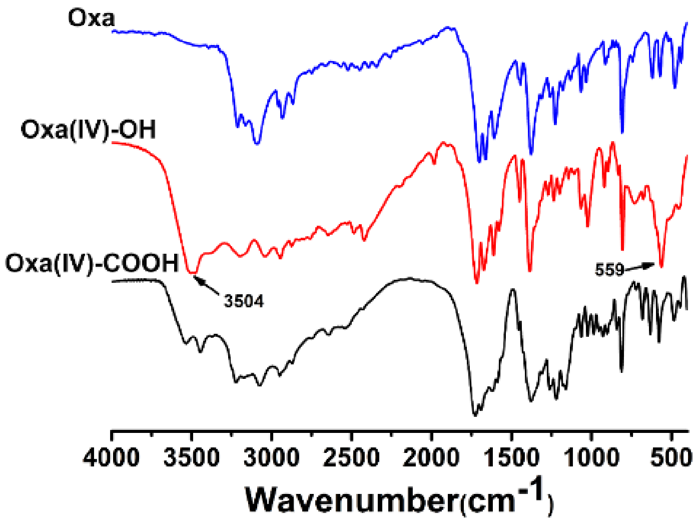

Supplementary Figure 6. FTIR spectra of Oxa, Oxa(IV)-OH and Oxa(IV)-COOH.

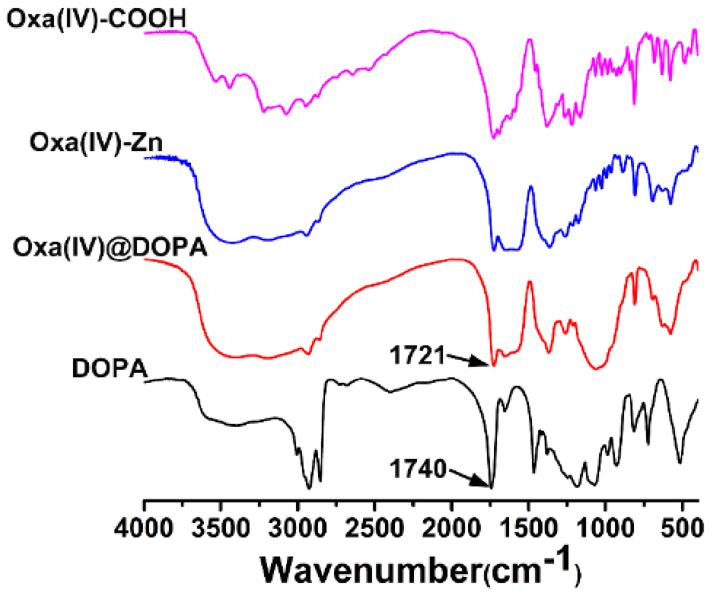

Supplementary Figure 7. FTIR spectra of Oxa(IV)-COOH, Oxa(IV)-Zn (without adding DOPA), Oxa(IV)@DOPA and DOPA.

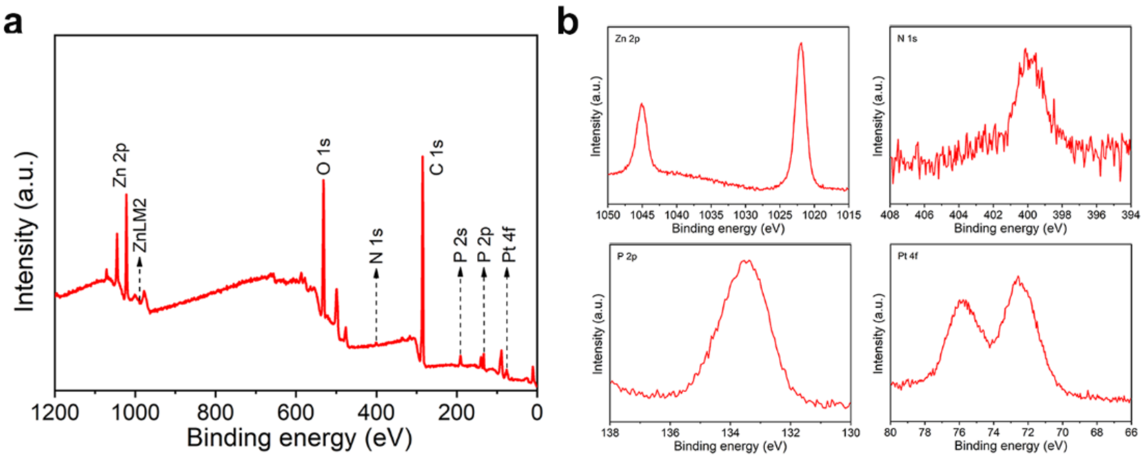

Supplementary Figure 8. (a) XPS survey spectrum of Oxa(IV)@DOPA. (b) The Zn 2p, N 1s, P 2p and Pt 4f core-line spectrum recorded for Oxa(IV)@DOPA.

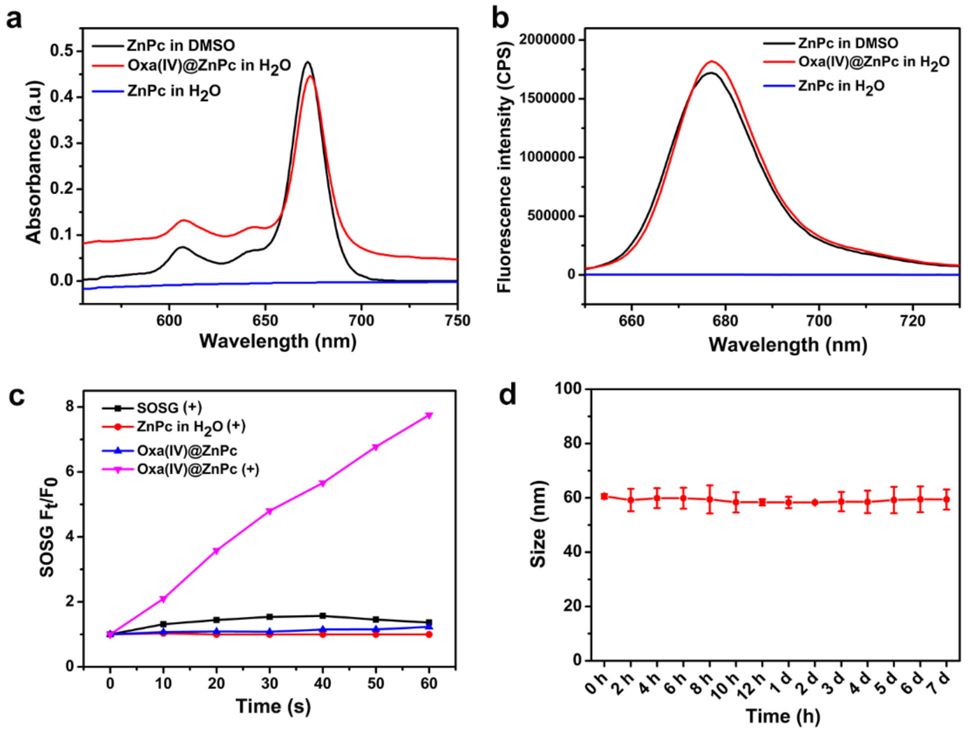

Supplementary Figure 9. (a) UV-vis spectrum and (b) Fluorescence emission spectrum of ZnPc in DMSO, ZnPc and Oxa(IV)@ZnPc in H_2O. (c) Extracellular $^1O_2$ generation capacity detected by SOSG. "(+)" represents light irradiation. (d) In vitro stability of Oxa(IV)@ZnPc in PBS containing 10% FBS for one week (n = 3 technical replicates), this experiment was performed three times with similar results. All data were presented as mean ± SD. Source data are provided as a Source data file.

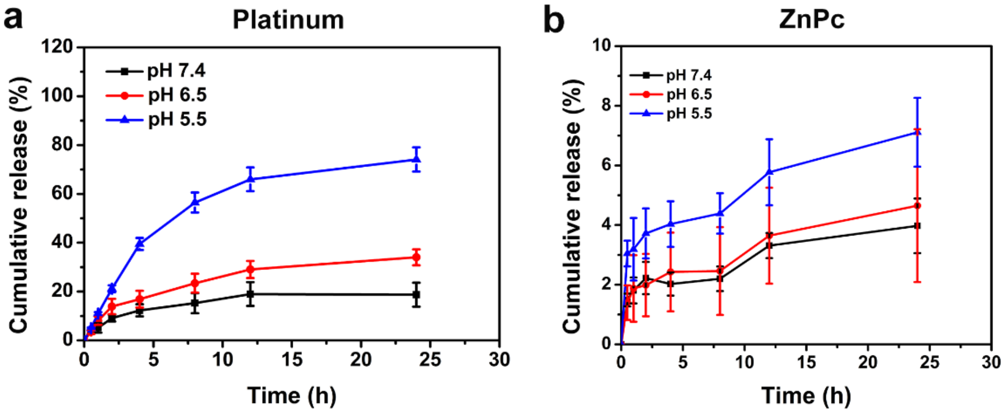

Supplementary Figure 10. (a) In vitro release profile of platinum from Oxa(IV)@ZnPc in PBS at different pH (n = 3 technical replicates). (b) In vitro release profile of ZnPc from Oxa(IV)@ZnPc in PBS containing 2% tween 80 at different pH (n = 3 technical replicates). All data were presented as mean ± SD. Source data are provided as a Source data file.

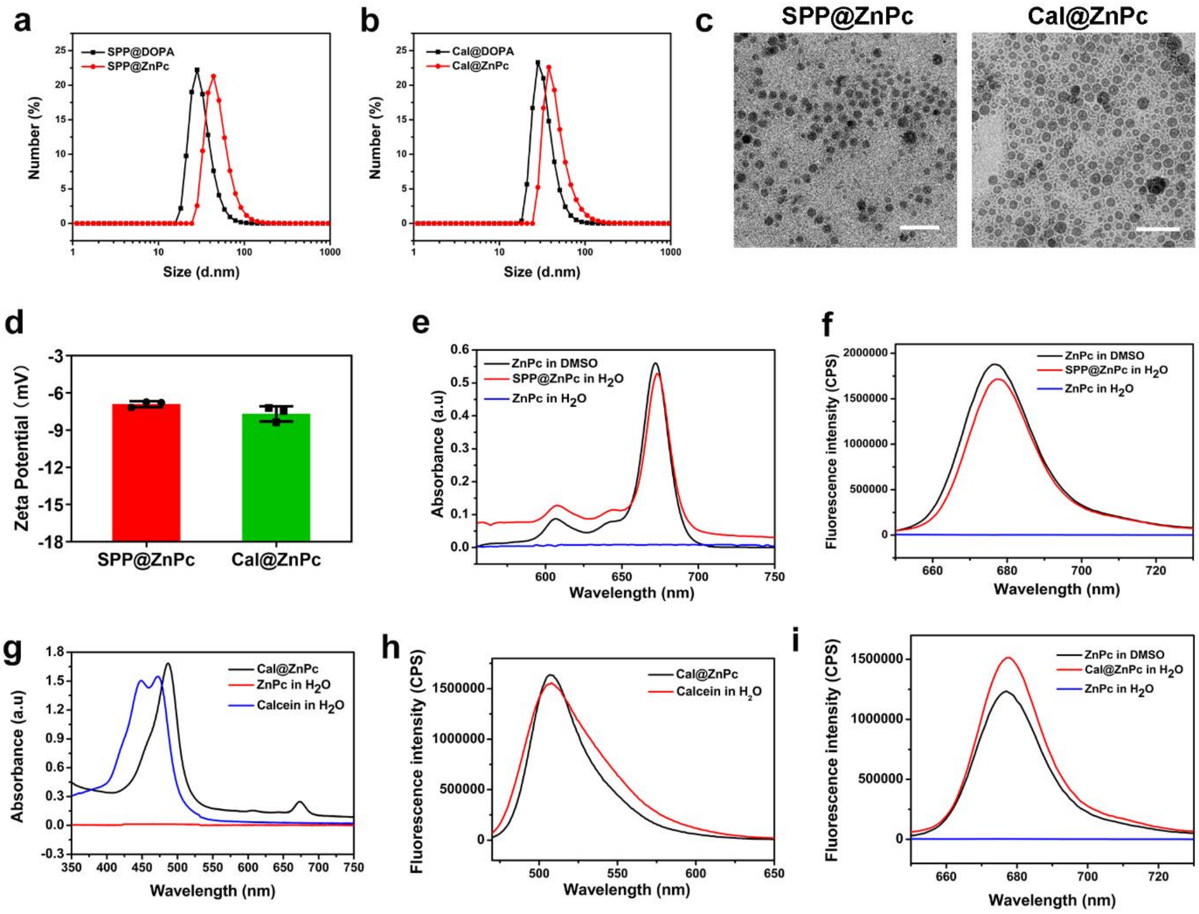

Supplementary Figure 11. Average diameters of (a) SPP@DOPA and SPP@ZnPc, and (b) Cal@DOPA and Cal@ZnPc measured by DLS. (c) TEM images of SPP@ZnPc and Cal@ZnPc without negative stain, this experiment was performed two times with similar results. Scale bars = 100 nm. (d) Zeta potential of SPP@ZnPc and Cal@ZnPc in H_2O (n = 3 technical replicates). (e) UV-vis spectrum and (f) Fluorescence emission spectrum of ZnPc in DMSO, ZnPc and SPP@ZnPc in H_2O. (g) UV-vis spectrum and (h-i) Fluorescence emission spectrum of Cal@ZnPc in H_2O. All data were presented as mean ± SD. Source data are provided as a Source data file.

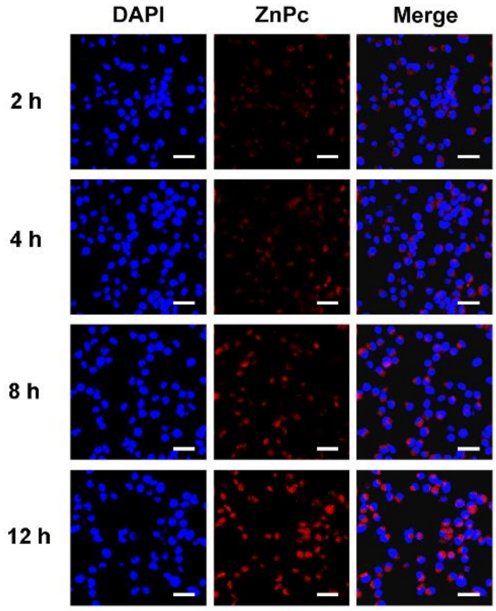

Supplementary Figure 12. Cellular internalization of Oxa(IV)@ZnPc in 4T1 cells at 2 h, 4 h, 8 h and 12 h incubation, this experiment was performed two times with similar results. Scale bars = 30 μm.

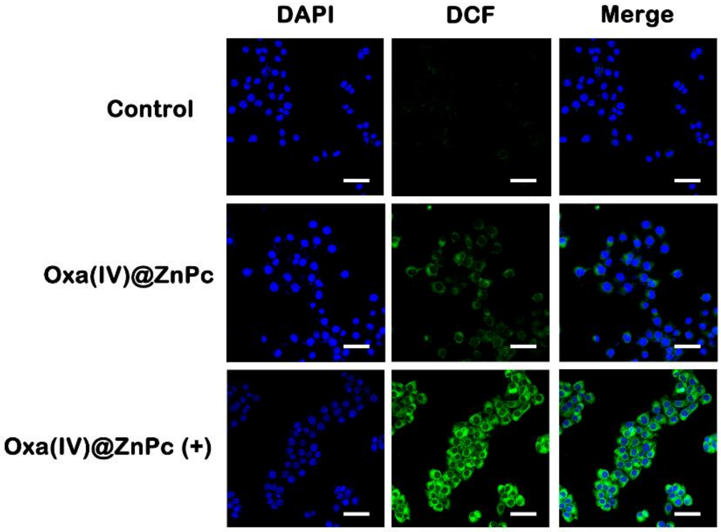

Supplementary Figure 13. Light-triggered ROS generation in 4T1 cells after Oxa(IV)@ZnPc incubation, this experiment was performed two times with similar results. Scale bars = 50 μm.

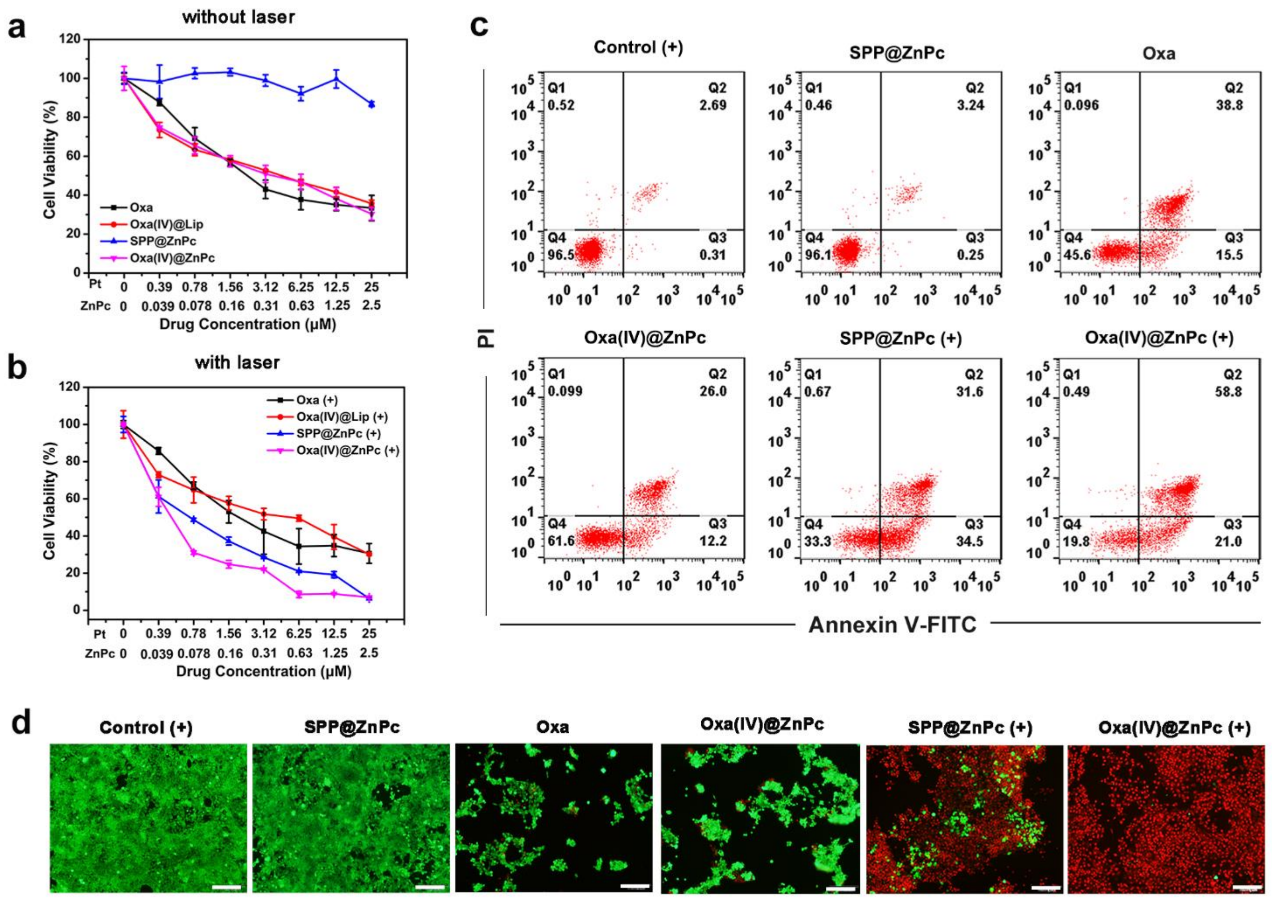

Supplementary Figure 14. Cell viability of 4T1 cells after treated with various drug formulations (a) without or (b) with irradiation (n = 3 technical replicates). (c) Quantitative apoptosis analysis of 4T1 cells stained with Annexin V-FITC/PI. (d) Live/dead cell staining of 4T1 cells with different treatments, this experiment was performed two times with similar results. Scale bars = 200 μm. "(+)" represents with irradiation. All data were presented as mean ± SD.

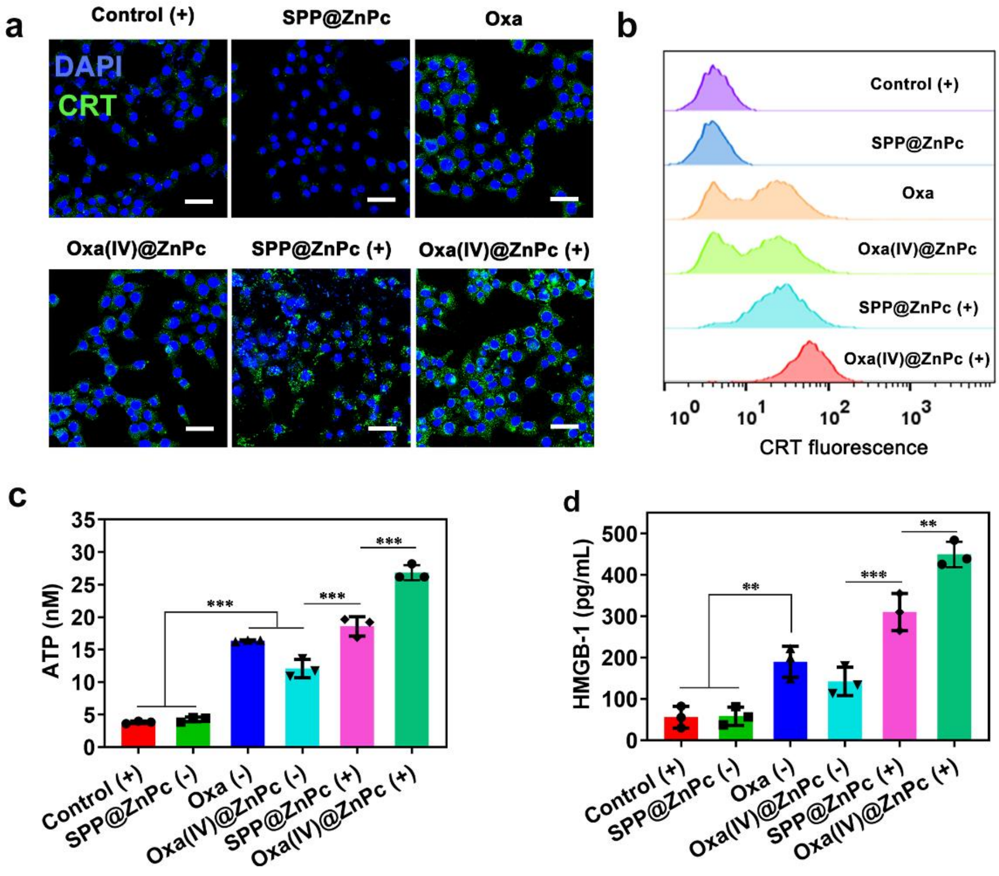

Supplementary Figure 15. (a) CLSM images and (b) FCM examination of CRT exposure on the surface of 4T1 cells, this experiment was performed two times with similar results. Scale bars = 50 µm. Quantitative determination of (c) ATP secretion (****p* < 0.0001, ****p* <0.0001, ****p* <0.0001) and (d) HMGB-1 release of 4T1 cells after various treatments (n = 3 technical replicates, ***p* = 0.0043, ****p* = 0.0005, ***p* <0.0027). All data were presented as mean ± SD. Statistical significance was calculated via ordinary one-way ANOVA with a Tukey's test. **p* <0.05, ***p* <0.01, ****p* < 0.001. Source data are provided as a Source data file.

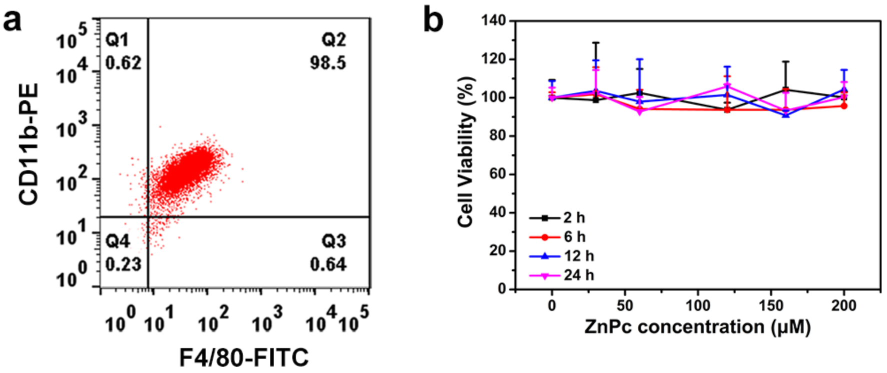

Supplementary Figure 16. (a) FCM analysis of the purity of BMMs cultured in commercial M-CSF doubly stained with F4/80-FITC and CD11b-PE. (b) Cell viability at 2 h, 6 h, 12 h and 24 h via CCK8 assay after incubation with ZnPc@Lip at different platinum concentrations for 2 h (n = 3 technical replicates). All data were presented as mean ± SD.

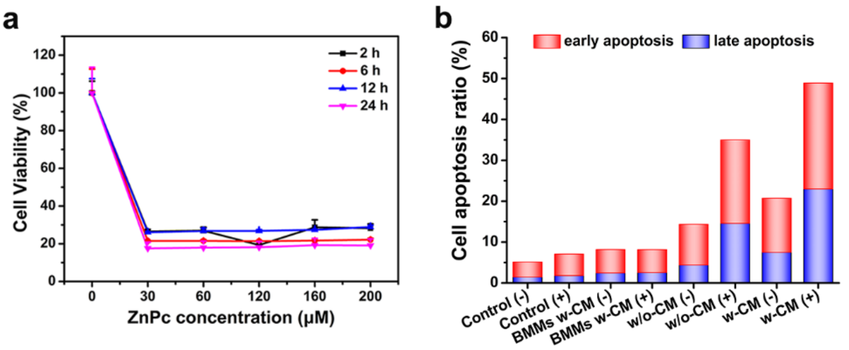

Supplementary Figure 17. (a) Cell viability of BMMs irradiated with LED light (671 nm, 20 mW/cm^2, 10 min) after Oxa(IV)@ZnPc loading, and then recovered for 2 h, 6 h, 12 h and 24 h prior to CCK8 assay (n = 5 technical replicates). (b) Cell apoptosis ratio of 4T1 cells detected by FCM. All data were presented as mean ± SD.

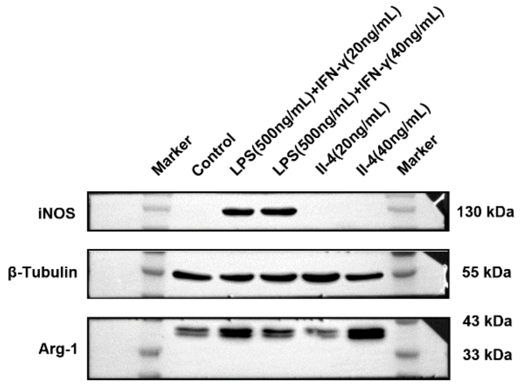

Supplementary Figure 18 Western blot detection of iNOS (M1 maker) and Arg-1(M2 Maker) of BMMs after various treatment for 24 h. The samples were derive from the same experiment and the gels were processed in parallel. This experiment was performed one time.

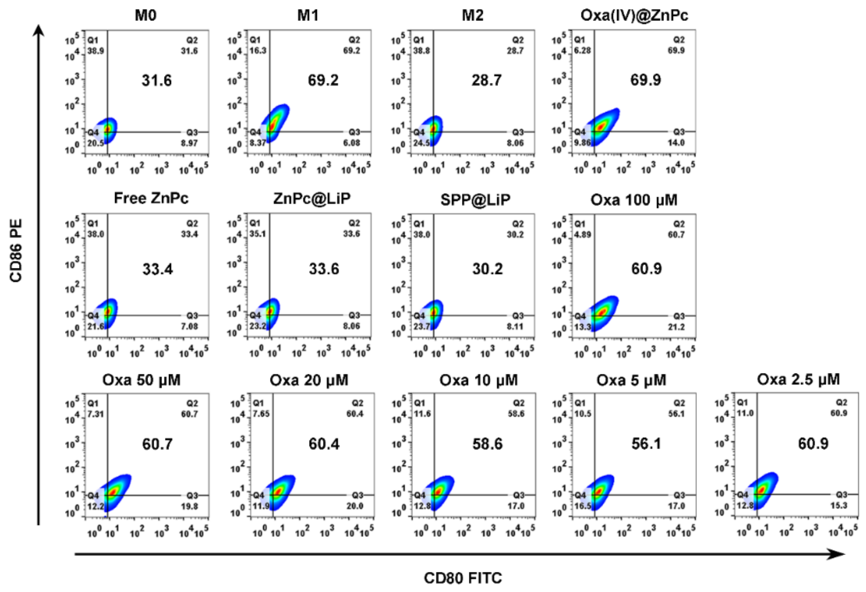

Supplementary Figure 19 Flow cytometric analysis of the expression of M1 marker (CD80, CD86) on BMMs after various treatment.

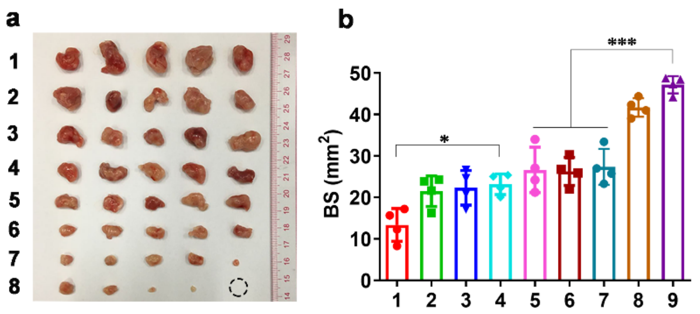

Supplementary Figure 20. Therapeutic efficacy of Oxa(IV)@ZnPc@M combined with anti-PD-L1 for primary and bone metastatic breast cancer in 4T1-bearing mice. (a) Photograph of ex vivo primary tumors of various groups. The architecture parameters of (b) BS (*p = 0.0201, ***p <0.0001) and (c) Tb.N (n = 4 technical replicates based on calculation parameters from one of the representative mouse of each group, **p = 0.0090, ***p <0.0001). 1: PBS (+); 2: free Oxa; 3: Oxa(IV)@ZnPc; 4: Oxa(IV)@ZnPc@M; 5: BMMs + anti-PD-L1; 6: Oxa(IV)@ZnPc (+); 7: Oxa(IV)@ZnPc@M (+); 8: Oxa(IV)@ZnPc@M (+) + anti-PD-L1; 9: Normal, "(+)" represent laser irradiation. All data were presented as mean ± SD. Statistical significance was calculated via ordinary one-way ANOVA with a Tukey's test. *p < 0.05, **p <0.01, ***p < 0.001. Source data are provided as a Source data file.

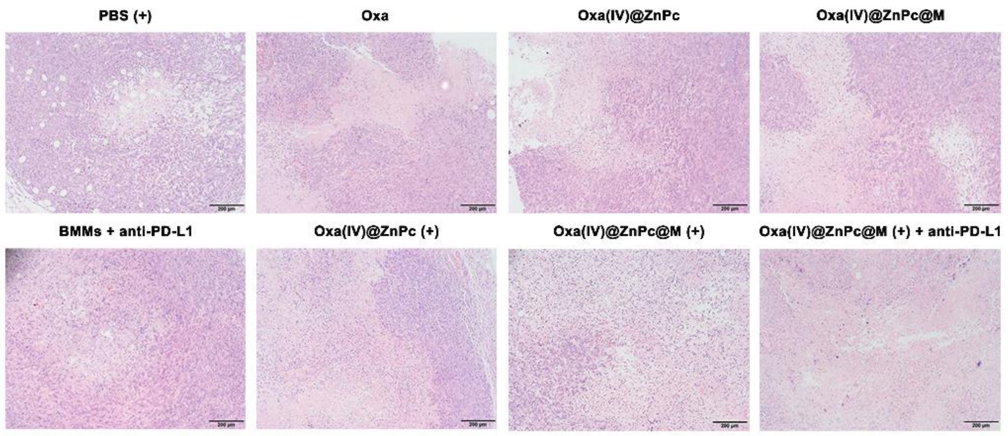

Supplementary Figure 21. H&E staining of primary tumors after various treatments in 4T1-bearing mice, this experiment was performed one time. Scale bars = 200 μm.

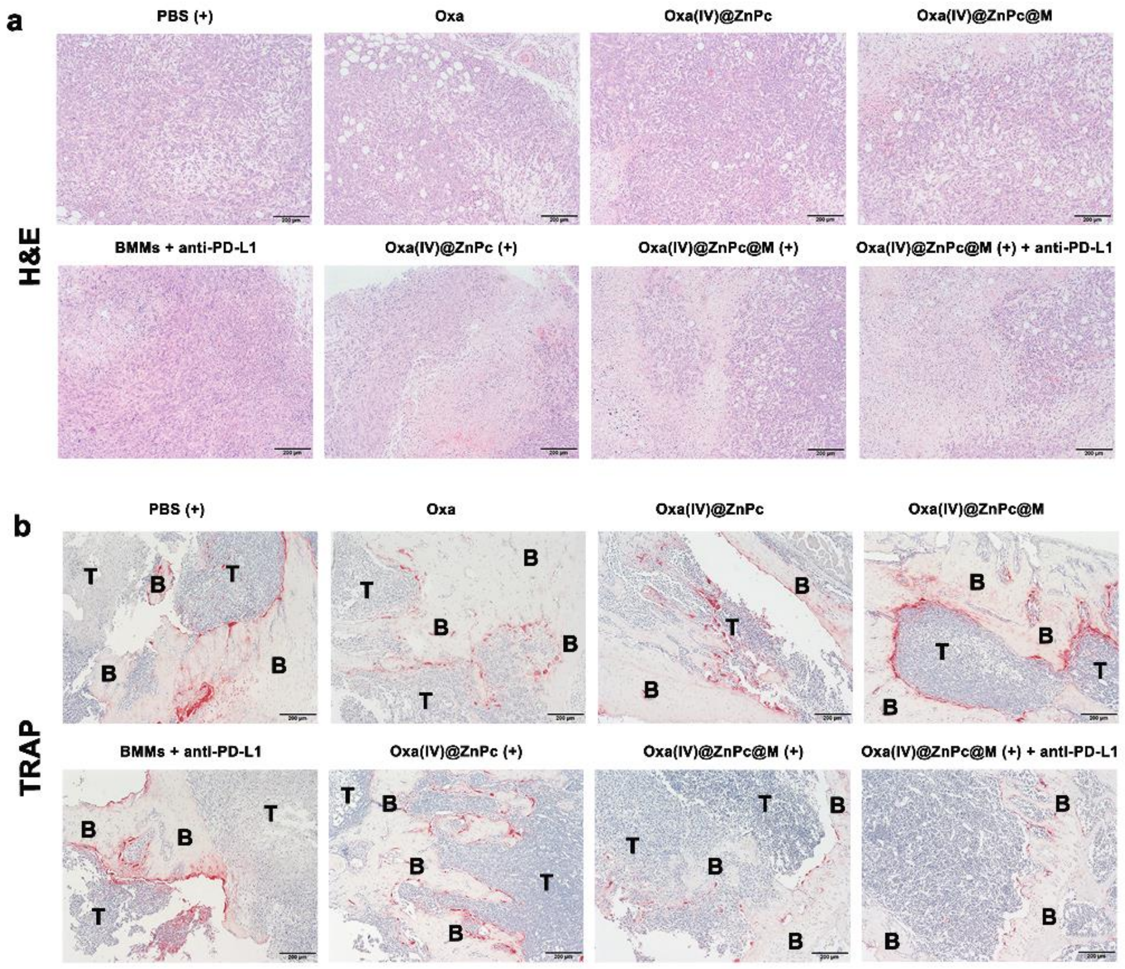

Supplementary Figure 22. (a) H&E staining of bone metastatic tumors after various treatments 4T1-bearing mice. (b) TRAP staining of the tumor-bearing tibias 4T1-bearing mice. These experiments were performed one time. T represents tumor and B represents bone. Scale bars = 200 μm.

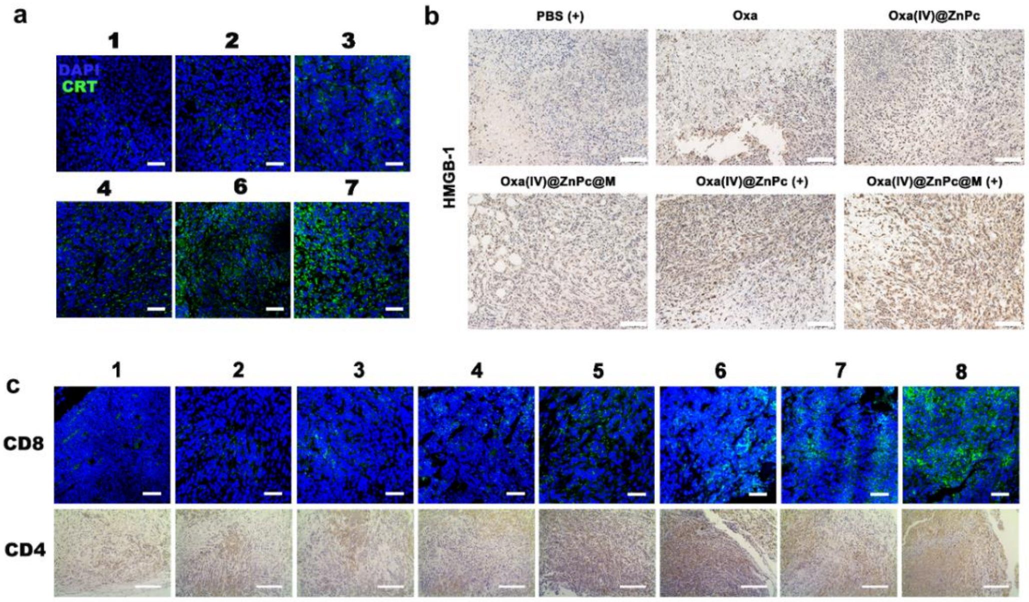

Supplementary Figure 23. The ICD induction and tumor infiltrating of $CD4^+$ and $CD8^+$ T cells in 4T1-tumor bearing mice corresponding to Fig. 6. (a) CLSM observation of CRT exposure in the primary tumor sections. Scale bars = 50 μm. (b) IHC staining of HMGB-1 in primary tumors. Scale bars = 100 μm. (c) Representative IF staining of $CD8^+$T cells (Scale bars = 50 μm), and IHC staining of $CD4^+$T cells (Scale bars = 200 μm) in bone metastatic tumors. (1: PBS (+); 2: free Oxa; 3: Oxa(IV)@ZnPc; 4: Oxa(IV)@ZnPc@M; 5: BMMs + anti-PD-L1; 6: Oxa(IV)@ZnPc (+); 7: Oxa(IV)@ZnPc@M (+); 8: Oxa(IV)@ZnPc@M (+) + anti-PD-L1, "(+)" represent with laser irradiation). These experiments were repeated once.

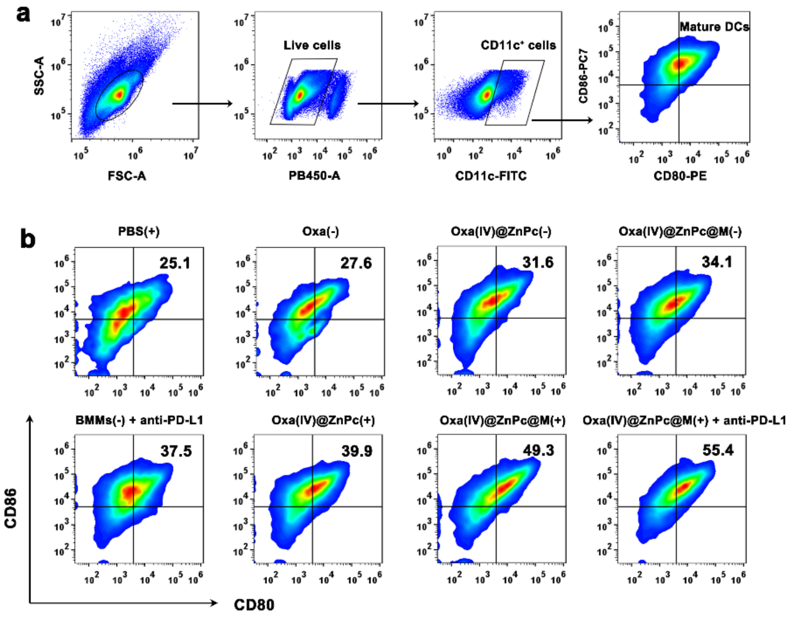

Supplementary Figure 24. (a) The gating strategy and (b) the representative flow cytometric

analysis of DCs maturation in 4T1 tumor-draining lymph nodes on day 2 after various treatment corresponding to Fig. 7b.

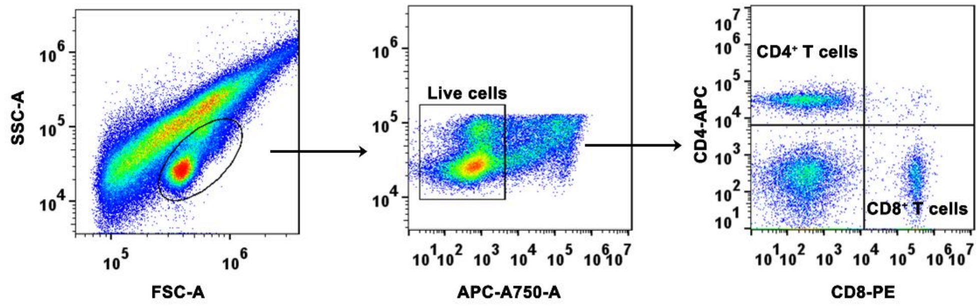

Supplementary Figure 25. The flow cytometric gating strategy of tumor-infiltrating $CD4^+$ T cells and $CD8^+$ T cells in 4T1 model corresponding to Fig. 7c.

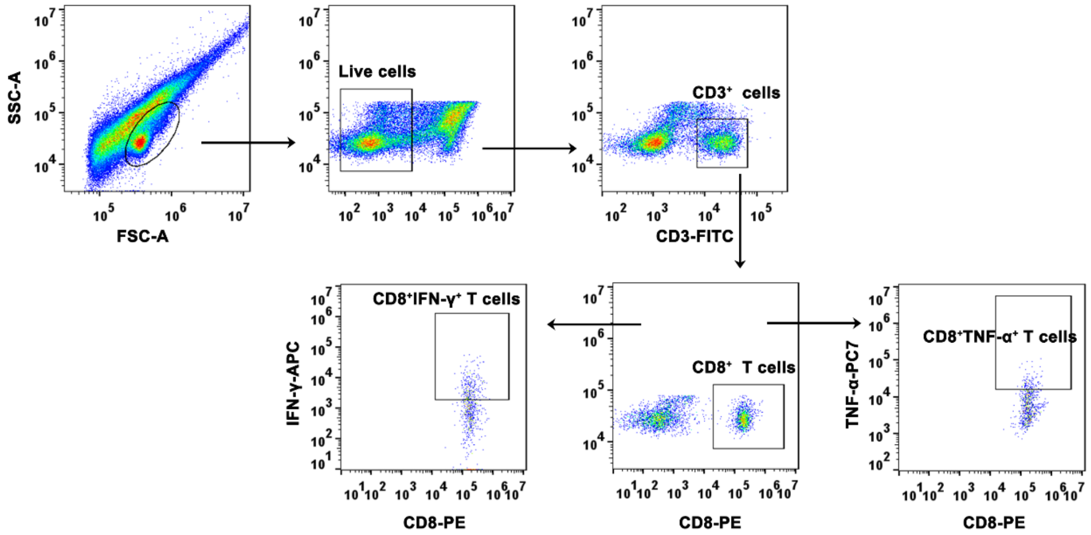

Supplementary Figure 26. The flow cytometric gating strategy of IFN-γ and TNF-α within $CD8^+$ T cells in 4T1 model corresponding to Fig. 7g.

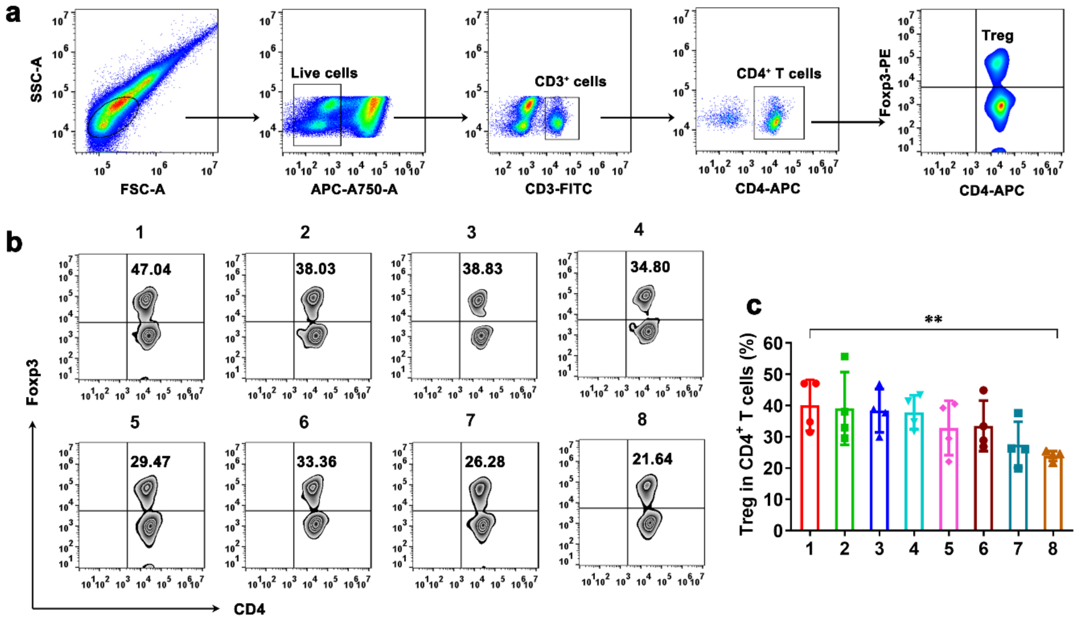

Supplementary Figure 27. (a) The gating strategy and (b) the representative flow cytometric plots of Treg ($CD3^+CD4^+Foxp^{3+}$) in 4T1 model. (c) Quantitation of the percent of Treg in tumors analyzed on day 6 after various treatment (1: PBS (+); 2: free Oxa; 3: Oxa(IV)@ZnPc; 4: Oxa(IV)@ZnPc@M; 5: BMMs + anti-PD-L1; 6: Oxa(IV)@ZnPc (+); 7: Oxa(IV)@ZnPc@M (+); 8: Oxa(IV)@ZnPc@M (+) + anti-PD-L1, "(+)" represent with laser irradiation) (**p = 0.0079). All data were presented as mean ± SD (n = 4 individual animals). Statistical significance was calculated via the unpaired two-tailed t test. **p <0.01.

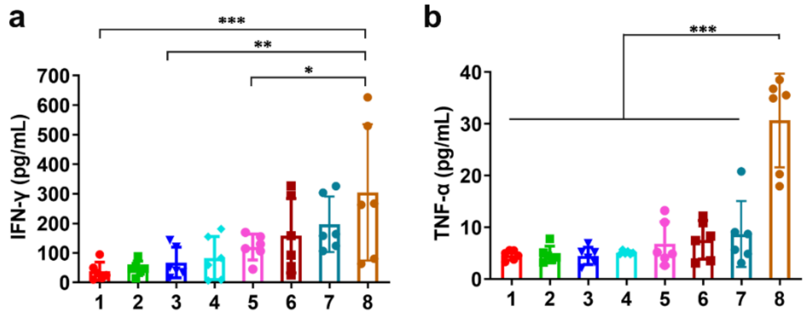

Supplementary Figure 28. Cytokine levels of (a) IFN-γ (***p = 0.0006, **p = 0.0023, *p = 0.0242) and (b) TNF-α (***p < 0.0001) in serum from 4T1 tumor-bearing mice harvested on day 6 after various treatment (1: PBS (+); 2: free Oxa; 3: Oxa(IV)@ZnPc; 4: Oxa(IV)@ZnPc@M; 5: BMMs + anti-PD-L1; 6: Oxa(IV)@ZnPc (+); 7: Oxa(IV)@ZnPc@M (+); 8: Oxa(IV)@ZnPc@M (+) + anti-PD-L1, "(+)" represent with laser irradiation). All data were presented as mean ± SD (n = 6 individual animals). Statistical significance was calculated via ordinary one-way ANOVA with a Dunnett's test. *p <0.05, **p <0.01, ***p < 0.001. Source data are provided as a Source data file.

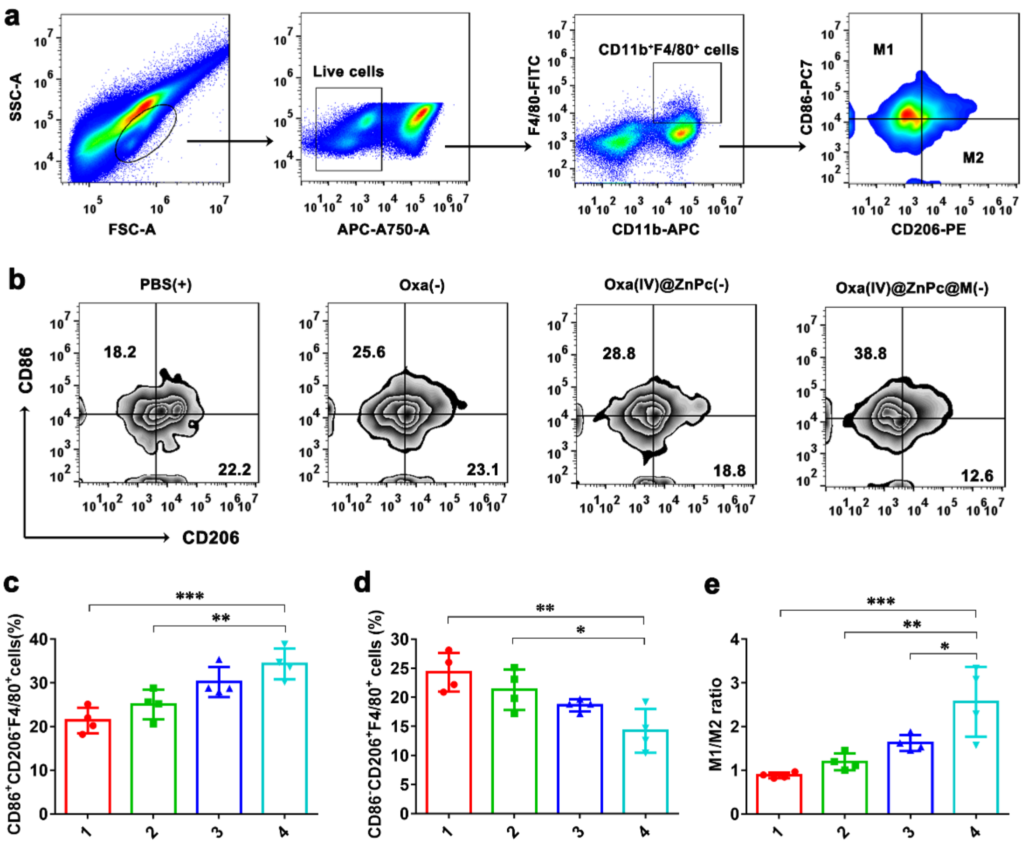

Supplementary Figure 29. (a) The gating strategy and (b) the representative flow cytometric plots of M1 macrophages ($CD86^{+}CD206^{-}F4/80^{+}$) and M2 macrophages ($CD86^{-}CD206^{+}F4/80^{+}$) in 4T1 model. Quantitation of the percent of (c) M1 macrophages (****p* = 0.0007, ***p* = 0.0092) and (d) M2 macrophages (***p* = 0.0030, **p* = 0.0321) analyzed on day 6 after various treatment (1: PBS (+); 2: free Oxa; 3: Oxa(IV)@ZnPc; 4: Oxa(IV)@ZnPc@M). (e) Quantitation of M1 macrophages to M2 macrophages (****p* = 0.0005, ***p* = 0.0029, **p* = 0.0356). All data were presented as mean ± SD (n = 4 individual animals). Statistical significance was calculated via ordinary one-way ANOVA with a Tukey's test. **p* < 0.05, ***p* < 0.01, ****p* < 0.001. Source data are provided as a Source data file.

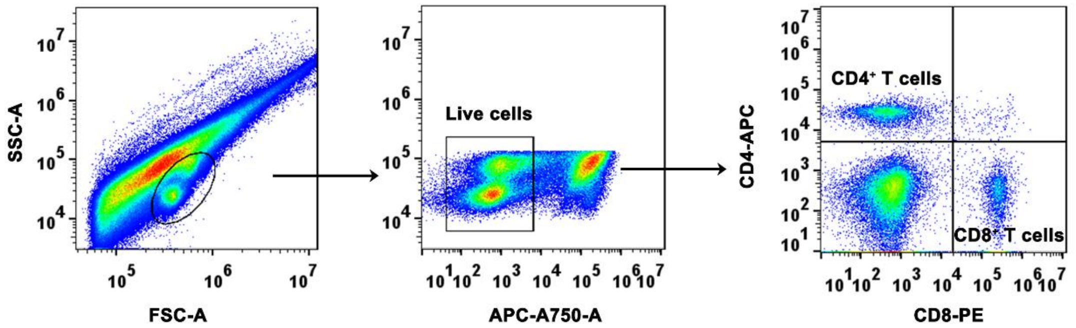

Supplementary Figure 30. The flow cytometric gating strategy of tumor-infiltrating CD4$^+$ T cells and CD8$^+$ T cells in EMT6 model corresponding to Fig. 9h.

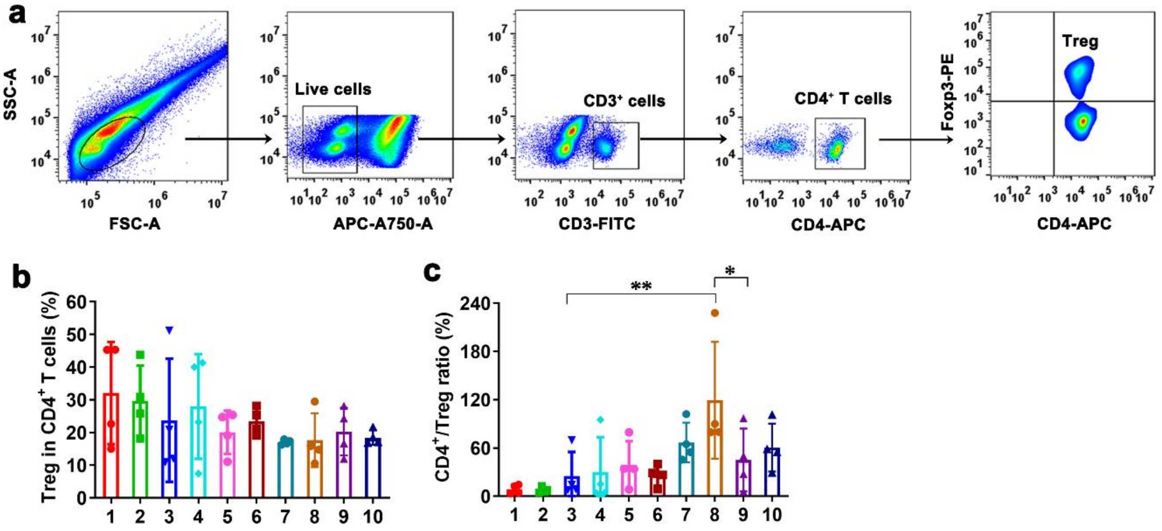

Supplementary Figure 31. (a) The gating strategy of Treg (CD3$^+$CD4$^+$Foxp^{3+}) in EMT6 model. (b) Quantitation of the percent of Treg in tumors analyzed on day 6 after various treatment (1: PBS (+); 2: free Oxa; 3: Oxa(IV)@ZnPc; 4: Oxa(IV)@ZnPc@M; 5: BMMs + anti-PD-L1; 6: Oxa(IV)@ZnPc (+); 7: Oxa(IV)@ZnPc@M (+); 8: Oxa(IV)@ZnPc@M (+) + anti-PD-L1; 9: Oxa + anti-PD-L1; 10: Oxa(IV)@ZnPc (+) + anti-PD-L1, "(+)" represent laser irradiation). (c) Quantitation of the percent of intratumoral ratio of CD4$^+$ T cells to Treg (**p = 0.0048, *p = 0.0356). Statistical significance was calculated via ordinary one-way ANOVA with a Dunnett's test. All data were presented as mean ± SD (n = 4 individual animals). *p <0.05, **p <0.01. Source data are provided as a Source data file.

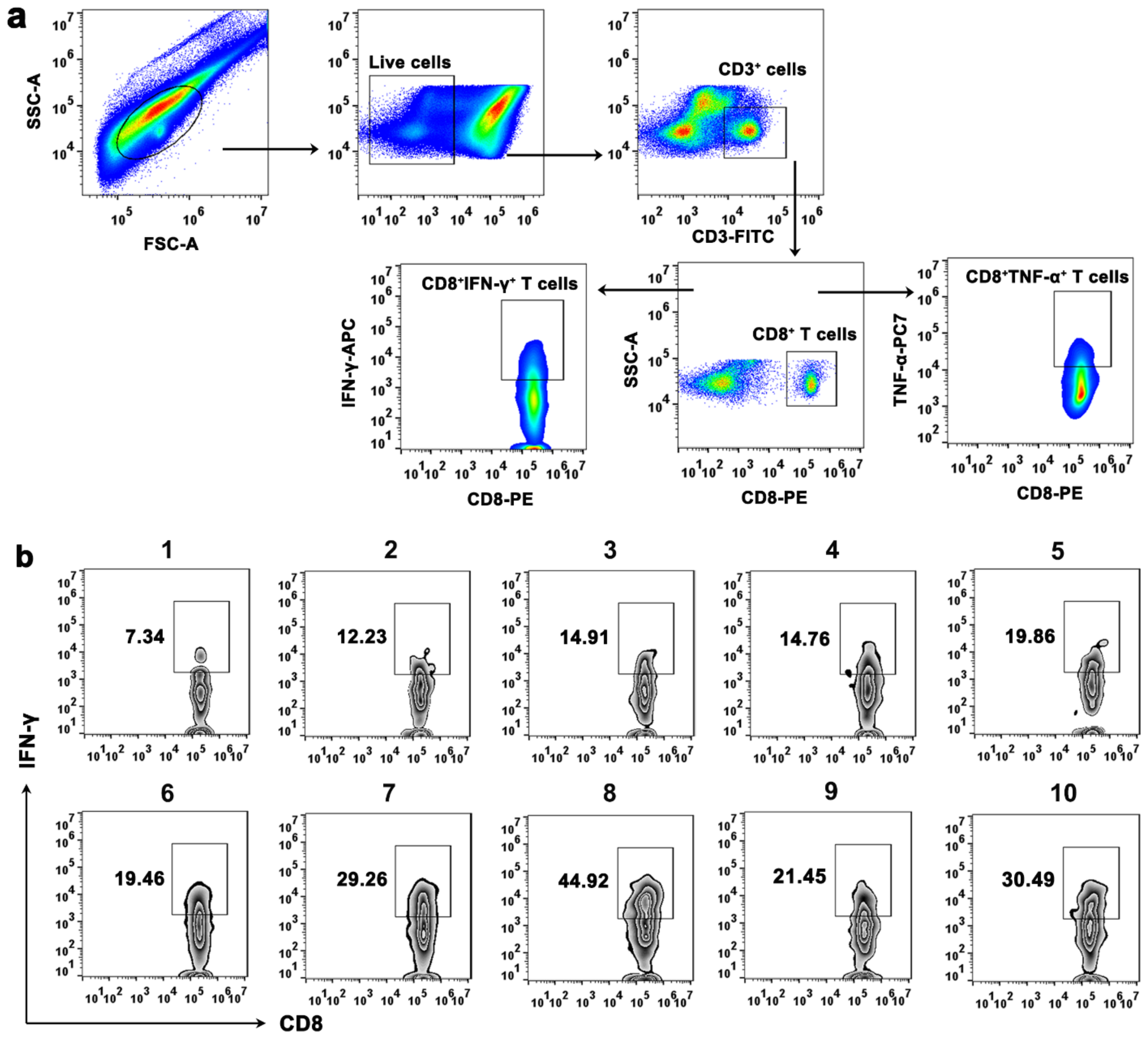

Supplementary Figure 32. (a) The flow cytometric gating strategy in EMT6 model. (b) The representative flow cytometric plots of IFN-γ within CD8$^+$ T cells in EMT6 model after various treatment (1: PBS (+); 2: free Oxa; 3: Oxa(IV)@ZnPc; 4: Oxa(IV)@ZnPc@M; 5: BMMs + anti-PD-L1; 6: Oxa(IV)@ZnPc (+); 7: Oxa(IV)@ZnPc@M (+); 8: Oxa(IV)@ZnPc@M (+) + anti-PD-L1; 9: Oxa + anti-PD-L1; 10: Oxa(IV)@ZnPc (+) + anti-PD-L1, "(+)" represent laser irradiation).

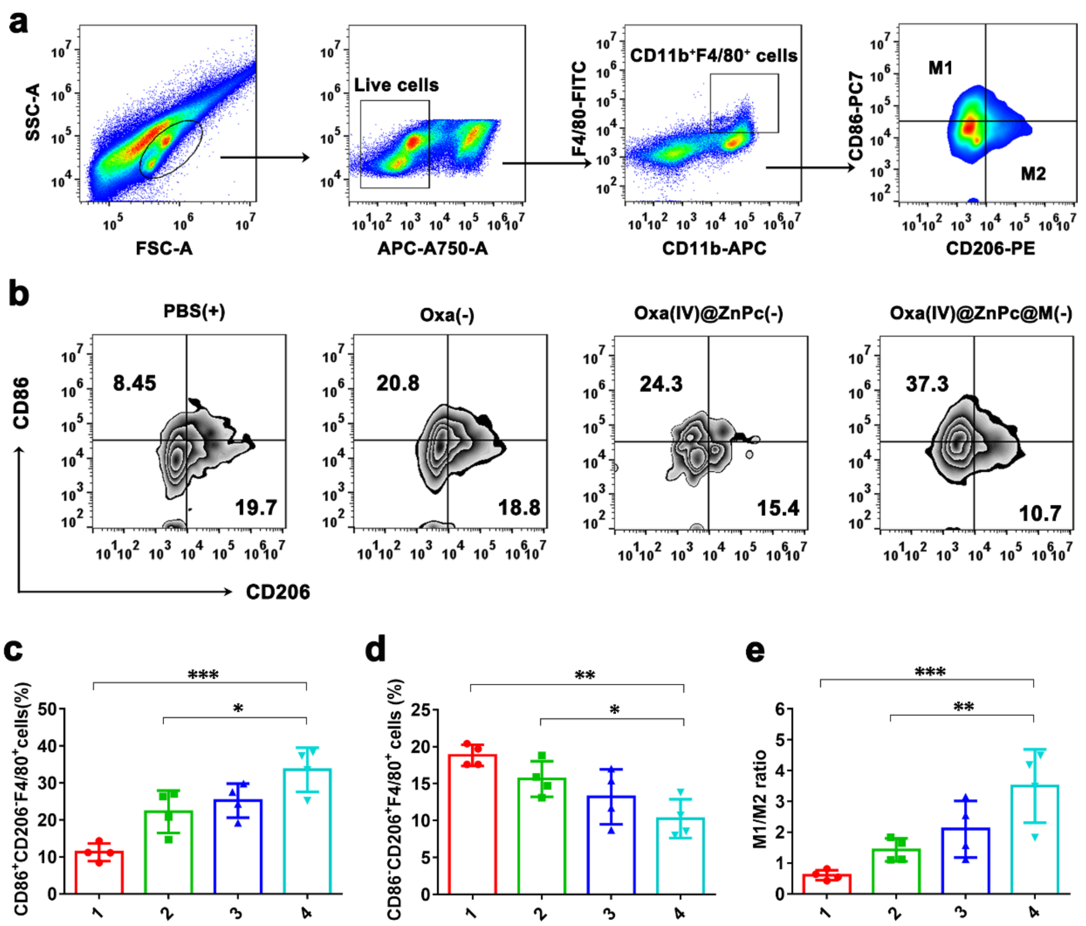

Supplementary Figure 33. (a) The gating strategy and (b) the representative flow cytometric plots of M1 macrophages ($CD86^{+}CD206^{-}F4/80^{+}$) and M2 macrophages ($CD86^{-}CD206^{+}F4/80^{+}$) in EMT6 model. Quantitation of the percent of (c) M1 macrophages (****p* < 0.0001, **p* = 0.0164) and (d) M2 macrophages (***p* = 0.0019, **p* = 0.0082) analyzed on day 6 after various treatment (1: PBS (+); 2: free Oxa; 3: Oxa(IV)@ZnPc; 4: Oxa(IV)@ZnPc@M). (e) Quantitation of M1 macrophages to M2 macrophages (****p* = 0.0006, ***p* = 0.0073). All data were presented as mean ± SD (n = 4 individual animals). Statistical significance was calculated via ordinary one-way ANOVA with a Dunnett's test. **p* < 0.05, ***p* < 0.01, ****p* < 0.001 Source data are provided as a Source data file.

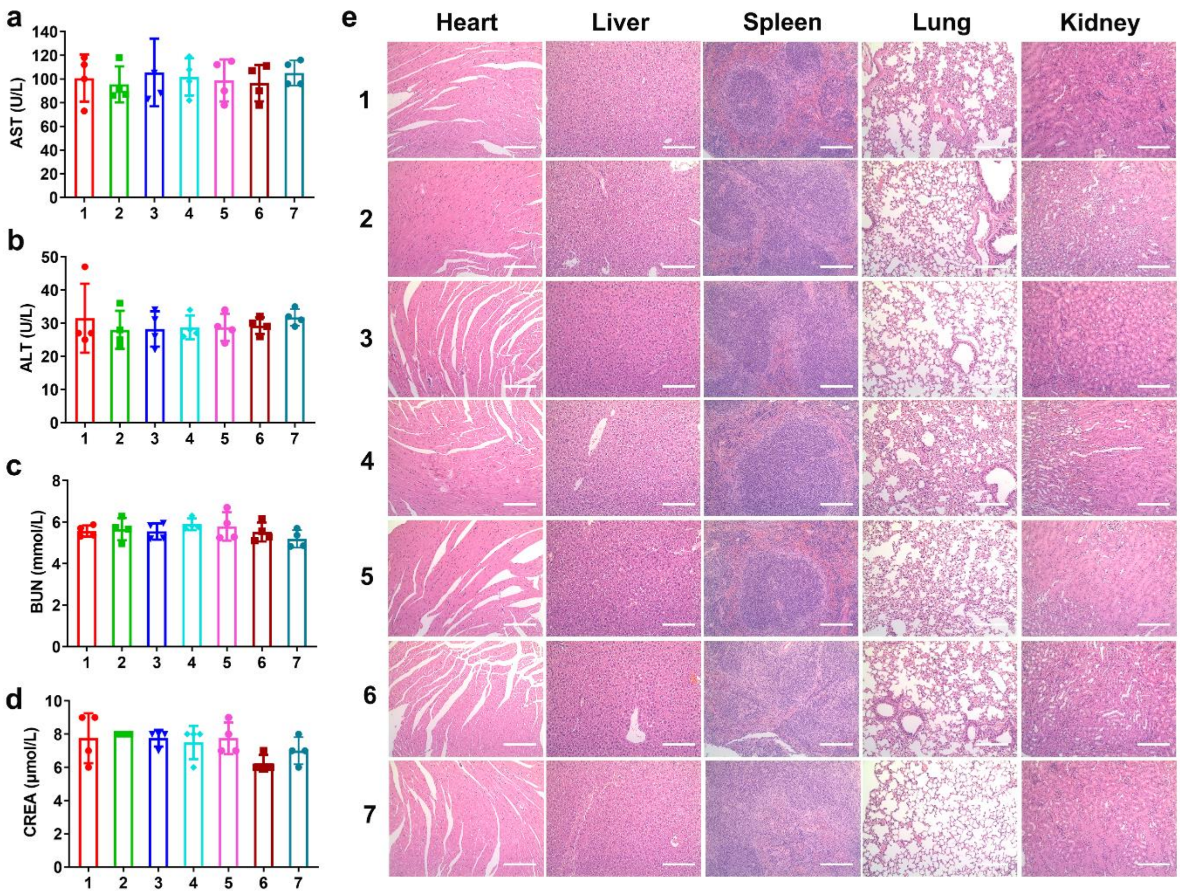

Supplementary Figure 34. Toxicity studies. Plasma levels of (a) AST, (b) ALT, (c) BUN and (d) CREA after single-dose injection of different drug formulations (1: PBS; 2: free Oxa; 3: Oxa(IV)@ZnPc; 4: Oxa(IV)@ZnPc@M; 5: BMMs; 6: Oxa(IV)@ZnPc@M + anti-PD-L1; 7: BMMs + anti-PD-L1) (n = 4 individual animals). (e) H&E staining of major organs after single-dose injection, This experiment was performed two times with similar results. Scale bars = 200 μm. All data were presented as mean ± SD. Source data are provided as a Source data file.

Supplementary Tables

Supplementary Table 1. Pharmacokinetics parameter AUC and $t_{1/2}$ in SD rats after intravenous injection (n=3).

	Oxa	Oxa(IV)@ZnPc	Oxa(IV)@ZnPc@M
AUC (mg/L*h)	6.71 ± 4.31	10.02 ± 1.80	175.59 ± 18.04
$t_{1/2}$ (h)	19.45 ± 0.26	19.45 ± 0.26	70.97 ± 11.43

Supplementary Table 2. Complete Blood Count Report of single-dose injection (n=4 individual animals)

	WBC [10^9/L]	RBC [10^{12}/L]	HGB [g/L]	HCT [%]	MCV [fL]	MCH [pg]	MCHC [g/L]	PLT [10^9/L]
PBS	4.67±0.48	8.96±0.33	149.40±4.88	40.33±1.26	44.98±0.42	16.88±0.23	374.67±4.08	819.00±87.63
Free Oxa(II)	4.50±0.52	8.91±0.44	145.60±14.27	39.90±1.45	44.82±0.95	16.36±1.44	364.80±32.34	823.00±61.29
Oxa(IV)@ZnPc	4.57±0.62	8.79±0.58	149.25±9.43	39.67±2.38	45.15±0.71	16.97±0.42	376.25±4.57	785.67±55.59
Oxa(IV)@ZnPc@M	4.34±0.25	8.35±0.19	142.20±1.48	37.88±0.70	45.36±0.54	17.02±0.27	375.40±3.43	788.00±23.31
BMMs	3.77±0.95	7.88±0.52	131.80±15.61	35.30±1.80	45.30±0.90	16.72±0.38	371.50±13.53	726.67±143.6
Oxa(IV)@ZnPc@M+anti PD-L1	3.82±1.46	8.36±0.76	139.86±13.47	37.97±3.37	45.43±0.39	16.71±0.77	373.83±10.09	811.67±53.88
BMMs+anti PD-L1	3.16±0.37	8.34±0.57	141.60±5.94	37.72±2.38	45.22±0.41	17.02±0.86	375.80±16.87	774.4±115.80

Supplementary Table 3. Complete Blood Count Report of multi-dose injection (n=4 individual animals)

	WBC [10^9/L]	RBC [10^{12}/L]	HGB [g/L]	HCT [%]	MCV [fL]	MCH [pg]	MCHC [g/L]	PLT [10^9/L]
PBS	3.36±1.12	8.28±0.28	142.71±4.99	38.2±1.16	46.13±0.52	17.21±0.21	373.43±4.54	799.28±80.99
Free Oxa(II)	2.78±0.46	8.12±0.26	141.00±5.10	37.90±1.07	46.65±0.24	17.32±0.27	372.00±6.68	864.00±26.87
Oxa(IV)@ZnPc	2.99±0.69	8.49±0.31	147.75±7.80	39.95±1.83	46.12±0.23	17.05±0.19	369.75±3.30	831.33±45.00
Oxa(IV)@ZnPc@M	2.64±0.76	8.19±0.35	141.60±6.54	38.44±1.31	46.94±0.49	17.28±0.22	368.20±3.43	760.00±99.82
BMMs	3.42±0.39	8.16±0.37	141.75±2.99	37.52±1.17	46.62±1.69	17.40±0.60	377.75±6.83	762.25±71.89
Oxa(IV)@ZnPc@M+anti PD-L1	2.63±0.58	8.13±0.64	143.20±11.19	37.58±2.20	46.26±0.94	17.62±0.30	380.80±9.73	721.33±99.30
BMMs+anti PD-L1	2.75±0.72	8.24±0.64	145.75±11.21	39.10±2.84	46.44±0.76	17.64±0.15	380.20±5.80	774.80±52.30
