# Supplementary material for: Engineered macrophages as near-infrared light activated drug vectors for chemo-photodynamic therapy of primary and bone metastatic breast cancer
Source: Nat Commun. 2021 Jul 14;12:4310. doi: 10.1038/s41467-021-24564-0 (PMC8280231; doi:10.1038/s41467-021-24564-0)
Supplement: Supplementary file 2 — Reporting Summary [file 41467_2021_24564_MOESM2_ESM.pdf]

nature research

Corresponding author(s): Chunshun Zhao

Last updated by author(s): Jun 7, 2021

Reporting Summary

Nature Research wishes to improve the reproducibility of the work that we publish. This form provides structure for consistency and transparency in reporting. For further information on Nature Research policies, see our Editorial Policies and the Editorial Policy Checklist.

Statistics

For all statistical analyses, confirm that the following items are present in the figure legend, table legend, main text, or Methods section.

n/a	Confirmed	
☐	☒	The exact sample size (*n*) for each experimental group/condition, given as a discrete number and unit of measurement
☐	☒	A statement on whether measurements were taken from distinct samples or whether the same sample was measured repeatedly
☐	☒	The statistical test(s) used AND whether they are one- or two-sided<br>*Only common tests should be described solely by name; describe more complex techniques in the Methods section.*
☒	☐	A description of all covariates tested
☐	☒	A description of any assumptions or corrections, such as tests of normality and adjustment for multiple comparisons
☐	☒	A full description of the statistical parameters including central tendency (e.g. means) or other basic estimates (e.g. regression coefficient) AND variation (e.g. standard deviation) or associated estimates of uncertainty (e.g. confidence intervals)
☐	☒	For null hypothesis testing, the test statistic (e.g. *F*, *t*, *r*) with confidence intervals, effect sizes, degrees of freedom and *P* value noted<br>*Give P values as exact values whenever suitable.*
☒	☐	For Bayesian analysis, information on the choice of priors and Markov chain Monte Carlo settings
☒	☐	For hierarchical and complex designs, identification of the appropriate level for tests and full reporting of outcomes
☒	☐	Estimates of effect sizes (e.g. Cohen's *d*, Pearson's *r*), indicating how they were calculated

Our web collection on statistics for biologists contains articles on many of the points above.

Software and code

Policy information about availability of computer code

Data collection	Particle size and zeta potential were performed on Malvern Zetasizer Nano ZS90 (Malvern Instruments, Malvern, UK). Transmission electron microscopy images were acquire on JEM-1400, (JEOL, Japan). Fourier transform infrared spectroscopy (FTIR) spectrum was recorded on VERTEX 70 (Bruker, Germany). X-ray photoelectron spectroscopy (XPS) spectrum was recorded by ESCALAB 250 (Thermo Fisher Scientific). Platinum content was detected by Graphite Furnace Atomic Absorption Spectrometer (GFAAS, AAnalyst 800, Perkin–Elmer, USA). Fluorescence spectrum and fluorescence detection was performed on Fluorescence spectrometer (Fluoromax-4, HORIBA, USA). Flow cytometry (CytoFLEX-S, Beckman Coulter) was used to acquire flow cytometry data in vivo. Flow cytometry (Guava EasyCyte 6-2L, Merck Millpore) was used to acquire flow cytometry data in vitro. Immunofluorescent staining was performed onconfocal laser scanning microscopy (LSM 710, Zeiss, Germany). In vivo fluorescence imaging was conducted on a in vivo imaging system (Lumina XRMS Series III, PerkinElmer, USA). The degree of osteolysis were analyzed by a Biograph 3D micro-CT device (ZKKS-MCT-Sharp-I, Caskaisheng, China).
Data analysis	Date analysis was performed using GraphPad Prism 8.3.0, Flow cytometry data was analyzed by FlowJo X10.0.7r2 and CytExpert 2.3.1. The 3D Micro-CT images were reconstructed and analyzed using the ZKKS-Micro CT analysis software 4.1 .

For manuscripts utilizing custom algorithms or software that are central to the research but not yet described in published literature, software must be made available to editors and reviewers. We strongly encourage code deposition in a community repository (e.g. GitHub). See the Nature Research guidelines for submitting code & software for further information.

Data

Policy information about availability of data

All manuscripts must include a data availability statement. This statement should provide the following information, where applicable:

- Accession codes, unique identifiers, or web links for publicly available datasets
- A list of figures that have associated raw data
- A description of any restrictions on data availability

The data that support the findings of this study are available within this article and its Supplementary Information or from the corresponding author upon reasonable request. A Source Data file, including Figures 2a, 2d, 2f, 2g, 2h-i, 3b-c, 3e, 3f-g, 3i, 4b, 4e, 4f-i, 4j-l, 4n, 5a, 5d-f, 6b, 6f-5g, 7b-j, 8b-d, 8e, 8f, 8g, 9b-e, 9g-l, 10a-d, and Supplementary Figures 9a-d, 10a-b, 11a-b, 11d-f, 11g-i, 14a-b, 15, 16b, 17a-b, 18, 20, 27-29, 31 and 33-34, have been provided.

Field-specific reporting

Please select the one below that is the best fit for your research. If you are not sure, read the appropriate sections before making your selection.

[x] Life sciences [ ] Behavioural & social sciences [ ] Ecological, evolutionary & environmental sciences

For a reference copy of the document with all sections, see nature.com/documents/nr-reporting-summary-flat.pdf

Life sciences study design

All studies must disclose on these points even when the disclosure is negative.

Sample size	Simple sizes of 3-8 biologically independent samples or animals per group were used for in vitro and in vitro studies, respectively, as indicated for specific experiments in Figure captions. We adhered to sample size requirements necessary for determining statistical significance with reference to the numbers used in recent relevant publications.
Data exclusions	No data were excluded from the analyses.
Replication	All key experiments were successfully replicated at least once, as indicated in figure legends.
Randomization	The samples and animals used in this paper were randomly distributed into several groups for the further experiments.
Blinding	We were blinded to group allocation during data collection and analysis for both in vitro and in vivo experiments.

Reporting for specific materials, systems and methods

We require information from authors about some types of materials, experimental systems and methods used in many studies. Here, indicate whether each material, system or method listed is relevant to your study. If you are not sure if a list item applies to your research, read the appropriate section before selecting a response.

Materials & experimental systems

n/a	Involved in the study	
[ ]	[x]	Antibodies
[ ]	[x]	Eukaryotic cell lines
[x]	[ ]	Palaeontology and archaeology
[ ]	[x]	Animals and other organisms
[x]	[ ]	Human research participants
[x]	[ ]	Clinical data
[x]	[ ]	Dual use research of concern

Methods

n/a	Involved in the study	
[x]	[ ]	ChIP-seq
[ ]	[x]	Flow cytometry
[x]	[ ]	MRI-based neuroimaging

Antibodies

Antibodies used	Anti-CD3e-FITC[145-2C11](Cat# 11-0031-81, diluted 1:100 with 4% FBS), anti-CD8α-PE[53-6.7](Cat# 12-0081-82, diluted 1:80 with 4% FBS), anti-CD4-APC[RM4-5](Cat# 17-0042-82, diluted 1:160 with 4% FBS), anti-Foxp3-PE[NRRF-30](Cat# 12-4771-80, diluted 1:80 with 4% FBS), anti-IFN-γ-APC[XMG1.2](Cat# 17-7311-81, diluted 1:160 with 4% FBS), anti-TNF-α-PC7[MP6-XT22](Cat# 25-7321-80, diluted 1:160 with 4% FBS), anti-F4/80-FITC[BM8](Cat# 11-4801-81, diluted 1:50 with 4% FBS), anti-CD11b-APC[M1/70](Cat# 17-0112-81, diluted 1:160 with 4% FBS), anti-CD86(B7-2)-PC7[GL1](Cat# 25-0862-80, diluted 1:80 with 4% FBS), anti-CD206-PE[MR6F3](Cat# 12-2061-82, diluted 1:160 with 4% FBS), anti-CD11c-FITC[N418](Cat# 11-0114-81, diluted 1:200 with 4% FBS), anti-CD80(B7-1)-PE [16-10A1](Cat# 12-0801-81, diluted 3:1000 with 4% FBS), anti-CD16/32[93](Cat# 14-0161-81, diluted 1:100 with 4% FBS) antibodies, and Fixable Viability Dye eFluorTM 450 (Cat# 65-0863, diluted 1:1000 with PBS) and eFluorTM 780 (Cat# 65-0865, diluted 1:1000 with PBS) were purchased from eBiosciences (Hatfield, UK). Anti-rabbit calreticulin polyclonal antibody (Cat# 27298-1-AP, diluted 1:250 with 5%BSA), anti-rabbit HMGB1 antibody (Cat# 10829-1-AP, diluted 1:100 with 5%BSA) and anti-rabbit Alexa Fluor® 488

	secondary antibody (Cat# srbAF488-1, diluted 1:100) were purchased from Proteintech Group (USA). InVivoPlus anti-mouse PD-L1 (B7-H1) antibody (α-PD-L1, Clone: 10 F.9G2, Cat# BP0101) was purchased from BioXcell (USA).
Validation	All antibodies were verified by the supplier and each lot has been quality tested. No further validation was performed. Suppiler validation included quality testing by immunofluorescent staining with flow cytometric analysis on samples cells. For examples, see: https://www.thermofisher.com/cn/zh/antibody/product/CD3e-Antibody-clone-145-2C11-Monoclonal/11-0031-81. https://www.thermofisher.com/cn/zh/antibody/product/CD8a-Antibody-clone-53-6-7-Monoclonal/12-0081-82. https://www.thermofisher.com/cn/zh/antibody/product/CD4-Antibody-clone-RM4-5-Monoclonal/17-0042-82. https://www.thermofisher.com/cn/zh/antibody/product/FOXP3-Antibody-clone-NRRF-30-Monoclonal/12-4771-80. https://www.thermofisher.com/cn/zh/antibody/product/IFN-gamma-Antibody-clone-XMG1-2-Monoclonal/17-7311-81. https://www.thermofisher.com/cn/zh/antibody/product/TNF-alpha-Antibody-clone-MP6-XT22-Monoclonal/25-7321-80

Eukaryotic cell lines

Policy information about cell lines

Cell line source(s)	4T1 cells and L929 cells were purchased from the Laboratory Animal Center of Sun Yat-sen University (Guangzhou, China). Luc-4T1 cells (FH1114) stably expressing high levels of luciferase and EMT6 cells were purchased from FuHeng Cell Cente (Shanghai, China).
Authentication	Cells were authenticated by short tandem repeat fingerprinting.
Mycoplasma contamination	All cells were tested negative for mycoplasma contamination.
Commonly misidentified lines (See ICLAC register)	No misidentified lines.

Animals and other organisms

Policy information about studies involving animals; ARRIVE guidelines recommended for reporting animal research

Laboratory animals	Healthy Balb/c female mice (5-6 weeks), C57BL/6 female mice (6-8 weeks) and Sprague-Dawley male rats (SD, 180-200 g) were obtained from the Laboratory Animal Center of Sun Yat-sen University (Guangzhou, China). The animals were hosted in an SPF barrier environment with temperature at 20–26 °C and humidity at 40%–70%, under the 12h/12h dark/light cycle.
Wild animals	No wild animals used.
Field-collected samples	No Field-collected samples used.
Ethics oversight	All animal experiments were conducted under protocols approved by the Institutional Animal Care and Use Committee of Sun Yat-sen University.

Note that full information on the approval of the study protocol must also be provided in the manuscript.

Flow Cytometry

Plots

Confirm that:

- [x] The axis labels state the marker and fluorochrome used (e.g. CD4-FITC).
- [x] The axis scales are clearly visible. Include numbers along axes only for bottom left plot of group (a 'group' is an analysis of identical markers).
- [x] All plots are contour plots with outliers or pseudocolor plots.
- [x] A numerical value for number of cells or percentage (with statistics) is provided.

Methodology

Sample preparation	Tumors were harvested, cut into small pieces and ground up, then passed through 200 μm and 70 μm filter to obtain single-cell suspensions. All the cells were treated with ACK Lysis Buffer, washed, and then collected by centrifugation.
Instrument	Flow cytometry (CytoFLEX-S, Beckman Coulter)
Software	FlowJo X10.0.7r2 and CytExpert 2.3.1.
Cell population abundance	FACS analysis was performed on each sample to a total cell number for at least 1,000,00 events.
Gating strategy	Cells were first gated by FSC/SSC, and then gated for live cells using eFluorTM 450 or 780. The detailed gating strategy were seen in Supplementary Figures 24-28 and 30-33.

- [x] Tick this box to confirm that a figure exemplifying the gating strategy is provided in the Supplementary Information.
