# Supplementary material for: Engineered macrophages as near-infrared light activated drug vectors for chemo-photodynamic therapy of primary and bone metastatic breast cancer
Source: Nat Commun. 2021 Jul 14;12:4310. doi: 10.1038/s41467-021-24564-0 (PMC8280231; doi:10.1038/s41467-021-24564-0)
Supplement: Supplementary file 4 — Peer Review File [file 41467_2021_24564_MOESM4_ESM.pdf]

Editorial note: Please note that statistical analyses have been repeated by the authors with a different software, thus minor differences could be present between the figures in the peer review file and in the final version of the manuscript.

REVIEWER COMMENTS

Reviewer #1 (Remarks to the Author): with expertise in chemo/photodynamic therapy

Huang et al. develop a macrophage-based NIR-responsive drug-delivering system, and present a detailed in vitro and in vivo assessment of their application in synergistic chemo/photodynamic/immune treatment of primary and bone metastatic tumors. The experiments are well-performed for the most part, and some results are interesting. However, the novelty of the paper is suggested to improve, because light-controlled cell carriers have been well developed in literature (Adv. Funct. Mater. 2015, 35, 2386; ACS Appl. Mater. Interfaces 2017, 9, 5855).

1. The major novelty of the present work may be the development of light-controlled macrophages as a drug carrier for synergistic cancer therapy. What are the advantages of macrophages as a drug carrier compared to other kinds of cells? Macrophages can be converted into the M1 phenotypes that can inhibit the tumor, but unfortunately, most macrophages were killed after light irradiation. The authors are suggested to give more evidence to present the merits of this strategy. 2. Delivering therapeutic agents to both primary and EPR-deficient bone-metastatic nodules may be an advantage of macrophages over nanocarriers (Line 73-78, 98-100). However, as shown in Figure 4c,e, more Oxa(IV)@ZnPc (without macrophages) was detected in the bone tumor than in the primary tumor. After loading into macrophages, the contents of the formulation in the bone tumor and the primary tumor were similar. What is the reason? A better EPR-deficient model is recommended.

3. The mechanism of the drug (Oxa and ZnPc) release from Oxa(IV)@ZnPc should be further investigated. Besides, for Oxa(IV)@ZnPc@M without irradiation, is the drug (Oxa and ZnPc) release from the system due to their exocytosis from the macrophages? Or the drug was first released from Oxa(IV)@ZnPc and then from macrophages.

4. Nanoparticles Oxa(IV)@ZnPc (without macrophages) can be captured by the reticuloendothelial system including macrophages, and may form a similar system with the present Oxa(IV)@ZnPc@M. What are the differences between the two systems?

5. In vitro stability of Oxa(IV)@ZnPc was evaluated within only 24 h (Figure S10d). The long-term stability of the nanoparticles should be evaluated.

6. Cal@DiI was used to assess the integrity of the core-shell nanoparticles after entering the cells. Why not use Cal@ZnPc as the model?

7. Oxa(IV)@ZnPc makes the polarization of macrophages to the M1 phenotype. Is it attributed to the released drugs (Oxa and ZnPc), the nanocarriers, or other reasons?

8. The level of mature DCs in the mice after various treatments should be evaluated.

9. The levels of immune-related cells in the metastatic tumor should be evaluated.

10. Grammar and format errors should be revised, and the language should be improved.

For example, Line 103: “avoided” should be “avoid”.

Line 126: “make” should be “makes”.

Line 132: “release” should be “releases”.

Line 133: “kill” should be “kills”.

Line 134: “stimulate” should be “stimulates”.

Line 139: “This” should be “The” or “These”.

The caption of Figure 3k is misleading. The CLSM images may show the 4T1 cells treated with the supernatant, not the supernatant only.
Some units cannot be seen (such as Line 641, 734, 737, and 790).
Some acronyms (e.g., SPP@ZnPc, Cal@LiP, Cal@DiI, and Oxa(IV)@LiP) should be defined in the main text (although some are defined in the experiment section).

Reviewer #2 (Remarks to the Author): with expertise in breast cancer and bone metastasis

This manuscript addresses an interesting approach to deliver therapies to breast tumors in the primary and bone sites. They showed strong potential therapy using this approach, but there are a few improvements that need to be made to this manuscript. Overall, it describes a novel approach to drug delivery that could be of interest to a general audience and potentially applicable to other diseases.
1) There are numerous grammatical errors and oddly worded sentences throughout the manuscript. The bulk of them are in the introduction. I would suggest very closely reading and editing this section. Some examples include:
a. Line 52/53: Despite promising, immunotherapy is restricted to only a fraction of cases and tumor types, and causes specific immunotoxicity in some patients.
i. I think promise instead of promising. Overall, the sentence is unclear. Do you mean that only a fraction of breast cancer patients are treated with immune therapy or that only a fraction on immune therapy have promising outcomes? Also, you should emphasize/clarify the immunotoxicity comment.
b. Line 74-77: However, well-vascularized primary tumors usually have tortuous vasculatures and elevated hydrostatic pressure, resulting in poor penetrability, while bone metastatic tumors especially frequency micrometastatic nodules are limited in well-build vasculature.
i. I am not sure if there are extra words or missing words here. Either way it is unclear and should be revised.
ii. Well-build should be well-built
c. Line 105: report on macrophages-mediated: macrophage-mediated
d. Line 160:The number-average sizes. Not sure on this, but maybe you mean, the average size?
e. Lines 502 and 505: Both of these sentences begin with Excitingly. Consider using something different in one of these sentences, since they are very close together.
2) The introduction is very long. It seems like some of the discussion of the results that is in the introduction would be better placed in the discussion.
3) Vaccine-like: I understand what the authors mean by this, but I would argue that it isn’t really vaccine-like since the tumors are formed and while this approach reduces tumors and prevents metastasis, I don’t think this study proves it is through functioning like a vaccine/preventative. The only sections that emphasize vaccine-like are the introduction and conclusion, and I would re-phrase it. Otherwise, I think more work needs to be performed to show it is vaccine-like, and I don’t know that it is critical to this study.
4) More discussion regarding the 4T1s is needed. The introduction sets up a lack of immune response (cold-tumor). It is generally accepted that 4T1s are not very immunogenic tumors, but

the authors should point this out and discuss it.
5) The authors just use the 4T1 model. I appreciate that there was a lot of work that went in to the animal studies that they report here. However, using another model would strengthen the study. Would another breast cancer model show similar results? How universal is this response?
6) The toxicity data establishes that the drug has limited toxicity, but I was curious about when the drug does kill a tumor and the tumor releases antigents. What about collateral damage? Since these cytotoxic agents kill normal cells to some extent too (especially within or near the tumor), could this approach lead to increased auto immune responses?

Reviewer #3 (Remarks to the Author): with expertise in breast cancer and macrophages

The authors evaluate oxaliplatin nanoparticle-loaded macrophages for their ability to inhibit the growth of mammary carcinoma and bone lesions. They find that the loaded macrophages can reduce tumor growth, especially in combination with anti-PD-L1 therapy, and that this efficacy correlates with increased T cell recruitment. The authors also find that the use of macrophages appears to improve drug delivery into tumors. However, the lack of appropriate controls for the in vivo studies make it unclear whether the use of macrophages improves therapeutic efficacy, and there is little in the way of mechanistic studies. The overall significance of the study is therefore limited.

1) The study claims that loading macrophages improves efficacy, but the data in Figure 5 and 6 very clearly demonstrate equivalent efficacy between Oxa(IV)@ZnPc and Oxa(IV)@ZnPC@M. Significance is observed when anti-PD-L1 is combined with Oxa(IV)@ZnPC@M, but the control group without macrophages is not included for a comparison.

2) Similarly, most of the immune observations require the presence of anti-PD-L1, but the only control group includes unloaded macrophages with anti-PD-L1. The authors do not demonstrate that Oxa(IV)@ZnPC@M + anti-PD-L1 is more efficacious than Oxa + anti-PD-L1. Given the known immunogenic potential of Oxa, this is a critical control group to include in the studies.

3) There is no demonstration that loaded macrophages are entering the tumors. It is possible that the cells are releasing the cargo from other sites.

4) There are no in vivo mechanistic studies performed, only in vitro observations that are claimed to represent mechanism of action. For example, M1 polarization is said to be important for efficacy, but it is not clear if the macrophages retain this phenotype in vivo. A role for HMGB1, CRT, etc are also not validated.

Reviewer #4 (Remarks to the Author): with expertise in nanoparticle and immunotherapy

In this manuscript, Huang and co-authors reported an engineered macrophages carrying nanomedicine containing oxaliplatin and photosensitizer for chemo/photo/immune-therapy of primary and bone metastatic breast cancer. They found that bone marrow-derived macrophages could effectively load Oxa(IV)@ZnPc nanoparticles and elongate the blood cirtulation time and have tumor targeting ability. In addition, they found that the cargos inside polarize macrophages to anti-tumor M1 phenotype. The engineered macrophages can effectively inhibit both primary and metastatic tumor growth, and activate immune-memory effects. This is an interesting study and the conclusions are well supported by the data. I have the following concerns for this manuscript:

1. What's the mechanism of Oxa(IV)@ZnPc loading for polarizing bone marrow-derived macrophages toward the proinflammation antitumor M1 phenotypes? Besides cytokine secretion, macrophage surface markers should be measured to further confirm the phenotype.
2. In Fig.1j, what does the white arrows and the blank dots represent? Does Oxa(IV)@ZnPc still exist as lipid nanoparticles inside the macrophages?
3. Characterization on Oxa(IV)-OH and Oxa(IV)-COOH is not clearly enough. There are some peaks unlabeled in Fig.S2 and Fig.S3. Please speculate the peaks in Fig.S4 and Fig.S5.
4. What does the control NPs (SPP@ZnPc and Cal@LiP) represents? Please give size, drug loading content and other characterizations on these nanoparticles.
5. In Fig.S13d, how long was the cells incubated with various drugs? What does red and green color represents? Why are there less cells in the Oxa and Oxa(IV)@ZnPc groups?
6. Please supplement the half-life and AUC results of the pharmacokinetics results in Fig.4a.
7. In the Introduction part (page 1, line 26), the authors describe the "therapeutics inside Oxa(IV)@ZnPc@M undergo spatiotemporally controlled release". How was "spatiotemporally controlled release" reflected?
8. In scheme 1, please speculate the name of each cell types.
9. The manuscript citations on Fig2, 3 and 4 are not in sequential. For example, Fig2f-I appears before Fig2d-e.
10. There are many grammar mistakes in the manuscript. Please carefully polish the whole manuscript.
11. Please give the full names of the abbreviations when they are firstly appeared in the manuscript.

Response to Reviewers

Dear Reviewers:

Thank you for you good comments concerning our manuscript entitled "Artificially engineered macrophages as light-activated drug vectors and in situ vaccine for chemo/photo/immune-therapy of primary and bone metastatic breast cancer" (ID: NCOMMS-20-28585A)". These comments are all valuable and very helpful for revising and improving our paper, as well as the important guiding significance to our researches. We have studied the comments carefully and tried our best to revise our manuscript which we hope meet with approval. All the changes made to the manuscript were highlighted in red. In the following pages are our point-by-point responses to each of the comments of the reviewers.

Responses to the comments of Reviewers

Reviewer #1 (Remarks to the Author): **with expertise in chemo/photodynamic therapy**

Huang et al. develop a macrophage-based NIR-responsive drug-delivering system, and present a detailed in vitro and in vivo assessment of their application in synergistic chemo/photodynamic/immune treatment of primary and bone metastatic tumors. The experiments are well-performed for the most part, and some results are interesting. However, the novelty of the paper is suggested to improve, because light-controlled cell carriers have been well developed in literature (Adv. Funct. Mater. 2015, 35, 2386; ACS Appl. Mater. Interfaces 2017, 9, 5855).

1. Comment: The major novelty of the present work may be the development of light-controlled macrophages as a drug carrier for synergistic cancer therapy. What are the advantages of macrophages as a drug carrier compared to other kinds of cells? Macrophages can be converted into the M1 phenotypes that can inhibit the tumor, but unfortunately, most macrophages were killed after light irradiation. The authors are suggested to give more evidence to present the merits of this strategy.

Response: Thanks for reviewer's comment. The major novelty of this work is not only the development of light-controlled macrophages as a drug carrier for synergistic cancer therapy, but also to engineer an "all in one" biological materials which has excellent integrated advantages, such as high drug loading, light-triggered drug release, potential antitumor function of drug-loaded-macrophage itself, excellent pharmacokinetics profile and superior tumor-homing capacity, and can address various obstacles faced by nano-based and cell-based drug delivery systems. At the same time, the function of macrophages and nanoparticles complement each other and benefit each other, they work as a unit in which macrophages enabled nanoparticles with excellent pharmacokinetics profile and superior tumor-homing capacity, while the loading of nanoparticles enabled macrophages with stronger anti-tumor ability compared with non-drug loaded macrophages. In addition, the distinct specificities of the primary and metastatic tumors exacerbates the difficulty of drug carrier design and effective treatment, while we provide a new treatment platform for simultaneous treatment of the primary and metastatic tumors. Moreover, we pioneered new ideas for the treatment of bone metastases that received very little attention.

In recently years, cell-based systems, such as erythrocytes, platelets, stem cells, monocytes/macrophages, lymphocytes, dendritic cells, neutrophils, have emerged as interesting alternative to biological drug carriers[1]. These cell systems possess numerous advantages including prolonged blood circulation time, high biocompatibility, high mobility and inherent biodegradability[2]. For example, erythrocytes are also named red blood cells (RBCs), in addition to the above advantages, they also have a high surface to volume ratio, which is beneficial for drug loading and surface modification, and they are no nucleus, nucleus, organelles, and DNA, so they are not tumorigenic[3]. Leukocytes including monocytes/macrophages and neutrophils, can sense cancer-related chemokine and cytokine cues, actively transmigrate into the tumor tissues, overcoming various biological barriers and evading unwanted immune reactions[4, 5, 6, 7]. We chose macrophage for the reasons below, a) Macrophage has higher tumor-homing ability compared with RBCs and platelets, b) Macrophage has relatively higher life span compared with neutrophils, c) Compared with stem cells, lymphocytes and dendritic cells, the preparation of macrophage is easier.

Drug loaded macrophages in primary tumor can be killed after light irradiation, while the drug loaded macrophages that home to the bone metastatic tumors without light irradiation may have their own function. Firstly, whether the drug loaded macrophages can retain the M1 phenotype in tumor was verified by detecting of the surface marker (M1 maker CD80 and CD86) using flow cytometry (CytoFLEX-S, Beckman Coulter). The cell membrane dye DiD were used to label macrophages. In order to eliminate the interference of the fluorescent dye ZnPc, which is non-toxic under dark, we use Oxa(IV)@Lip (lipid coated but without ZnPc loading) to replace Oxa(IV)@ZnPc for drug loading. The DiD labelled blank BMMs and drug loaded BMMs were named as BMMs-DiD and Oxa(IV)@Lip@M-DiD. In addition, typical LPS (500 ng/mL) and IFN-γ (40 ng/mL) treated M1 BMMs (M1-DiD) were served as positive control.

Briefly, mice bearing 4T1 tumors were intravenously injected with saline, BMMs-DiD, M1-DiD and Oxa(IV)@Lip@M-DiD. At 8 h post-injection, tumors were harvested, cut into small pieces and ground up, then passed through 200 μm and 70 μm filter to obtain single-cell suspensions. The tumor cells were first incubated with anti-mouse CD16/32 antibody to prevent non-specific staining, then it was further stained with anti-CD80-PE and anti-CD86-PC7 according to manufacturer's instructions, and then analyzed by flow cytometry (CytoFLEX-S, Beckman Coulter). As shown in **Fig. R1**, the population of $CD80^{+}CD86^{+}$ cells in DiD positive macrophages of Oxa(IV)@Lip@M were counted to be about 70%, which was similar to that of LPS treated M1 cells, while blank BMMs only counted to be about 37%, indicating that the drug loaded macrophages could retain its M1 phenotype in the tumor tissue.

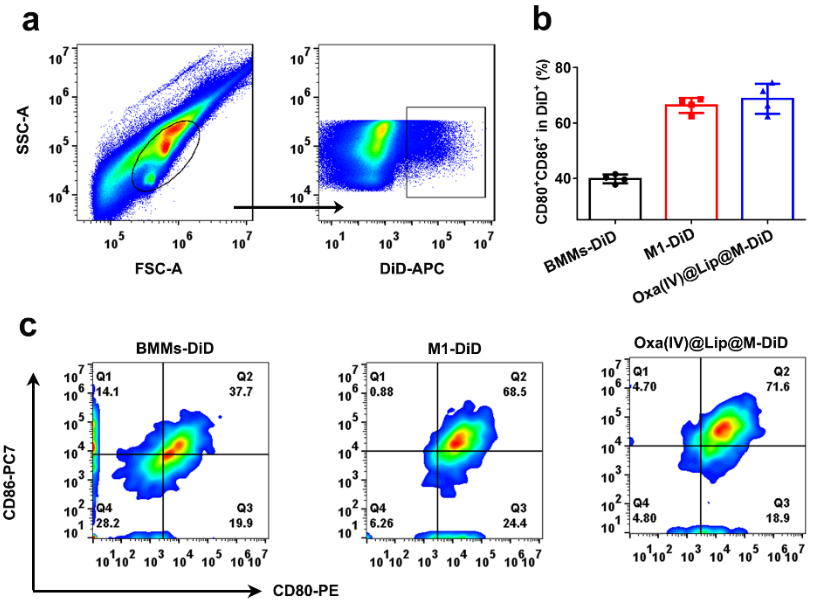

Fig. R1. (a) The gating strategy of macrophage polarization phenotype in tumor tissue. (b) Quantitative analysis of $CD80^+CD86^+$ cells in the DiD positive macrophages. (c) Representative flow cytometric analysis of the expression of M1 marker (CD80, CD86).

Next, whether Oxa(IV)@ZnPc@M with M1 phenotype could re-educate tumor-associated macrophage (TAM) from tumor-promoting M2 phenotype toward tumor-killing M1 phenotype were further evaluated by flow cytometry (CytoFLEX-S, Beckman Coulter), the experiment details were described in comment 7. M1 macrophages were denoted as $CD86^+CD206^-F4/80^+$ cells, and M2 macrophages were denoted as $CD86^-CD206^+F4/80^+$ cells. As show in **Fig. R2**, free Oxa and Oxa(IV)@ZnPc exhibited increased percentage of M1 macrophages and decreased percentage of M2 macrophages compared with PBS group, which might be attributed to the effect of Oxa (comment 5). In comparison with free Oxa and Oxa(IV)@ZnPc, the percentage of M1 macrophages in Oxa(IV)@ZnPc@M group was markedly increased, and the percentage of M2 macrophages was significantly decreased, accompanied by remarkably elevated M1/M2 ratio, which was 1.58-fold of Oxa(IV)@ZnPc and 2.15-fold of free Oxa. The result might be ascribed to the combination effect of Oxa and the M1 phenotype of Oxa(IV)@ZnPc@M that can produce proinflammatory cytokines to re-educate TAM toward tumor-killing M1 phenotypes, acquiring the antitumor effect. Therefore, the M1 phenotype of Oxa(IV)@ZnPc@M itself and its ability to re-educate TAM to M1 phenotype enabled the ability to inhibit the tumor to some extent.

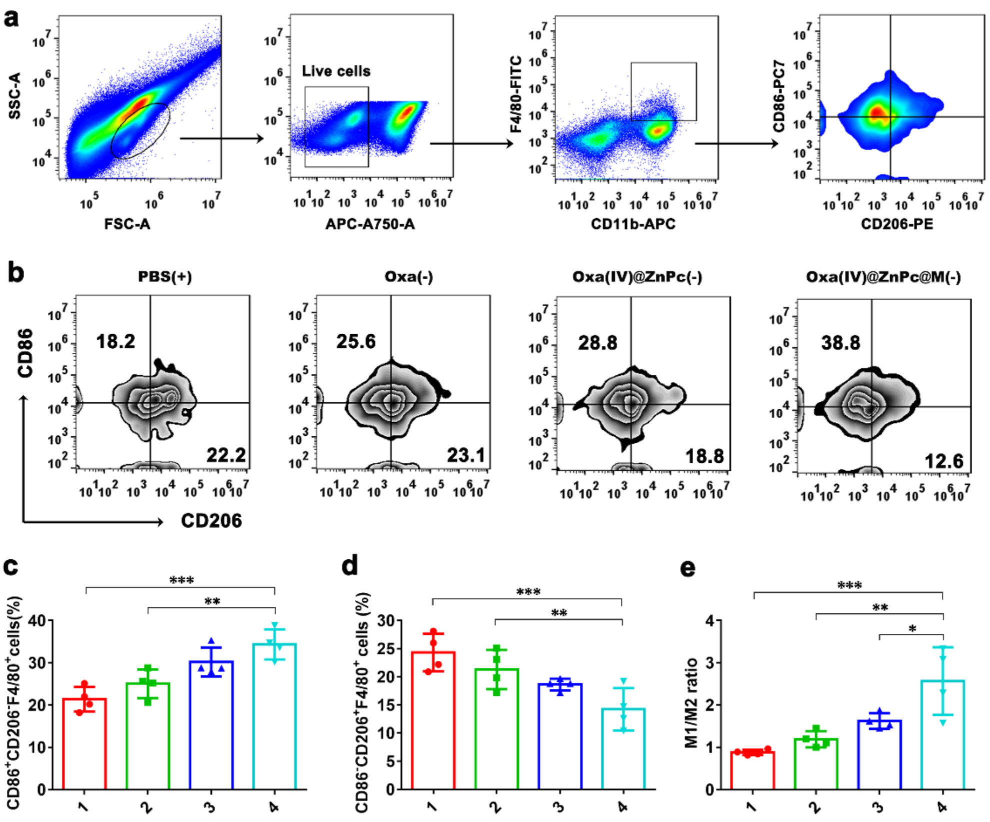

Fig. R2. (a) The gating strategy and (b) the representative flow cytometric plots of M1 macrophages ($CD86^{+}CD206^{-}F4/80^{+}$) and M2 macrophages ($CD86^{-}CD206^{+}F4/80^{+}$) after various treatment (1: PBS (+); 2: free Oxa; 3: Oxa(IV)@ZnPc; 4: Oxa(IV)@ZnPc@M). Quantitation of the percent of (c) M1 macrophages and (d) M2 macrophages in tumors analyzed on day 6. (e) Quantitation of the percent of M1/M2 ratio. **p* <0.05, ***p* <0.01, ****p* < 0.001 (n=4).

2. Comment: Delivering therapeutic agents to both primary and EPR-deficient bone-metastatic nodules may be an advantage of macrophages over nanocarriers (Line 73-78, 98-100). However, as shown in Figure 4c,e, more Oxa(IV)@ZnPc (without macrophages) was detected in the bone tumor than in the primary tumor. After loading into macrophages, the contents of the formulation in the bone tumor and the primary tumor were similar. What is the reason? A better EPR-deficient model is recommended.

Response: Thanks for reviewer's comment. According to **Fig. 4c** and **e** (**Fig. R3b** and **d**), though Oxa(IV)@ZnPc@M had similar contents in the bone tumor and the primary tumor, it is no doubt that the drug loaded macrophages have stronger tumor targeting ability than nanoparticles in both primary tumor and bone tumors. According to the results, is indeed not reasonable to define the bone metastatic tumor as EPR-deficient, we changed the description in the revised manuscript, and EPR-deficient was deleted. Actually, in addition to the EPR

effect, there are so many factors that can affect the tumor distribution behaviors no matter the nano-based or cell-based systems. We know that using a better EPR-deficient model can more perfectly support our paper, we tried to use mice that have been inoculated with tumor cell but did not form visible tumors to serve as the EPR-deficient bone metastatic tumor model, but we failed to get obvious imaging results, so we used mice that have established visible bone metastatic tumors for in vivo fluorescence imaging, but it is not strictly a EPR-deficient tumor model, thus the result may reasonable. We will try our best in building a generally accepted EPR-deficient bone metastatic tumor model in future studies. Thanks you again for you valuable suggestions.

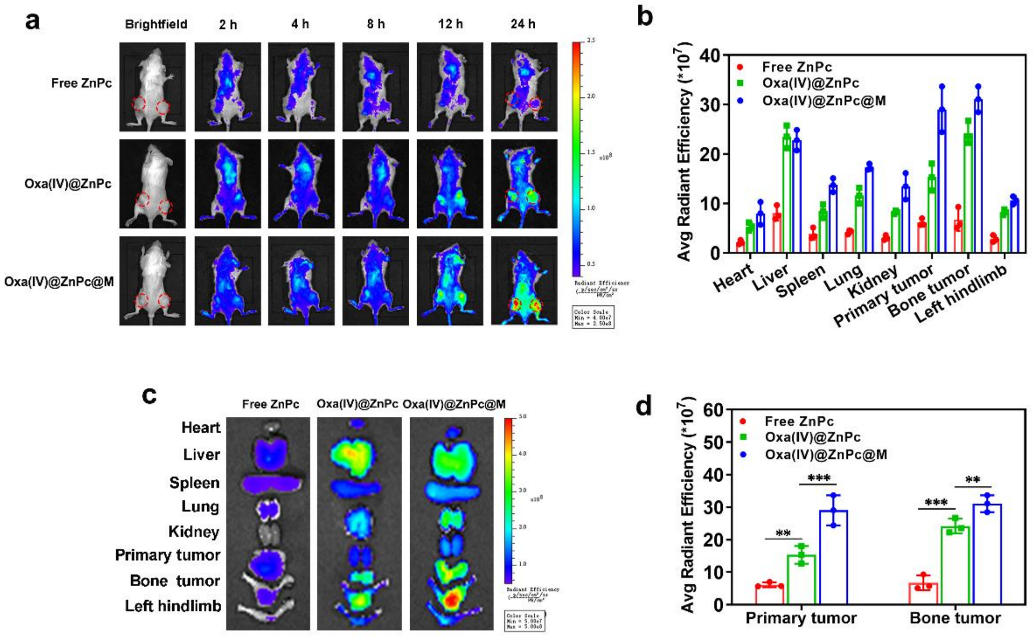

Fig. R3. (a) Fluorescence imaging of free ZnPc, Oxa(IV)@ZnPc and Oxa(IV)@ZnPc@M distribution in primary tumors and bone metastatic tumors in 4T1 tumor-bearing mice. Red circle represents tumor area. (b) Quantitative fluorescence intensity of the excised organs and tumors at 24 h post-injection. (c) Ex vivo fluorescence imaging of the excised organs and tumors at 24 h post-injection. (d) Quantification fluorescence intensity of primary tumors and bone metastatic tumors. **p* <0.05, ***p* <0.01, ****p* < 0.001 (n=3).

3. Comment: The mechanism of the drug (Oxa and ZnPc) release from Oxa(IV)@ZnPc should be further investigated. Besides, for Oxa(IV)@ZnPc@M without irradiation, is the drug (Oxa and ZnPc) release from the system due to their exocytosis from the macrophages? Or the drug was first released from Oxa(IV)@ZnPc and then from macrophages.

Response: Thanks for reviewer's comment. The mechanism of the drug release from Oxa(IV)@ZnPc was further evaluated. Since ZnPc is highly hydrophobic, we investigated the

release behavior of Oxa and ZnPc respectively. For in vitro drug release of Oxa from Oxa(IV)@ZnPc, briefly, 1mL of Oxa(IV)@ZnPc suspension was sealed in dialysis bag (MWCO: 3500 Da), and then immersed in 50 mL of PBS buffer (pH 7.4, 6.5 and 5.0) with stirring at 100 rpm in a shaker at 37 ℃. Periodically, 2 mL of sample was withdraw from the medium and replaced with an equal volume of buffer solution. The samples were dried, redissolved in 1% HNO_3, and the platinum content was detected by GFAAS (AAnalyst 800, Perkin–Elmer, Norwalk, CT, USA). For in vitro drug release of ZnPc from Oxa(IV)@ZnPc, PBS buffer containing 2% tween 80 (pH 7.4, 6.5 and 5.0) was used, and the content of ZnPc was directly detected by fluorescence spectrometer (Fluoromax-4, HORIBA, USA, Ex = 670 nm, Em = 690 nm).

The release of platinum from Oxa(IV)@ZnPc was in a pH dependent manner, with less than 20% was released at pH 7.4 within 12 h, while a significantly increased release at pH 5.5 was observed (**Fig. R4a**). This was attributed to the formation of the characteristic acid-cleavable coordination bonds[8, 9] in the core of Oxa(IV)@ZnPc. Free ZnPc is an extremely hydrophobic photosensitizer, it can easily accumulate in the aqueous, resulting in UV-vis and fluorescence quenching (**Fig. R4c-d**). Therefore, it is hard to investigate the in vitro drug release behavior of ZnPc. After a series of screening, we tried to use PBS buffer containing 2% tween 80 as release medium. The release of ZnPc from Oxa(IV)@ZnPc is slow under the current release medium. The drug release rate at pH 5.5 is slightly higher than pH 6.5 and pH 7.4, this might be due to that the acid-cleavable coordination bonds in the core of Oxa(IV)@ZnPc could fracture in acidic conditions, leading to the decomposition of nanoparticles and the re-assembly of the outer lipid.

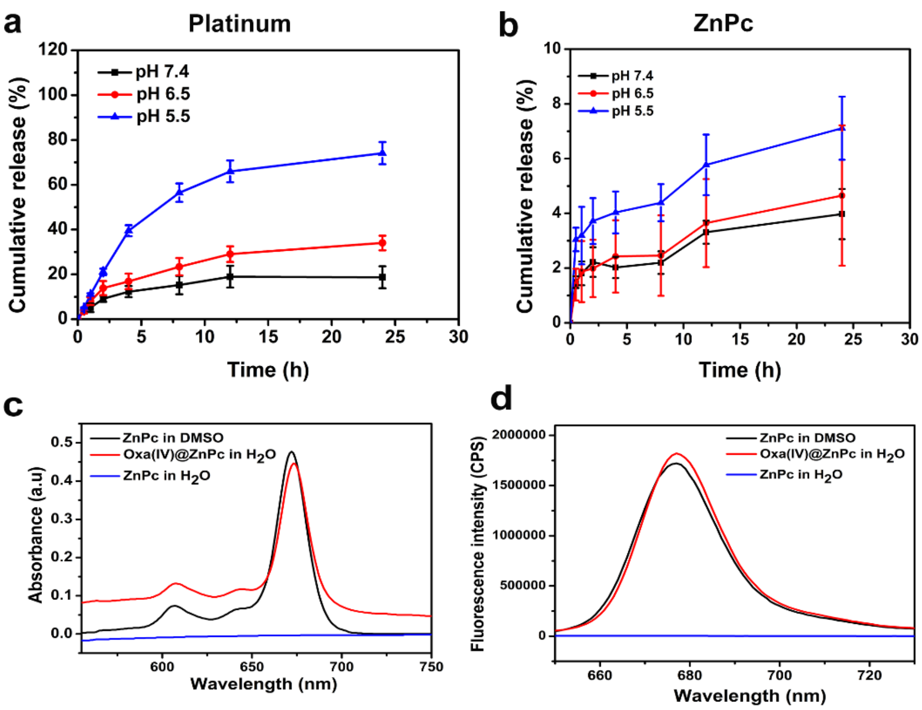

Fig. R4. (a) In vitro release profile of platinum from Oxa(IV)@ZnPc in PBS at different pH. (b) In vitro release profile of ZnPc from Oxa(IV)@ZnPc in PBS containing 2% tween 80 at different pH. (c) UV-vis spectrum and (d) Fluorescence emission spectrum of ZnPc in DMSO, ZnPc and Oxa(IV)@ZnPc in H_2O.

To investigate the drug release patterns (free drug or nanoparticles) from Oxa(IV)@ZnPc@M, for the purpose of convenience and sensitivity of testing, the model nanoparticle Cal@ZnPc (using fluorescent dye calcein to replace Oxa prodrug), the core of which was constructed by fluorescent dye calcein and ZnPc was loaded in the lipid bilayer, was used for this experiment. Briefly, BMMs were loaded with Cal@ZnPc for 2 h, washed three times with ice-cold PBS, and then dispersed in 1.3 mL of DMEM contain 10% FBS and placed in 37℃ incubator. At predetermined time points, the mixture was centrifuged (1000 g, 5 min), supernatant (1 mL) was withdraw and an equal volume of fresh medium was added. The collected supernatant was centrifuged (17745 g, 20 min) immediately and the 1mL supernatant was withdraw, diluted with DMEM (10% FBS) to 2 mL for fluorescence detection of calcein and ZnPc respectively (Release 1-free drug). The sediment was dispersed in 2 mL H_2O for fluorescence detection of calcein and ZnPc respectively (Release 2-nanoparticle).

The detectable fluorescence intensity of calcein and ZnPc in Release 1 (**Fig. R5a**), indicated that the drug within BMMs can be released in the form of free molecule. The detectable fluorescence intensity of calcein and ZnPc in Release 2 (**Fig. R5b**), indicated that

the drug within BMMs can be partly released in the form of nanoparticles, consistent with the result in **Fig. 3b** and **3e**. According to the above analysis, the release of drug from Oxa(IV)@ZnPc@M could be exist in two patterns, one part released by exocytosis in the form of nanoparticles, the other part was first released from Oxa(IV)@ZnPc and then from macrophages.

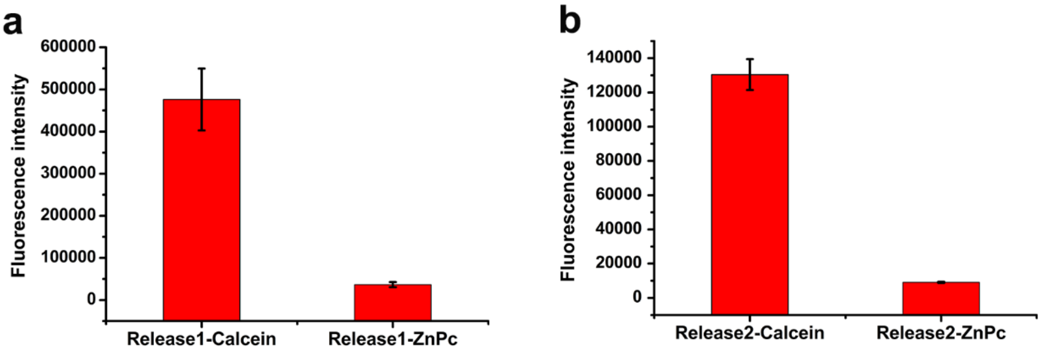

Fig. R5. The fluorescence intensity of calcein and ZnPc in (a) Release 1 and (b) Release 2 in 4h.

4. Comment: Nanoparticles Oxa(IV)@ZnPc (without macrophages) can be captured by the reticuloendothelial system including macrophages, and may form a similar system with the present Oxa(IV)@ZnPc@M. What are the differences between the two systems?

Response: Thanks for reviewer's comment. The drug loading process of the present Oxa(IV)@ZnPc@M is carried out in a static environment in vitro, which can ensure a higher incubation concentration, thus the present Oxa(IV)@ZnPc@M has high drug loading. As nanoparticles can be distributed in various tissues throughout the body after intravenous injection, the amount of nanoparticles that can really bind to the monocytes in the blood can be very small, and since the blood is in a flowing state, which affect the uptake of nanoparticles by the blood monocytes as well. As for nanoparticles captured by the reticuloendothelial system including macrophages, on the one hand, the amount of nanoparticles loading by macrophages of RES also cannot exceed the present Oxa(IV)@ZnPc@M under a safe injection dosage, on the other hand, current reports suggested that the inflammatory monocytes in circulation and the in vitro imported monocytes/macrophages have tumor-tropic property, while these macrophages of RES are mostly tissue-resident macrophages, whether it can actively transmigrate into the tumor tissues is unclear. Thus, the present Oxa(IV)@ZnPc@M

has high drug loading and tumor-tropic properties.

5. Comment: In vitro stability of Oxa(IV)@ZnPc was evaluated within only 24 h (Figure S10d). The long-term stability of the nanoparticles should be evaluated.

Response: Thanks for reviewer's comment. We further evaluated the long-term stability of Oxa(IV)@ZnPc for one week. As show in **Fig. R6,** negligible size changes were detected by dynamic light scattering (DLS) after one week incubation. The result indicted the excellent colloidal stability of Oxa(IV)@ZnPc. We have modified the experimental method and the result and discussion section of the in vitro stability, the short-term stability of Oxa(IV)@ZnPc (**Fig. S9d**) was deleted, and **Fig. R6** was added to the supporting information as **Fig. S9d**. All the changes were highlighted in red.

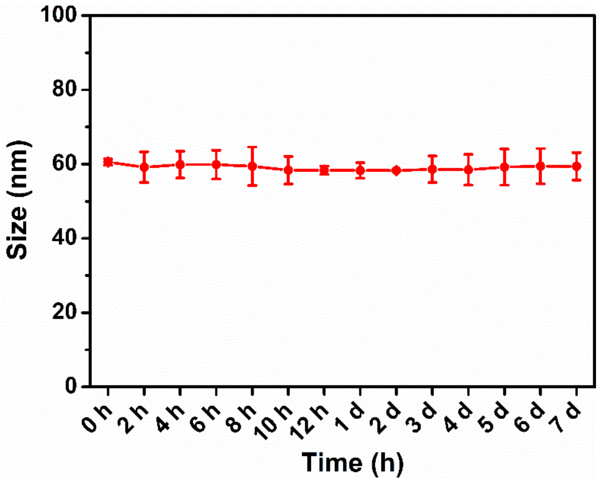

Fig. R6. In vitro stability of Oxa(IV)@ZnPc in PBS containing 10% FBS for one week.

6. Comment: Cal@DiI was used to assess the integrity of the core-shell nanoparticles after entering the cells. Why not use Cal@ZnPc as the model?

Response: Thanks for reviewer's comment. Indeed, as ZnPc is a fluorescent tracer, using Cal@ZnPc (using fluorescent dye calcein to replace Oxa prodrug) can be more directly explain the problem. Therefore, we further performed the experiment using Cal@ZnPc as the model (**Fig. R7**), the results was consistent with that of using Cal@DiI. In the revised manuscript, we replaced **Fig. 1e** with **Fig. R7**, and all the changes were highlighted in red.

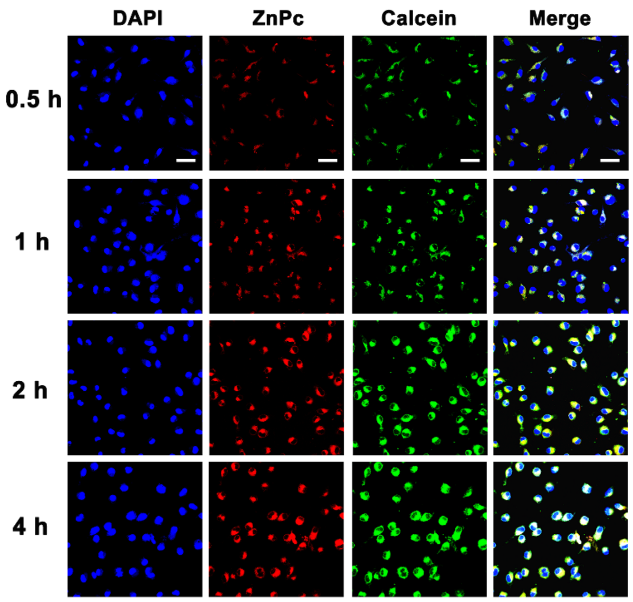

Fig. R7. Cellular uptake by BMMs observed by CLSM at 0.5, 1, 2 and 4 h incubation with Cal@ZnPc nanoparticles. Scale bars = 30 μm.

7. Comment: Oxa(IV)@ZnPc makes the polarization of macrophages to the M1 phenotype. Is it attributed to the released drugs (Oxa and ZnPc), the nanocarriers, or other reasons?

Response: Thanks for reviewer's comment. We performed supplementary experiment to address this question. Briefly, BMMs were seeded in 6-well plates and treated with Oxa(IV)@ZnPc, free ZnPc, ZnPc@Lip (ZnPc loaded lipsome), SPP@ZnPc (control nanoparticle, using low-toxic sodium pyrophosphate (SPP) to replace Oxa prodrug), and free Oxa for 2 h, then cells were washed, after further incubation for 12 h, all cells were harvested, stained with anti-CD80-FITC and anti-CD86-PE and then analyzed by flow cytometry (Guava EasyCyte 6-2L, Merck Millpore). Typical LPS (500 ng/mL) and IFN-γ (40 ng/mL) treated M1 macrophages with the expression of iNOS, and IL-4 (40 ng/mL) treated M2 macrophages with the expression of Arg-1 were used as control, and verified by western bolt (**Fig. R8**). The population of $CD80^+CD86^+$ cells treated with Oxa(IV)@ZnPc showed similar to LPS treated M1 cells (**Fig. R9**), indicating that Oxa(IV)@ZnPc could polarize BMMs toward the proinflammation M1 phenotypes. In order to explore the reason for macrophage polarization, the effect of free ZnPc, free Oxa, ZnPc@Lip and SPP@ZnPc were further compared. Free ZnPc, ZnPc@Lip and SPP@ZnPc showed similar $CD80^+CD86^+$ cells to IL-4 treated M2 cells, while free Oxa with concentration 100 μM-2.5 μM showed similar $CD80^+CD86^+$ cells to LPS

treated M1 cells (**Fig. R9**), indicating that the polarization of macrophages may attributed to the released Oxa. This part of the experiment has been added to the revised manuscript.

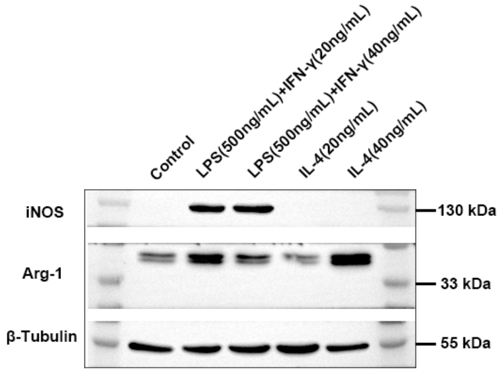

Fig. R8. Western blot detection of iNOS (M1 maker) and Arg-1(M2 Maker) of BMMs after various treatment for 24 h.

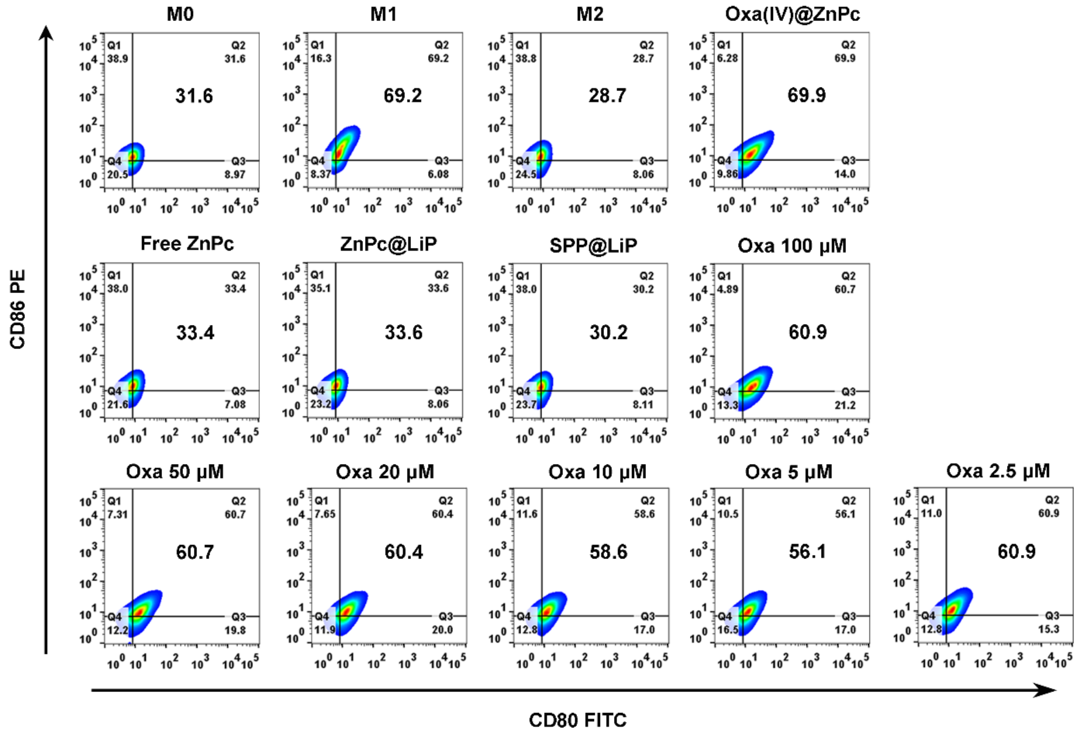

Fig. R9. Flow cytometric analysis of the expression of M1 marker (CD80, CD86) on BMMs after various treatment.

8. Comment: The level of mature DCs in the mice after various treatments should be evaluated.

Response: Thanks for reviewer's comment. The level of mature DCs in the mice lymph nodes after various treatments were further evaluated. Briefly, the mice bearing bilateral 4T1 tumors were randomly assigned into various groups, and received the same treatment regimen

as the anti-tumor experiment. The next day after the first treatment, the tumor draining lymph node from each treatment were harvested, ground up and then passed through 200 μm and 70 μm filter to obtain single-cell suspensions. These cells were stained with CD16/32, eFluor^{TM} 450, anti-CD11c-FITC, anti-CD80-PE and anti-CD86-PC7 antibodies according to the manufacturer's instructions, then analyzed by flow cytometry (CytoFLEX-S, Beckman Coulter) to delineate mature DC cells. The matured DCs were denoted as CD11c^{+}CD80^{+}CD86^{+} cells. According to the flow cytometry result, the Oxa(IV)@ZnPc@M (+) + anti-PD-L1 treatment showed the best effect on promoting DCs maturation, with the expression of CD80 and CD86 were dramatically up-regulated compared with other treatment groups (**Fig. R10 and R11**), indicating the best effect on maturating and activating of DCs, which showing great potential to stimulate the subsequent tumor-specific immune response. This section of experiment were added to the revised manuscript, and all the changes were highlighted in red.

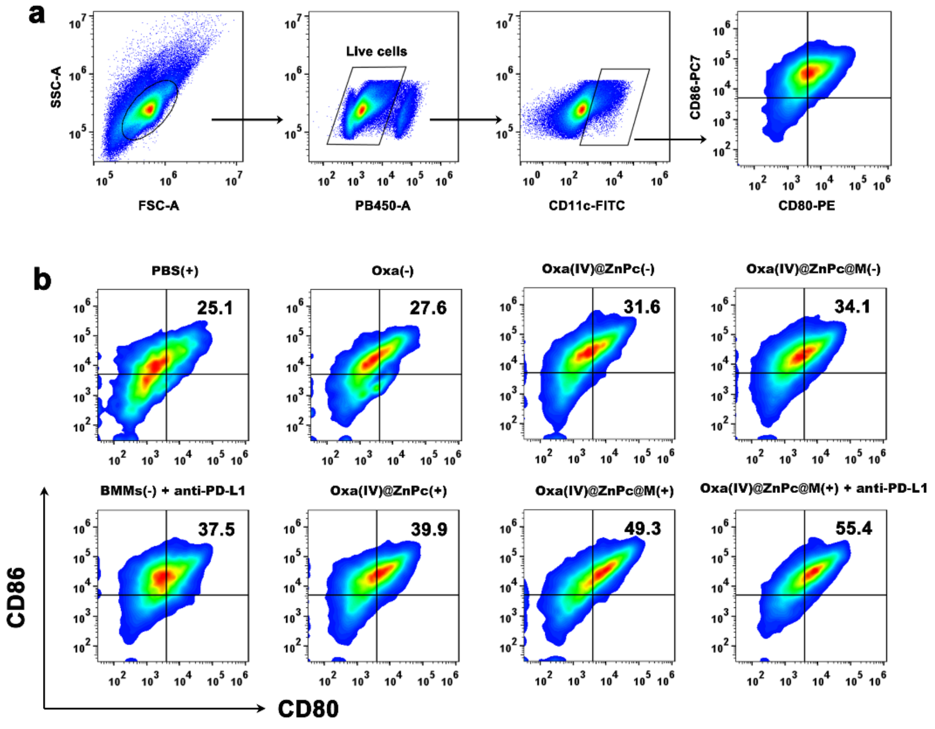

Fig. R10. (a) The gating strategy and (b) the representative flow cytometric analysis of DCs maturation in tumor-draining lymph nodes on day 2 after various treatment.

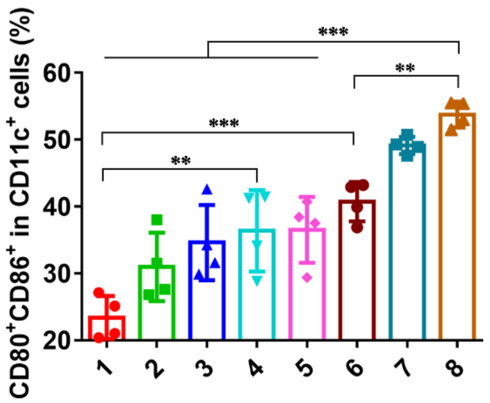

Fig. R11. Quantitation of the percent of mature DCs (CD11c$^+$CD80$^+$CD86$^+$) in tumor-draining lymph nodes on day 2 after various treatment (1: PBS (+); 2: free Oxa; 3: Oxa(IV)@ZnPc; 4: Oxa(IV)@ZnPc@M; 5: BMMs + anti-PD-L1; 6: Oxa(IV)@ZnPc (+); 7: Oxa(IV)@ZnPc@M (+); 8: Oxa(IV)@ZnPc@M (+) + anti-PD-L1, "(+)" represent with laser irradiation). $*p < 0.05$, $**p < 0.01$, $***p < 0.001$ (n=4).

9. Comment: The levels of immune-related cells in the metastatic tumor should be evaluated.

Response: Thanks for reviewer's comment. In the current manuscript, we only detected CD4$^+$ T cells and CD8$^+$ T cells in the metastatic tumors using flow cytometry (Guava EasyCyte 6-2L, Merck Millpore). Due to the limitation of the channels and functions of this flow cytometry (Guava EasyCyte 6-2L, Merck Millpore), we did not do more immune-related indicators. In order to perform immune-related cells detection more rigorously, we re-conducted the experiments with the same treatment regimen as before, and used another flow cytometry (CytoFLEX-S, Beckman Coulter) to analyze the sample. Immune-related cells, cytotoxic T lymphocytes (CTLs) including CD4$^+$ and CD8$^+$ T cells, regulatory T cells (Treg), IFN-γ and TNF-α in CD8$^+$ T cells and tumor-associated macrophage (TAM), were detected by flow cytometry (CytoFLEX-S, Beckman Coulter).

Briefly, the next day after the last irradiation, bone metastatic tumors were harvested, cut into small pieces and ground up, then passed through 200 μm and 70 μm filter to obtain single-cell suspensions. All the cells were treated with ACK Lysis Buffer, washed, and then collected by centrifugation. Cells were first incubating with anti-mouse CD16/32 antibody to prevent non-specific staining. The tumor cells were stained with anti-CD3e-FITC, anti-CD8a-PE and anti-CD4-APC antibodies for CD4$^+$ and CD8$^+$ T cells infiltration evaluation. For Treg evaluation, tumor cells were stained with anti-CD3e-FITC, anti-CD4-APC and anti-Foxp3-PE antibodies. To analyze tumor-associated macrophage (TAMs), tumor cells were stained with

anti-CD11b-APC, anti-F4/80-FITC, anti-CD206-PE and anti-CD86-PC7. In addition, tumor cells were stained with anti-CD3e-FITC, anti-CD8a-PE, anti-IFN-γ-APC and anti-TNF-α-PC7 for assessing IFN-γ and TNF-α in $CD8^+$ T cells. Live/dead cell discrimination was conducted using eFluor^{TM} 780 according to manufacturer's instructions.

As shown in **Fig. R12**, mice treated with Oxa(IV)@ZnPc@M (+) + anti-PD-L1 exhibited the highest effect in promoting intratumoral infiltration of $CD8^+$ and $CD4^+$ T cells , where 9.99% $CD8^+$ T cells and 19.0% $CD4^+$ T cells were induced, which were 5.9-fold and 6.9-fold higher than the PBS (+) group, respectively. Mice treated with BMMs + anti-PD-L1 and Oxa(IV)@ZnPc (+) led to moderate increase of $CD8^+$ and $CD4^+$ T cells, by contrast, mice treated with Oxa(IV)@ZnPc@M (+) showed a lightly increased effect. The populations of Treg ($CD3^+CD4^+Foxp3^+$) of Oxa(IV)@ZnPc@M (+) + anti-PD-L1 group decreased compared with PBS (+) group, and the $CD8^+$T/Treg ratio and $CD4^+$T/Treg ratio were dramatically increased compared with other groups (**Fig. R13**). Except that a higher population of infiltrated $CD8^+$ T cells, interferon-γ (IFN-γ)-positive $CD3^+CD8^+IFN\text{-}\gamma^+$ T cells and TNF-α-positive $CD3^+CD8^+$ $TNF\text{-}\alpha^+$ T cells were also important to the therapeutic effect. Notably, the levels of $CD3^+CD8^+IFN\text{-}\gamma^+$ T cells and $CD3^+CD8^+$ $TNF\text{-}\alpha^+$ T cells in tumor were significantly elevated by Oxa(IV)@ZnPc@M (+) + anti-PD-L1 treatment (**Fig. R14**). All of these results reveled that Oxa(IV)@ZnPc@M (+) treatment or combined with anti-PD-L1 could significantly promote tumor-infiltration of antitumor lymphocyte.

As demonstrated that Oxa(IV)@ZnPc loading could polarize BMMs to anti-tumor M1 phenotype, and the macrophages could retain the M1 phenotype in vivo, we further evaluated whether Oxa(IV)@ZnPc@M could re-educate TAM from tumor-promoting M2 phenotype toward tumor-killing M1 phenotype by flow cytometry, where M1 macrophages were denoted as $CD86^+CD206^-F4/80^+$ cells, and M2 macrophages were denoted as $CD86^-CD206^+F4/80^+$ cells. As show in **Fig. R15**, free Oxa and Oxa(IV)@ZnPc exhibited increased percentage of M1 macrophages and decreased percentage of M2 macrophages compared with PBS group, which might be attributed to the effect Oxa. In comparison with free Oxa and Oxa(IV)@ZnPc, the percentage of M1 macrophages in Oxa(IV)@ZnPc@M group was markedly increased, and the percentage of M2 macrophages was significantly decreased, accompanied by remarkably elevated M1/M2 ratio, which was 1.58-fold of Oxa(IV)@ZnPc and 2.15-fold of free Oxa. The

result might be ascribed to the combination effect of Oxa and the M1 phenotype of Oxa(IV)@ZnPc@M that can produce proinflammatory cytokines to re-educate TAM toward tumor-killing M1 phenotypes, enhancing the antitumor effect.

All of these results indicating the successful elicitation of host defense and cellular immunity by the macrophage mediated chemo/photo/immuo-therapy system. The data of immune related cells in the previous manuscript were replaced by the current data, all the related pictures have been reformatted in the revised manuscript, and all the changes were highlighted in red.

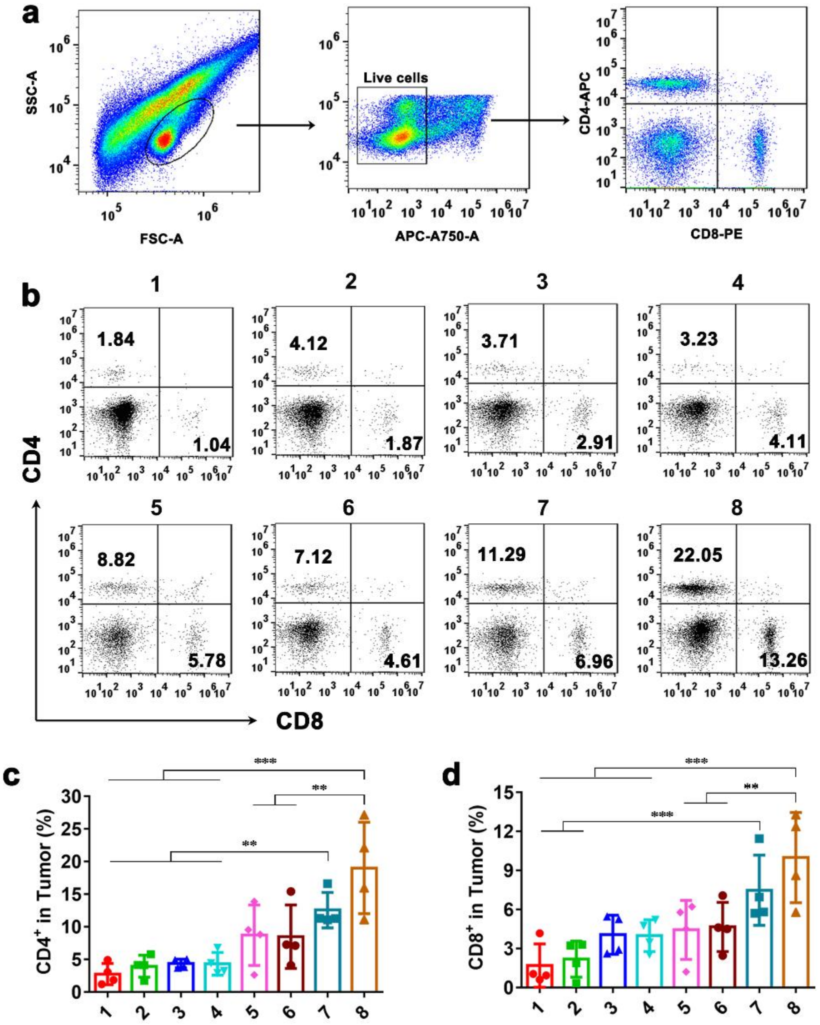

Fig. R12. (a) The gating strategy and (b) the representative flow cytometric plots of tumor-infiltrating $CD4^+$ T cells and $CD8^+$ T cells. Quantitation of the percent of tumor-infiltrating (c) $CD4^+$T cells and (d) $CD8^+$T cell in tumors analyzed on day 6 after various treatment (1: PBS (+); 2: free Oxa; 3: Oxa(IV)@ZnPc; 4: Oxa(IV)@ZnPc@M; 5: BMMs + anti-PD-L1; 6: Oxa(IV)@ZnPc (+); 7: Oxa(IV)@ZnPc@M (+); 8: Oxa(IV)@ZnPc@M (+) + anti-PD-L1, “(+)” represent with laser irradiation). *p <0.05, **p <0.01, ***p < 0.001 (n=4).

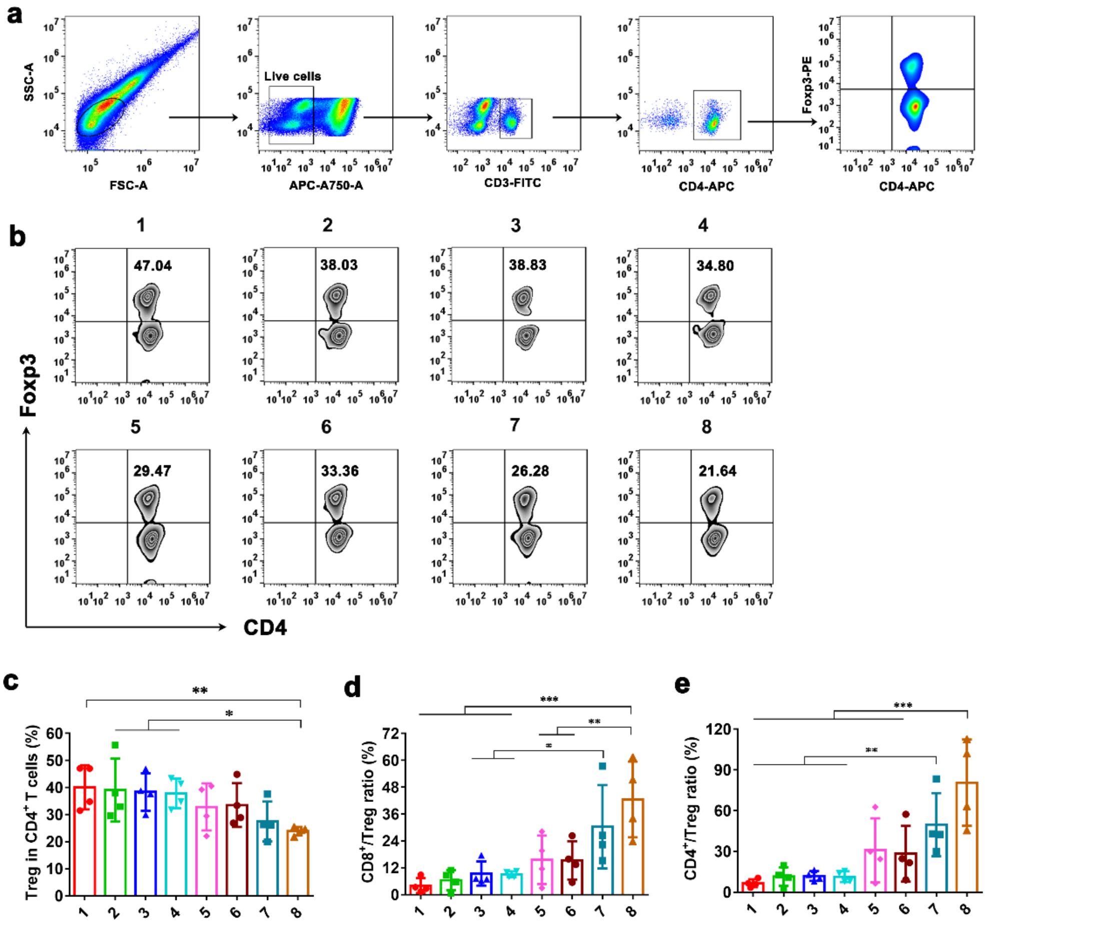

Fig. R13. (a) The gating strategy and (b) the representative flow cytometric plots of Treg ($CD3^{+}CD4^{+}Foxp^{3+}$). (c) Quantitation of the percent of Treg in tumors analyzed on day 6 after various treatment (1: PBS (+); 2: free Oxa; 3: Oxa(IV)@ZnPc; 4: Oxa(IV)@ZnPc@M; 5: BMMs + anti-PD-L1; 6: Oxa(IV)@ZnPc (+); 7: Oxa(IV)@ZnPc@M (+); 8: Oxa(IV)@ZnPc@M (+) + anti-PD-L1, "(+)" represent with laser irradiation). Quantitation of the percent of (d) intratumoral ratio of $CD4^{+}$ T cells to Treg and (e) $CD8^{+}$ T cells to Treg. *p <0.05, **p <0.01, ***p < 0.001 (n=4).

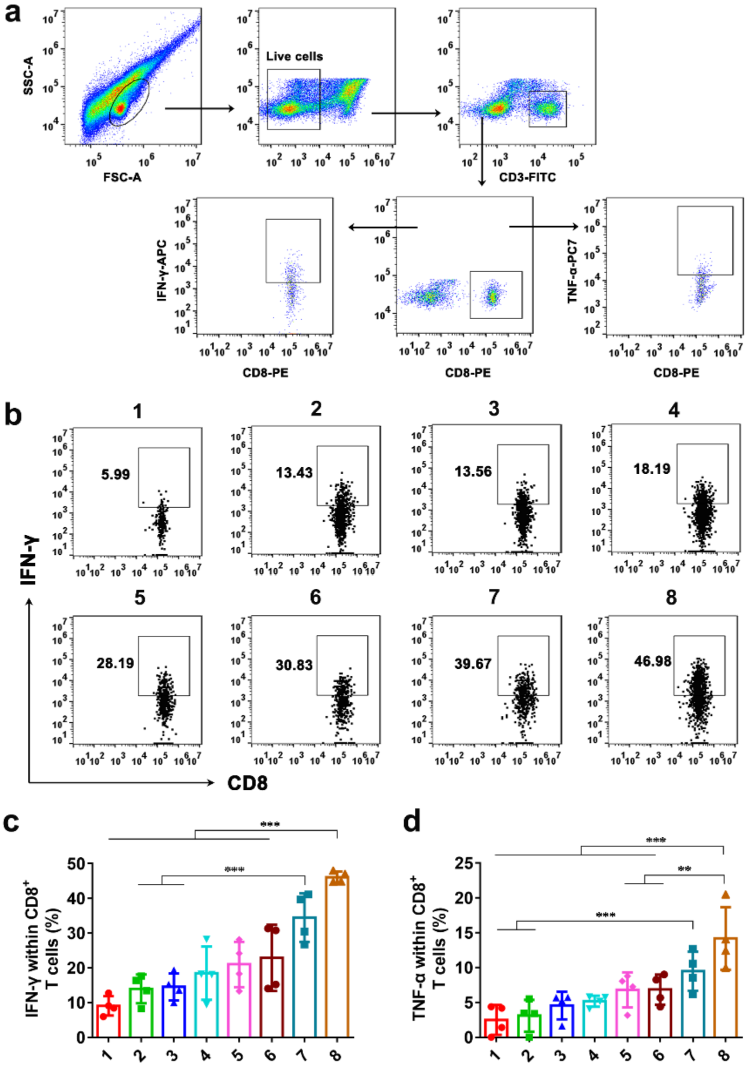

Fig. R14. (a) The gating strategy and (b) the representative flow cytometric plots of IFN-γ within CD8$^+$ T cells. Quantitation of the percent of (c) IFN-γ within CD8$^+$ T cells and (d)TNF-α within CD8$^+$ T cells in tumors analyzed on day 6 after various treatment (1: PBS (+); 2: free Oxa; 3: Oxa(IV)@ZnPc; 4: Oxa(IV)@ZnPc@M; 5: BMMs + anti-PD-L1; 6: Oxa(IV)@ZnPc (+); 7: Oxa(IV)@ZnPc@M (+); 8: Oxa(IV)@ZnPc@M (+) + anti-PD-L1, "(+)" represent with laser irradiation). **p* <0.05, ***p* <0.01, ****p* < 0.001 (n=4).

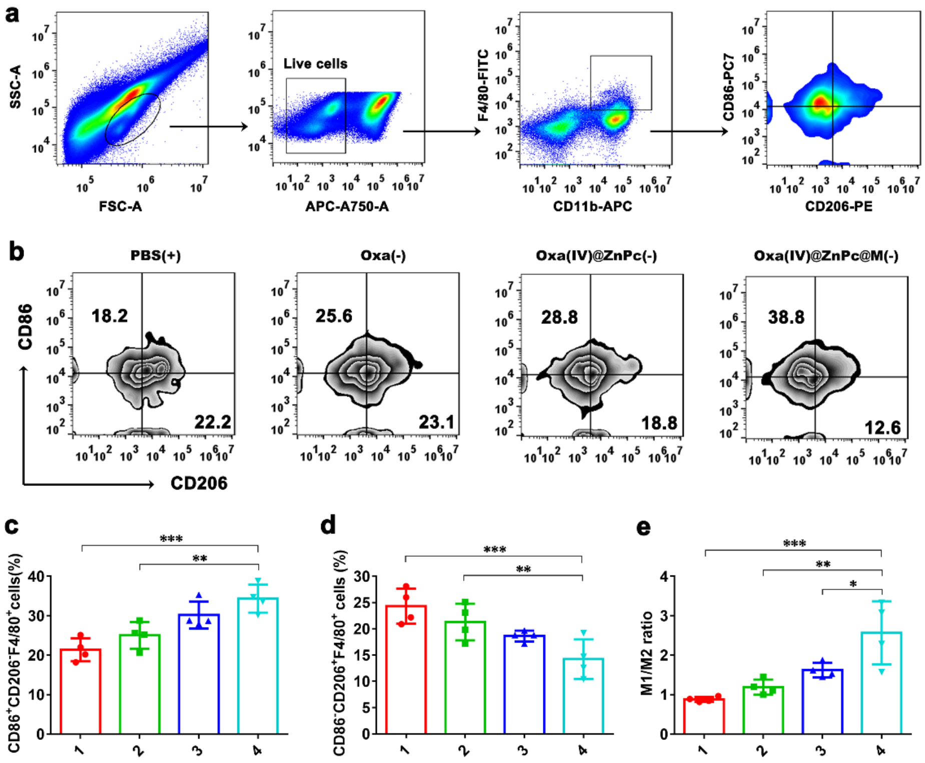

Fig. R15. (a) The gating strategy and (b) the representative flow cytometric plots of M1 macrophages (CD86$^+$CD206$^-$F4/80$^+$) and M2 macrophages (CD86$^-$CD206$^+$F4/80$^+$). Quantitation of the percent of (c) M1 macrophages and (d) M2 macrophages analyzed on day 6 after various treatment (1: PBS (+); 2: free Oxa; 3: Oxa(IV)@ZnPc; 4: Oxa(IV)@ZnPc@M). (e) Quantitation of M1 macrophages to M2 macrophages.**p* <0.05, ***p* <0.01, ****p* < 0.001 (n=4).

10. Comment: Grammar and format errors should be revised, and the language should be improved.

For example, Line 103: “avoided” should be “avoid”.

Line 126: “make” should be “makes”.

Line 132: “release” should be “releases”.

Line 133: “kill” should be “kills”.

Line 134: “stimulate” should be “stimulates”.

Line 139: “This” should be “The” or “These”.

The caption of Figure 3k is misleading. The CLSM images may show the 4T1 cells treated with the supernatant, not the supernatant only.

Some units cannot be seen (such as Line 641, 734, 737, and 790).

Some acronyms (e.g., SPP@ZnPc, Cal@LiP, Cal@DiI, and Oxa(IV)@LiP) should be defined in the main text (although some are defined in the experiment section).

Response: Thanks for reviewer's comment. The above grammar and format errors were checked and revised in the revised manuscript. Some acronyms used in the main text including SPP@ZnPc, Cal@ZnPc and Oxa(IV)@Lip were defined in the main text for better understanding. Cal@DiI was deleted in the revised manuscript. Some missing unit is "°C", this may be due to a problem with the submission system when converting word to PDF. Besides, the entire manuscript was carefully checked and some writing and grammatical mistakes were corrected in the revised manuscript, and all the changes made in the manuscript were highlighted in red. We appreciate the reviewer so much to help us improve our manuscript.

Reviewer #2 (Remarks to the Author): **with expertise in breast cancer and bone metastasis**

This manuscript addresses an interesting approach to deliver therapies to breast tumors in the primary and bone sites. They showed strong potential therapy using this approach, but there are a few improvements that need to be made to this manuscript. Overall, it describes a novel approach to drug delivery that could be of interest to a general audience and potentially applicable to other diseases.

1. Comment: There are numerous grammatical errors and oddly worded sentences throughout the manuscript. The bulk of them are in the introduction. I would suggest very closely reading and editing this section. Some examples include:

a. Line 52/53: Despite promising, immunotherapy is restricted to only a fraction of cases and tumor types, and causes specific immunotoxicity in some patients.

i. I think promise instead of promising. Overall, the sentence is unclear. Do you mean that only a fraction of breast cancer patients are treated with immune therapy or that only a fraction on immune therapy have promising outcomes? Also, you should emphasize/clarify the immunotoxicity comment.

b. Line 74-77: However, well-vascularized primary tumors usually have tortuous vasculatures and elevated hydrostatic pressure, resulting in poor penetrability, while bone metastatic tumors especially frequency micrometastatic nodules are limited in well-build vasculature.

i. I am not sure if there are extra words or missing words here. Either way it is unclear and should be revised.

ii. Well-build should be well-built

c. Line 105: report on macrophages-mediated: macrophage-mediated

d. Line 160:The number-average sizes. Not sure on this, but maybe you mean, the average size?

e. Lines 502 and 505: Both of these sentences begin with Excitingly. Consider using something different in one of these sentences, since they are very close together.

Response: Thanks for reviewer's comment. The grammatical errors and oddly worded sentences in the entire manuscript was carefully checked, and some writing and grammatical mistakes were corrected in the revised manuscript, and all the changes made in the manuscript were highlighted in red. We appreciate the reviewer so much to help us improve our manuscript.

The sentence "Despite promising, immunotherapy is restricted to only a fraction of cases and tumor types, and causes specific immunotoxicity in some patients" was revised as "Despite promise, only a fraction of cancer patients benefit from current immunotherapy, and the lack of tumor specificity causes specific immunotoxicity in a considerable subset of patients receiving treatment".

The sentence in Line 74-77 was revised as "However, well-vascularized primary tumors usually have tortuous vasculatures and elevated hydrostatic pressure, resulting in poor penetrability. While bone metastatic tumors, especially multiple micrometastatic nodules, are limited in well-built vasculature, leading to inadequate drug concentration in metastatic tumor to fully realize the synchronous therapeutic potential"

Macrophages-mediated were revised as macrophage-mediated in the entire manuscript.

Line 160: The number-average sizes mean the value of average sizes were displayed in number (Percent) but not in intensity (Percent). In order to avoid ambiguity, we revised the description as "the average sizes".

e. Lines 502 and 505: Both of these sentences begin with Excitingly. Consider using something different in one of these sentences, since they are very close together. The duplicate description was revised.

2. Comment: The introduction is very long. It seems like some of the discussion of the results that is in the introduction would be better placed in the discussion.

Response: Thanks for reviewer's comment. We tried our best to condense the

introduction, the sentence that discuss the results in the introduction were deleted or moved to the part of discussion. The detected contents were listed as follows. We appreciate the reviewer so much to help us improve our manuscript.

Oxa(IV)@ZnPc, a kind of coordination polymer nanoparticles, is a class of burgeoning materials self-assembled from metal ions and organic bridging ligands, and it has tunable sizes and compositions, high drug loading and inherent biodegradability.

Oxa(IV)@ZnPc possesses two therapeutic modalities: chemotherapy and PDT. It can directly kill cancer cells by combined chemo-photodynamic therapy, and enhance in vitro ICD induction ability to promote antitumor immunity.

Then they work as a unit, in which the functions of macrophages and nanoparticles complement each other and benefit each other.

Infiltration of cytotoxic $CD8^+$ and $CD4^+$ T cells with ultra-high biocompatibility and little systemic toxicity. Furthermore, the combined chemo/photo/immuno-therapy strategy shows a long-term immune memory function to protect against the subsequent tumor challenge and prolong overall survival.

3. Comment: Vaccine-like: I understand what the authors mean by this, but I would argue that it isn't really vaccine-like since the tumors are formed and while this approach reduces tumors and prevents metastasis, I don't think this study proves it is through functioning like a vaccine/preventative. The only sections that emphasize vaccine-like are the introduction and conclusion, and I would re-phrase it. Otherwise, I think more work needs to be performed to show it is vaccine-like, and I don't know that it is critical to this study.

Response: Thanks for reviewer's comment. For the sake of rigor, Vaccine-like was re-phrased as "immunogenic cell death (ICD) or deleted" in the entire manuscript. We appreciate the reviewer so much to help us improve our manuscript.

4. Comment: More discussion regarding the 4T1 is needed. The introduction sets up a lack of immune response (cold-tumor). It is generally accepted that 4T1s are not very immunogenic tumors, but the authors should point this out and discuss it.

Response: Thanks for reviewer's comment. We added the discussion regarding 4T1 in

the section of “In vivo antitumor efficiency in 4T1 tumor mouse model” and “Mechanism of systematic antitumor immune responses”, and it was describe as “ 4T1 is a well-known aggressive tumor model with high metastasis rate, we first evaluate the antitumor effect in 4T1 tumor model”, and “As 4T1 is a highly accepted poorly immunogenic tumor model[10], we firstly used the 4T1-tumor-bearing mice to verify whether Oxa(IV)@ZnPc@M-augmented chemo-photodynamic therapy could create a immunogenic hot tumor microenvironment to boost antitumor immune response”, respectively.

5. Comment: The authors just use the 4T1 model. I appreciate that there was a lot of work that went in to the animal studies that they report here. However, using another model would strengthen the study. Would another breast cancer model show similar results? How universal is this response?

Response: Thanks for reviewer’s comment. EMT6 is a representative preclinical model of Triple Negative Breast Cancer (TNBC), similar to 4T1, it was commonly used for immunological evaluation[11, 12]. Thus, the anti-tumor effect and the mechanism of systematic antitumor immune responses were further verified on another breast cancer cell model EMT6 model. The EMT6 cells were purchased from FuHeng Cell Cente (Shanghai, China) and maintained in RPMI-1640 medium containing 10% FBS and 1% antibiotics. The bilateral EMT6 tumor model was established similar to that of 4T1. For more rigorous comparison， in the EMT6 model, two control groups including Oxa + anti-PD-L1 and Oxa(IV)@ZnPc (+) + anti-PD-L1 were added.

To investigate the in vivo anti-tumor efficiency on EMT6, Balb/c female mice (5-6 weeks) were subcutaneously injected with 1×10^6 EMT6 cells in the left flank (primary tumor). When the primary tumors reached about 50 mm^3, which was about 6 days after inoculation, 5×10^5 EMT6 cells were intra-tibia injected into the right tibia using a 26-gauge needle under anesthesia. Six days after intra-tibia injection, the mice bearing primary tumors (100-150 mm^3) and bone metastatic tumors were randomly divided into various groups (n=6), and intravenously injected with various formulations including following: (1) PBS (+), (2) free Oxa, (3) Oxa(IV)@ZnPc, (4) Oxa(IV)@ZnPc@M, (5) BMMs + anti-PD-L1, (6) Oxa(IV)@ZnPc (+), (7) Oxa(IV)@ZnPc@M (+), (8) Oxa(IV)@ZnPc@M (+) + anti-PD-L1, (9) Oxa + anti-

PD-L1 and (10) Oxa(IV)@ZnPc (+) + anti-PD-L1. "(+)" represents laser irradiation. The injection was conducted every two days for three times at an equal platinum dose of 1 mg/kg and ZnPc dose of 0.75 mg/kg (about 1 $\times 10^6$ cells/mouse). Four hour and 24 h post-injection, the primary tumors were irradiated with a NIR laser (671 nm, 250 mW cm^{-2}) and continued for 10 min, respectively. After the second irradiation, mice were intraperitineally injected with anti-PD-L1 antibody (100 μg/mouse). Tumor size and body weight were monitored every other day. Tumor volume was measured using calipers and calculated as length $\times$ width^2 $\times$ 0.5 (**Fig. R16a**).

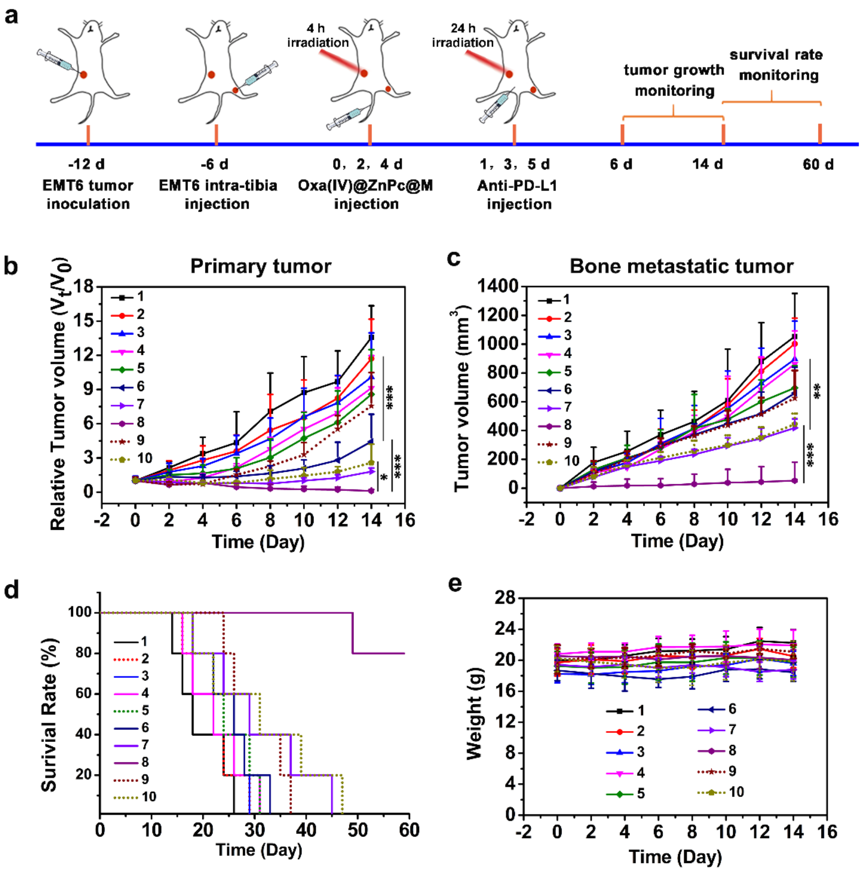

Fig. R16. In vivo antitumor effect of combined chemo/photo/immune-therapy of primary and bone metastatic breast cancer in EMT6 model. (a) Schematic illustration of experimental design. Tumor growth curves of (b) primary tumors and (c) bone metastatic tumors after various treatments (1: PBS (+); 2: free Oxa; 3: Oxa(IV)@ZnPc; 4: Oxa(IV)@ZnPc@M; 5: BMMs + anti-PD-L1; 6: Oxa(IV)@ZnPc (+); 7: Oxa(IV)@ZnPc@M (+); 8: Oxa(IV)@ZnPc@M (+) + anti-PD-L1, 9: Oxa + anti-PD-L1 and 10: Oxa(IV)@ZnPc (+) + anti-PD-L1, "(+)" represent laser irradiation). (d) Overall survival curves of mice after various treatments. (e) Body weight after various treatments. Values were presented as means ± SD (*p <0.05，**p <0.01, ***p < 0.001).

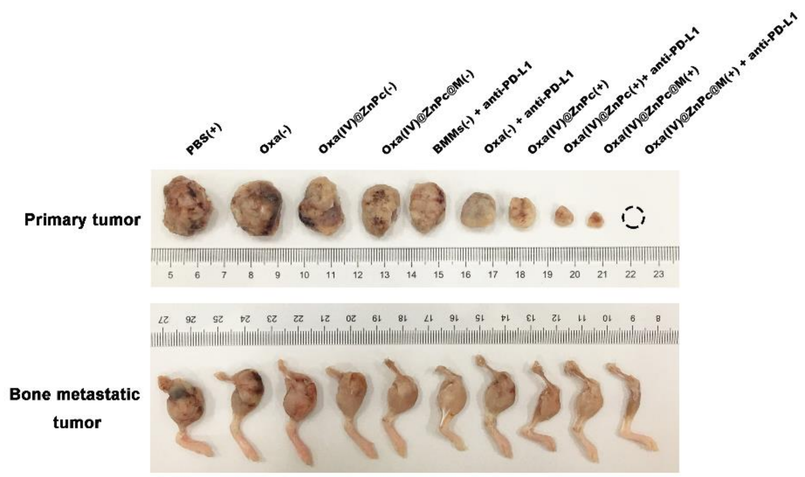

Fig. 17. Representative ex vivo images of primary and bone metastatic tumors after various treatment in day 14.

The mechanism of systematic antitumor immune responses was conducted on breast cancer cell model EMT6 model as well. The immune-related cells, cytotoxic T lymphocytes (CTLs) including $CD4^+$ and $CD8^+$ T cells, regulatory T cells (Treg), IFN-γ and TNF-α in $CD8^+$ T cells and tumor-associated macrophage (TAM), were detected by flow cytometry (CytoFLEX-S, Beckman Coulter). Similar to 4T1 model, the next day after the last irradiation, bone metastatic tumors were harvested, cut into small pieces and ground up, then passed through 200 μm and 70 μm filter to obtain single-cell suspensions. All the cells were treated with ACK Lysis Buffer, washed, and then collected by centrifugation. Cells were first incubating with anti-mouse CD16/32 antibody to prevent non-specific staining. The tumor cells were stained with anti-CD3e-FITC, anti-CD8a-PE and anti-CD4-APC antibodies for $CD4^+$ and $CD8^+$ T cells infiltration evaluation. For Treg evaluation, tumor cells were stained with anti-CD3-FITC, anti-CD4-APC and anti-Foxp3-PE antibodies. To analyze tumor-associated macrophage (TAMs), tumor cells were stained with anti-CD11b-APC, anti-F4/80-FITC, anti-CD80-PE and anti-CD86-PC7. In addition, tumor cells were stained with anti-CD3e-FITC, anti-CD8a-PE, anti-IFN-γ-APC and anti-TNF-α-PC7 for assessing IFN-γ and TNF-α in $CD8^+$ T cells. Live/dead cell discrimination was conducted using eFluor™ 780 according to manufacturer's instructions.

Consistent with the results in 4T1 model, Oxa(IV)@ZnPc@M (+) + anti-PD-L1 treated mice exhibited the highest frequency of $CD8^+$ and $CD4^+$ T cells infiltration, which were 10.4-

fold and 8.7-fold higher than the PBS (+) group, respectively. Mice treated with Oxa(IV)@ZnPc@M (+) showed a slightly increased CD8$^+$ and CD4$^+$ T cells compared with BMMs + anti-PD-L1 and Oxa(IV)@ZnPc (+), and it showed comparable effect to nanoparticles combined with anti-PD-L1 (Oxa(IV)@ZnPc (+) + anti-PD-L1 group) (**Fig. R18a-d**). The proportion of Treg (CD3$^+$CD4$^+$Foxp3$^+$) of Oxa(IV)@ZnPc@M (+) + anti-PD-L1 group decreased compared with PBS (+) group, and the CD8$^+$ T/Treg ratio and CD4$^+$ T/Treg ratio were dramatically increased compared with other groups (**Fig. R18e-h**). Except that a higher population of infiltrated CD8$^+$ T cells, IFN-γ and TNF-α within CD8$^+$ T cells were also important to the therapeutic effect. Notably, the levels of IFN-γ$^+$ and TNF-α$^+$ in CD8$^+$ T cells in tumor was significantly elevated by Oxa(IV)@ZnPc@M (+) + anti-PD-L1 treatment (**Fig. R19**). All of these results reveled that Oxa(IV)@ZnPc@M (+) treatment or combined with anti-PD-L1 could significantly promote tumor-infiltration of antitumor lymphocyte.

As demonstrated that Oxa(IV)@ZnPc loading could polarize BMMs to anti-tumor M1 phenotype, and the macrophages could retain the M1 phenotype in vivo, we further evaluated whether Oxa(IV)@ZnPc@M could re-educate TAM from tumor-promoting M2 phenotype toward tumor-killing M1 phenotype by flow cytometry, where M1 macrophages were denoted as CD86$^+$CD206$^-$F4/80$^+$ cells, and M2 macrophages were denoted as CD86$^-$CD206$^+$F4/80$^+$ cells. As show in **Fig. R20**, free Oxa and Oxa(IV)@ZnPc exhibited increased percentage of M1 macrophages and decreased percentage of M2 macrophages compared with PBS group, which might be attributed to the effect Oxa. In comparison with free Oxa and Oxa(IV)@ZnPc, the percentage of M1 macrophages in Oxa(IV)@ZnPc@M group was markedly increased, and the percentage of M2 macrophages was significantly decreased, accompanied by remarkably elevated M1/M2 ratio, which was 1.66-fold of Oxa(IV)@ZnPc and 2.45-fold of free Oxa. The result might be ascribed to the combination effect of Oxa and the M1 phenotype of Oxa(IV)@ZnPc@M that can produce proinflammatory cytokines to re-educate TAM toward tumor-killing M1 phenotypes, enhancing the antitumor effect.

All of these results indicated the cell-based system had higher effect to elicit the immune system by the combined chemo/photo/immuo-therapy than that of nano-based system. This part of experiment were added to the revised manuscript, the related pictures have been reformatted in the revised manuscript, and all the changes were highlighted in red.

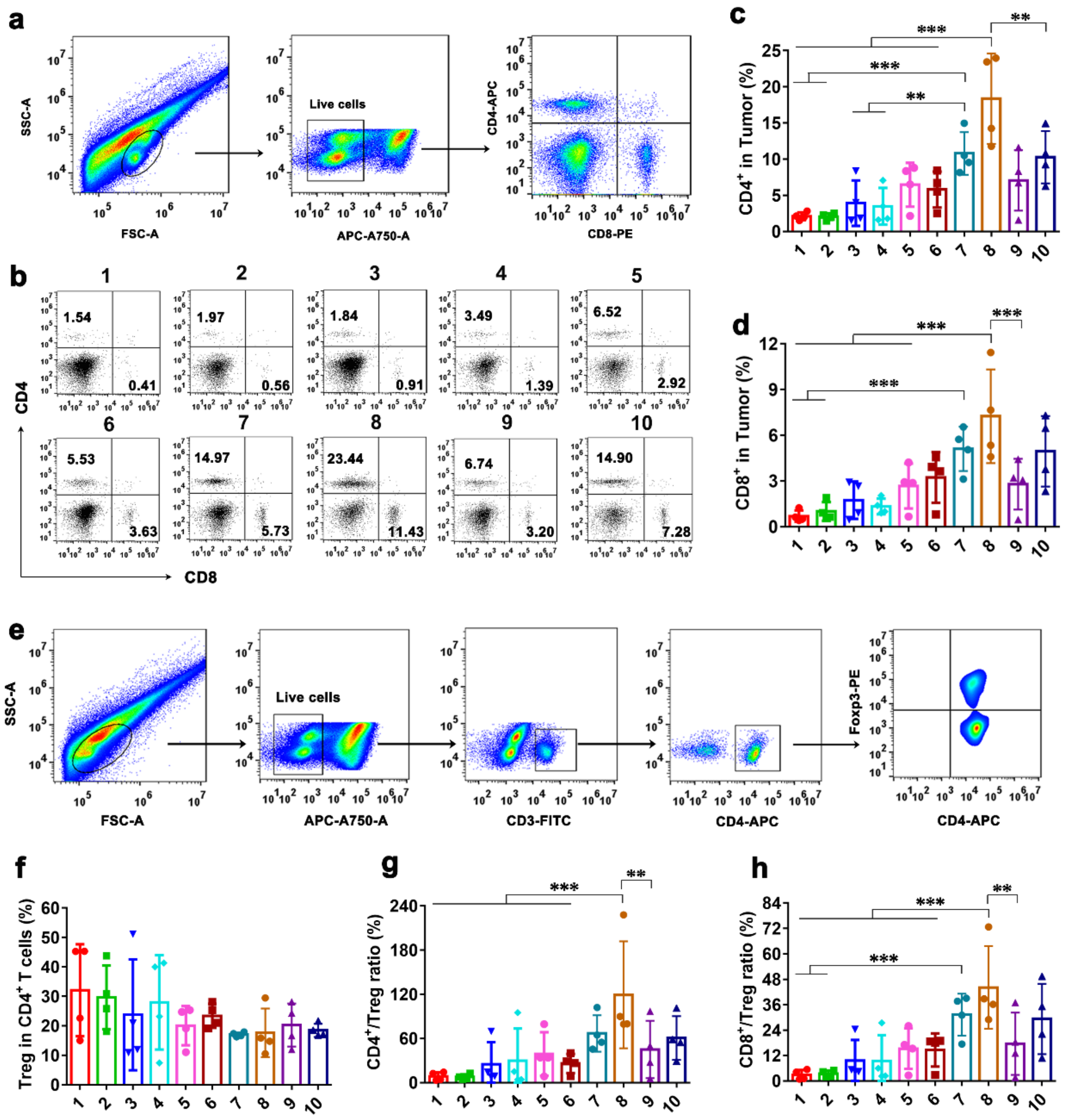

Fig. R18. (a) The gating strategy and (b) the representative flow cytometric plots of tumor-infiltrating CD4^{+} T cells and CD8^{+} T cells. Quantitation of the percent of tumor-infiltrating (c) CD4^{+}T cells and (d) CD8^{+}T cell in tumors analyzed on day 6 after various treatment (1: PBS (+); 2: free Oxa; 3: Oxa(IV)@ZnPc; 4: Oxa(IV)@ZnPc@M; 5: BMMs + anti-PD-L1; 6: Oxa(IV)@ZnPc (+); 7: Oxa(IV)@ZnPc@M (+); 8: Oxa(IV)@ZnPc@M (+) + anti-PD-L1, 9: Oxa + anti-PD-L1 and 10: Oxa(IV)@ZnPc (+) + anti-PD-L1, "(+)" represent with laser irradiation). (e) The gating strategy of flow cytometric analysis of Treg (CD3^{+}CD4^{+}Foxp3^{+}). (f) Quantitation of the percent of Treg in tumors analyzed on day 6 after various treatment. Quantitation of the percent of (g) intratumoral ratio of CD4^{+} T cells to Treg and (h) CD8^{+} T cells to Treg. *p <0.05, **p <0.01, ***p < 0.001 (n=4)

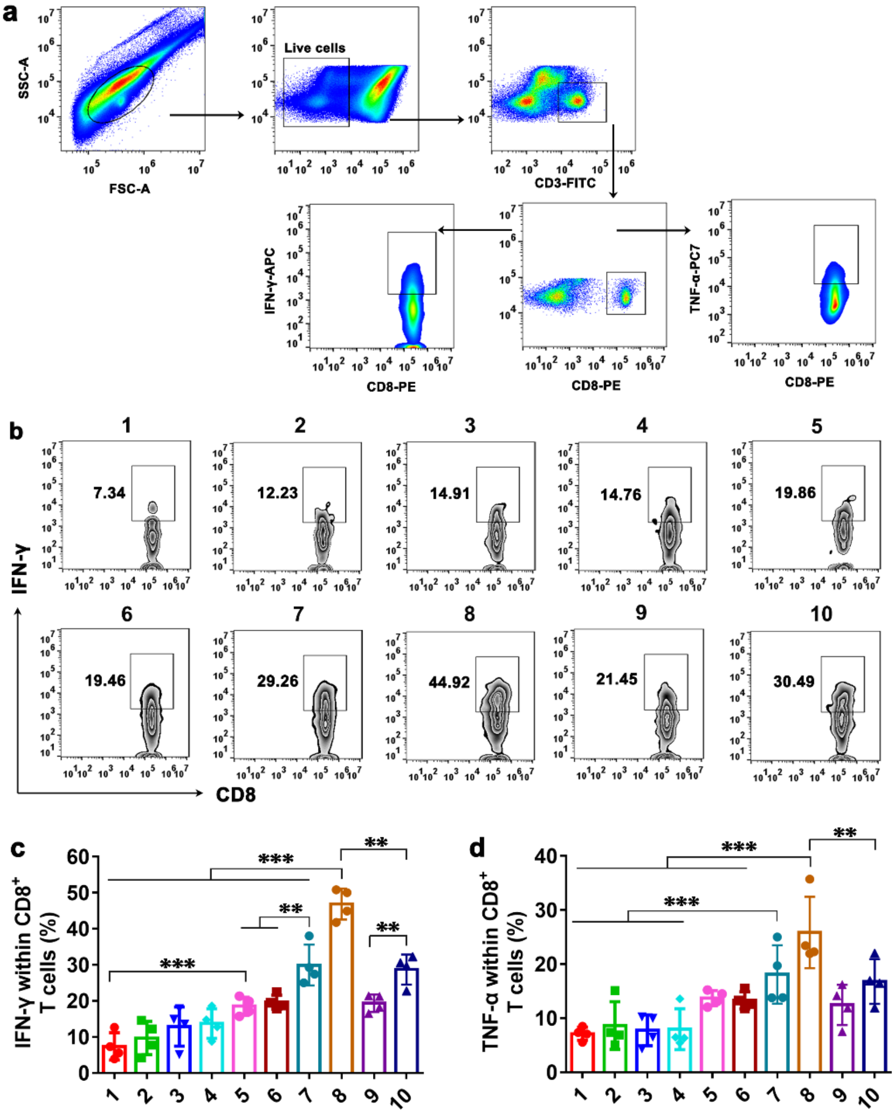

Fig. R19. (a) The gating strategy and (b) the representative flow cytometric plots of IFN-γ within CD8+ T cells. Quantitation of the percent of (c) IFN-γ within CD8$^+$ T cells and TNF-α within CD8$^+$ T cells in tumors analyzed on day 6 after various treatment (1: PBS (+); 2: free Oxa; 3: Oxa(IV)@ZnPc; 4: Oxa(IV)@ZnPc@M; 5: BMMs + anti-PD-L1; 6: Oxa(IV)@ZnPc (+); 7: Oxa(IV)@ZnPc@M (+); 8: Oxa(IV)@ZnPc@M (+) + anti-PD-L1, 9: Oxa + anti-PD-L1 and 10: Oxa(IV)@ZnPc (+) + anti-PD-L1, "(+)" represent with laser irradiation). *p <0.05, **p <0.01, ***p < 0.001 (n=4).

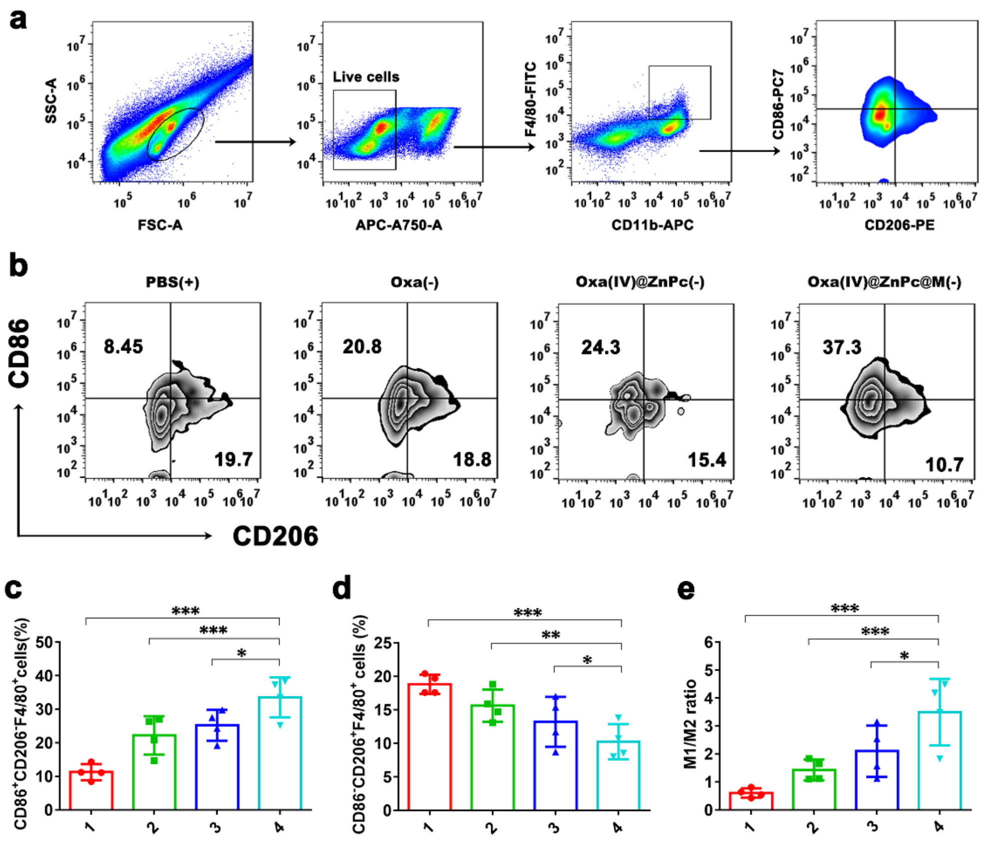

Fig. R20. (a) The gating strategy and (b) the representative flow cytometric plots of M1 macrophages ($CD86^{+}CD206^{-}F4/80^{+}$) and M2 macrophages ($CD86^{-}CD206^{+}F4/80^{+}$). Quantitation of the percent of (c) M1 macrophages and (d) M2 macrophages analyzed on day 6 after various treatment (1: PBS (+); 2: free Oxa; 3: Oxa(IV)@ZnPc; 4: Oxa(IV)@ZnPc@M). (e) Quantitation of M1 macrophages to M2 macrophages.**p* <0.05, ***p* <0.01, ****p* < 0.001 (n=4).

6. Comment: The toxicity data establishes that the drug has limited toxicity, but I was curious about when the drug does kill a tumor and the tumor releases antigens. What about collateral damage? Since these cytotoxic agents kill normal cells to some extent too (especially within or near the tumor), could this approach lead to increased auto immune responses?

Response: Thanks for reviewer's comment. The purpose of this study is to design a tumor targeted system that can accurately deliver cytotoxic agent to the tumor site using engineered macrophages to reduce collateral damage. At the meanwhile, an effective and minimally invasive therapeutic modality PDT was applied, which enabling the cytotoxic agent to be released in a near-infrared (NIR) light controlled mode, further reducing the toxicity to normal tissues. Therefore, this system could increase the accumulation of drugs in tumor tissues, reduce drug distribution in the normal tissues to decrease damage to a certain extent. The NIR-

triggererea, PDT is currently recognized as a low-invasive treatment mode. Chemotherapeutics kill tumor cells and promote antigens release when it was entered into tumor cells and interact with corresponding organelles, while PDT kill tumor cells and promote antigens release when NIR was conducted and corresponding cytotoxic reaction rd release of chemotherapeutics further reduces the damage to normal tissues. PDT, a spatiotemporal controllable treatment mode, though it may cause damage to the normal tissues in the illuminated aeactive oxygen was produced. So far, no treatment is determined to be safe and effective, what we done is try our best to increase efficacy while reducing toxicity. Although we think that auto immune responses is hard to avoid, based on the body weight change and the safety evaluation results, this cell-based formulation is a relatively biocompatibible system that reduces auto immune responses.

Reviewer #3 (Remarks to the Author) : **with expertise in breast cancer and macrophages**

The authors evaluate oxaliplatin nanoparticle-loaded macrophages for their ability to inhibit the growth of mammary carcinoma and bone lesions. They find that the loaded macrophages can reduce tumor growth, especially in combination with anti-PD-L1 therapy, and that this efficacy correlates with increased T cell recruitment. The authors also find that the use of macrophages appears to improve drug delivery into tumors. However, the lack of appropriate controls for the in vivo studies make it unclear whether the use of macrophages improves therapeutic efficacy, and there is little in the way of mechanistic studies. The overall significance of the study is therefore limited.

1. Comment: The study claims that loading macrophages improves efficacy, but the data in Figure 5 and 6 very clearly demonstrate equivalent efficacy between Oxa(IV)@ZnPc and Oxa(IV)@ZnPC@M. Significance is observed when anti-PD-L1 is combined with Oxa(IV)@ZnPC@M, but the control group without macrophages is not included for a comparison.

Response: Thanks for reviewer's comment. In order to verify the necessity of loading nanoparticles into macrophage, the anti-tumor effect and the mechanism of systematic

antitumor immune responses were further conducted on another breast cancer cell model EMT6 model. EMT6 is a representative preclinical model of Triple Negative Breast Cancer (TNBC), similar to 4T1, it was commonly used for immunological evaluation[11, 12]. In the EMT6 model, two control groups including Oxa + anti-PD-L1 and Oxa(IV)@ZnPc (+) + anti-PD-L1 were added. In addition, in order to perform immune-related cells detection more rigorously, we re-conducted the experiments in 4T1 model with the same treatment regimen as before, and use another flow cytometry (CytoFLEX-S, Beckman Coulter) to analyze the sample. Immune-related cells, DCs, cytotoxic T lymphocytes (CTLs) including $CD4^+$ and $CD8^+$ T cells, regulatory T cells (Treg), IFN-γ and TNF-α in $CD8^+$ T cells and tumor-associated macrophage (TAM), were detected by flow cytometry. In the process of detecting the immune-related cells in the 4T1 model, control groups Oxa + anti-PD-L1 and Oxa(IV)@ZnPc (+) + anti-PD-L1 were added as well (see in comment 2).

The EMT6 cells were purchased from FuHeng Cell Cente (Shanghai, China) and maintained in RPMI-1640 medium containing 10% FBS and 1% antibiotics. The bilateral EMT6 tumor model was established similar to that of 4T1.

To investigate the in vivo anti-tumor efficiency on EMT6, Balb/c female mice (5-6 weeks) were subcutaneously injected with 1×10^6 EMT6 cells in the left flank (primary tumor). When the primary tumors reached about 50 mm^3, which was about 6 days after inoculation, 5×10^5 EMT6 cells were intra-tibia injected into the right tibia using a 26-gauge needle under anesthesia. Six days after intra-tibia injection, the mice bearing primary tumors (100-150 mm^3) and bone metastatic tumors were randomly divided into various groups (n=6), and intravenously injected with various formulations including following: (1) PBS (+), (2) free Oxa, (3) Oxa(IV)@ZnPc, (4) Oxa(IV)@ZnPc@M, (5) BMMs + anti-PD-L1, (6) Oxa(IV)@ZnPc (+), (7) Oxa(IV)@ZnPc@M (+), (8) Oxa(IV)@ZnPc@M (+) + anti-PD-L1, (9) Oxa + anti-PD-L1, (10) Oxa(IV)@ZnPc (+) + anti-PD-L1. "(+)" represents laser irradiation. The injection was conducted every two days for three times at an equal platinum dose of 1 mg/kg and ZnPc dose of 0.75 mg/kg (about 1×10^6 cells/mouse). Four hour and 24 h post-injection, the primary tumors were irradiated with a NIR laser (671 nm, 250 mW cm^{-2}) and continued for 10 min, respectively. After the second irradiation, mice were intraperitineally injected with anti-PD-L1 antibody (100 μg/mouse). Tumor size and body weight were monitored every other day. Tumor

volume was measured using calipers and calculated as length × width^2 × 0.5 (**Fig. R21a**).

Compared with PBS (+) group, Oxa and Oxa(IV)@ZnPc showed a slight inhibitory effect on primary and bone metastatic tumors, and BMMs + anti-PD-L1 group exhibited moderate inhibitory effect. Oxa + anti-PD-L1 showed remarkably enhanced effect compared with Oxa, but comparable or a little stronger inhibitory effect than anti-PD-L1, this might be caused by the relative low dosage of Oxa injected. After laser irradiation, the antitumor efficiency of Oxa(IV)@ZnPc (+) and Oxa(IV)@ZnPc@M (+) group on primary tumors were significantly enhanced, with tumor volume cut down by 72.6% and 89.5%, respectively, suggesting remarkable chemo-photodynamic therapeutic effect (**Fig. R21b-c**). Meanwhile, Oxa(IV)@ZnPc@M (+) showed stronger antitumor effect on bone metastatic tumors than Oxa(IV)@ZnPc (+), possibly due to the superior pharmacokinetics and intrinsic tumor accumulation of the cell-based pharmaceutics (**Fig. R21b-c**). However, Oxa(IV)@ZnPc (+) combined with anti-PD-L1 only exhibited comparable effect to Oxa(IV)@ZnPc@M (+) along. Excitingly, when Oxa(IV)@ZnPc@M (+) combined with anti-PD-L1, strongest antitumor effect among all groups on both primary and bone metastatic tumors were observed, with tumor shrinkage rate up to 99.4% and 95.1% respectively compared with PBS (+) group (**Fig. R21b-c**). The anti-tumor effect on EMT6 model was consistent with that of 4T1 model. In addition, mice in the PBS (+) group showed a 28-day life span after inoculation, and mice in the other groups without laser irradiation died within 38 days after inoculation. Oxa(IV)@ZnPc@M (+) showed a significantly prolonged life span which was comparable to Oxa(IV)@ZnPc (+) + anti-PD-L1, with about 50 days after inoculation. In remarked contrast, 80% of mice in the Oxa(IV)@ZnPc@M (+) + anti-PD-L1 group survived over 60 days after inoculation (**Fig. R21d**), and tumors of these mice were cured, demonstrating the prominent effect of the cell-based targeted chemo/photo/immune-therapy system. Besides, no obvious body weight loss was observed after various treatment (**Fig. R21e**), further confirming the good biocompatibility of the cell-based formulation and the cell formulation mediated chemo/photo/immuno-therapy.

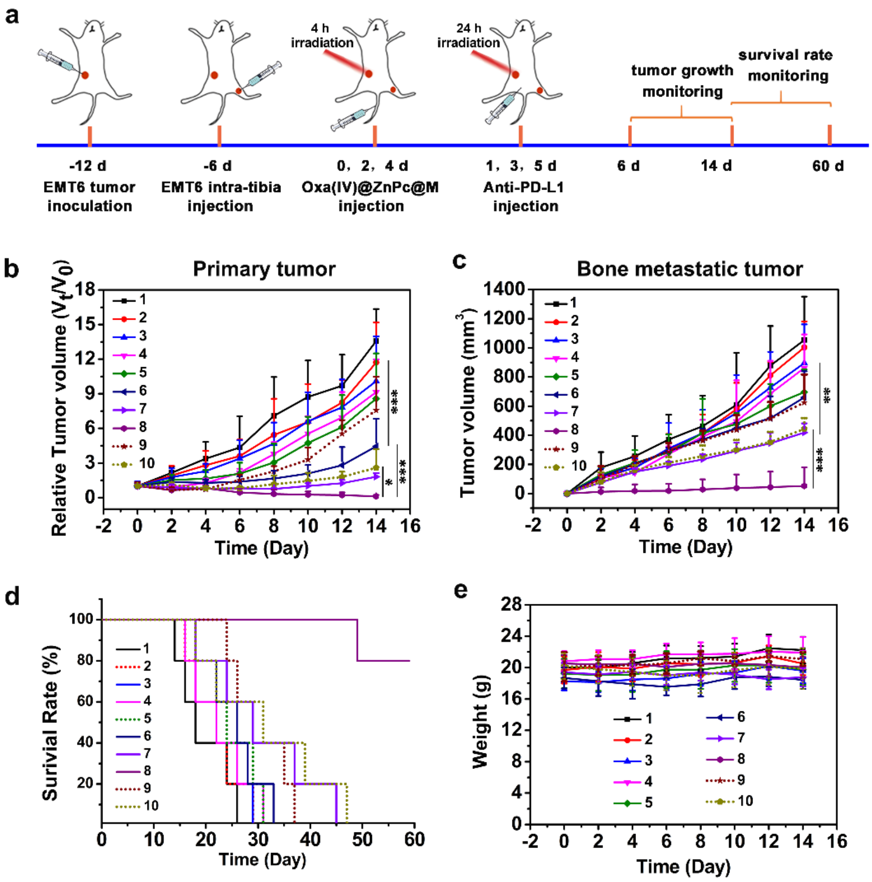

Fig. R21. In vivo antitumor effect of combined chemo/photo/immune-therapy of primary and bone metastatic breast cancer in EMT6 model. (a) Schematic illustration of the experimental design. Tumor growth curves of (b) primary tumors and (c) bone metastatic tumors after various treatments (1: PBS (+); 2: free Oxa; 3: Oxa(IV)@ZnPc; 4: Oxa(IV)@ZnPc@M; 5: BMMs + anti-PD-L1; 6: Oxa(IV)@ZnPc (+); 7: Oxa(IV)@ZnPc@M (+); 8: Oxa(IV)@ZnPc@M (+) + anti-PD-L1; 9: Oxa + anti-PD-L1; 10: Oxa(IV)@ZnPc (+) + anti-PD-L1, "(+)" represent laser irradiation, applied for all the antitumor studies in vivo). (d) Overall survival curves of mice after various treatments. (e) Body weight after various treatments. Values were presented as means ± SD (*p <0.05, **p <0.01, ***p < 0.001).

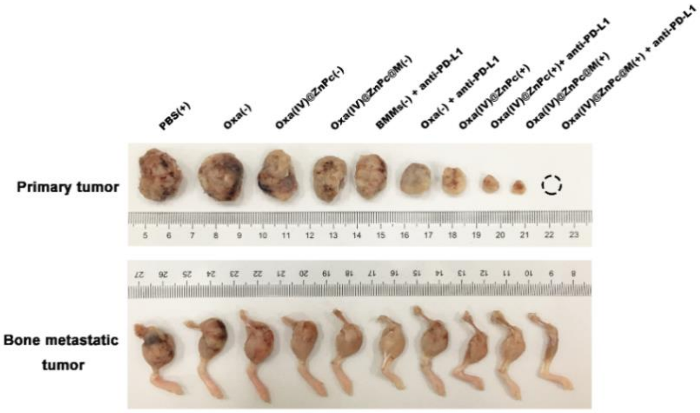

Fig. R22. Representative ex vivo images of primary and bone metastatic tumors after various treatment.

The mechanism of systematic antitumor immune responses were conducted on breast cancer cell model EMT6 model as well. Consistent with the results in 4T1 model, Oxa(IV)@ZnPc@M (+) + anti-PD-L1 treated mice exhibited the highest frequency infiltration of $CD8^+$ and $CD4^+$ T cells, which were 10.4-fold and 8.7-fold higher than the PBS (+) group, respectively. Compared with BMMs + anti-PD-L1 and Oxa(IV)@ZnPc (+), mice treated with Oxa(IV)@ZnPc@M (+) showed increased $CD8^+$ and $CD4^+$ T cells, and it showed comparable effect to nanoparticles combined with anti-PD-L1 (Oxa(IV)@ZnPc (+) + anti-PD-L1 group) (**Fig. R23a-d**). The proportion of Treg ($CD3^+CD4^+Foxp3^+$) of Oxa(IV)@ZnPc@M (+) + anti-PD-L1 group decreased compared with PBS (+) group, and the $CD8^+$ T/Treg ratio and $CD4^+$ T/Treg ratio were dramatically increased compared with other groups (**Fig. R23e-h**). Except that a higher population of infiltrated $CD8^+$ T cells, interferon-γ (IFN-γ)-positive $CD3^+CD8^+IFN\text{-}\gamma^+$ T cells and TNF-α-positive $CD3^+CD8^+$ $TNF\text{-}\alpha^+$ T cells were also important to the therapeutic effect. Notably, the levels of $CD3^+CD8^+IFN\text{-}\gamma^+$ T cells and $CD3^+CD8^+$ $TNF\text{-}\alpha^+$ T cells in tumor were significantly elevated by Oxa(IV)@ZnPc@M (+) + anti-PD-L1 treatment (**Fig. R24**). All of these results reveled that Oxa(IV)@ZnPc@M (+) treatment or combined with anti-PD-L1 could significantly promote tumor-infiltration of antitumor lymphocyte.

As demonstrated that Oxa(IV)@ZnPc loading could polarize BMMs to anti-tumor M1 phenotype, and the macrophages could retain the M1 phenotype in vivo, we further evaluated whether Oxa(IV)@ZnPc@M could re-educate TAM from tumor-promoting M2 phenotype toward tumor-killing M1 phenotype by flow cytometry, where M1 macrophages were denoted as $CD86^+CD206^-F4/80^+$ cells, and M2 macrophages were denoted as $CD86^-CD206^+F4/80^+$ cells. As show in **Fig. R25**, free Oxa and Oxa(IV)@ZnPc exhibited increased percentage of M1 macrophages and decreased percentage of M2 macrophages compared with PBS group, which might be attributed to the effect Oxa. In comparison with free Oxa and Oxa(IV)@ZnPc, the percentage of M1 macrophages in Oxa(IV)@ZnPc@M group was markedly increased, and the percentage of M2 macrophages was significantly decreased, accompanied by remarkably elevated M1/M2 ratio, which was 1.66-fold of Oxa(IV)@ZnPc and 2.45-fold of free Oxa. The result might be ascribed to the combination effect of Oxa and the M1 phenotype of Oxa(IV)@ZnPc@M that can produce proinflammatory cytokines to re-educate TAM toward

tumor-killing M1 phenotypes, enhancing the antitumor effect.

All of these results indicated the cell-based system had higher effect to elicit the immune system by the combined chemo/photo/immuo-therapy than that of nano-based system. This part of experiment were added to the revised manuscript, the related pictures have been reformatted in the revised manuscript, and all the changes were highlighted in red.

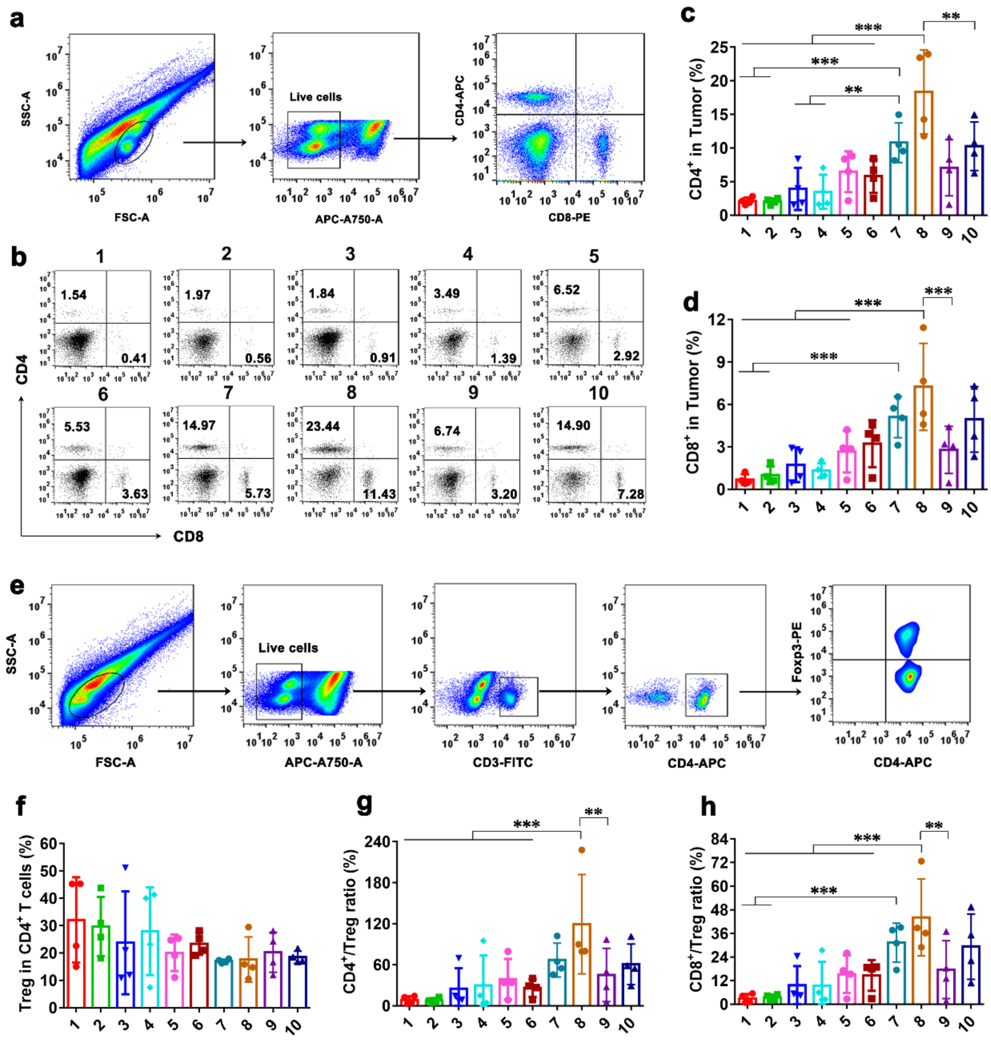

Fig. R23. (a) The gating strategy and (b) the representative flow cytometric plots of tumor-infiltrating $CD4^+$ T cells and $CD8^+$ T cells in EMT6 model. Quantitation of the percent of tumor-infiltrating (c) $CD4^+$ T cells and (d) $CD8^+$ T cell in tumors analyzed on day 6 after various treatment (1: PBS (+); 2: free Oxa; 3: Oxa(IV)@ZnPc; 4: Oxa(IV)@ZnPc@M; 5: BMMs + anti-PD-L1; 6: Oxa(IV)@ZnPc (+); 7: Oxa(IV)@ZnPc@M (+); 8: Oxa(IV)@ZnPc@M (+) + anti-PD-L1, 9: Oxa + anti-PD-L1; 10: Oxa(IV)@ZnPc (+) + anti-PD-L1, “(+)” represent with laser irradiation). (e) The gating strategy of flow cytometric analysis of Treg ($CD3^+CD4^+Foxp3^+$). (f) Quantitation of the percent of Treg in tumors analyzed on day 6 after various treatment. Quantitation of the percent of (g) intratumoral ratio of $CD4^+$ T cells to Treg and (h) $CD8^+$ T cells to Treg. **p* <0.05, ***p* <0.01, ****p* < 0.001 (n=4).

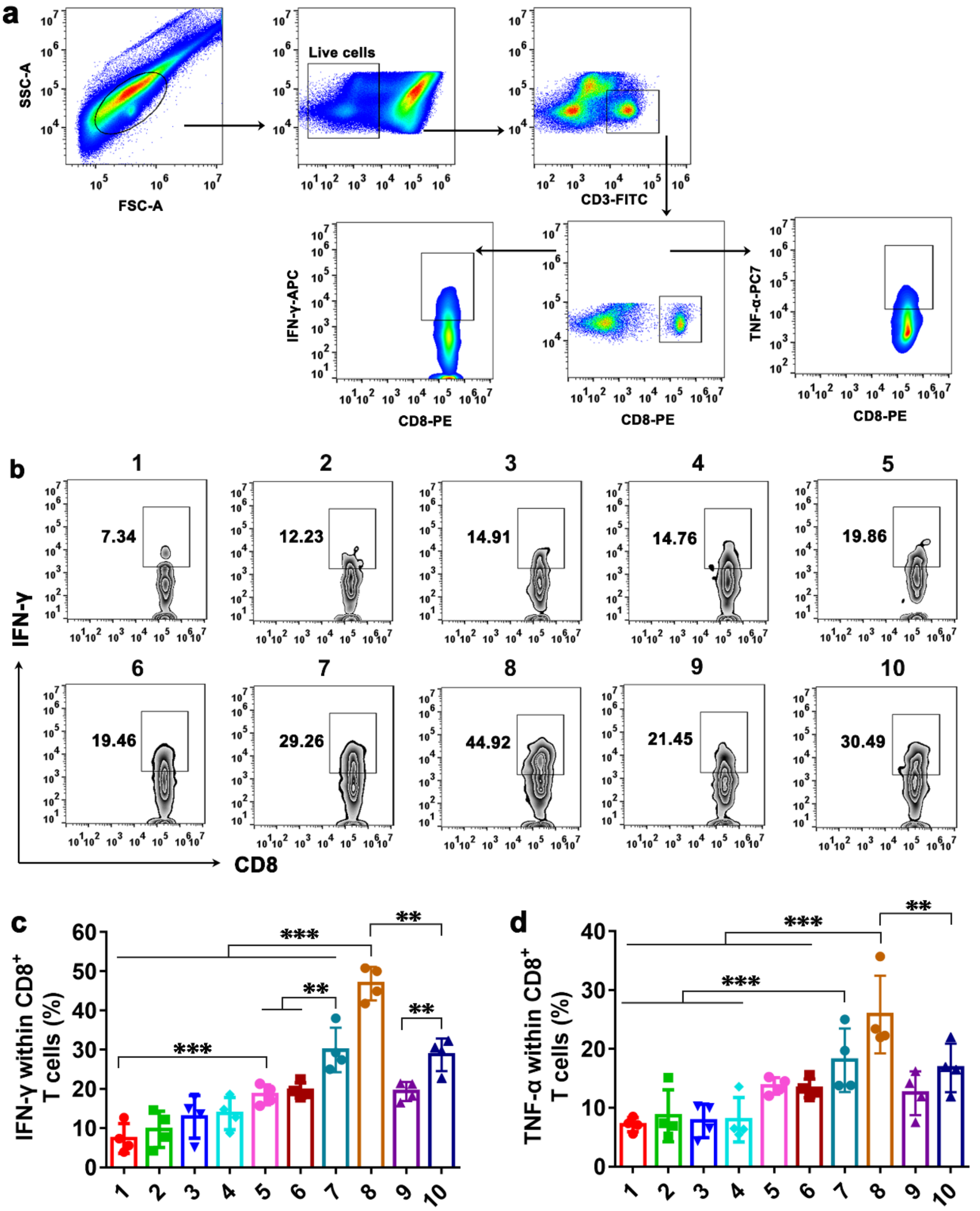

Fig. R24. (a) The gating strategy and (b) the representative flow cytometric plots of IFN-γ within CD8$^+$ T cells in EMT6 model. Quantitation of the percent of (c) IFN-γ within CD8$^+$ T cells and TNF-α within CD8$^+$ T cells in tumors analyzed on day 6 after various treatment (1: PBS (+); 2: free Oxa; 3: Oxa(IV)@ZnPc; 4: Oxa(IV)@ZnPc@M; 5: BMMs + anti-PD-L1; 6: Oxa(IV)@ZnPc (+); 7: Oxa(IV)@ZnPc@M (+); 8: Oxa(IV)@ZnPc@M (+) + anti-PD-L1, 9: Oxa + anti-PD-L1; 10: Oxa(IV)@ZnPc (+) + anti-PD-L1, "(+)" represent with laser irradiation). *p <0.05, **p <0.01, ***p < 0.001 (n=4).

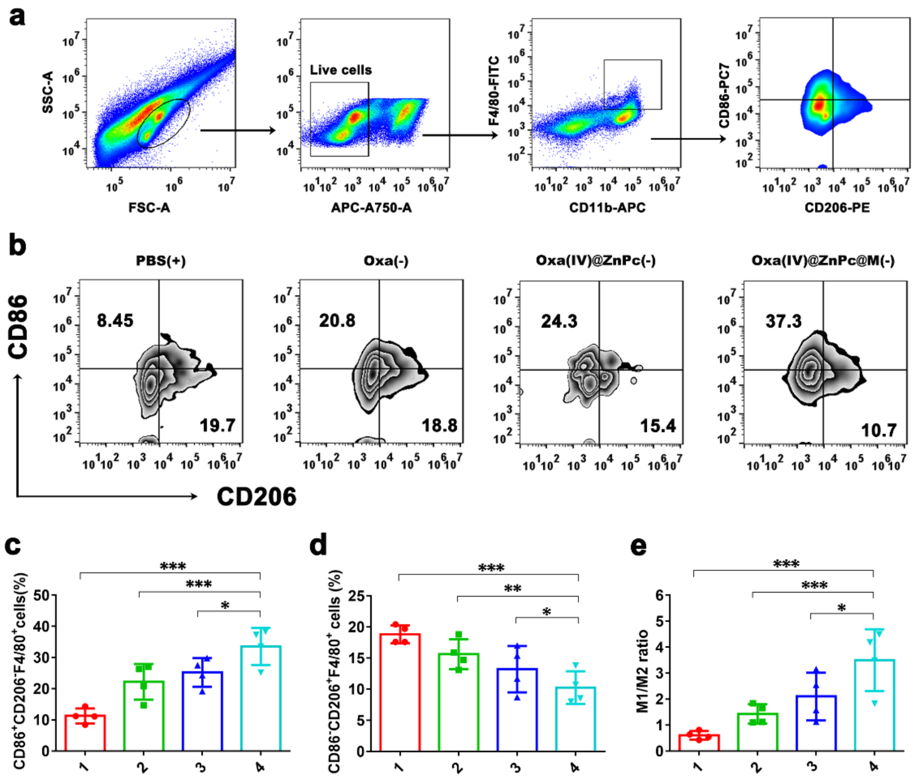

Fig. R25. (a) The gating strategy and (b) the representative flow cytometric plots of M1 macrophages ($CD86^{+}CD206^{-}F4/80^{+}$) and M2 macrophages ($CD86^{-}CD206^{+}F4/80^{+}$) in EMT6 model. Quantitation of the percent of (c) M1 macrophages and (d) M2 macrophages analyzed on day 6 after various treatment (1: PBS (+); 2: free Oxa; 3: Oxa(IV)@ZnPc; 4: Oxa(IV)@ZnPc@M). (e) Quantitation of M1 macrophages to M2 macrophages. **p* <0.05, ***p* <0.01, ****p* < 0.001 (n=4).

2. Comment: Similarly, most of the immune observations require the presence of anti-PD-L1, but the only control group includes unloaded macrophages with anti-PD-L1. The authors do not demonstrate that Oxa(IV)@ZnPC@M + anti-PD-L1 is more efficacious than Oxa + anti-PD-L1. Given the known immunogenic potential of Oxa, this is a critical control group to include in the studies.

Response: Thanks for reviewer's comment. In order to perform immune-related cells detection more rigorously, we re-conducted the experiments in 4T1 model with the same treatment regimen as before, and use another flow cytometry (CytoFLEX-S, Beckman Coulter) to analyze the sample. Immune-related cells, DCs, cytotoxic T lymphocytes (CTLs) including $CD4^{+}$ and $CD8^{+}$ T cells, regulatory T cells (Treg), IFN-γ and TNF-α in $CD8^{+}$ T cells, were detected by flow cytometry. In the process of detecting the immune-related cells in the 4T1 model, in addition to the previous 8 group, control groups Oxa + anti-PD-L1 and

Oxa(IV)@ZnPc (+) + anti-PD-L1 were added.

The level of mature DCs in the mice lymph nodes after various treatments were further evaluated. Briefly, the mice bearing bilateral 4T1 tumors were randomly assigned into various groups (n=4), and received the same treatment regimen as the anti-tumor experiment. The next day after the first treatment, the tumor draining lymph node from each treatment were harvested, ground up and then passed through 200 μm and 70 μm filter to obtain single-cell suspensions. These cells were stained with CD16/32, eFluor^{TM} 450, anti-CD11c-FITC, anti-CD80-PE and anti-CD86-PC7 antibodies according to the manufacturer's instructions, then analyzed by flow cytometry (CytoFLEX-S, Beckman Coulter) to delineate mature DC cells. The matured DCs were denoted as CD11c$^+$CD80$^+$CD86$^+$ cells. According to the flow cytometry result, Oxa(IV)@ZnPc (+) + anti-PD-L1 treatment only showed comparable effect to Oxa(IV)@ZnPc@M (+), while Oxa(IV)@ZnPc@M (+) + anti-PD-L1 treatment showed the best effect on promoting DCs maturation, with the expression of CD80 and CD86 were dramatically up-regulated compared with other treatment groups (**Fig. R26**), indicating the best effect on maturating and activating of DCs, which showed great potential to stimulate the subsequent tumor-specific immune response. This section of experiment was added to the revised manuscript, and all the changes were highlighted in red.

Other immune related cells were detected as well. As shown in **Fig. R27a-d**, Mice treated with Oxa(IV)@ZnPc@M (+) + anti-PD-L1 exhibited the highest effect in promoting intratumoral infiltration of CD8$^+$ and CD4$^+$ T cells , where 9.99% CD8$^+$ T cells and 19.0% CD4$^+$ T cells were induced, which were 5.9-fold and 6.9-fold higher than the PBS (+) group, respectively. Mice treated with BMMs + anti-PD-L1 and Oxa(IV)@ZnPc (+) led to moderate increase of CD8$^+$ and CD4$^+$ T cells, by contrast, mice treated with Oxa(IV)@ZnPc@M (+) showed a lightly increased effect, which was comparable to nanoparticles combined with anti-PD-L1 group (Oxa(IV)@ZnPc (+) + anti-PD-L1 group). The populations of Treg (CD3$^+$CD4$^+$Foxp3$^+$) of Oxa(IV)@ZnPc@M (+) + anti-PD-L1 group decreased compared with PBS (+) group (**Fig. R27f**), and the CD8$^+$T/Treg ratio and CD4$^+$T/Treg ratio were dramatically increased compared with other groups (**Fig. R27g-h**). Except that a higher population of infiltrated CD8$^+$ T cells, IFN-γ and TNF-α within CD8$^+$ T cells were also important to the therapeutic effect. Notably, the levels of IFN-γ$^+$ and TNF-α$^+$ in CD8$^+$ T cells in tumor was

significantly elevated by Oxa(IV)@ZnPc@M (+) + anti-PD-L1 treatment (**Fig. R28**). All of these results reveled that Oxa(IV)@ZnPc@M (+) treatment or combined with anti-PD-L1 could significantly promote tumor-infiltration of antitumor lymphocyte.

All of these results indicated the cell-based system had higher effect to elicit the immune system by the combined chemo/photo/immuo-therapy than that of nano-based system. This part of experiment were added to the revised manuscript, the related pictures have been reformatted in the revised manuscript, and all the changes were highlighted in red.

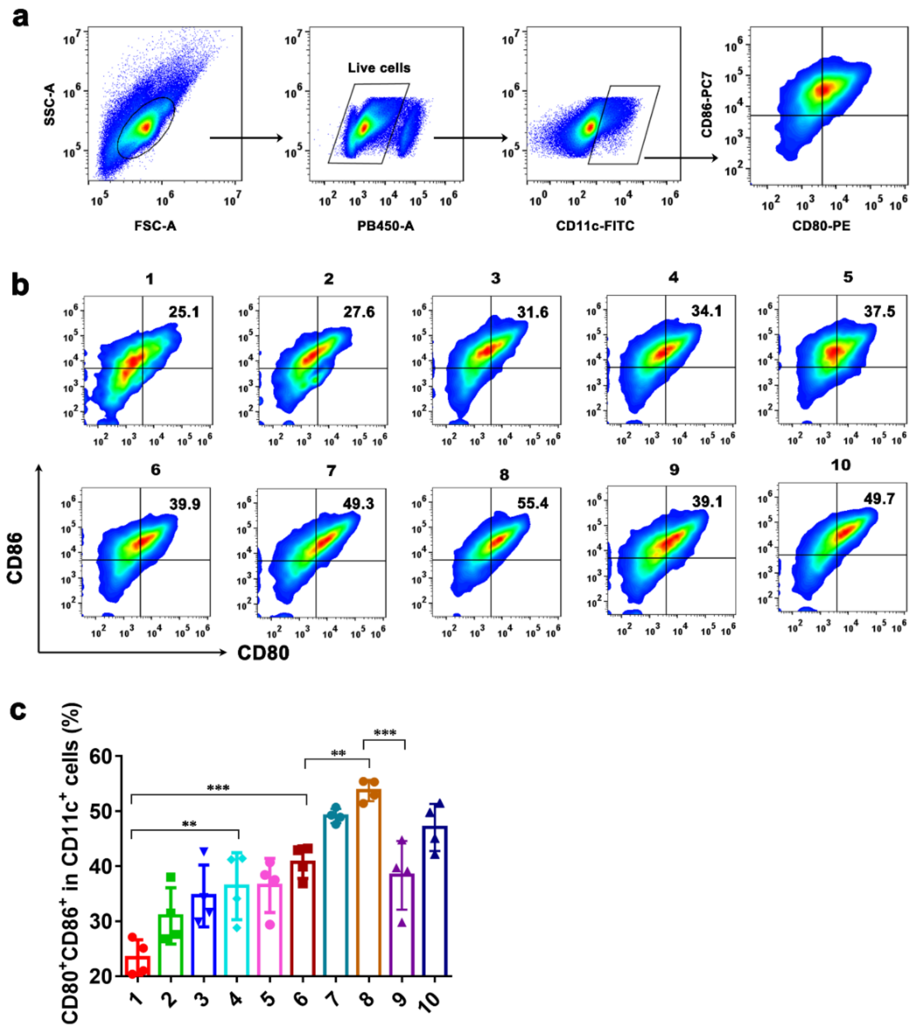

Fig. R26. (a) The gating strategy and (b) the representative flow cytometric analysis of DCs maturation in tumor-draining lymph nodes on day 2 after various treatment (1: PBS (+); 2: free Oxa; 3: Oxa(IV)@ZnPc; 4: Oxa(IV)@ZnPc@M; 5: BMMs + anti-PD-L1; 6: Oxa(IV)@ZnPc (+); 7: Oxa(IV)@ZnPc@M (+); 8: Oxa(IV)@ZnPc@M (+) + anti-PD-L1, 9: Oxa + anti-PD-L1; 10: Oxa(IV)@ZnPc (+) + anti-PD-L1, “(+)” represent with laser irradiation). (c) Quantitation of the percent of mature DCs ($CD11c^{+}CD80^{+}CD86^{+}$) in tumor-draining lymph nodes on day 2 after various treatment. *p <0.05, **p <0.01, ***p < 0.001 (n=4).

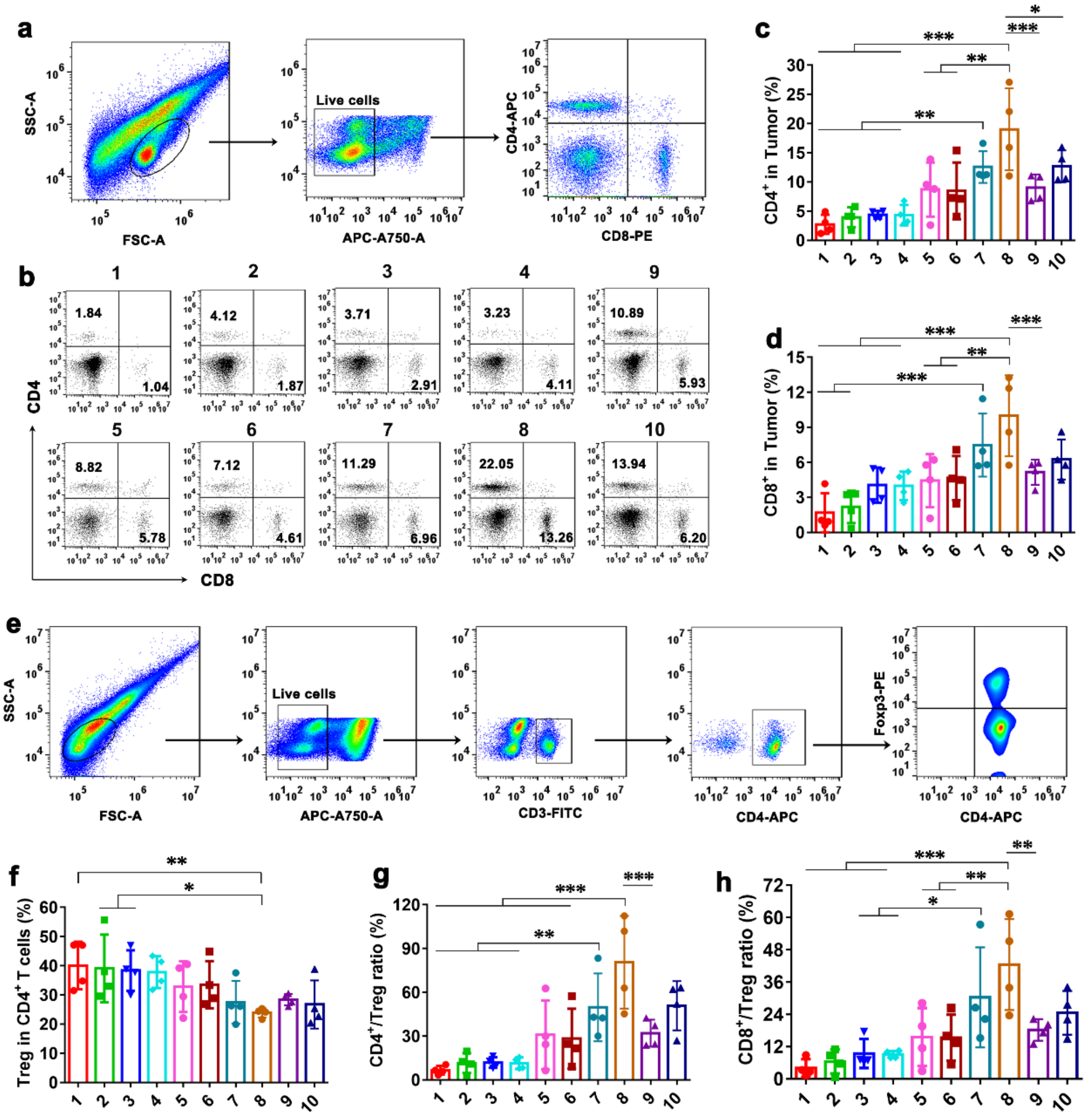

Fig. R27. (a) The gating strategy and (b) the representative flow cytometric plots of tumor-infiltrating $CD4^+$ T cells and $CD8^+$ T cells. Quantitation of the percent of tumor-infiltrating (c) $CD4^+$T cells and (d) $CD8^+$T cell in tumors analyzed on day 6 after various treatment (1: PBS (+); 2: free Oxa; 3: Oxa(IV)@ZnPc; 4: Oxa(IV)@ZnPc@M; 5: BMMs + anti-PD-L1; 6: Oxa(IV)@ZnPc (+); 7: Oxa(IV)@ZnPc@M (+); 8: Oxa(IV)@ZnPc@M (+) + anti-PD-L1, 9: Oxa + anti-PD-L1; 10: Oxa(IV)@ZnPc (+) + anti-PD-L1, “(+)” represent with laser irradiation). (e) The gating strategy of flow cytometric analysis of Treg ($CD3^+CD4^+Foxp3^+$). (f) Quantitation of the percent of Treg in tumors analyzed on day 6 after various treatment. Quantitation of the percent of (g) intratumoral ratio of $CD4^+$ T cells to Treg and (h) $CD8^+$ T cells to Treg. *p <0.05, **p <0.01, ***p < 0.001 (n=4).

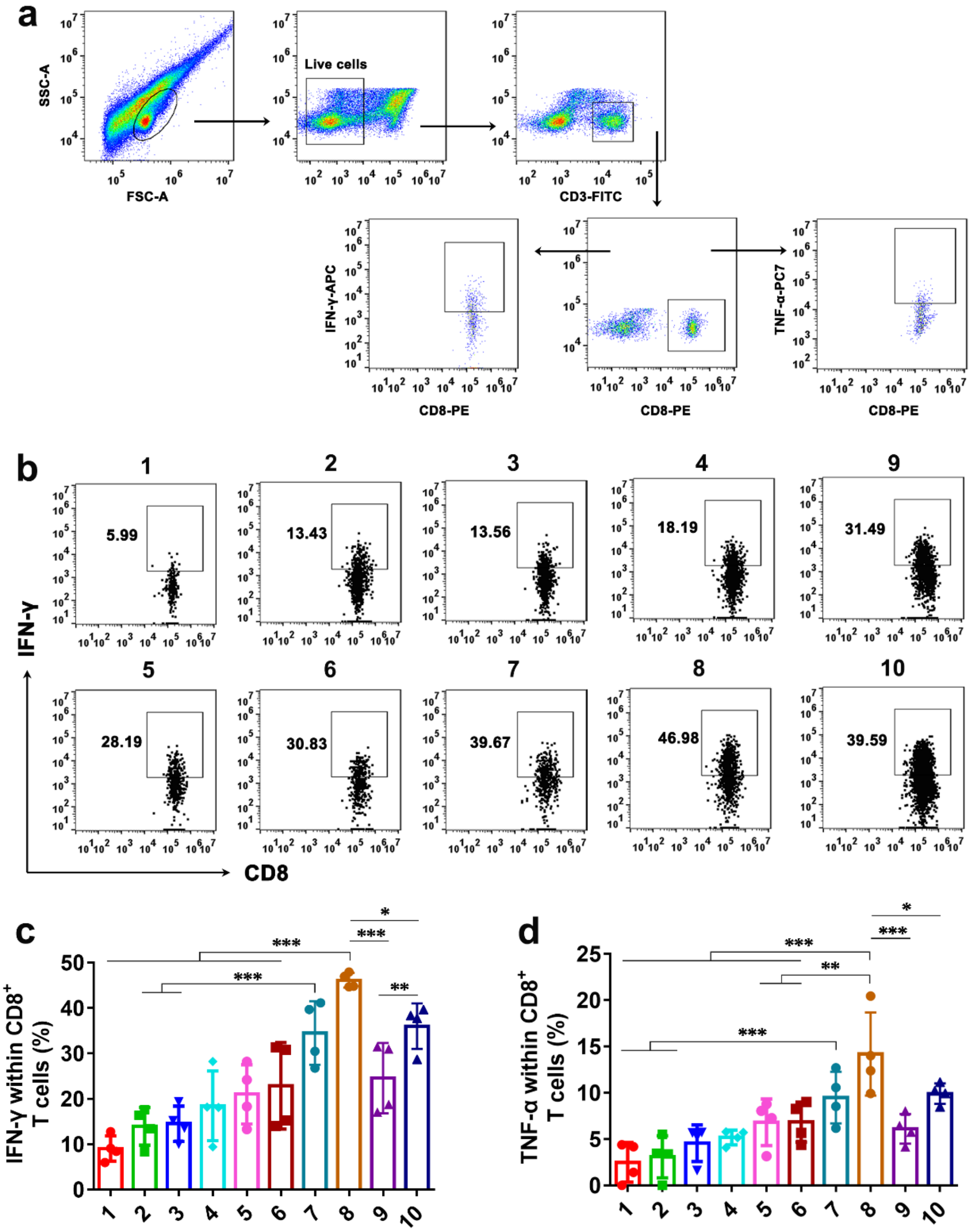

Fig. R28. (a) The gating strategy and (b) the representative flow cytometric plots of IFN-γ within $CD8^+$ T cells. Quantitation of the percent of (c) IFN-γ within $CD8^+$ T cells and TNF-α within $CD8^+$ T cells in tumors analyzed on day 6 after various treatment (1: PBS (+); 2: free Oxa; 3: Oxa(IV)@ZnPc; 4: Oxa(IV)@ZnPc@M; 5: BMMs + anti-PD-L1; 6: Oxa(IV)@ZnPc (+); 7: Oxa(IV)@ZnPc@M (+); 8: Oxa(IV)@ZnPc@M (+) + anti-PD-L1, 9: Oxa + anti-PD-L1; 10: Oxa(IV)@ZnPc (+) + anti-PD-L1, "(+)" represent with laser irradiation). *p <0.05, **p <0.01, ***p < 0.001 (n=4).

The supplement experiment in EMT6 and 4T1 model all showed that Oxa + anti-PD-L1 showed comparable effect to anti-PD-L1, while Oxa(IV)@ZnPc (+) + anti-PD-L1 showed

comparable effect to Oxa(IV)@ZnPc@M (+) (without adding anti-PD-L1), thus no matter adding or not adding the two control group will not affect the conclusion of the full paper. Considering the unity of the full text and the readability of the paper, though 10 group of experiments in the detection of immune-related cells in 4T1 model were conducted, we only added the previous 8 group of data of the 4T1 model to the text. However, in the EMT6 model, 10 group of data in the evaluation of anti-tumor efficacy and immune mechanism in vivo were added to the revised manuscript.

3. Comment: There is no demonstration that loaded macrophages are entering the tumors. It is possible that the cells are releasing the cargo from other sites.

Response: Thanks for reviewer's comment. We further performed in vivo fluorescence imaging and flow cytometry using cell membrane dye DiR and DiD labelled macrophages respectively to address this question. In order to eliminate the interference of the fluorescent dye ZnPc, which is non-toxic under dark, we use Oxa(IV)@Lip (lipid coated but without ZnPc loading) to replace Oxa(IV)@ZnPc for drug loading. The DiR or DiD labelled blank BMMs and drug loaded BMMs were named as BMMs-DiR, BMMs-DiD, Oxa(IV)@Lip@M-DiR and Oxa(IV)@Lip@M-DiD.

For in vivo fluorescence imaging study, mice bearing 4T1 primary and bone metastatic tumors were intravenously injected with free DiR, BMMs-DiR and Oxa(IV)@Lip@M-DiR with an equivalent DiR dose. At 24 h post-injection, hearts, livers, spleens, lungs, kidneys, primary tumors, right tumor-bearing hindlimbs and left hindlimbs were excised for ex vivo imaging using an in vivo imaging system (IVIS) (Lumina XRMS Series III, PerkinElmer, USA). Compared with free DiR, both BMMs-DiR and Oxa(IV)@Lip@M-DiR showed obvious fluorescence signal in both primary tumor and bone metastatic tumor, indicating that both blank and loaded macrophages could entering the tumors (**Fig. R29**).

For in vivo flow cytometry study, mice bearing 4T1 tumors were intravenously injected with saline, BMMs-DiD and Oxa(IV)@Lip@M-DiD. At 8 h post-injection, tumors were harvested, cut into small pieces and ground up, then passed through 200 μm and 70 μm filter to obtain single-cell suspensions, and then analyzed by flow cytometry (CytoFLEX-S, Beckman Coulter). Compared with Saline group, the population of DiD positive cells of

BMMs-DiD and Oxa(IV)@Lip@M-DiD group in the tumor tissue were significantly increased, further confirming that the loaded macrophages can entering the tumors (**Fig. R30**).

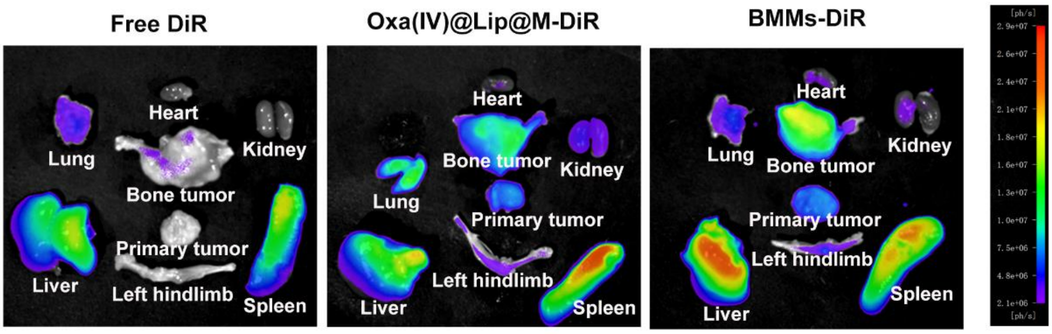

Fig. R29. Ex vivo fluorescence imaging of DiR, BMMs-DiR and Oxa(IV)@Lip@M-DiR in primary tumors and bone metastatic tumors in 4T1 tumor-bearing mice.

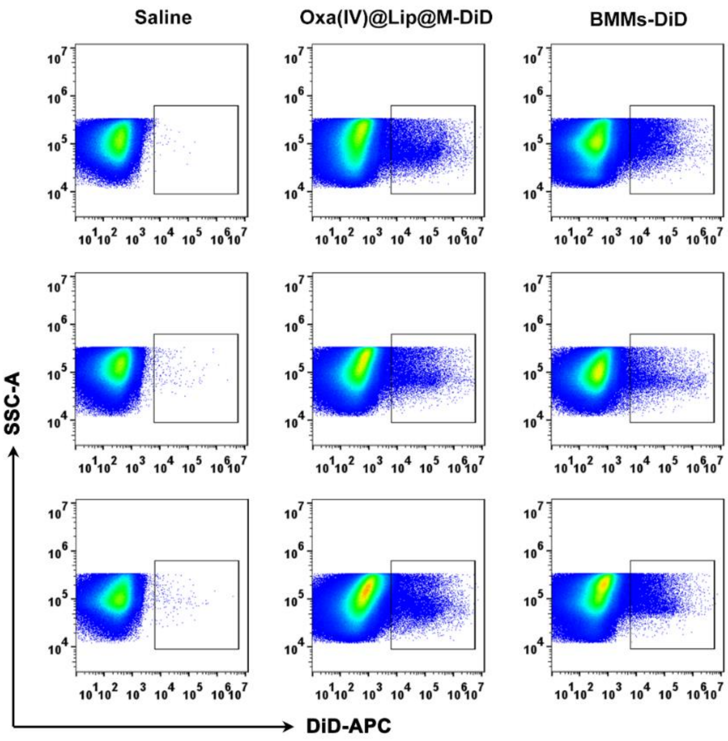

Fig. R30. Flow cytometry analysis of the DiD positive cells of Saline, BMMs-DiD and Oxa(IV)@Lip@M-DiD (n=3) in tumor tissue after intravenous injection.

4. Comment: There are no in vivo mechanistic studies performed, only in vitro observations that are claimed to represent mechanism of action. For example, M1 polarization is said to be important for efficacy, but it is not clear if the macrophages retain this phenotype in vivo. A role for HMGB1, CRT, etc are also not validated.

Response: Thanks for reviewer's comment. Whether the macrophages can retain the M1 polarization in vivo was conducted by detecting the surface marker (M1 maker CD80 and CD86) of macrophages using flow cytometry (CytoFLEX-S, Beckman Coulter). Similar to the response of **comment 3**, DiD labelled macrophages BMMs-DiD, and Oxa(IV)@Lip@M-DiD were prepared. In addition, typical LPS (500 ng/mL) and IFN-γ (40 ng/mL) treated M1 BMMs were served as positive control. Briefly, mice bearing 4T1 tumors were intravenously injected with saline, BMMs-DiD, M1-DiD and Oxa(IV)@Lip@M-DiD. At 8 h post-injection, tumors were harvested, cut into small pieces and ground up, then passed through 200 μm and 70 μm filter to obtain single-cell suspensions. The tumor cells were first incubated with anti-mouse CD16/32 antibody to prevent non-specific staining, then it was further stained with anti-CD80-PE and anti-CD86-PC7 according to manufacturer's instructions, and then analyzed by flow cytometry (CytoFLEX-S, Beckman Coulter). As shown in **Fig. R31**, the population of $CD80^+CD86^+$ cells in DiD positive macrophages of Oxa(IV)@Lip@M were counted to be about 70%, which was similar to that of LPS treated M1 cells, while blank BMMs only counted to be about 37%, indicating that the drug loaded macrophages could retain its M1 phenotype in the tumor tissue.

In order to verify the immunogenic cell death (ICD) effect in vivo, we performed immunofluorescence staining for CRT (**Supplementary Fig. 23a**) and immunohistochemical staining for HMGB1 (**Supplementary Fig. 23b**) on 4T1 primary tumors after mice received various treatments. To further verify the role of HMGB1 and CRT in vivo, the level of mature DCs in the mice lymph nodes after various treatments were further evaluated.

Briefly, the mice bearing bilateral 4T1 tumors were randomly assigned into various groups (n=4), and received the same treatment regimen as the anti-tumor experiment. The next day after the first treatment, the tumor draining lymph node from each treatment were harvested, ground up and then passed through 200 μm and 70 μm filter to obtain single-cell suspensions. These cells were stained with CD16/32, eFluor^{TM} 450, anti-CD11c-FITC, anti-CD80-PE and anti-CD86-PC7 antibodies according to the manufacturer's instructions, then analyzed by flow cytometry (CytoFLEX-S, Beckman Coulter) to delineate mature DC cells. The matured DCs were denoted as $CD11c^+CD80^+CD86^+$ cells. According to the flow cytometry result, the Oxa(IV)@ZnPc@M (+) + anti-PD-L1 treatment showed the best effect on promoting DCs

maturation, with the expression of CD80 and CD86 were dramatically up-regulated compared with other treatment groups (**Fig. R32-33**), indicating the best effect on maturating and activating of DCs, which showed great potential to stimulate the subsequent tumor-specific immune response. This section of experiment was added to the revised manuscript, and all the changes were highlighted in red.

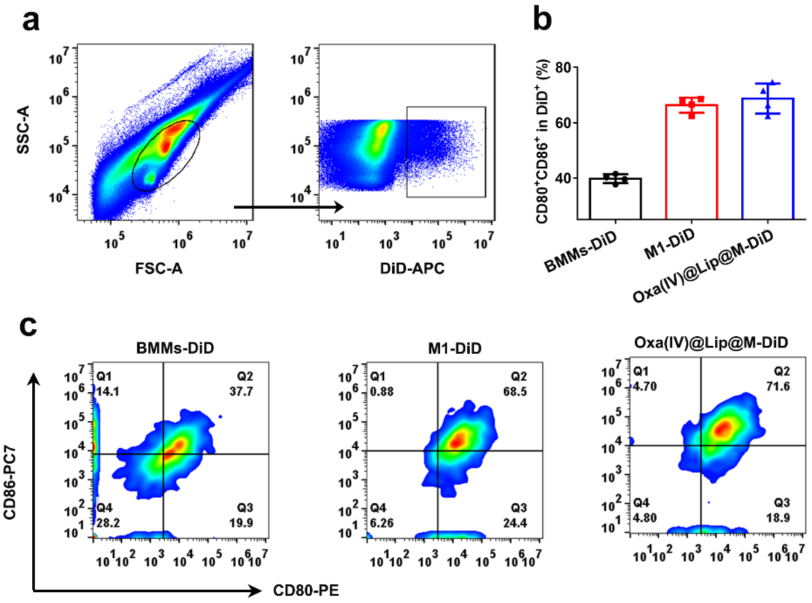

Fig. R31. (a) The gating strategy of macrophage polarization phenotype in tumor tissue. (b) Quantitative analysis of $CD80^+CD86^+$ cells in the DiD positive macrophages. (c) Representative flow cytometric analysis of the expression of M1 marker (CD80, CD86).

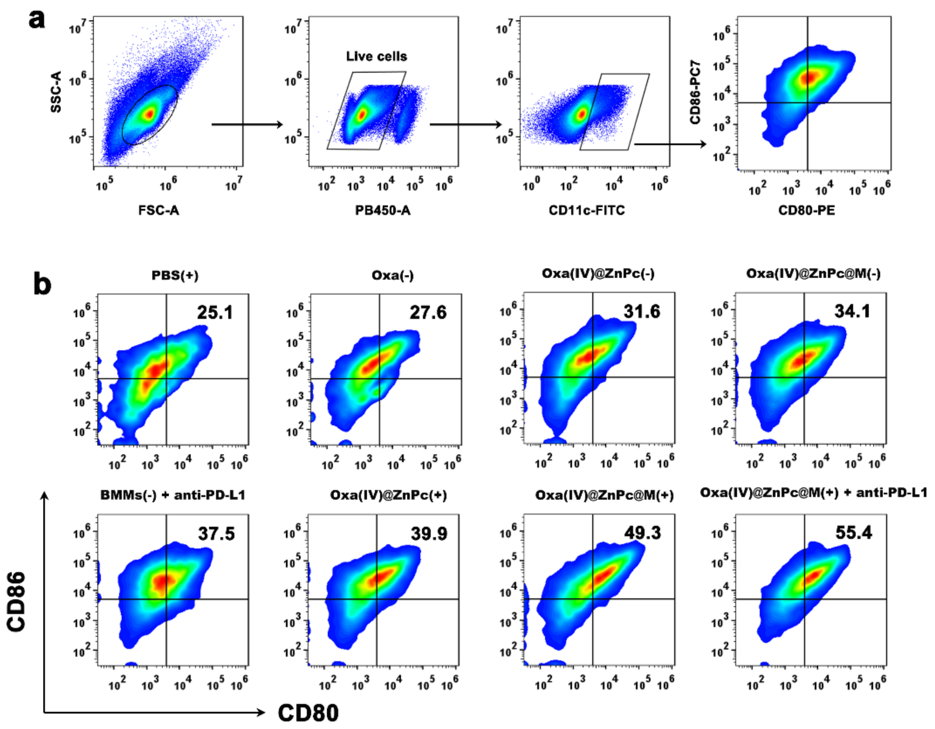

Fig. R32. (a) The gating strategy and (b) the representative flow cytometric analysis of DCs maturation in tumor-draining lymph nodes on day 2 after various treatment.

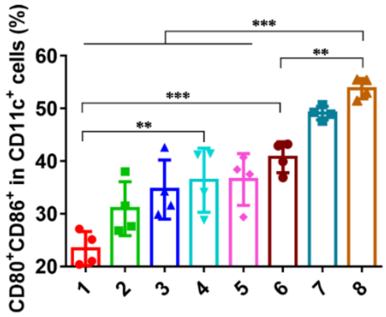

Fig. R33 Quantitation of the percent of mature DCs (CD11c+CD80+CD86+) in tumor-draining lymph nodes on day 2 after various treatment (1: PBS (+); 2: free Oxa; 3: Oxa(IV)@ZnPc; 4: Oxa(IV)@ZnPc@M; 5: BMMs + anti-PD-L1; 6: Oxa(IV)@ZnPc (+); 7: Oxa(IV)@ZnPc@M (+); 8: Oxa(IV)@ZnPc@M (+) + anti-PD-L1, "(+)" represent with laser irradiation). *p <0.05, **p <0.01, ***p < 0.001 (n=4).

Reviewer #4 (Remarks to the Author): **with expertise in nanoparticle and immunotherapy**

In this manuscript, Huang and co-authors reported an engineered macrophages carrying nanomedicine containing oxaliplatin and photosensitizer for chemo/photo/immune-therapy of primary and bone metastatic breast cancer. They found that bone marrow-derived macrophages

could effectively load Oxa(IV)@ZnPc nanoparticles and elongate the blood cirtulation time and have tumor targeting ability. In addition, they found that the cargos inside polarize macrophages to anti-tumor M1 phenotype. The engineered macrophages can effectively inhibit both primary and metastatic tumor growth, and activate immune-memory effects. This is an interesting study and the conclusions are well supported by the data. I have the following concerns for this manuscript:

1. Comment: What's the mechanism of Oxa(IV)@ZnPc loading for polarizing bone marrow-derived macrophages toward the proinflammation antitumor M1 phenotypes? Besides cytokine secretion, macrophage surface markers should be measured to further confirm the phenotype.

Response: Thanks for reviewer's comment. We performed supplementary experiment to address this question. Briefly, BMMs were seeded in 6-well plates and treated with Oxa(IV)@ZnPc for 2 h, then cells were washed, after further incubation for 12 h, all cells were harvested, stained with anti-CD80-FITC and anti-CD86-PE and then analyzed by flow cytometry (Guava EasyCyte 6-2L, Merck Millpore). Typical LPS treated M1 macrophages and IL-4 treated M2 macrophages were served as control, and the expression of iNOS (M1 maker) and Arg-1 (M2 maker) were verified by western blot **(Fig. R34)**. As shown in **Fig. R35,** the population of $CD80^{+}CD86^{+}$ cells treated with Oxa(IV)@ZnPc showed similar to LPS treated M1 cells. Together with the cytokine secretion determination experiment, the result further confirming the polarization of BMMs to proinflammation M1 phenotypes after Oxa(IV)@ZnPc loading.

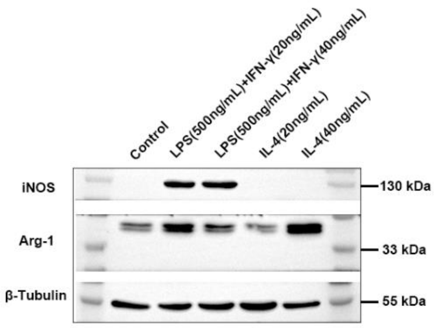

Fig. R34. Western blot detection of iNOS (M1 maker) and Arg-1(M2 Maker) of BMMs after various treatment for 24 h.

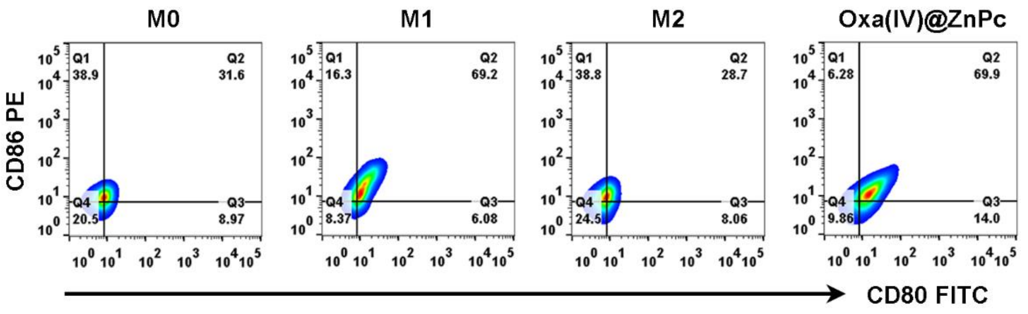

Fig. R35. Flow cytometric analysis of the expression of M1 marker (CD80, CD86) on BMMs after loading Oxa(IV)@ZnPc.

In order to further explore the reason for macrophage polarization, the effect of free ZnPc, ZnPc@Lip (ZnPc loaded lipsome), SPP@ZnPc (control nanoparticle, using low-toxic sodium pyrophosphate to replace Oxa prodrug), and free Oxa were further compared using the same experimental procedure. Free ZnPc, ZnPc@Lip and SPP@ZnPc showed similar $CD80^{+}CD86^{+}$ cells to IL-4 treated M2 cells, while free Oxa with concentration 100 μM-2.5 μM showed similar $CD80^{+}CD86^{+}$ cells to LPS treated M1 cells (**Fig. R36**), indicating that the polarization of macrophages may attributed to the released Oxa.

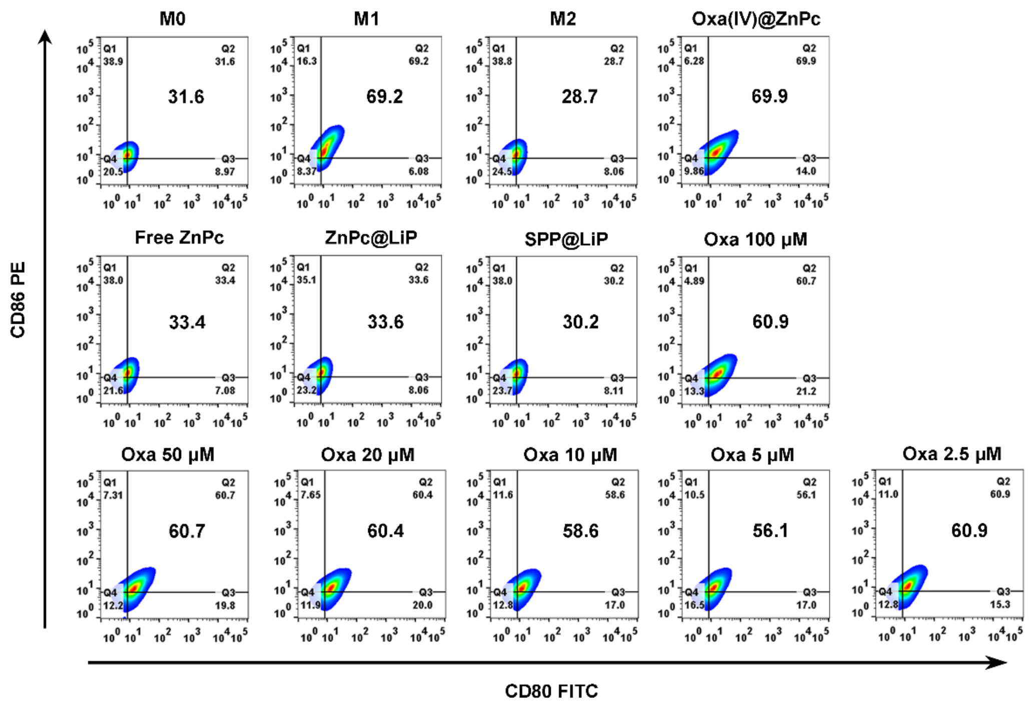

Fig. R36. Flow cytometric analysis of the expression of M1 marker (CD80, CD86) on BMMs after different treatment.

2. Comment: In Fig.1j, what does the white arrows and the blank dots represent? Does

Oxa(IV)@ZnPc still exist as lipid nanoparticles inside the macrophages?

Response: Thanks for reviewer's comment. The white arrow in Fig. 1j was intended to indicate the gradual enlargement of the part where the nanoparticles exist. We are not sure what the blank dots is. The solid black dots represent lipid nanoparticles Oxa(IV)@ZnPc. We collected the BMMs for TEM sample preparation after incubation with Oxa(IV)@ZnPc for 2 h, according to the result of TEM image, Oxa(IV)@ZnPc existed as lipid nanoparticles inside the macrophages at least during the drug loading period.

3. Comment: Characterization on Oxa(IV)-OH and Oxa(IV)-COOH is not clearly enough. There are some peaks unlabeled in Fig.S2 and Fig.S3. Please speculate the peaks in Fig.S4 and Fig.S5.

Response: Thanks for reviewer's comment. The peaks at chemical shift 3.3 ppm and 2.5 ppm of the NMR spectrum in **Fig. S2** and **Fig. S3** were assigned to the peak of solvent DMSO-d6, thus it was unlabeled, other characteristic peaks of Oxa(IV)-OH and Oxa(IV)-COOH were labeled in the ^{1}H NMR spectrum. Some unlabeled peaks in the structure of Oxa(IV)-OH and Oxa(IV)-COOH in **Fig. S2** and **Fig. S3** were labeled in the revised manuscript. In addition, the ESI-MS peaks of Oxa(IV)-OH and Oxa(IV)-COOH in **Fig. S4** and **Fig. S5** were speculated in the revised manuscript. Thank you again for your kind remind.

4. Comment: What does the control NPs (SPP@ZnPc and Cal@LiP) represents? Please give size, drug loading content and other characterizations on these nanoparticles.

Response: Thanks for reviewer's comment. Sodium pyrophosphate ($Na_4P_2O_7 \cdot 10H_2O$, SPP, >99.0%) and calcein are two kinds of compounds with coordination groups that can coordinate with metal ions to form coordination nanoparticles similar to Oxa(IV)@DOPA. Lipid coated and ZnPc loaded SPP@ZnPc were prepared similar to Oxa(IV)@ZnPc, but add SPP@DOPA instead of Oxa(IV)@DOPA. Since SPP has low cytotoxicity, SPP@ZnPc was prepared to serve as a control coordination nanoparticle for single photodynamic therapy (PDT), as to investigate the synergistic effect of chemotherapy and PDT.

As calcein is a fluorescent dye, nanoparticles using Calcein to replace Oxa as the core have fluorescent properties, thus it could be detected by fluorescence imaging and fluorescence

spectroscopy. Using nanoparticles containing Calcein can be more convenient to investigate the cell uptake and drug release behavior of the cell-based drug delivery system. In the process of revising the manuscript, we used Cal@ZnPc as the model particle instead of Cal@DiI to explore the cell uptake behavior, lipid coated and ZnPc loaded Cal@ZnPc were prepared similar to Oxa(IV)@ZnPc, but add Cal@DOPA instead of Oxa(IV)@DOPA. In the revised manuscript, Cal@Lip was deleted and the characterization on Cal@ZnPc was added.

As shown in **Fig. R37a and b**, the average sizes of SPP@DOPA and Cal@DOPA detected by DLS were about 32 nm, and the average sizes of SPP@ZnPc and Cal@ZnPc were about 43 nm. Transmission electron microscopy (TEM) images of SPP@ZnPc and Cal@ZnPc were showed well-dispersed uniform spherical nanoparticles (**Fig. R37c**). Both SPP@ZnPc and Cal@ZnPc were showed near-neutral zeta potential at about -7 mV (**Fig. R37d**). The drug loading of Zn in SPP@ZnPc was detected by Inductively Coupled Plasma-Atomic Emission Spectrometry (ICP-AES, Optima 8300, Perkin–Elmer) and calculated to be about 14.7%, and the drug loading of Calcein in Cal@ZnPc was detected by fluorescence spectrometer and calculated to be about 28.1%. The UV-vis and fluorescence spectrum of SPP@ZnPc were similar to that of Oxa(IV)@ZnPc (**Fig. R37e-f**), suggesting that ZnPc was successfully loaded and finely dispersed in the lipid bilayer. The UV-vis and fluorescence spectrum of Cal@ZnPc showed the UV-vis and fluorescence characteristic peaks of calcein and ZnPc (**Fig. R37g-h**). All of these results suggested the successfully preparation of SPP@ZnPc and Cal@ZnPc.

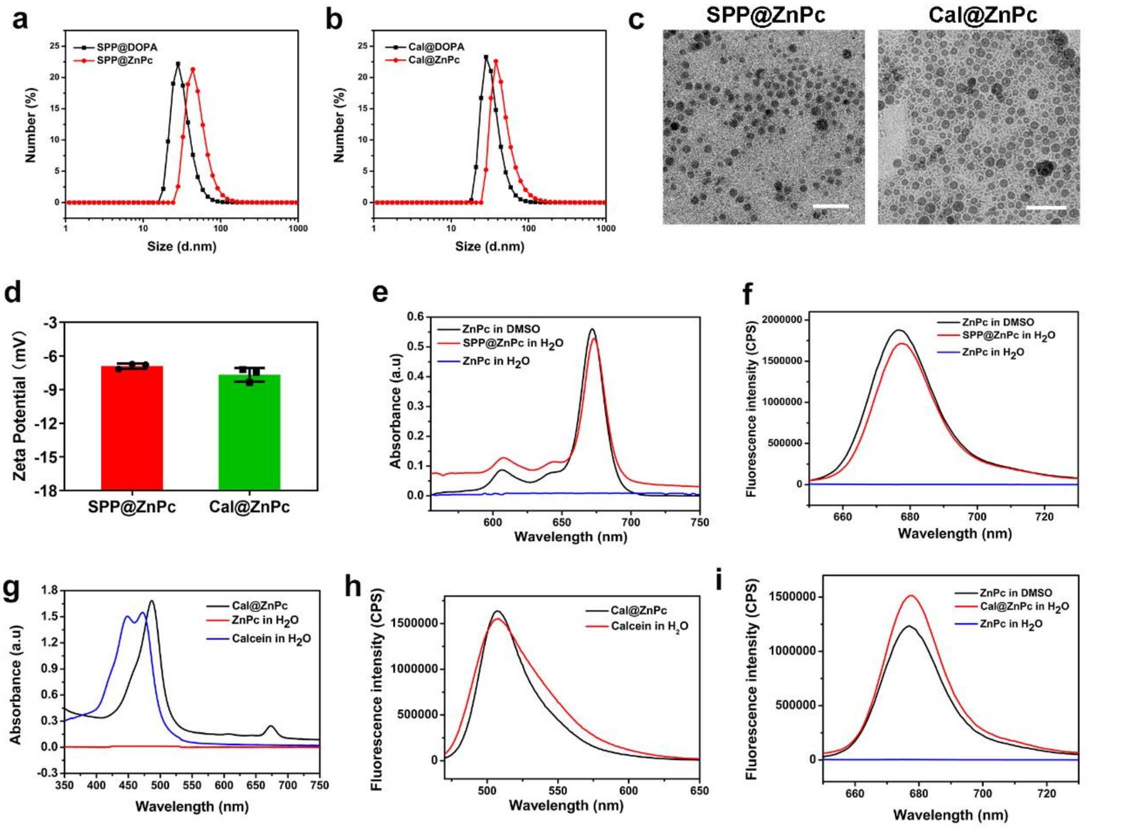

Fig. R37. Average diameters of (a) SPP@DOPA and SPP@ZnPc, and (b) Cal@DOPA and Cal@ZnPc measured by DLS. (c) TEM images of SPP@ZnPc and Cal@ZnPc without negative stain. Scale bars = 100 nm. (d) Zeta potential of SPP@ZnPc and Cal@ZnPc in H2O. (e) UV-vis spectrum and (f) Fluorescence emission spectrum of ZnPc in DMSO, ZnPc and SPP@ZnPc in H2O. (g) UV-vis spectrum and (h-i) Fluorescence emission spectrum of Cal@ZnPc in H_2O.

5. Comment: In Fig.S13d, how long was the cells incubated with various drugs? What does red and green color represents? Why are there less cells in the Oxa and Oxa(IV)@ZnPc groups?

Response: Thanks for reviewer's comment. Oxa, SPP@ZnPc and Oxa(IV)@ZnPc were first incubation with 4T1 cells for 24 h under dark, then drug media was replaced with fresh media, and the cells were irradiated or not with a LED light (671 nm, 20 mW cm^{-2}) for 5 min. After further incubation for 12 h, cells were incubated with fluorescein diacetate (FDA) and PI for 10 min to stain live and dead cells respectively, and then imaged by an inverted fluorescent microscope (IX73, Olympus, Japan).

Live cells can emit green fluorescence after being stained by FDA, and dead cells can emit red fluorescence after being stained by PI, thus red and green color represents the dead and live cells respectively. Oxa and Oxa(IV)@ZnPc groups without irradiation showed less

amount of live cells and a slightly increased dead cells compared with Control (+) group, this is because free Oxa and Oxa released from Oxa(IV)@ZnPc can inhibit tumor cell proliferation and kill tumor cells after treated for 36 h, and some dead cells are washed away in the process of washing off the dye before imaging.

Compared with Control group, Oxa(IV)@ZnPc (+) group showed less cells, this is because the released Oxa can inhibit tumor cell proliferation at the first incubation with 4T1 cells for 24 h under dark, after irradiated with light for 5 min, the ROS produced by PDT process killed tumor cells more faster than Oxa, thus Oxa(IV)@ZnPc (+) group showed more cells than Oxa(IV)@ZnPc, and they were almost dead cells. In the revised manuscript, we added a more detailed discussion in this section.

6. Comment: Please supplement the half-life and AUC results of the pharmacokinetics results in Fig.4a.

Response: Thanks for reviewer's comment. The half-life and area under curves (AUC) results of the pharmacokinetics were supplemented in the revised manuscript. As shown in table R1, Oxa(IV)@ZnPc@M showed about 3.9-fold longer blood clearance half-life ($t_{1/2}$) and 17.5-fold higher area under curves (AUC) compared with Oxa(IV)@ZnPc respectively.

Table R1. Pharmacokinetics parameter in SD rats after intravenous injection of free Oxa, Oxa(IV)@ZnPc and Oxa(IV)@ZnPc@M (n=3).

	Oxa	Oxa(IV)@ZnPc	Oxa(IV)@ZnPc@M
AUC (mg/L*h)	6.71 ±4.31	10.02 ±1.80	175.59 ±18.04
$t_{1/2}$ (h)	19.45 ±0.26	19.45 ±0.26	70.97 ±11.43

7. Comment: In the Introduction part (page 1, line 26), the authors describe the "therapeutics inside Oxa(IV)@ZnPc@M undergo spatiotemporally controlled release". How was "spatiotemporally controlled release" reflected?

Response: Thanks for reviewer's comment. The "spatiotemporally controlled release" intended to mean that the therapeutics inside Oxa(IV)@ZnPc@M can be released in a near-infrared light (NIR) triggered mode. As the irradiation time and location can be controlled by

outsiders, we think that the manner of NIR-triggering is a time and space controllable mode. In order to be more rigorous, we replaced the description with “therapeutics inside Oxa(IV)@ZnPc@M underwent NIR triggered release”.

8. Comment: In scheme 1, please speculate the name of each cell types.

Response: Thanks for reviewer’s comment. We have added the name of each cell types in scheme1 in the revised manuscript, thank you again for you kind remind.

9. Comment: The manuscript citations on Fig2, 3 and 4 are not in sequential. For example, Fig2f-I appears before Fig2d-e.

Response: Thanks for reviewer’s comment. We reformatted the pictures in Fig.2, Fig.3 and Fig.4, or made slight adjustments to the description order of the content in the main text to make the order of the content description consistent with the order of the pictures.

10. Comment: There are many grammar mistakes in the manuscript. Please carefully polish the whole manuscript.

Response: Thanks for reviewer’s comment. The entire manuscript was carefully checked and some writing and grammatical mistakes were corrected in the revised manuscript, and all the changes made in the manuscript were highlighted in red. We appreciate the reviewer so much to help us improve our manuscript.

11. Comment: Please give the full names of the abbreviations when they are firstly appeared in the manuscript.

Response: Thanks for reviewer’s comment. The above format errors were checked and revise in the revised manuscript, and all the changes made in the manuscript were highlighted in red. We appreciate the reviewer so much to help us improve our manuscript.

Reference:

1. Batrakova EV, Gendelman HE, Kabanov AV. Cell-mediated drug delivery. *Expert opinion on drug delivery* **8**, 415-433 (2011).

2. Agrahari V, Agrahari V, Mitra AK. Next generation drug delivery: circulatory cells-mediated nanotherapeutic approaches. (ed^(eds). Taylor & Francis (2017).

3. Sun X, Wang C, Gao M, Hu A, Liu Z. Remotely Controlled Red Blood Cell Carriers for Cancer Targeting and Near-Infrared Light-Triggered Drug Release in Combined Photothermal–Chemotherapy. *Advanced Functional Materials* **25**, 2386-2394 (2015).

4. Choi M-R, *et al.* A cellular Trojan Horse for delivery of therapeutic nanoparticles into tumors. *Nano letters* **7**, 3759-3765 (2007).

5. Zhang W, *et al.* Nanoparticle-Laden Macrophages for Tumor-Tropic Drug Delivery. *Advanced Materials* **30**, 1805557 (2018).

6. Shi C, Pamer EG. Monocyte recruitment during infection and inflammation. *Nature reviews immunology* **11**, 762 (2011).

7. Dong X, Chu D, Wang Z. Leukocyte-mediated delivery of nanotherapeutics in inflammatory and tumor sites. *Theranostics* **7**, 751 (2017).

8. Xing L, Cao Y, Che S. Synthesis of core–shell coordination polymer nanoparticles (CPNs) for pH-responsive controlled drug release. *Chemical Communications* **48**, 5995-5997 (2012).

9. Zheng H, Xing L, Cao Y, Che S. Coordination bonding based pH-responsive drug delivery systems. *Coordination Chemistry Reviews* **257**, 1933-1944 (2013).

10. Huang L, *et al.* Mild photothermal therapy potentiates anti-PD-L1 treatment for immunologically cold tumors via an all-in-one and all-in-control strategy. *Nature communications* **10**, 1-15 (2019).

11. Wang H, *et al.* Biomaterial-based scaffold for in situ chemo-immunotherapy to treat poorly immunogenic tumors. *Nature communications* **11**, 1-14 (2020).

12. Gujar S, Pol JG, Kroemer G. Heating it up: Oncolytic viruses make tumors ‘hot’and suitable for checkpoint blockade immunotherapies. (ed^(eds). Taylor & Francis (2018).

REVIEWERS' COMMENTS

Reviewer #1 (Remarks to the Author):

I am satisfactory with the revision. The only suggestion is that the authors need to incorporate all meaningful responses into the revised manuscript or supplementary information, since readers may have similar questions (of course, the authors may choose to publish their responses as supplementary information if accepted).

Reviewer #2 (Remarks to the Author):

Overall, the authors have sufficiently attempted to address all 4 reviewers concerns. I think that there are some that were not fully addressed, but they weren't in response to a comment that I made, so I will let the other reviewers address those.

• Scheme 1 remains slightly unclear. I think it is close to illustrating the overall picture, but some clarification would help. For example, what is meant by anti-primary and bone metastatic tumors in panel b? Also, I think the authors need to make it clearer where the figure "starts". My eyes were drawn to the center, but I think the real start is at the lower left where the nanoparticle is injected. The authors may want to consider rotating this to make it clearer.

• Some grammatical errors remain, but it is overall much improved. Some of the new next needs to be carefully reviewed. For example on Pg 21 (line 499) "The 4T1-tumor-bearing mice were received the same treatment procedure as antitumor studies (Fig. 6a)." You should remove "were" from this sentence.

• Pg 21 (line 515)"…9.99% CD8+ T cells and 19.0% CD4+ T Cells…" Some discussion or mention as to what % you would expect to see in an active T-cell response would help put this into context. I think this is still somewhat lower compared to some of the other anti-tumor models. I know these can be lower cell populations, so this may be a great response, but highlighting/clarifying this would be helpful to the reader.

• I am very happy with the addition of the EMT6 model and happy to see a similar response.

• The authors did some to address my concerns over toxicity as the macrophages induce tumor killing, but I would have liked to see a little more of this. I see that there isn't a specific discussion section, which is where I would normally suggest such things be added. Maybe you could address this in the toxicity section. I do appreciate the added Tox data.

Reviewer #3 (Remarks to the Author):

The authors have addressed my major concerns regarding inclusion of critical controls and additional in validation of mechanism of action.

The only remaining concern I have is that the number of independent experiments performed should be included in each of the figure legends.

Reviewer #4 (Remarks to the Author):

The authors have addressed all my concerns in the revised manuscript. I have no more questions.

Response to Reviewers

Dear Reviewers:

We appreciate you so much for your positive and constructive comments to our manuscript entitled "Engineered macrophages as light-activated drug vectors for chemo/photo/immunotherapy of primary and bone metastatic breast cancer" (**ID: NCOMMS-20-28585B**)". These comments are all valuable and very helpful for revising and improving our paper. All the changes made to the manuscript were highlighted in red, and the point to point response to your comments are listed as follows.

Responses to the comments of Reviewers

Reviewer #1 (Remarks to the Author):

Comment: I am satisfactory with the revision. The only suggestion is that the authors need to incorporate all meaningful responses into the revised manuscript or supplementary information, since readers may have similar questions (of course, the authors may choose to publish their responses as supplementary information if accepted).

Response: We appreciate the reviewer's positive comments. Most of the meaningful results in the responses letters have been added to the revised manuscript or supporting information, and we will publish the responses letters as supplementary peer review file if accepted.

Reviewer #2 (Remarks to the Author):

Overall, the authors have sufficiently attempted to address all 4 reviewers concerns. I think that there are some that were not fully addressed, but they weren't in response to

a comment that I made, so I will let the other reviewers address those.

1. Comment: Scheme 1 remains slightly unclear. I think it is close to illustrating the overall picture, but some clarification would help. For example, what is meant by anti-primary and bone metastatic tumors in panel b? Also, I think the authors need to make it clearer where the figure “starts”. My eyes were drawn to the center, but I think the real start is at the lower left where the nanoparticle is injected. The authors may want to consider rotating this to make it clearer.

Response: Thanks for reviewer’s comment. Anti-primary and bone metastatic tumors meant that Oxa(IV)@ZnPc@M mediated chemo-photodynamic therapy combined with PD-L1 blockade can inhibit primary tumor and bone metastatic tumor simultaneously. To make Scheme 1 (Fig 1 in the revised manuscript) more clear and avoid misunderstanding, we numbered each process in Fig 1 and gave a brief description in the legend, thank you again for your constructive suggestions.

2. Comment: Some grammatical errors remain, but it is overall much improved. Some of the new next needs to be carefully reviewed. For example on Pg 21 (line 499) “The 4T1-tumor-bearing mice were received the same treatment procedure as antitumor studies (Fig. 6a).” You should remove “were” from this sentence.

Response: Thanks for reviewer’s comment. The entire manuscript has been carefully checked again for grammar issues, ant the manuscript has been proofed by a native speaker.

3. Comment: Pg 21 (line 515) “…9.99% CD8+ T cells and 19.0% CD4+ T Cells…” Some discussion or mention as to what % you would expect to see in an active T-cell response would help put this into context. I think this is still somewhat lower compared to some of the other anti-tumor models. I know these can be lower cell populations, so this may be a great response, but highlighting/clarifying this would be helpful to the reader.

Response: Thanks for reviewer’s comment. We calculated the ratio of CD4+ T

cells and CD8+ T cells in tumor tissue, not in CD3 positive cells, so the population is relatively low, but in order to avoid misleading of the readers, we have changed the description of this sentence, thank you for your constructive suggestions.

4. Comment: I am very happy with the addition of the EMT6 model and happy to see a similar response.

Response: We appreciate the reviewer's positive comments.

5. Comment: The authors did some to address my concerns over toxicity as the macrophages induce tumor killing, but I would have liked to see a little more of this. I see that there isn't a specific discussion section, which is where I would normally suggest such things be added. Maybe you could address this in the toxicity section. I do appreciate the added Tox data.

Response: Thanks for reviewer's comment. We performed whole blood analysis and biochemical indexes (AST, ALT, BUN, CREA) detection, as well as the histopathological analysis of major organs including heart, liver, spleen, lung and kidney no matter single-dose and multi-dose injections, in order to evaluate the potential toxicity of the current cell-based formulations and treatment models. Now we added a specific discussion section in the revised manuscript, and a more detailed discussion of the toxicity of the current treatment models were added. Thank you again for your constructive suggestions.

Reviewer #3 (Remarks to the Author):

Comment: The authors have addressed my major concerns regarding inclusion of critical controls and additional in validation of mechanism of action. The only remaining concern I have is that the number of independent experiments performed should be included in each of the figure legends.

Response: We thank the reviewer for their positive feedback. We have now added

the independent experiments preformed in the figure legends according to the editorial indications.

Reviewer #4 (Remarks to the Author):

Comment: The authors have addressed all my concerns in the revised manuscript. I have no more questions.

Response: We appreciate the reviewer's positive comments.
